# First-line talazoparib with enzalutamide in HRR-deficient metastatic castration-resistant prostate cancer: the phase 3 TALAPRO-2 trial

**DOI:** 10.1038/s41591-023-02704-x

**Published:** 2023-12-04

**Authors:** Karim Fizazi, Arun A. Azad, Nobuaki Matsubara, Joan Carles, Andre P. Fay, Ugo De Giorgi, Jae Young Joung, Peter C. C. Fong, Eric Voog, Robert J. Jones, Neal D. Shore, Curtis Dunshee, Stefanie Zschäbitz, Jan Oldenburg, Dingwei Ye, Xun Lin, Cynthia G. Healy, Nicola Di Santo, A. Douglas Laird, Fabian Zohren, Neeraj Agarwal

**Affiliations:** 1 Institut Gustave Roussy, University of Paris-Saclay, Villejuif, France; 2 Peter MacCallum Cancer Centre, Melbourne, Victoria Australia; 3 National Cancer Center Hospital East, Chiba, Japan; 4 Vall d’Hebron University Hospital, Vall d’Hebron Institute of Oncology (VHIO), Barcelona, Spain; 5 PUCRS School of Medicine, Porto Alegre, Brazil; 6 IRCCS Istituto Romagnolo per lo Studio dei Tumori (IRST) Dino Amadori, Meldola, Italy; 7 National Cancer Center, Goyang, Republic of Korea; 8 Auckland City Hospital, Auckland, New Zealand; 9 University of Auckland, Auckland, New Zealand; 10 Clinique Victor Hugo Centre Jean Bernard, Le Mans, France; 11 School of Cancer Sciences, University of Glasgow, Beatson West of Scotland Cancer Centre, Glasgow, UK; 12 Carolina Urologic Research Center, Myrtle Beach, SC USA; 13 Arizona Urology Specialists, Tucson, AZ USA; 14 National Center for Tumor Diseases (NCT), Heidelberg University Hospital, Heidelberg, Germany; 15 Akershus University Hospital (Ahus), Lørenskog, Norway; 16 Fudan University Shanghai Cancer Center, Shanghai, China; 17 Pfizer Inc., La Jolla, CA USA; 18 Pfizer Inc., Collegeville, PA USA; 19 Pfizer Inc., Durham, NC USA; 20 Pfizer Inc., New York, NY USA; 21 Huntsman Cancer Institute (NCI-CCC), University of Utah, Salt Lake City, UT USA

**Keywords:** Prostate cancer, Prostate cancer

## Abstract

Preclinical evidence has suggested an interplay between the androgen receptor, which largely drives the growth of prostate cancer cells, and poly(ADP-ribose) polymerase. This association provides a rationale for their co-inhibition for the treatment of metastatic castration-resistant prostate cancer (mCRPC), an area of unmet medical need. The phase 3 TALAPRO-2 study investigated combining the poly(ADP-ribose) polymerase inhibitor talazoparib with enzalutamide versus enzalutamide alone as first-line treatment of mCRPC. Patients were prospectively assessed for tumor alterations in DNA damage response genes involved in homologous recombination repair (HRR). Two cohorts were enrolled sequentially: an all-comers cohort that was enrolled first (cohort 1; *N* = 805 (169 were HRR-deficient)), followed by an HRR-deficient-only cohort (cohort 2; *N* = 230). We present results from the alpha-controlled primary analysis for the combined HRR-deficient population (*N* = 399). Patients were randomized in a 1:1 ratio to talazoparib or placebo, plus enzalutamide. The primary endpoint, radiographic progression-free survival, was met (median not reached at the time of the analysis for the talazoparib group versus 13.8 months for the placebo group; hazard ratio, 0.45; 95% confidence interval, 0.33 to 0.61; *P* < 0.0001). Data for overall survival, a key secondary endpoint, are immature but favor talazoparib (hazard ratio, 0.69; 95% confidence interval, 0.46 to 1.03; *P* = 0.07). Common adverse events in the talazoparib group were anemia, fatigue and neutropenia. Combining talazoparib with enzalutamide significantly improved radiographic progression-free survival in patients with mCRPC harboring HRR gene alterations, supporting talazoparib plus enzalutamide as a potential first-line treatment for these patients. ClinicalTrials.gov Identifier: NCT03395197.

## Abstract (web summary)

In the homologous recombination repair-deficient population of TALAPRO-2, a placebo-controlled phase 3 trial, treatment of patients with metastatic castration-resistant prostate cancer with the poly(ADP-ribose) polymerase inhibitor talazoparib plus enzalutamide led to prolonged progression-free survival compared with placebo plus enzalutamide.

## Main

Recent approvals of new treatments have led to improved outcomes for patients with advanced prostate cancer^1,2^. However, metastatic disease remains aggressive and progression is inevitable, necessitating additional therapies for this population of often elderly men^1,3,4^. Around a quarter of advanced prostate cancers have alterations in DNA damage response genes involved directly or indirectly in homologous recombination repair (HRR), including *BRCA1/BRCA2* (refs. ^5–8^); these can sensitize tumors to treatment with poly(ADP-ribose) polymerase (PARP) inhibitors^9–14^. PARP inhibition as monotherapy is an established standard of care for those patients with late-stage prostate cancer.

Preclinical evidence suggests interplay between the androgen receptor, which largely drives the growth of prostate cancer cells, and PARP, providing a rationale for their co-inhibition^15,16^. Androgen receptor inhibition is associated with upregulated PARP activity and downregulated HRR gene expression^17,18^, while PARP inhibition suppresses androgen receptor transcriptional activity^19–21^.

**Extended Data Fig. 1:**
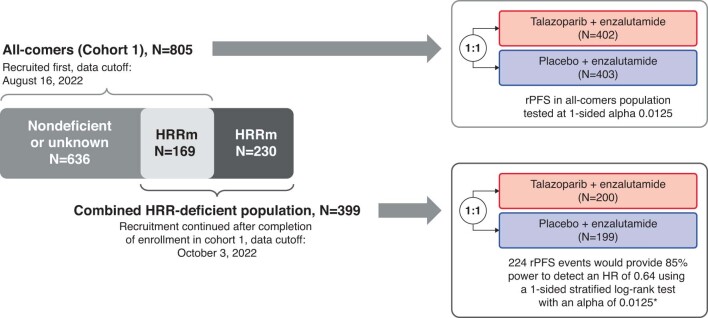
Study Cohorts and Enrollment. *An interim analysis (IA) was planned with ~70% of the total required events. The HRRm cohort would be stopped for efficacy if the pre-specified efficacy boundary was crossed (P ≤ 0.003). As the efficacy boundary was crossed at the IA rPFS, this became the final analysis. Survival and safety follow-up is continuing. All other endpoints are final. HRR denotes homologous recombination repair, HRRm HRR mutation-positive, and rPFS radiographic progression-free survival.

Monotherapy with the PARP inhibitor talazoparib (1 mg per day) showed durable antitumor activity and a favorable benefit–risk profile in patients with heavily pretreated mCRPC with HRR gene alterations in the phase 2, TALAPRO-1 study^22^. TALAPRO-2 is a multinational phase 3 study evaluating talazoparib in combination with the androgen receptor inhibitor enzalutamide as a first-line treatment in patients with mCRPC^23^. An initial, non-randomized, open-label run-in study (part 1; *N* = 19) showed that when combined with enzalutamide at 160 mg per day, similar talazoparib exposure levels to the recommended monotherapy dose were achieved at 0.5 mg per day, establishing this as the starting dose for the combination^23,24^. Patients were then enrolled sequentially in two cohorts: unselected (cohort 1, all-comers cohort, recruited first) for alterations in DNA damage response genes directly or indirectly involved in HRR and selected (cohort 2) to ensure exclusive enrollment of patients with HRR-deficient disease. The first 805 patients with (*N* = 169) and without (*N* = 636) HRR gene alterations were enrolled as all-comers in cohort 1. Subsequently, an additional 230 patients selected for HRR gene alterations were recruited to complete the predefined enrollment for a combined HRR-deficient population (*N* = 399; Extended Data Fig. 1). All patients were prospectively tested for HRR gene alterations^23^.

A recent analysis of the all-comers population of TALAPRO-2 revealed significant improvement in radiographic progression-free survival (rPFS) for talazoparib plus enzalutamide compared with enzalutamide as standard of care (hazard ratio (HR), 0.63; 95% confidence interval (CI), 0.51 to 0.78; *P* < 0.0001)^25^. Here, we report results of the prespecified alpha-powered independent analysis for the combined HRR-deficient population from both cohorts of TALAPRO-2.

## Results

### Patients

**Fig. 1:**
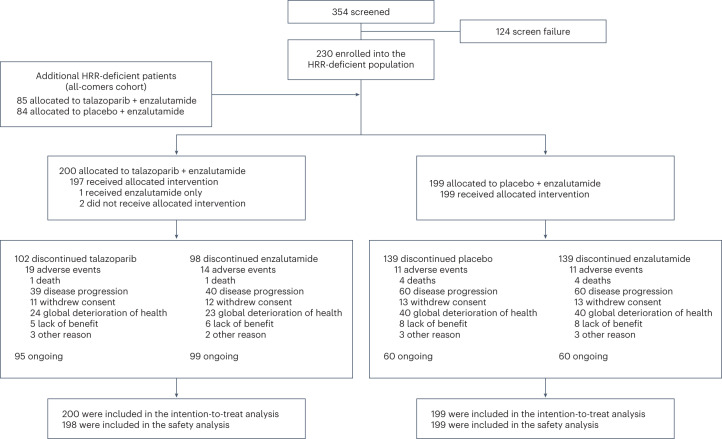
Trial profile. Flow diagram showing participant recruitment into the HRR-deficient population, randomization, follow-up and analysis populations.

Between 18 December 2018 and 20 January 2022, 399 patients with HRR gene alterations were enrolled (169 enrolled during the accrual of the all-comers cohort; 230 additional patients to complete the planned accrual target of the combined HRR-deficient population; Fig. 1). Of the 399 enrolled patients, 397 had available prospective tumor tissue test results. Of these, 236 patients with central laboratory, nonhistorical tissue records also had blood samples that underwent concurrent prospective circulating tumor DNA testing after a protocol amendment (26 February 2020). The remaining 2 of 399 patients were enrolled based on circulating tumor DNA testing alone (*n* = 1) or had unspecified tissue source (*n* = 1).

The data cutoff date for the HRR-deficient cohort was 3 October 2022. Baseline characteristics were well balanced (Table 1 and Extended Data Table 1); representativeness of the patients is addressed in Extended Data Table 2. The most commonly altered HRR genes were *BRCA2, ATM* and *CDK12*.

**Table 1:** Summary of baseline characteristics (HRR-deficient intention-to-treat population)

Characteristic	Talazoparib + enzalutamide (*N* = 200)	Placebo + enzalutamide (*N* = 199)
Median age (range)—years	70 (41–90)	71 (44–90)
Race		
White	137 (68)	136 (68)
Black or African American	6 (3)	5 (3)
Asian	45 (22)	39 (20)
Multiracial	0	1 (<1)
Other^a^	1 (<1)	1 (<1)
Not reported or unknown	11 (6)	17 (9)
Median baseline serum PSA (range)—µg l^−1^	19.6 (0.2–3412.0)	18.0 (0.0–1055.0)
Gleason score^b^		
<8	42 (21)	52 (26)
≥8	152 (76)	143 (72)
Disease site		
Bone (including with soft tissue component)	175 (88)	158 (79)
Lymph node	82 (41)	94 (47)
Visceral (lung)	23 (12)	26 (13)
Visceral (liver)	9 (4)	6 (3)
Other soft tissue	23 (12)	20 (10)
ECOG performance status		
0	128 (64)	118 (59)
1	72 (36)	81 (41)
Prior treatment with a second-generation androgen receptor pathway inhibitor	17 (9)	17 (9)
Abiraterone	16 (8)	16 (8)
Orteronel	1 (<1)	1 (<1)
Prior taxane-based chemotherapy^c^	57 (28)	60 (30)
Patients with at least one alteration in corresponding HRR gene^d^	198 (99)	197 (99)
* ATM*	47 (24)	39 (20)
* ATR*	3 (2)	12 (6)
* BRCA1*	11 (6)	12 (6)
* BRCA2*	62 (31)	73 (37)
* CDK12*	36 (18)	39 (20)
* CHEK2*	34 (17)	37 (19)
* FANCA*	4 (2)	5 (3)
* MLH1*	9 (4)	1 (<1)
* MRE11A*	1 (<1)	2 (1)
* NBN*	8 (4)	3 (2)
* PALB2*	9 (4)	8 (4)
* RAD51C*	2 (1)	2 (1)

Data are *n* (%), unless otherwise indicated.

^a^American Indian, Alaska Native, Native Hawaiian or Other Pacific Islander.

^b^Not reported for the remaining patients.

^c^All received docetaxel; HRR-deficient safety population.

^d^*N* = 3 patients (1, talazoparib plus enzalutamide; 2, placebo plus enzalutamide) did not have HRR gene alterations and 1 patient in the talazoparib group was of unknown HRR gene alteration status.

ECOG, Eastern Cooperative Oncology Group.

### Efficacy

**Fig. 2:**
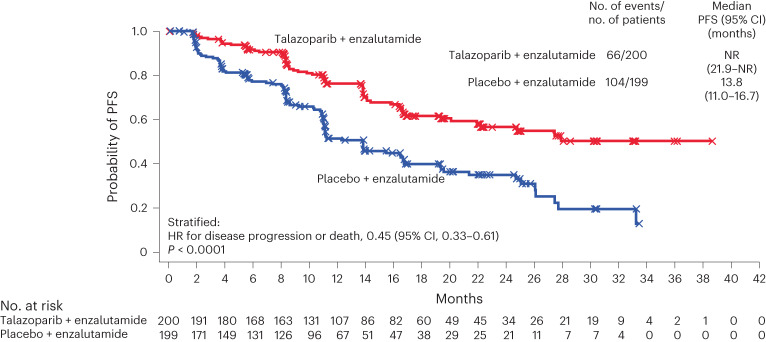
rPFS in patients with any HRR gene alteration (assessed by blinded independent central review; HRR-deficient intention-to-treat population). rPFS was compared between treatment groups using stratified log-rank test. HRs and associated 95% two-sided CIs were estimated by a Cox proportional hazards model. Median time to event was estimated by the Kaplan–Meier method, and 95% CIs were based on the Brookmeyer–Crowley method. The *P* value is two-sided. NR, not reached at the time of the analysis.

Median follow-up for rPFS was 17.5 and 16.8 months for the talazoparib and placebo groups, respectively. Talazoparib plus enzalutamide significantly improved rPFS by blinded independent central review compared with placebo plus enzalutamide (HR, 0.45; 95% CI, 0.33 to 0.61; *P* < 0.0001; median not reached at the time of the analysis versus 13.8 months; Fig. 2).

**Fig. 3:**
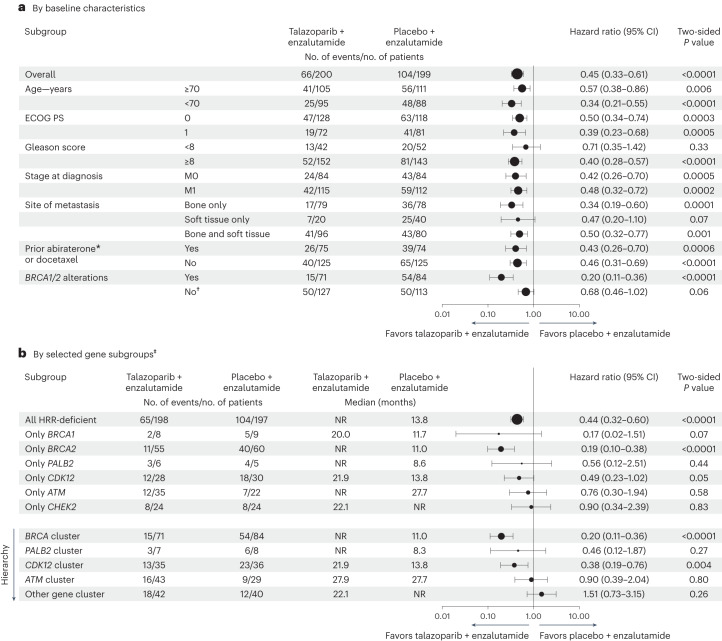
Subgroup analysis of rPFS. **a**,**b**, Subgroup analysis of rPFS by baseline characteristics (**a**) and by gene subgroups (**b**) (assessed by blinded independent central review; HRR-deficient intention-to-treat population). The overall HR for all patients, and by *BRCA1/BRCA2* alteration status, was based on a Cox proportional hazards model stratified by the randomization stratification factors. For all other subgroups, the HR was based on an unstratified Cox model with treatment as the only covariate. Data are presented as HRs with two-sided 95% CIs. *P* values are two sided. The asterisk indicates the inclusion of one patient in each treatment arm who received prior orteronel. †Excludes four patients who did not have HRR gene alterations but were incorrectly randomized to the HRR-deficient population; including these patients resulted in an HR of 0.72 (95% CI, 0.49 to 1.07) for the non-*BRCA* alterations subgroup. ‡Post hoc exploratory analysis; as this analysis was underpowered, the data are hypothesis-generating and should be interpreted with caution. Gene clustering alteration dominance hierarchy is any *BRCA1/BRCA2* alteration (*BRCA* cluster), then any *PALB2* (*PALB2* cluster), next any *CDK12* (*CDK12* cluster), then any *ATM* (*ATM* cluster), and finally, any of all other genes (with each patient counted only once). For the single-gene subgroups, only patients bearing alteration(s) in the designated HRR gene and none of the other HRR genes tested are shown, with a prevalence cutoff for display of ≥10 across arms. PS, performance status.

**Extended Data Fig. 2:**
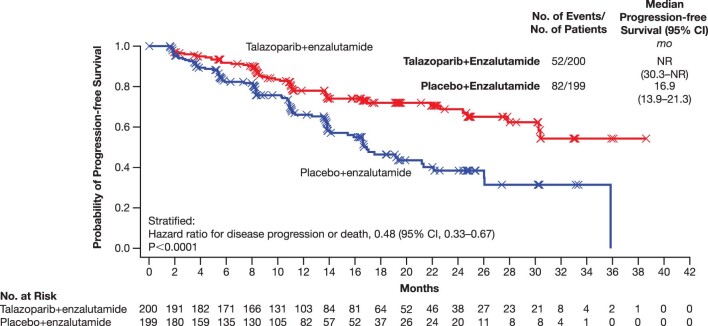
Investigator-Assessed rPFS (HRR-Deficient Intention-to-Treat Population). rPFS was compared between treatment groups using stratified log-rank test. HRs and associated 95% two-sided CIs were estimated by a Cox proportional hazards model. Median time to event was estimated by the Kaplan-Meier method, and 95% CIs based on the Brookmeyer-Crowley method. The P value is two-sided. CI denotes confidence interval, HR hazard ratio, HRR homologous recombination repair, NR not reached at the time of the analysis, and rPFS radiographic progression-free survival.

A consistent treatment effect for rPFS was observed across prespecified clinical subgroups (Fig. 3a) and by investigator assessment (Extended Data Fig. 2). Among 149 patients who had received prior abiraterone or orteronel (a CYP17 inhibitor) or docetaxel for castration-sensitive disease, the HR was 0.43 (95% CI, 0.26 to 0.70; *P* = 0.0006) in favor of talazoparib plus enzalutamide. Among patients who had received abiraterone or orteronel (*n* = 34), the HR was 0.53 (95% CI, 0.20 to 1.42; *P* = 0.20), and among those who had received docetaxel (*n* = 117), the HR was 0.39 (95% CI, 0.22 to 0.69; *P* = 0.0008).

In a post hoc analysis, patients with *BRCA1/BRCA2* alterations had an 80% lower risk of radiographic progression or death (HR, 0.20; 95% CI, 0.11 to 0.36; *P* < 0.0001; Fig. 3a); those with non-*BRCA1/BRCA2* alterations had a 32% lower risk (HR, 0.68; 95% CI, 0.46 to 1.02; *P* = 0.06) with talazoparib plus enzalutamide. Further, notable improvements in rPFS were observed with talazoparib plus enzalutamide in the *BRCA2* single-gene subgroup, and in *BRCA* and *CDK12* clusters (Fig. 3b).

**Fig. 4:**
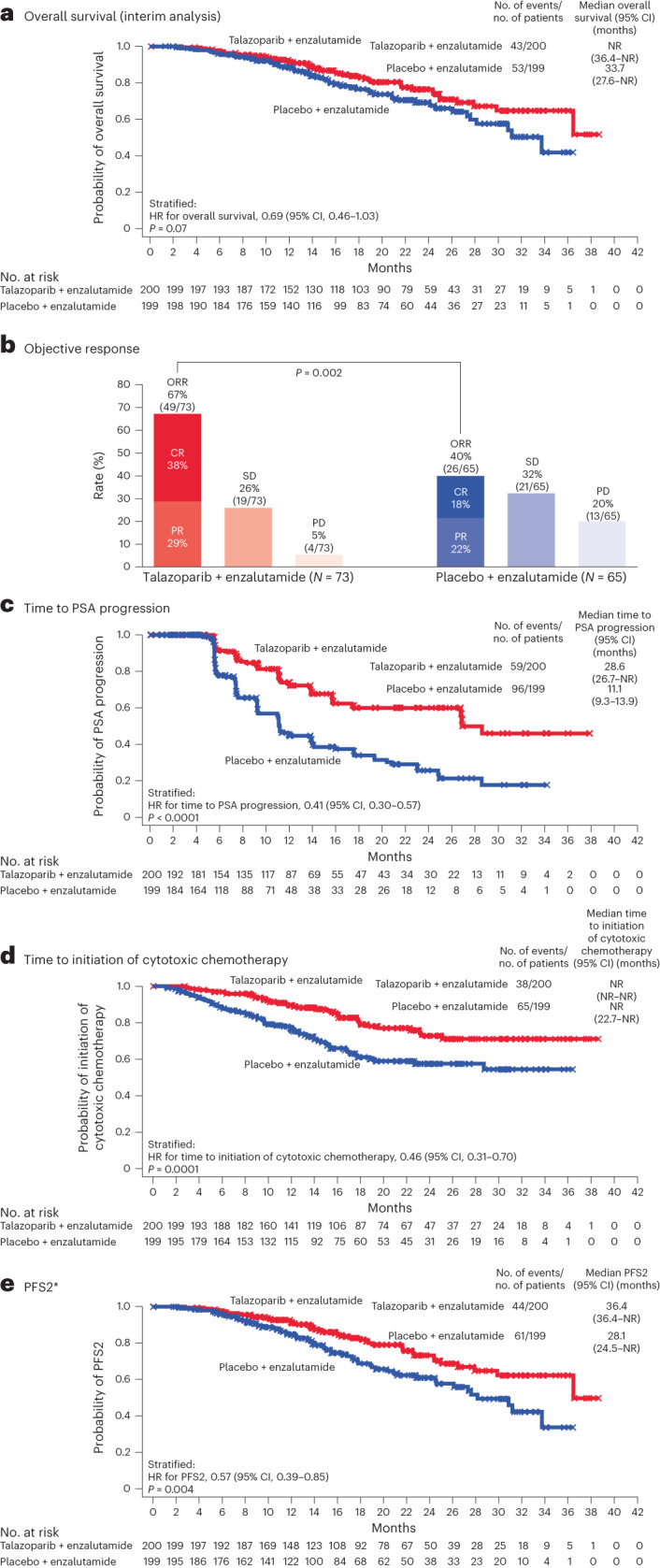
Secondary efficacy endpoints. **a**–**e**, Secondary efficacy endpoints: overall survival (**a**), objective response (**b**), time to PSA progression (**c**), time to initiation of cytotoxic chemotherapy (**d**) and PFS2 (**e**) (HRR-deficient intention-to-treat population). Time-to-event endpoints were compared between treatment groups using a stratified log-rank test. HRs and associated 95% two-sided CIs were estimated by a Cox proportional hazards model. Median time-to-event endpoints were estimated by the Kaplan–Meier method, and 95% CIs were based on the Brookmeyer–Crowley method. *P* values are two sided. The asterisk denotes that PFS2 was based on investigator assessment (time from randomization to the date of documented progression on the first subsequent antineoplastic therapy or death from any cause, whichever occurs first). CR, complete response; ORR, objective response rate; PD, progressive disease; PFS2, progression-free survival 2; PR, partial response; SD, stable disease.

Overall survival data remain immature, with the majority of patients being alive: 43 (22%) patients in the talazoparib group and 53 (27%) in the placebo group had died at data cutoff. Three patients in the talazoparib group and 18 in the placebo group subsequently received a PARP inhibitor (all received olaparib) per the treating physician’s judgment and local approval and availability of a PARP inhibitor. The HR for death was 0.69 (95% CI, 0.46 to 1.03; *P* = 0.07; Fig. 4a). In the *BRCA1/BRCA2* and non-*BRCA1/BRCA2* altered subgroups, the HRs for death were 0.61 (95% CI, 0.31 to 1.23; *P* = 0.16) and 0.66 (95% CI, 0.40 to 1.10; *P* = 0.11), respectively.

Confirmed objective response rate in patients with measurable disease at baseline was 67% (49/73; 95% CI, 55.1% to 77.7%) for the talazoparib group and 40% (26/65; 95% CI, 28.0% to 52.9%) for the placebo group (Fig. 4b). Time to prostate-specific antigen (PSA) progression, time to initiation of cytotoxic chemotherapy and investigator-assessed time to progression or death on first subsequent antineoplastic therapy were significantly prolonged in the talazoparib group (Fig. 4c–e; see Extended Data Table 3 for results of other secondary efficacy endpoints).

### Safety

Median duration of treatment was 14.6 months for talazoparib and 14.7 months for enzalutamide in the talazoparib group, and 12.0 months for placebo and 12.1 months for enzalutamide in the placebo group. Median relative dose intensities in the talazoparib group were 81% for talazoparib and 100% for enzalutamide; 10% of the talazoparib group had moderate renal impairment at baseline requiring a starting dose of talazoparib of 0.35 mg per day.

The most common adverse events in the talazoparib group were anemia, fatigue, neutropenia, thrombocytopenia, nausea and decreased appetite. In the placebo group, fatigue, back pain and arthralgia were the most common adverse events (Table 2). The most common grade ≥3 adverse event in the talazoparib group was anemia (41%; Table 2), with a median time to onset of 3.3 months, and requiring dose modification of talazoparib according to the protocol. Thirty-six percent of patients in the talazoparib group received a packed red blood cell transfusion. At baseline, 56% of patients in the talazoparib group had grade 1–2 anemia. Only 4% of patients in the talazoparib group discontinued talazoparib due to anemia.

**Table 2:** Summary of treatment-emergent adverse events (HRR-deficient safety population)^a^

	Talazoparib + enzalutamide (*N* = 198)		Placebo + enzalutamide (*N* = 199)	
Adverse event	All grades	Grade ≥3	All grades	Grade ≥3
Any adverse event	196 (99)	134 (68)	191 (96)	79 (40)
Treatment-related adverse event	180 (91)	105 (53)	144 (72)	28 (14)
Serious adverse event	60 (30)	54 (27)	40 (20)	32 (16)
Serious and treatment-related adverse event	27 (14)	23 (12)	0	0
Adverse event resulting in dose interruption of:				
Talazoparib/placebo^b^	114 (58)	**·**	34 (17)	**·**
Enzalutamide^c^	67 (34)	**·**	31 (16)	**·**
Adverse event resulting in dose reduction of:				
Talazoparib/placebo^b^	103 (52)	**·**	11 (6)	**·**
Enzalutamide^c^	28 (14)	**·**	12 (6)	**·**
Adverse event resulting in permanent drug discontinuation of:				
Talazoparib/placebo^b^	20 (10)	**·**	14 (7)	**·**
Enzalutamide^c^	15 (8)	**·**	14 (7)	**·**
Grade 5 adverse event	3 (2)^d^	**·**	5 (3)^d^	**·**
Most common adverse events (all grades in ≥10% of patients)^e^				
Anemia	128 (65)	81 (41)	31 (16)	9 (5)
Fatigue	66 (33)	3 (2)	53 (27)	2 (1)
Neutropenia	64 (32)	37 (19)	13 (7)	2 (1)
Thrombocytopenia	49 (25)	14 (7)	5 (3)	1 (<1)
Nausea	41 (21)	3 (2)	34 (17)	1 (<1)
Decreased appetite	40 (20)	2 (1)	28 (14)	2 (1)
Back pain	39 (20)	3 (2)	44 (22)	2 (1)
Leukopenia	37 (19)	11 (6)	15 (8)	0
Hypertension	36 (18)	16 (8)	38 (19)	16 (8)
Asthenia	31 (16)	4 (2)	29 (15)	0
Constipation	26 (13)	0	33 (17)	0
Fall	26 (13)	4 (2)	24 (12)	3 (2)
Arthralgia	25 (13)	0	44 (22)	0
Diarrhea	24 (12)	0	22 (11)	0
Hot flush	23 (12)	0	28 (14)	0
Dizziness	20 (10)	1 (<1)	15 (8)	2 (1)
Headache	12 (6)	0	22 (11)	1 (<1)

Data are *n* (%).

^a^Shown are adverse events that occurred from the time of the first dose of study treatment through 28 d after permanent discontinuation of all study treatments or before initiation of a new antineoplastic or any investigational therapy, whichever occurs first. Adverse events were graded according to National Cancer Institute Common Terminology Criteria for Adverse Events version 4.03. All data are reported per the safety population defined as all patients who were treated with at least one dose of study treatment, including one patient who was randomized to talazoparib plus enzalutamide but received enzalutamide only (patients treated with both study treatments: *N* = 197 for talazoparib plus enzalutamide; *N* = 199 for placebo plus enzalutamide).

^b^Includes permanent discontinuation/dose reduction/dose interruption of talazoparib/placebo only plus permanent discontinuation/dose reduction/dose interruption of both talazoparib/placebo and enzalutamide.

^c^Includes permanent discontinuation/dose reduction/dose interruption of enzalutamide only plus permanent discontinuation/dose reduction/dose interruption of both talazoparib/placebo and enzalutamide.

^d^None were considered treatment related.

^e^None of these events were recorded as grade 5.

There were more dose interruptions and reductions due to adverse events in the talazoparib group than in the placebo group, but permanent discontinuation rates were similar (discontinuation of talazoparib in 10% versus placebo in 7% of patients; discontinuation of enzalutamide in 8% versus 7%; Table 2).

After a median follow-up for safety of 15.4 and 12.9 months for the talazoparib and placebo groups, respectively, no cases of myelodysplastic syndrome or acute myeloid leukemia were reported. Venous embolic and thrombotic events were reported in seven patients in the talazoparib group and two patients in the placebo group. There were four cases of pulmonary embolism (one grade 2, three grade 3) in the talazoparib group and two cases (both grade 3) in the placebo group. There were no treatment-related deaths.

## Discussion

In one of the largest studies in patients with mCRPC with HRR gene alterations, the prospectively defined, alpha-controlled primary outcome in the combined HRR-deficient population showed that talazoparib plus enzalutamide resulted in a clinically meaningful and statistically significant 55% reduction in risk of progression or death versus placebo plus enzalutamide as first-line treatment. These results build on the previous subgroup analysis of the HRR-deficient patients in the all-comers cohort, which showed a 54% reduction in risk of progression or death for talazoparib plus enzalutamide versus placebo plus enzalutamide (HR, 0.46; 95% CI, 0.30 to 0.70; *P* = 0.0003)^25^. Although overall survival data are immature and statistical significance was not reached, interim data favor this combination. Other key secondary endpoints, including time to PSA progression, time to cytotoxic chemotherapy and time to progression or death on the first subsequent antineoplastic therapy, favored the talazoparib group.

Importantly, TALAPRO-2 was not enriched for patients with *BRCA1/BRCA2* alterations, which were well balanced between the treatment arms (talazoparib group, 36%; placebo group, 42%); the observed *BRCA1/BRCA2* prevalence in the prospectively determined HRR-deficient population was in line with previous reports^5,7,8^. This is notable since *BRCA* alterations are a strong predictive factor toward improved treatment outcomes for patients receiving PARP inhibitor monotherapy^26^. Talazoparib plus enzalutamide reduced risk of progression or death by 80% in the *BRCA1/BRCA2* subgroup and by 32% in the non-*BRCA1/BRCA2* subgroup. The *CDK12* results are striking given an alteration prevalence of 5% to 7% according to the literature and that limited clinical data indicate poor prognosis with minimal benefit from PARP inhibitor monotherapy in patients who have prostate cancer and *CDK12* alterations^27^. *CDK12* deficiency is associated with a distinct chromosomal damage signature and disrupted replication and transcription^28,29^, perhaps resulting in vulnerability to the combination of enzalutamide and talazoparib, a potent PARP trapper^30^. Although patient numbers were very small and the CIs wide, a similar benefit to that seen with *BRCA2* was also apparent in the *BRCA1* single-gene subgroup, with a smaller benefit apparent in the *PALB2* cluster. However, these post hoc analyses were underpowered and hypothesis generating, so the results should be interpreted with caution.

Two other recent phase 3 trials have explored the combination of PARP inhibitors and the androgen biosynthesis inhibitor abiraterone acetate/prednisone as first-line treatment for mCRPC. The PROpel (NCT03732820) trial, which enrolled all-comers without prospective assessment of *BRCA* or HRR status, demonstrated improved rPFS with the combination of olaparib plus abiraterone versus placebo plus abiraterone for patients with HRR gene alterations (HR, 0.50; 95% CI, 0.34 to 0.73)^31^. Exploratory analysis in the *BRCA1/BRCA2* subgroup showed an HR of 0.24 (95% CI, 0.12 to 0.45)^32^. The MAGNITUDE (NCT03748641) trial also showed improved rPFS with the combination of niraparib plus abiraterone versus placebo plus abiraterone for patients with HRR gene alterations (HR, 0.73; 95% CI, 0.56 to 0.96), with particular benefit in the *BRCA1/BRCA2* subgroup (HR, 0.53; 95% CI, 0.36 to 0.79)^33^. Exploratory single-gene analysis of the MAGNITUDE trial, although underpowered, showed potential benefit of combined PARP and androgen receptor inhibition in patients with a Fanconi anemia pathway gene alteration (*PALB2*, *BRIP1* and *FANCA*) beyond *BRCA1/BRCA2* (ref. ^34^), whereas a lack of differential benefit was seen in tumors with *CDK12* alterations^34^. Results from the PROfound (NCT02987543)^13^ and TRITON-3 (NCT02975934)^14^ phase 3 trials of PARP monotherapy (olaparib and rucaparib, respectively) in patients with pretreated mCRPC also indicated that patients with *BRCA2* alterations derived benefit. There was inconclusive evidence supporting *BRCA1* due to small patient numbers, preliminary positive evidence for *CDK12* in PROfound (HR below 1 but wide CIs) and lack of efficacy with *ATM*^13,14^.

The main limitations of this study are due to the rapidly changing treatment landscape for patients with mCRPC. For example, the use of PARP inhibitors as a subsequent therapy was limited to a small number of patients (3 in the talazoparib group and 18 in the placebo group; all received olaparib). This small number most likely reflects the limited availability of PARP inhibitors for the treatment of mCRPC when the TALAPRO-2 study was carried out. Based on established phase 3 data^35^, it is anticipated that survival in the placebo group in those who did not receive a subsequent PARP inhibitor may be shorter than in those who did. Also, the use of androgen receptor-targeted therapy has become more commonplace since patients were recruited to the TALAPRO-2 study^36–38^. Over one-third of the HRR-deficient population in TALAPRO-2 had received prior docetaxel or abiraterone for castration-sensitive disease^36^, and these patients had a significant 57% reduction in risk of radiographic progression or death. However, only 8% of patients in either arm had received prior abiraterone; the benefit in these patients is hypothesis generating and warrants further studies.

The safety profile of talazoparib plus enzalutamide was closely aligned with that observed in the previously reported all-comers population^25^. The incidence of anemia, including grade 3 and 4 events, was higher than with talazoparib monotherapy^22,39^. Anemia was managed through close patient monitoring, protocol-mandated dose interruption to permit recovery followed by dose reduction for grade ≥3 anemia (once hemoglobin levels were <8 g dl^−1^; to optimize individual treatment), and supportive measures, including packed red blood cell transfusions. To reflect the real-world patient population of mCRPC, often with bone metastases and bone marrow insufficiency, TALAPRO-2 could enroll patients with hemoglobin levels as low as 9 g dl^−1^. Notably, more than half of the patients (56%) had grade 1 and 2 anemia at baseline. Although 41% developed grade 3 and 4 anemia after a median talazoparib treatment duration of 3.3 months, only 4% of patients discontinued talazoparib because of anemia. Importantly, no cases of myelodysplastic syndrome or acute myeloid leukemia were reported. The incidence of permanent discontinuation was similar between the treatment groups, and the median relative dose intensity of talazoparib remained high at >80%.

In conclusion, these results support the use of talazoparib plus enzalutamide as a potential first-line treatment option for patients with mCRPC harboring tumor HRR gene alterations.

## Methods

### Trial design and patients

TALAPRO-2 (NCT03395197) is an ongoing double-blind, randomized, placebo-controlled trial. Details of the trial design have been published^23^ and are in the protocol (Supplementary Protocol).

Eligibility criteria included ongoing androgen deprivation therapy; asymptomatic or mildly symptomatic mCRPC with HRR gene alterations; Eastern Cooperative Oncology Group performance status score of 0 or 1; progressive disease; adequate bone marrow function (hemoglobin ≥9 g dl^−1^); and no prior life-prolonging systemic therapy for castration-resistant disease^23^. Prior docetaxel and abiraterone or orteronel in the castration-sensitive setting were allowed. Patients were randomized in a 1:1 ratio (using a centralized, interactive web response system and a permuted block size of 4) to receive 0.5 mg talazoparib (moderate renal impairment, 0.35 mg) or placebo (all received enzalutamide, 160 mg) once daily. Randomization was stratified by prior second-generation androgen receptor pathway inhibitor (abiraterone or orteronel) or docetaxel (yes/no). Formal crossover from the placebo group to the talazoparib group was not part of the study design.

Before randomization, patients consented to provide solid tumor tissue (de novo or archival) and/or blood-based samples, for prospective assessment of HRR gene alterations (*BRCA1*, *BRCA2*, *PALB2*, *ATM*, *ATR*, *CHEK2*, *FANCA*, *RAD51C*, *NBN*, *MLH1*, *MRE11A*, *CDK12*) using FoundationOne® CDx and/or FoundationOne Liquid® CDx (Foundation Medicine). Historical FoundationOne® test results could also be used. Patients were considered HRR-deficient if they had one or more alteration(s) in at least one of these 12 genes. For prospective HRR status determination, test records generated after the randomization date were excluded. Alterations were defined as truncating short variants, selected inactivating short variants identified as known/likely pathogenic per FoundationOne® pipeline, inactivating rearrangements or homozygous deletion of one or more exons. The definition of HRR alterations was the same for FoundationOne® Liquid CDx, except homozygous deletion of one or more exons was limited to *BRCA1/BRCA2* only. Notably, patients with heterozygous deletions of one or more exons alone were not enrolled in the HRR-deficient population.

Enrollment of patients with *ATM* and/or *CDK12* gene alterations was paused between January and November 2021 as their observed prevalence exceeded expectations^7^ and was anticipated to suppress the representation of alterations in the remaining genes under study. The pause in enrollment of patients with *ATM* and/or *CDK12* gene alterations was driven by expected prevalence numbers based on the largest and most comprehensive prospective assessment of prostate cancer tumor samples using the FoundationOne® assay^7^. This pause occurred in a blinded fashion regarding distribution of HRR alterations to the two treatment arms and allowed a rebalancing of the distribution across the 12-gene panel in an effort to best reflect the prevalence in mCRPC^7^.

Study treatment continued until radiographic progression, adverse event leading to permanent discontinuation, patient decision to discontinue or death. Treatment could continue after radiographic progression if the investigator determined benefit was still being derived.

The trial was conducted in accordance with the International Ethical Guidelines for Biomedical Research Involving Human Subjects, Good Clinical Practice guidelines, the principles of the Declaration of Helsinki and local laws. The protocol and amendments were approved by the institutional review board and independent ethics committee for each site. The following independent ethics committees or Institutional Review Boards provided study approval: Comite de Revision Institucional - Hospital Britanico de Buenos Aires, CABA, Argentina; Comite de Etica ‘Dr. Claude Bernard’, Rosario, Argentina; Comite de Etica en Investigacion - Centro de Educacion Medica e Investigaciones Clinicas ‘Norberto Quirno’ – CEMIC, CABA, Argentina; Comite de Etica en Investigacion de la Fundacion OncoSalud (CEIFOS), Pergamino, Argentina; Comite Independiente De Etica Para Ensayos En Farmacologia Clinica, CABA, Argentina; Comite Institucional de Etica de la Investigacion en Salud (C.I.E.I.S) de la Clinica Universitaria Reina Fabiola, Cordoba, Argentina; Comite Institucional de Etica de Investigacion en Salud del Hospital Privado Centro Medico de Cordoba, Cordoba, Argentina; St Vincent’s Hospital Human Research Ethics Committee, Darlinghurst, Australia; Bellberry Limited, Eastwood, Australia; Commissie Voor Medische Ethiek, Gent, Belgium; Comissao Nacional de Etica em Pesquisa/CONEP, Brasilia, Brazil; Comite de Etica em Pesquisa da Fundacao Pio XII - Hospital de Cancer de Barretos, Barretos, Brazil; Comite de Etica em Pesquisa da Universidade do Vale do Taquari – UNIVATES, Lajeado, Brazil; Comite de Etica em Pesquisa do Hospital Mae de Deus, Porto Alegre, Brazil; Comite de Etica em Pesquisa do Instituto D’Or de Pesquisa e Ensino, Rio de Janeiro, Brazil; Comite de Etica em Pesquisa-Hospital Universitario Pedro Ernesto, Rio de Janeiro, Brazil; Comite de Etica em Pesquisa da Universidade Regional do Noroeste do Estado do Rio Grande do Sul, Ijui, Brazil; Comite de Etica em Pesquisa - CEP do Hospital das Clinicas da Faculdade de Medicina da Universidade de Sao Paulo - HCFMUSP, Sao Paulo, Brazil; Comite de Etica em Pesquisa da Faculdade de Medicina do ABC, Santo Andre, Brazil; Comitê de Ética em Pesquisa do Instituto Nacional de Câncer Jose Alencar Gomes da Silva – INCA, Rio de Janeiro, Brazil; Comite de Etica em Pesquisa do Hospital Nossa Senhora da Conceicao - Grupo Hospitalar Conceicao, Porto Alegre, Brazil; Comite de Etica em Pesquisa da Sociedade Beneficente de Senhoras Hospital Sirio Libanes, Sao Paulo, Brazil; Comitê de Ética em Pesquisa do Hospital Alemão Oswaldo Cruz – SP, Sao Paulo, Brazil; Comite de Etica em Pesquisa da Pontificia Universidade Catolica do Rio Grande do Sul-PUC/RS, Porto Alegre, Brazil; Comite d’ethique de la recherche du CHUM, Montreal, Canada; Health Research Ethics Board of Alberta - Cancer Committee, Edmonton, Canada; Ontario Cancer Research Ethics Board, Toronto, Canada; Comite de Etica Cientifico Servicio de Salud Metropolitano Oriente, Santiago, Chile; Comite Etico Cientifico Hospital Gustavo Fricke Servicio de Salud Vina del Mar – Quillota, Vina del Mar, Chile; Comite de Etica Cientifica Servicio Salud Araucania Sur, Temuco, Chile; Ethics Committee of Zhongshan Hospital Fudan University, Shanghai, China; Ethics Committee of The First Affiliated Hospital of Xi’an Jiaotong University, Xi’an, China; Ethics committee of Zhejiang Cancer Hospital, Hangzhou, China; Ethics Committee of National Cancer Center/Cancer Hospital, Chinese Academy of Medical Sciences and Peking Union Medical College, Beijing, China; Ethics Committee of Chongqing University Cancer Hospital, Chongqing, China; Ethics Committee of Beijing Cancer Hospital, Beijing, China; Ethics Committee of The First Affiliated Hospital of Anhui Medical University, Hefei, China; Medical Ethics Committee of First Affiliated Hospital of Xiamen University, Xiamen, China; Wuxi People’s Hospital Ethics Committee, Wuxi, China; Ethics Committee of Nanjing Drum Tower Hospital, Nanjing, China; Ethics Committee of Shanghai Tenth People’s Hospital, Shanghai, China; Clinical Trial Ethics Committee of Huazhong University of Science and Technology, Wuhan, China; Ethics Committee of Huashan Hospital, Fudan University, Shanghai, China; Ethics Committee of Fudan University Cancer Hospital, Shanghai, China; Ethics Committee of Beijing Hospital, Beijing, China; Ethics Committee of Peking University First Hospital, Beijing, China; Peking University Third Hospital Medical Science Research Ethics Committee, Beijing, China; Ethics Committee for Clinical Trials of Drugs (Medical Apparatus) of Ningbo First Hospital, Ningbo, China; Ethics Committee of Ruijin Hospital Affiliated to Shanghai Jiaotong University School of Medicine, Shanghai, China; West China Hospital of Sichuan University Clinical Trial Ethics Committee, Chengdu, China; EC of Second Affiliated Hospital of Suzhou University, Suzhou, China; Shanghai General Hospital Medical Ethics Committee, Shanghai, China; Ethics Committee of Nanjing First Hospital, Nanjing, China; Clinical Trial Ethics Committee of Huazhong University of Science and Technology, Wuhan, China; Drug and Machinery Clinical trial Branch of EC of The First Affiliated Hospital of Fujian Medical University, Fuzhou, China; Ethics Committee of Yunnan Cancer Hospital, Kunming, China; The First Affiliated Hospital of Nanchang University Ethics Committee, Nanchang, China; Jilin Cancer Hospital Institutional Review Board, Changchun, China; Ethics Committee of The Second Hospital of Tianjin Medical University, Tianjin, China; Ethics Committee of Zhejiang Provincial People’s Hospital, Hangzhou, China; Ethics Committee of The Fifth People’s Hospital of Shanghai, Fudan University, Shanghai, China; Ethics Committee of Nantong Tumor Hospital, Nantong, China; Medical Ethics Committee of The First People’s Hospital of Lianyungang, Lianyungang, China; The Clinical Trial Ethics Committee of The First Affiliated Hospital of Wenzhou Medical University, Wenzhou, China; Eticka komise Krajska zdravotni a.s., Masarykova nemocnice v Usti nad Labem, Usti and Labem, Czech Republic; Eticka komise pro multicentricke klinicke hodnoceni Fakultni nemocnice Kralovske Vinohrady, Praha, Czech Republic; Eticka komise Fakultni Nemocnice Ostrava, Ostrava-Poruba, Czech Republic; Eticka komise Fakultni nemocnice Hradec Kralove, Hradec Kralove, Czech Republic; Helsingin ja Uudenmaan sairaanhoitopiiri, Helsinki, Finland; Comite De Protection Des Personnes (CPP) Sud-Ouest Et Outre-Mer III, Bordeaux, France; Ethikkommission der Aerztekammer Hamburg, Hamburg, Germany; Egészségügyi Tudományos Tanács Klinikai Farmakológiai Etikai Bizottsága, Budapest, Hungary; Bnai Zion Medical Center Helsinki Committee, Haifa, Israel; Rambam Health Care Campus Helsinki Committee, Haifa, Israel; Tel Aviv Sourasky Medical Center Helsinki Committee, Tel Aviv, Israel; Rabin Medical Center Helsinki Committee, Petah Tikva, Israel; Shaare Zedek Medical Center Helsinki Committee, Jerusalem, Israel; Comitato Etico Azienda Ospedaliero Universitaria San Luigi Gonzaga, Orbassano, Italy; Comitato Etico Val Padana, Cremona, Italy; Comitato Etico Regionale (CER) dell’Umbria, Perugia, Italy; Comitato Etico Cardarelli-Santobono, Napoli, Italy; Comitato Etico Per Le Sperimentazioni Cliniche Dell’Azienda Provinciale Per I Servizi Sanitari, Trento, Italy; Comitato Etico della Romagna (CEROM), Meldola, Italy; Comitato Etico di Brescia, Brescia, Italy; Comitato Etico di Area Vasta Emilia Centro, Bologna, Italy; Comitato Etico IRCCS Pascale, Napoli, Italy; National Hospital Organization Central Review Board, Meguro-ku, Tokyo, Japan; National Cancer Center Institutional Review Board, Chuo-ku, Tokyo, Japan; Kindai University Hospital Institutional Review Board, Osakasayama, Japan; Yokohama City University Medical Center Institutional Review Board, Yokohama, Japan; Keio University Hospital Institutional Review Board, Shinjuku-ku, Tokyo, Japan; Nagoya University Hospital Institutional Review Board, Nagoya, Japan; Hokkaido University Hospital Institutional Review Board, Sapporo, Japan; Tokushima University Hospital Institutional Review Board, Tokushima, Japan; Chiba Cancer Center Institutional Review Board, Chiba, Japan; Hirosaki University School of Medicine & Hospital Institutional Review Board, Hirosaki, Japan; Yamagata Prefectural Central Hospital Institutional Review Board, Yamagata, Japan; Yokosuka Kyosai Hospital Institutional Review Board, Yokosuka, Japan; Hamamatsu University School of Medicine, University hospital Institutional Review Board, Hamamatsu, Japan; Osaka International Cancer Institute Institutional Review Board, Osaka-shi, Japan; Osaka University Hospital Institutional Review Board, Suita, Japan; Kanazawa University Hospital Institutional Review Board, Kanazawa, Japan; Kagoshima University Hospital Institutional Review Board, Kagoshima, Japan; Yamagata University Hospital Institutional Review Board, Yamagata, Japan; Kyungpook National University Chilgok Hospital Institutional Review Board, Daegu, Republic of Korea; Samsung Medical Center Institutional Review Board, Seoul, Republic of Korea; Asan Medical Center Institutional Review Board, Seoul, Republic of Korea; Severance Hospital, Yonsei University Health System Institutional Review Board, Seoul, Republic of Korea; Pusan National University Hospital Institutional Review Board, Busan, Republic of Korea; Seoul National University Hospital Institutional Review Board, Seoul, Republic of Korea; National Cancer Center Institutional Review Board, Goyang-si, Republic of Korea; The Catholic University of Korea Seoul St. Mary’s Hospital Institutional Review Board, Seoul, Republic of Korea; Health and Disability Ethics Committee, Wellington, New Zealand; REK Sor-Ost, Oslo, Norway; Comite Institucional de Etica en Investigacion del INEN, Lima, Peru; Comite Institucional de Bioetica de Via Libre, Lima, Peru; Komisja Bioetyczna przy Okregowej Izbie Lekarskiej w Gdansku, Gdansk, Poland; Comissao de Etica para a Investigacao Clinica, Lisboa, Portugal; University of the Witwatersrand Human Research Ethics Committee (Medical), Johannesburg, South Africa; CEIm del Hospital Universitari Vall d’Hebron, Barcelona, Spain; Etikprovningsmyndigheten, Uppsala, Sweden; Health and Care Research Wales, Wales REC 3, Cardiff, United Kingdom; Advarra Institutional Review Board, Columbia, MD, United States; Vanderbilt Human Research Protection Program (VHRPP) Institutional Review Board, Nashville, TN, United States; University of Utah Institutional Review Board, Salt Lake City, UT, United States; Biomedical Research Alliance of New York, LLC/Institutional Review Board, Lake Success, NY, United States; Western Institutional Review Board, Puyallup, WA, United States; Sharp HealthCare Institutional Review Board, San Diego, CA, United States; Schulman Associates Institutional Review Board, Cincinnati, OH, United States; Loma Linda University Health - Institutional Review Board, Loma Linda, CA, United States; Administrative Panels on Human Subjects in Medical Research (‘Stanford Institutional Review Board’), Palo Alto, CA, United States; University of Maryland, Baltimore - Institutional Review Board, Baltimore, MD, United States; Cook County Health Office of Research and Regulatory Affairs, Chicago, IL, United States; Samaritan Health Services Regional Institutional Review Board, Corvallis, OR, United States; University of Iowa Institutional Review Board-01, Human Subjects Office, Iowa City, IA, United States; Lakeland Regional Medical Center, Inc. Institutional Review Board, Lakeland, FL, United States; VA Med Ctr, Long Beach CA Institutional Review Board #1, Long Beach, CA, United States; Rush University Medical Center Institutional Review Board, Chicago, IL, United States; UCLA Office of the Human Research Protection Program, Los Angeles, CA, United States; VA Saint Louis Healthcare System Institutional Review Board, St. Louis, MO, United States; Baylor Scott and White Research Institute Institutional Review Board-Gold, Temple, TX, United States; Providence St. Joseph Health Institutional Review Board, Renton, WA, United States; IntegReview, Austin, TX, United States; Kaiser Permanente Northwest Institutional Review Board, Portland, OR, United States; Ochsner Institutional Review Board, New Orleans, LA, United States; Eisenhower Medical Center, Institutional Review Board, Rancho Mirage, CA, United States. All patients provided written informed consent.

### Trial endpoints

The primary endpoint was rPFS by blinded independent central review per Response Evaluation Criteria in Solid Tumors (version 1.1; soft tissue disease) and Prostate Cancer Clinical Trials Working Group 3 (bone disease)^23^. A full list of secondary endpoints is included in the Supplementary Methods, and these endpoints have been previously listed^23^. Planned secondary endpoints not reported in this article are: time to opiate use for prostate cancer pain, pharmacokinetics and patient-reported outcomes.

Exploratory subgroup analyses were conducted for rPFS by baseline characteristics (Supplementary Methods). A post hoc analysis of rPFS by *BRCA1/BRCA2* alteration status (yes/no) and by single genes and hierarchical gene clusters (*BRCA*, *PALB2*, *CDK12*, *ATM* and any of all other HRR genes) was also performed (Supplementary Methods).

### Statistical analysis

Approximately 380 patients with HRR gene alterations were to be enrolled. To maintain overall type I error at or below a one-sided alpha level of 0.025, alpha was split equally between the all-comers cohort and HRR-deficient population.

For the primary comparison in the HRR-deficient population, 224 PFS events based on a Lan DeMets α-spending function would provide 85% power to detect an HR of 0.64 using a one-sided stratified log-rank test at a significance level of 0.0125. A prespecified interim analysis of PFS was planned after approximately 70% of the expected events (157 events). The HRR-deficient cohort would be stopped if the efficacy boundary was crossed and an interim efficacy analysis of overall survival would be performed. As the efficacy boundary (*P* ≤ 0.0038) was crossed at this interim analysis, this became the final analysis. Other endpoints had no adjustment for multiplicity. Survival and safety follow-up continue.

Time-to-event endpoints were compared between treatment groups using a stratified log-rank test unless otherwise stated. HRs and associated 95% two-sided CIs were estimated by a Cox proportional hazards model. Median time-to-event endpoints were estimated by the Kaplan–Meier method, and 95% CIs based on the Brookmeyer–Crowley method. For subgroup analysis of rPFS (except by *BRCA* status), the HR was based on an unstratified Cox model with treatment as the only covariate due to small patient numbers in some subgroups. Missing/partial dates were imputed as specified per protocol. Other missing data were not imputed. Reported *P* values are two sided.

Oracle Clinical Remote Data Capture was used for data collection, and SAS version 9.4 was used for data analysis.

### Reporting summary

Further information on research design is available in the Nature Portfolio Reporting Summary linked to this article.

## Online content

Any methods, additional references, Nature Portfolio reporting summaries, source data, extended data, supplementary information, acknowledgements, peer review information; details of author contributions and competing interests; and statements of data and code availability are available at 10.1038/s41591-023-02704-x.

### Supplementary information

Supplementary Information List of investigators, supplementary methods and redacted protocol.

Reporting Summary

## Figures and Tables

**Extended Data Table 1:**
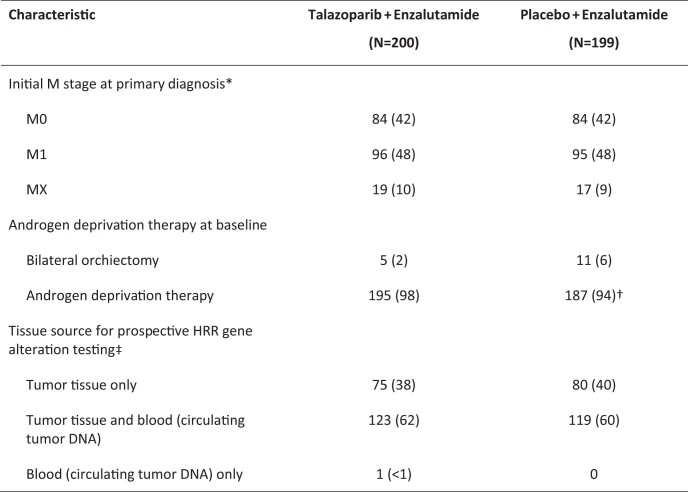
Additional Baseline Disease Characteristics (HRR-Deficient Intention-to-Treat Population)

Data are no. (%).

*Not reported for the remaining patients.

†Prior androgen deprivation therapy was not available in the clinical database for one patient as of the data cutoff, although this patient received androgen deprivation therapy prior to enrollment and continued to receive this treatment.

‡The tissue source was not specified for one patient in the talazoparib arm.

HRR denotes homologous recombination repair.

**Extended Data Table 2:**
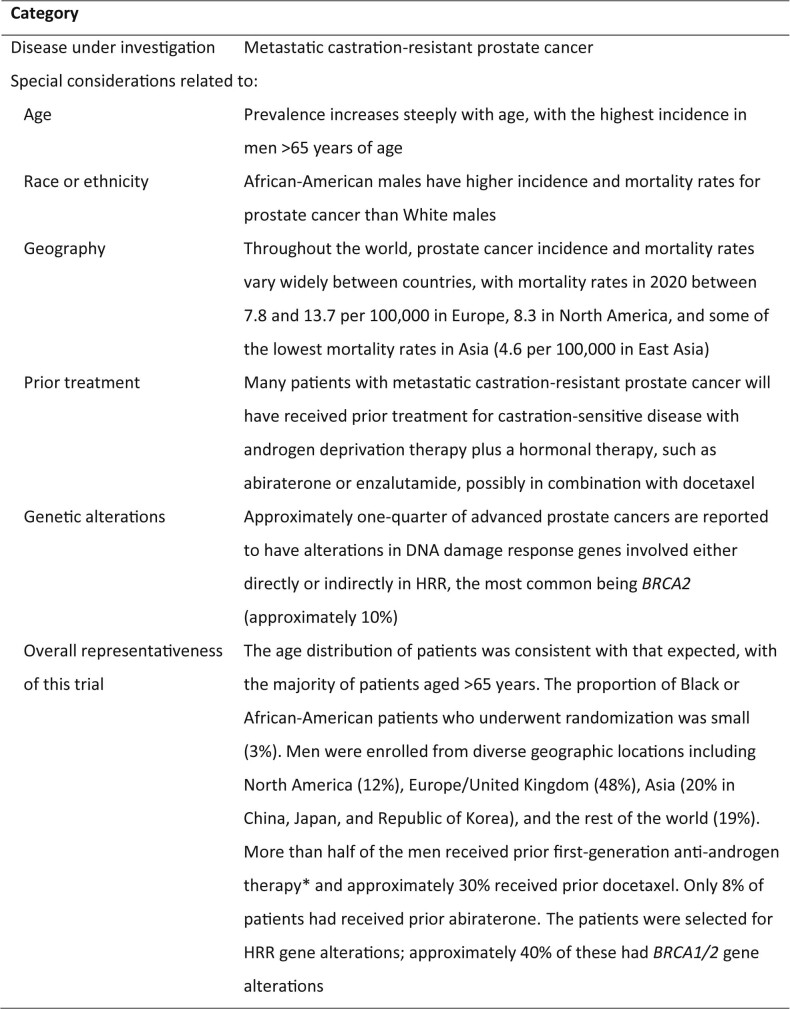
Representativeness of study participants

*Bicalutamide, flutamide, nilutamide, cyproterone acetate.

HRR denotes homologous recombination repair.

**Extended Data Table 3:**
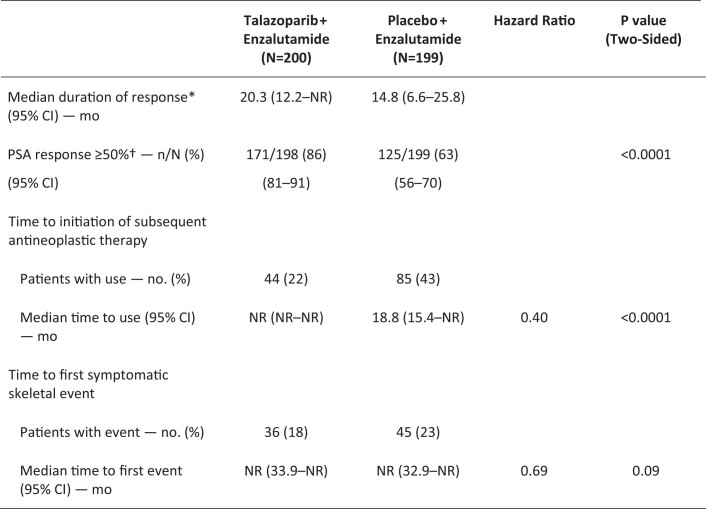
Additional secondary efficacy outcomes (HRR-deficient intention-to-treat population)

Time-to-event endpoints were compared between treatment groups using stratified log-rank test. HRs and associated 95% two-sided CIs were estimated by a Cox proportional hazards model. Median time-to-event endpoints were estimated by the Kaplan–Meier method, and 95% CIs based on the Brookmeyer–Crowley method. P values are two-sided.

*Only includes patients with confirmed complete response or partial response: talazoparib plus enzalutamide (*N* = 49); placebo plus enzalutamide (*N* = 26).

†The number of patients with a baseline PSA value and at least one post-baseline PSA value: talazoparib plus enzalutamide (*N* = 198); placebo plus enzalutamide (*N* = 199).

CI denotes confidence interval, HRR homologous recombination repair, NR not reached at the time of the analysis, and PSA prostate-specific antigen.

## Data Availability

Upon request, and subject to review, Pfizer will provide the data that support the findings of this study. Subject to certain criteria, conditions and exceptions, Pfizer may also provide access to the related individual de-identified participant data. See https://www.pfizer.com/science/clinical-trials/trial-data-and-results/ for more information.
