# Publisher Correction: First-line talazoparib with enzalutamide in HRR-deficient metastatic castration-resistant prostate cancer: the phase 3 TALAPRO-2 trial

**DOI:** 10.1038/s41591-024-02835-9

**Published:** 2024-01-31

**Authors:** Karim Fizazi, Arun A. Azad, Nobuaki Matsubara, Joan Carles, Andre P. Fay, Ugo De Giorgi, Jae Young Joung, Peter C. C. Fong, Eric Voog, Robert J. Jones, Neal D. Shore, Curtis Dunshee, Stefanie Zschäbitz, Jan Oldenburg, Dingwei Ye, Xun Lin, Cynthia G. Healy, Nicola Di Santo, A. Douglas Laird, Fabian Zohren, Neeraj Agarwal

**Affiliations:** 1 Institut Gustave Roussy, University of Paris-Saclay, Villejuif, France; 2 Peter MacCallum Cancer Centre, Melbourne, Victoria Australia; 3 National Cancer Center Hospital East, Chiba, Japan; 4 Vall d’Hebron University Hospital, Vall d’Hebron Institute of Oncology (VHIO), Barcelona, Spain; 5 PUCRS School of Medicine, Porto Alegre, Brazil; 6 IRCCS Istituto Romagnolo per lo Studio dei Tumori (IRST) Dino Amadori, Meldola, Italy; 7 National Cancer Center, Goyang, Republic of Korea; 8 Auckland City Hospital, Auckland, New Zealand; 9 University of Auckland, Auckland, New Zealand; 10 Clinique Victor Hugo Centre Jean Bernard, Le Mans, France; 11 School of Cancer Sciences, University of Glasgow, Beatson West of Scotland Cancer Centre, Glasgow, UK; 12 Carolina Urologic Research Center, Myrtle Beach, SC USA; 13 Arizona Urology Specialists, Tucson, AZ USA; 14 National Center for Tumor Diseases (NCT), Heidelberg University Hospital, Heidelberg, Germany; 15 Akershus University Hospital (Ahus), Lørenskog, Norway; 16 Fudan University Shanghai Cancer Center, Shanghai, China; 17 Pfizer Inc., La Jolla, CA USA; 18 Pfizer Inc., Collegeville, PA USA; 19 Pfizer Inc., Durham, NC USA; 20 Pfizer Inc., New York, NY USA; 21 Huntsman Cancer Institute (NCI-CCC), University of Utah, Salt Lake City, UT USA

**Keywords:** Prostate cancer, Prostate cancer

Correction to: *Nature Medicine* 10.1038/s41591-023-02704-x, published online 4 December 2023.

In the version of the article initially published, the top right label in Fig. 4e, now reading “Median PSF2”, was a duplicate of the top right label in Fig. 4d. The error has been corrected in the HTML and PDF versions of the article.

