# Supplementary material for: First-line talazoparib with enzalutamide in HRR-deficient metastatic castration-resistant prostate cancer: the phase 3 TALAPRO-2 trial
Source: Nat Med. 2023 Dec 4;30(1):257–64. doi: 10.1038/s41591-023-02704-x (PMC10803259; doi:10.1038/s41591-023-02704-x)
Supplement: Supplementary file 1 — List of investigators, supplementary methods and redacted protocol. [file 41591_2023_2704_MOESM1_ESM.pdf]

nature medicine

Article

https://doi.org/10.1038/s41591-023-02704-x

First-line talazoparib with enzalutamide in HRR-deficient metastatic castration-resistant prostate cancer: the phase 3 TALAPRO-2 trial

In the format provided by the authors and unedited

List of Investigators

**Country**	**Principal Investigator**
Argentina	Santiago Bella, Clinica Universitaria Reina Fabiola
	Luis Fein, Instituto de Oncologia de Rosario
	Martin Greco, Centro de Educacion Medica e Investigaciones Clinicas "Norberto Quirno" Cemic
	Susana Kahl, Centro de Investigacion Pergamino SA - Clinica Pergamino SA
	Ernesto Korbenfeld, Hospital Británico de Buenos Aires
	Esteban Metrebian, Hospital Privado Centro Medico de Cordoba
	Luis Montes de Oca, Centro de Urologia-CDU
Australia	Arun Azad, Peter MacCallum Cancer Centre
	Stephen Begbie, Port Macquarie Base Hospital
	Lisa Horvath, Chris O'Brien Lifehouse
	Anthony Joshua, St Vincent's Hospital Sydney
	Elizabeth McCaffrey, Princess Alexandra Hospital
	Paul Vasey, Integrated Clinical Oncology Network Cancer Centre
Belgium	Wim Demey, AZ Klina
	Lionel D'Hondt, CHU UCL Namur site Godinne
	Karl Lesage, AZ Groeninge
	Nicolaas Lumen, UZ Gent
	Vincent Renard, A.Z. Sint-Lucas
	Siska Van Bruwaene, AZ Groeninge
	Nicolas Whenham, Clinique Saint-Pierre Ottignies Oncologie
Brazil	Alan Azambuja, Centro Gaucho Integrado - Hospital Mae de Deus
	Diogo Bastos, Sociedade Beneficente de Senhoras Hospital Sírio Libanês
	Leandro Brust, Sociedade Beneficencia e Caridade de Lajeado - Hospital Bruno Born
	Flavio Carcano, Fundacao Pio XII - Hospital de Cancer de Barretos
	Ronaldo Damiao, Hospital Universitario Pedro Ernesto-Centro de Pesquisas (CEPUSA)
	Andre Fay, Centro de Pesquisa Clinica em Oncologia - Hospital Sao Lucas da Pontificia Universidade Catolica do Rio Grande do Sul - PUC/RS
	Fabio Franke, Associacao Hospital de Caridade de Ijui
	Carlos Gorini, Hospital Nossa Senhora da Conceicao - Grupo Hospitalar Conceicao
	Daniel Herchenhorn, Hospital CopaDor
	Ariel Kann, Hospital Alemão Oswaldo Cruz Centro Internacional de Pesquisa – HAOC
	Suelen Patricia Martins, Faculdade de Medicina do ABC - Centro de Estudos e Pesquisa de Hematologia e Oncologia (CEPHO)
	David Muniz, Instituto do Cancer do Estado de Sao Paulo - ICESP
	Monica Padoan, Instituto Nacional de Câncer José de Alencar Gomes

	da Silva - INCA
	Daniel Preto, Fundacao Pio XII - Hospital de Cancer de Barretos
Canada	Christina Canil, The Ottawa Hospital Cancer Center
	Michel Carmel, Centre integre universitaire de sante et de services sociaux de l'Estrie
	Cristiano Ferrario, Jewish General Hospital
	Jean-Benoit Paradis, Centre integre universitaire de sante et de services sociaux du Saguenay-Lac-Saint-Jean
	Fred Saad, CHUM - Centre Hospitalier de l'Universite de Montreal
	Steven Yip, Tom Baker Cancer Centre - Alberta Health Services
Chile	Pablo Gonzalez Mella, Centro de Investigaciones Clinicas Vina del Mar
	Eugenia Loredo Fort, Centro de Investigaciones Clinicas Vina del Mar
	Francisco Orlandi Jorquera, Sociedad Prosalud Montes y Orlandi Ltda
	Pedro Pastor Arroyo, Centro de Investigacion Clinica del Sur
	Eduardo Yanez Ruiz, Sociedad de Investigaciones Medicas Ltda (SIM)
China	Wei Chen, The First Affiliated Hospital of Wenzhou Medical University
	Ying Cheng, Jilin Cancer Hospital
	Hongqian Guo, Nanjing Drum Tower Hospital
	Jianming Guo, Zhongshan Hospital Fudan University
	Dalin He, The First Affiliated Hospital of Xi'an Jiaotong University
	Zhisong He, Peking University First Hospital
	Qiang Hu, Wuxi People's Hospital
	Ruipeng Jia, Nanjing First Hospital
	Haowen Jiang, Huashan Hospital Fudan University
	Junhui Jiang, Ningbo First Hospital
	Xiaodong Jiang, The First People's Hospital of Lianyungang
	Chaozhao Liang, The First affiliated Hospital of Anhui Medical University
	Hong Luo, Chongqing University Cancer Hospital
	Lulin Ma, Peking University Third Hospital
	Guowei Shi, The Fifth People's Hospital of Shanghai
	Ting Sun, The First Affiliated Hospital of Nanchang University
	Ben Wan, Beijing Hospital
	Qilin Wang, Yunnan Cancer Hospital
	Shaogang Wang, Tongji Hospital, Tongji Medical College
	Xiaolin Wang, Nantong Tumor Hospital
	Yong Wang, The Second Hospital of Tianjin Medical University
	Shujie Xia, Shanghai General Hospital
	Jinchun Xing, First Affiliated Hospital of Xiamen University
	Nianzeng Xing, Cancer Institute and Hospital, Chinese Academy of Medical Sciences
	Danfeng Xu, Ruijin Hospital Affiliated to Shanghai Jiaotong University School of Medicine
	Boxin Xue, Second Affiliated Hospital of Suzhou University
	Xueyi Xue, The First Affiliated Hospital of Fujian Medical University

	Yong Yang, Beijing Cancer Hospital
	Xudong Yao, Shanghai Tenth People's Hospital
	Dingwei Ye, Fudan University Cancer Hospital
	Hao Zeng, West China Hospital of Sichuan University
	Qi Zhang, Zhejiang Provincial People's Hospital
	Xiaoping Zhang, Union Hospital, Tongji Medical College
	Shaoxing Zhu, Zhejiang Cancer Hospital
Czech Republic	Jan Dvorak, Fakultní nemocnice Královské Vinohrady
	Jaroslav Hajek, Fakultni nemocnice Ostrava
	Karel Odrazka, Multiscan s.r.o.
	Jiri Petera, Fakultní nemocnice Hradec Králové
	Jan Schraml, Masarykova nemocnice v Usti nad Labem, o.z, Krajska zdravotni a.s.
	Jaroslav Vanasek, Multiscan s.r.o.
Finland	Tuomo Alanko, Docrates Cancer Center
	Paivi Auvinen, Kuopio University Hospital
	Peter Bostrom, Turku University Hospital
	Okko Kaariainen, Kuopio University Hospital
	Antti Rannikko, HUS Helsinki University Hospital
	Hanna Ronkainen, OYS apteekki
	Teuvo Tammela, Tampere University Hospital
France	Philippe Barthelemy, Hopitaux Universitaires de Strasbourg - ICANS
	Philippe Beuzeboc, Hopital Foch
	Stephane Culine, Hopital Saint-Louis
	Louis Marie Dourthe, Clinique Sainte Anne
	Karim Fizazi, Institut Gustave Roussy
	Aude Flechon, Centre Leon Berard
	Marine Gross-Goupil, Hopital Saint Andre - CHU de Bordeaux
	Stephane Oudard, Hopital Europeen Georges Pompidou
	Frank Priou, CHD Vendée
	Maciej Rotarski, Centre d'Oncologie du Pays-Basque
	Delphine Topart, CHU Montpellier-Hopital Saint Eloi
	Eric Voog, Clinique Victor Hugo
Germany	Martin Boegemann, Universitaetsklinikum Muenster
	Susan Feyerabend, Diagnostikzentrum
	Peter Goebell, Universitaetsklinikum Erlangen
	Hans Heinzer, Universitaetsklinikum Hamburg-Eppendorf
	Andreas Huebner, Wissenschaftskontor Nord GmbH & Co. KG
	Theodor Klotz, Kliniken Nordoberpfalz AG
	Guenter Niegisch, Universitaetsklinikum Duesseldorf
	Gunhild Von Amsberg, Universitaetsklinikum Hamburg-Eppendorf
	Stefanie Zschäbitz, Universitaetsklinik Heidelberg
Hungary	Peter Arkosy, Debreceni Egyetem Klinikai Kozpont
	László Csaba Mangel, Pecsi Tudomanyegyetem Klinikai Kozpont
	Lajos Geczi, Országos Onkológiai Intézet
	Laszlo Landherr, Uzsoki Utcai Korhaz

	Peter Nyirady, Semmelweis Egyetem Urologiai Klinika
Israel	Suliman Boulos, Shaare Zedek Medical Center
	Sarel Halachmi, Bnai Zion Medical Center
	Gleb Kornev, Shaare Zedek Medical Center
	David Margel, Rabin Medical Center, Beilinson Hospital
	Ofer Nativ, Bnai Zion Medical Center
	Avivit Peer, Rambam Health Care Campus
	David Sarid, Tel Aviv Sourasky Medical Center
Italy	Consuelo Buttigliero, AOU San Luigi Gonzaga
	Orazio Caffo, Ospedale Santa Chiara
	Claudia Caserta, Azienda Ospedaliera S. Maria
	Ugo De Giorgi, Istituto Scientifico Romagnolo per lo Studio e la Cura dei Tumori (IRST) IRCCS
	Gaetano Facchini, Istituto Nazionale Tumori IRCCS
	Francesco Massari, Azienda Ospedaliero-Universitaria
	Rodolfo Passalacqua, ASST di Cremona
	Bruno Perrucci, ASST di Cremona
	Sandro Pignata, Istituto Nazionale Tumori IRCCS
	Ferdinando Riccardi, A.O.R.N. "A. Cardarelli"
	Francesca Valcamonico, ASST-Spedali Civili di Brescia
Japan	Hideki Enokida, Kagoshima University Hospital
	Kazutoshi Fujita, Osaka University Hospital
	Satoshi Fukasawa, Chiba Cancer Center
	Tomoya Fukawa, Tokushima University Hospital
	Toru Harabayashi, National Hospital Organization Hokkaido Cancer Center
	Yasuhiro Hashimoto, Hirosaki University School of Medicine & Hospital
	Katsuyoshi Hashine, National Hospital Organization Shikoku Cancer Center
	Koji Hatano, Osaka University Hospital
	Masashi Kato, Nagoya University Hospital
	Hiroaki Kikukawa, National Hospital Organization Kumamoto Medical Center
	Kazuki Kobayashi, Yokosuka Kyosai Hospital
	Atsushi Komaru, Chiba Cancer Center
	Takeo Kosaka, Keio University Hospital
	Nobuaki Matsubara, National Cancer Center Hospital East
	Ryuji Matsumoto, Hokkaido University Hospital
	Hideaki Miyake, Hamamatsu University School of Medicine
	Atsushi Mizokami, Kanazawa University Hospital
	Tetsuo Momma, National Hospital Organization Tokyo Medical Center
	Motonobu Nakamura, National Hospital Organization Kyushu Cancer Center
	Kazuo Nishimura, Osaka International Cancer Institute
	Kenji Numahata, Yamagata Prefectural Central Hospital

	Shiro Saito, National Hospital Organization Tokyo Medical Center
	Masanobu Shigeta, National Hospital Organization Kure Medical Center and Chugoku Cancer Center
	Shuichi Tatarano, Kagoshima University Hospital
	Ryotaro Tomida, National Hospital Organization Shikoku Cancer Center
	Norihiko Tsuchiya, Yamagata University Hospital
	Hirotsugu Uemura, Kindai University Hospital
	Hiroji Uemura, Yokohama City University Medical Center
	Motohide Uemura, Osaka University Hospital
Republic of Korea	Young Deuk Choi, Severance Hospital, Yonsei University
	Tae Gyun Kwon, Kyungpook National University Chilgok Hospital
	Se Hoon Park, Clinical Trials Center Pharmacy, Samsung Medical Center
	Kang Hyun Lee, Clinical Trial Pharmacy, National Cancer Center
	Hong Koo Ha, Pusan National University Hospital
	Cheol Kwak, Seoul National University Hospital
	Bumjin Lim, Asan Medical Center
	Choung Soo Kim, Asan Medical Center
	Ji Youl Lee, Clinical Trial Pharmacy, The Catholic University of Korea
	Jae Young Joung, Clinical Trial Pharmacy, National Cancer Center
New Zealand	James Edwards, Canterbury District Health Board
	Peter Fong, Auckland City Hospital
	Peter Gilling, Tauranga Urology Research Limited
	Alvin Tan, Waikato Hospital
Norway	Kristine Andreassen, Oslo University Hospital - Ullevål & Radiumhospitalet
	Reino Heikkila, Oslo University Hospital - Ullevål & Radiumhospitalet
	Daniel Heinrich, Akershus University Hospital
	Christian Kersten, Akershus University Hospital
	Jan Oldenburg, Akershus University Hospital
	Anne Marie Storas, Oslo University Hospital - Ullevål & Radiumhospitalet
	Torgrim Tandstad, St. Olavs Hospital, Trondheim University Hospital
	Oyvind Tennoe, Ostfold County Hospital, Kalnes
Peru	Renzo Alvarez Barreda, Clinica Monte Carmelo S.C.R.LTDA.
	Carlos Farfan Tello, Clinica Internacional Sede San Borja
	Zaida Morante Cruz, Clinica Oncosalud
	Silvia Neciosup Delgado, Instituto Nacional de Enfermedades Neoplasicas
	Alberto Pazos Franco, Hospital Militar Central "Coronel Luis Arias Schreiber"
	Luis Riva Gonzalez, Clinica Internacional Sede San Borja
Poland	Dorota Filarska, Szpitale Pomorskie Sp. z o.o.
	Albert Gugala, NZOZ Szpital Mazovia; City Clinic Sp. z o.o.
	Boguslawa Karaszewska, Przychodnia Lekarska "Komed" Roman Karaszewski

	Dariusz Sawka, Szpital Specjalistyczny w Brzozowie
	Cezary Szczylik, Europejskie Centrum Zdrowia Otwock
Portugal	Rui Dinis, Hospital do Espirito Santo de Evora
	Joana Febra, Centro Hospitalar do Porto
	Estevao Lima, Centro Clínico Académico - Braga
	Monica Mariano, Instituto Português Oncologia de Coimbra Francisco Gentil - E.P.E
	Paulo Mota, Centro Clínico Académico - Braga
	Alina Rosinha, Instituto Português de Oncologia do Porto Francisco Gentil
	Teresa Timóteo, Hospital da Luz Lisboa
	Nuno Vau, Fundação Champalimaud
South Africa	Michiel Botha, Outeniqua Cancercare Oncology Unit
	Khabane Chabane, Cancercare Langenhoven Drive Oncology Centre
	Jill Harris, Cancercare Rondebosch Oncology
	Conrad Jacobs, Cape Town Oncology Trials
	Peter Kraus, Outeniqua Cancercare Oncology Unit
	Jorn Malan, Cancercare Langenhoven Drive Oncology Centre
	Bernardo Rapoport, The Medical Oncology Centre of Rosebank
	Paul Ruff, Wits Clinical Research
Spain	Urbano Anido Herranz, Hospital Clinico Universitario de Santiago de Compostela
	Jose Arranz Arija, Hospital General Universitario Gregorio Maranon
	Daniel Castellano Gauna, Hospital Universitario 12 De Octubre
	Joan Carles Galceran, Hospital Universitari Vall d'Hebron
	Enrique Gallardo Diaz, Corporacio Sanitaria i Universitaria Parc Tauli
	Enrique Grande Pulido, MD Anderson Cancer Center
	Begoña Mellado Gonzalez, Hospital Clinic de Barcelona
	Josep Maria Piulats Rodriguez, Institut Catala d'Oncologia - ICO
	Nuria Romero Laorden, Hospital Universitario La Princesa
	Silverio Ros Martínez, Hospital Universitario Virgen de la Arrixaca
	Federico Jose Vazquez Mazon, Hospital General Universitario de Elche
Sweden	Lennart Astrom, Akademiska University Hospital
	Enrique Castellanos, Patientomrade Backencancer, Tema Cancer
	Claes Molin, Klinisk Provningsenhet (KPE) Onkologi
	Camilla Thellenberg Karlsson, Cancercentrum Norrlands Universitetssjukhus
United Kingdom	Robert Jones, NHS Greater Glasgow and Clyde
	John McGrane, Royal Cornwall Hospitals NHS Trust
	Peter Sankey, University Hospitals Plymouth NHS Trust
	Naveed Sarwar, Imperial College Healthcare NHS Trust
	Isabel Syndikus, The Clatterbridge Cancer Centre NHS Foundation Trust
	Mark Tuthill, Oxford University Hospitals NHS Trust
United States of America	Neeraj Agarwal, Farmington Health Center - University of Utah
	Carlos Alemany, AdventHealth Hematology and Oncology

	Leonard Appleman, UPMC Hillman Cancer Center
	Jeanny Aragon-Ching, Inova Schar Cancer Institute
	Edward Arrowsmith, Sarah Cannon Research Institute
	Vasileios Assikis, Piedmont Cancer Institute
	Adanma Ayanambakkam, Stephenson Cancer Center
	James Bailen, Clark Memorial Hospital Radiology
	Mohamed Bidair, San Diego Clinical Trials
	Mehmet Bilen, Emory University Hospital
	Gordon Brown, New Jersey Urology, LLC
	Mark Carmichael, Desert Hematology Oncology Medical Group, Incorporation
	Chien-Shing Chen, Loma Linda University Cancer Center
	William Clark, Alaska Urological Institute dba Alaska
	Patrick Cobb, St. Vincent Frontier Cancer Center
	King Coffield, Baylor Scott & White Medical Center - Temple
	Brendan Curti, Providence Cancer Institute Clackamas Clinic
	Jay Dalal, AMITA Health Adventist Medical Center
	Tracy Dobbs, Center for Biomedical Research
	Curtis Dunshee, Arizona Urology Specialists
	Richard Frank, Danbury Hospital
	Afshin Gabayan, Beverly Hills Cancer Center
	Sunil Gandhi, Florida Cancer Specialists
	Benjamin Gartrell, Montefiore Medical Center
	Lawrence Gervasi, Clinical Research Solutions
	Evan Goldfischer, Premier Medical Group of the Hudson Valley PC
	Keith Goldstein, Good Samaritan Hospital - Corvallis
	Pankaj Gupta, VA Long Beach Healthcare System
	Varun Gupta, Eisenhower Medical Center
	Jason Hafron, Michigan Institute of Urology
	Richard Harris, UroPartners LLC
	Ralph Hauke, Oncology Hematology West, PC dba
	Ralph Henderson, Regional Urology Oncology & Radiation Center
	Courtney Hollowell, Cook County Health (CCH)
	Arif Hussain, University of Maryland Greenebaum
	Sardar Imam, San Juan Oncology Associates
	Shikha Jain, Rush University Medical Center
	Fadi Joudi, Advanced Cancer Therapies
	Sujith Kalmadi, Ironwood Physicians P.C. dba Ironwood Cancer & Research Centers
	Lawrence Karsh, The Urology Center of Colorado
	Elizabeth Kessler, University of Colorado Denver
	Eric Knoche, VA Saint Louis Healthcare System
	Timothy Kuzel, Rush University Medical Center
	Daniel Lee, UPMC Hillman Cancer Center
	Glenn Liu, University of Wisconsin Clinical Science Center
	Constantine Mantz, 21st Century Oncology

	Sandeep Mashru, Kaiser Permanente Northwest
	Marc Matrana, Ochsner Clinic Foundation
	Brian Mazzarella, Urology Austin PLLC
	Bryan Mehlhaff, Oregon Urology Institute
	Alex Metzger, Marin Cancer Care, Inc.
	David Morris, Urology Associates P.C.
	Kelvin Moses, The Vanderbilt Clinic
	Sushma Nakka, Lakeland Regional Health Hollis Cancer Center
	Norman Nemoy, Tower Urology
	Petros Nikolinakos, University Cancer & Blood Center, LLC
	Luke Nordquist, GU Research Network/Urology Cancer Center
	James Orsini, Clara Maass Medical Center
	Rahul Parikh, The University of Kansas Cancer Center
	Belur Patel, Baylor Scott & White Medical Center - Temple
	Christopher Pieczonka, Associated Medical Professionals of New York, PLLC
	Evan Pisick, Midwestern Regional Medical Center
	Marc Pliskin, TriState Urologic Services PSC Inc., dba
	Santosh Rao, Banner Gateway Medical Center
	Charles Redfern, Cancer Center Oncology Medical Group
	James Reeves, Florida Cancer Specialists
	Matthew Rettig, UCLA Clark Urology Center
	Zvi Schiffman, Houston Metro Urology
	Mihran Shirinian, Glendale Adventist Medical Center
	Neal Shore, Carolina Urologic Research Center
	Paul Sieber, Keystone Urology Specialists
	Frederick Snoy, Urology Group of New Mexico
	Dan Spitz, Florida Cancer Specialists
	Sandhya Srinivas, Stanford Cancer Institute
	Donald Sweet, AMITA Health Adventist Medical Center
	Mahdi Taha, Southeastern Regional Medical Center
	David Topolsky, Eastern Regional Medical Center, Inc.
	Abhishek Tripathi, Stephenson Cancer Center
	Edward Uchio, University of California, Irvine Medical Center
	Madhavi Venigalla, Lakeland Regional Health Hollis Cancer Center
	Daniel Voglewede, Rio Grande Urology, P.A.
	Kristofer Wagner, Baylor Scott & White Medical Center - Temple
	John Wong, Loma Linda University Cancer Center
	Yousef Zakharia, University of Iowa Hospital and Clinics
	Song Zhao, Swedish Cancer Institute

Supplementary Methods

Secondary endpoints were overall survival, rPFS (investigator-assessed), objective response rate (in patients with measurable soft tissue disease at baseline per RECIST 1.1) and duration of soft tissue response (both by blinded independent central review), proportion of patients with PSA response ≥50%, time to PSA progression, time to initiation of cytotoxic chemotherapy, time to initiation of subsequent antineoplastic therapy, time to first symptomatic skeletal event, time to disease progression or death on the first subsequent antineoplastic therapy for prostate cancer (investigator-assessed), time to opiate use for prostate cancer pain (to be reported separately), safety (incidence of adverse events characterized by type, severity [graded by National Cancer Institute Common Terminology Criteria for Adverse Events version 4.03],[1] timing, seriousness, and relationship to study treatment), pharmacokinetics (to be reported separately), and patient-reported outcomes (to be reported separately).

Exploratory subgroup analyses for rPFS by blinded independent central review were performed by baseline characteristics (age [<70/≥70 years], ECOG performance status [0/1], Gleason score [<8/≥8], stage at diagnosis [M0/M1], site of metastasis [bone only/soft tissue only/bone and soft tissue], and prior second-generation androgen receptor pathway inhibitor (abiraterone or orteronel) or docetaxel for castration-sensitive prostate cancer [yes vs. no]).

For post hoc analysis of rPFS by single genes and gene clusters, only patients bearing alteration(s) in the designated HRR gene and none of the other HRR genes investigated were included in the single-gene subgroups and alteration dominance hierarchy for the clusters was any *BRCA1/2* alteration (*BRCA* cluster), then any *PALB2* (*PALB2* cluster), next any *CDK12* (*CDK12* cluster), then any *ATM* (*ATM* cluster), and finally any of all other genes (with each patient counted only once).

Reference

1. U.S. Department of Health and Human Services. Common terminology criteria for adverse events (CTCAE) v4.03 June 14, 2010. National Cancer Institute. (Accessed October 31, 2023 at

https://evs.nci.nih.gov/ftp1/CTCAE/CTCAE_4.03/CTCAE_4.03_2010-06-14_QuickReference_8.5x11.pdf.)

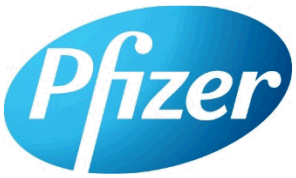

TALAPRO-2

A PHASE 3, RANDOMIZED, DOUBLE-BLIND, PLACEBO-CONTROLLED STUDY OF TALAZOPARIB WITH ENZALUTAMIDE IN METASTATIC CASTRATION-RESISTANT PROSTATE CANCER

**Investigational Product Number:**	PF-06944076
**Investigational Product Name:**	Talazoparib
**United States (US) Investigational New Drug (IND) Number:**	129,642
**European Clinical Trials Database (EudraCT) Number:**	2017-003295-31
**Protocol Number:**	C3441021
**Phase:**	3

This document and accompanying materials contain confidential information belonging to Pfizer. Except as otherwise agreed to in writing, by accepting or reviewing these documents, you agree to hold this information in confidence and not copy or disclose it to others (except where required by applicable law) or use it for unauthorized purposes. In the event of any actual or suspected breach of this obligation, Pfizer must be promptly notified.

090177e19c2fb240\Approved\Approved On: 23-Dec-2022 10:25 (GMT)

Document History

Document	Version Date	Summary of Changes and Rationale
Amendment 8	17 June 2021	The primary purpose of this amendment is to update the analysis trigger of IA1 (futility analysis only) for the DDR-deficient cohort and to introduce a second interim analysis for efficacy for the DDR-deficient cohort (section 9.1).<br><br>Additional changes/clarifications have been done throughout the protocol:<br><br>- In the statistical methods added an IA for futility in the DDR-deficient population at the final rPFS analysis for all comers and updated the definition of PFS2 Extended window of obtaining consent from 28 to 42 days from randomization in table 1 and added information about contact cards. Specified that serum folate will need to be measured (not RBC folate). Reiterated that bone disease must be assessed only by bone scan.<br><br>- In table 3, scans frequency per SOC will be accepted for patients on follow up cancer therapy<br><br>- Section 1.3.2 provided a safety update from the phase 2 study of talazoparib in DDR-deficient patients with mCRPC<br><br>- Updated section 5.6.3<br><br>- Section 5.9 added statement that COVID-19 vaccines are allowed and clarified that any anticoagulant allowed per local enzalutamide prescribing information can be used in lieu of rivaroxaban and apixaban<br><br>- Updated Sections 9.1 and 9.8.1 (introduction of IAs)<br><br>- Section 9.5 added language to clarify the potential for early PK unblinding

090177e19c2fb240\Approved\Approved On: 23-Dec-2022 10:25 (GMT)

Document	Version Date	Summary of Changes and Rationale
		- Appendices 2, 3, 4 and 5 removed due to copyrightsOther clarifications done throughout the protocol
Amendment 7	18 September 2020	The primary purpose of this amendment is to introduce an extension cohort to ensure at least 113 mCRPC patients are randomized in China. The additional patients randomized in the extension cohort will not be included in the analysis of the primary or secondary study endpoints. Data from the China cohort will be analyzed separately per local regulatory requirement (see Appendix 10).
Amendment 6	26 February 2020	The primary purpose of the amendment is to introduce the use of liquid biopsies to assess the patient's tumor DDR status at pre-screening or screening. Concordance of DDR testing results between liquid and tumor tissue biopsies has been added as exploratory endpoint. Both samples must be provided prior to randomization (Table 1, Table 5, Sections 3, 4, 5.1, 6.1, 6.2, 7.1).<br><br>In addition, the following changes or clarifications were introduced:<br><br>• Table 3: collection of skeletal events should continue in long term follow up. This was added to align with the text in Section 6.5 of the prior protocol version.<br><br>• In the study design (Section 3) removed redundant information and clarified that all randomized patients can receive surgery or palliative radiotherapy after radiographic progression (not only patients from France).<br><br>• Inclusion criteria 4 and 5 were updated to reflect the introduction of liquid biopsies in the study, the PSA progression definition at study entry was clarified in criterion 8, legal guardian was removed from criterion 15 since not applicable for this patient population. Clarified in exclusion criterion 1 that the patient may have received first generation anti-androgens in the

090177e19c2fb240\Approved\Approved On: 23-Dec-2022 10:25 (GMT)

Document	Version Date	Summary of Changes and Rationale
		CRPC settings; in exclusion criterion 5 clarified that no washout period is required for prior abiraterone administered for treatment of castration sensitive disease. Also updated that >10 mg/day prednisone (or equivalent dose of other steroids) will be considered exclusionary and removed language regarding 5-alpha reductase inhibitor (use during study treatment is discussed in Section 5.9). Provided more information regarding eligibility based on prior cancer diagnoses and added reference to Appendix 9 which include a guidance on how to enter all the variables into the MDRD equation in exclusion criteria 16 and 11, respectively.<br><br>• In Sections 3, 4.3, 5.1, 6.1 and 7.1 and Table 1 added that if results from liquid and tumor tissue sample are available prior to randomization and results are not concordant for the presence of the selected DDR mutation(s), positive DDR results will be used for eligibility and stratification regardless of the tissue used to generate the data.<br><br>• Updated and clarified reportability of AE and research related injury during prescreening (Table 1 and Section 8).<br><br>• Removed the collection of the patient medical history at prescreening to avoid collecting data not required for analyses for patients that may not be randomized into the study (Table 1 and Section 6.1).<br><br>• Instructions were provided in Table 8 for the management of symptomatic Grade 1 and 2 anemia (eg, fatigue) to ensure the investigator will be able to manage the patient quality of life while on treatment.<br><br>• B12, reticulocyte count, erythropoietin, folate (introduced in protocol amendment 2 to explore the mechanism of talazoparib induced anemia) and assessment of bicarbonate (or CO_2) are no

090177e19c2fb240\Approved\Approved On: 23-Dec-2022 10:25 (GMT)

Document	Version Date	Summary of Changes and Rationale
		longer required if not available at the site laboratory (Table 12 footnote).<br><br>• Scans can be done per standard of care at the institution if patient is continuing study treatment after determination of progression by investigator and BICR (Table 2 footnote and Section 6.3).<br><br>• Table 11: text updated to align with text in Section 8.4.2.<br><br>• Section 8.4.4.1 included as medication error the lack of study drugs withholding per criteria provided in the protocol.<br><br>• Section 9.3.2 added planned analyses of objective response and PSA progression.<br><br>• Provided clarifications for biomarker samples collected in China in Section 7.5.1.<br><br>• Clarifications were added throughout the protocol considering the questions received from the sites (eg, in Section 6).
Amendment 5	28 Oct 2019	Changes at this time are specific for South Africa:<br><br>Optional HIV testing using the ELISA test may be conducted for subjects of unknown HIV status per investigator discretion. Subjects will be asked to sign an HIV consent form and be given pre and post test counseling at the site. (Added to Table 1, Section 6.2, Section 7.9 and Table 12).
Amendment 4	22 July 2019	The primary purpose of this amendment was to increase the patient size. N in Part 2 increased from 860 to 1018; Cohort 1 updated from 560 to 750; Cohort 2 updated from 300 to 268. The estimation of overlap of patients with DDR deficiency updated from ~85-140 to 112.<br><br>An exploratory endpoint was added to correlate to the previously listed exploratory objective.

090177e19c2fb240\Approved\Approved On: 23-Dec-2022 10:25 (GMT)

Document	Version Date	Summary of Changes and Rationale
		Clarification of the recording of AEs and Research Related Injuries if a de novo tumor biopsy is obtained at a prescreening visit (also in Sections 8.1.4 and 8.1.5).<br><br>Schedule of Activities: clarified when reticulocyte count, erythropoietin, folate, and B12 are to be collected; clarified when measurement of height is required for patients in Part 2; added an additional PSA at Week 53 so subsequent PSA required tests and site visits are on the same 8 week schedule; deleted optional repeat tumor biopsy at Safety Follow-Up visit or End of Treatment (also in Section 6.3); instructions for CTC sample specific for patients enrolled in China added; clarification of requirements for radiographic imaging if a patient continues on study treatment after disease progression documented radiographically but the patient is clinically benefiting (also in Section 6.3); clarified that the electronic Patient Reported Outcomes are to be completed remotely if telephone visits are being conducted (also in Section 6.5).<br><br>Sections 1.3.1 and 1.6.1 updated information regarding talazoparib added.<br><br>Section 1.5.1.5 clarification that the SmPC is the reference document for enzalutamide.<br><br>Number of study sites updated from 200 to 270.<br><br>Inclusion Criteria: (#8) definition of PSA progression updated to align with PCWG3 guidelines; (#9) clarification that the use of bisphosphonate or denosumab is allowed during the study but is not mandatory.<br><br>Exclusion Criteria: (#1) clarification that ADT in the castration resistant prostate cancer disease state is not exclusionary; (#3) clarification that patients who have previously received enzalutamide be excluded from the study. (This would be enzalutamide treatment at any time – including the CSPC disease state); (#4) clarification of the exclusionary time

090177e19c2fb240\Approved\Approved On: 23-Dec-2022 10:25 (GMT)

Document	Version Date	Summary of Changes and Rationale
		period of prior treatment with platinum base chemotherapy; (#5) clarification of cytotoxic chemotherapy; (#6) clarification of time period for previous investigational agents; (#7) clarification of previous opioid treatment; (#8) clarification of exclusionary medications; (#9) additional information regarding palliative localized radiation being exclusionary; (#16) clarification of exclusion due to cancer history.<br><br>Randomization criteria updated for clarification.<br><br>Section 5.5.1 clarified that study treatment should be administered in the morning of each day until PK testing is completed for each patient.<br><br>Section 5.6.2 clarified study treatment as talazoparib or talazoparib/placebo to refer to both treatment in Part 1 and in Part 2.<br><br>Table 8 anemia reference updated to specific laboratory value, not grade of AE.<br><br>Section 5.9 prohibited and allowable concomitant medications clarified.<br><br>Section 6.2 added information regarding biopsy to the screening visit as previously omitted in error. This information was previously included in the pre-screening section but not in the Screening section and it is permitted per the protocol to have these tests performed at the screening visit if the pre-screening visit was not done.<br><br>Section 7.5 clarification of biomarkers are being collected on all tested patients.<br><br>Section 7.5.1 added to clarify allowable tests per China regulatory agency.<br><br>Table 12 added urea as allowable where BUN is not collected; footnote c clarified that bicarbonate is not collected in Japan.<br><br>Section 9.1 sample size determination updated to reflect changes in analysis.

090177e19c2fb240\Approved\Approved On: 23-Dec-2022 10:25 (GMT)

Document	Version Date	Summary of Changes and Rationale
		Table 13 updated to reflect updated sample size population and analysis.<br><br>Section 9.8.1 updated to reflect the updated analyses from updated patient enrollment numbers.<br><br>Minor typographical changes have been made throughout the document for clarification.
Amendment 3	12 March 2019	Changes at this time specific for France:<br><br>Study design:<br><br>• Updated to clarify allowable additional treatment, following consultation with Sponsor medical monitor, if a patient remains on study drug following radiographic progression.<br><br>• Updated discontinuation of study treatment(s) requirements for patients who met progressive disease eligibility criterion for the study solely per PSA progression, and PSA continues to rise while on treatment.
Amendment 2	30 October 2018	The primary purpose of Protocol Amendment 2 was to allow the inclusion of patients without DNA Damage Repair (DDR) deficiencies as well as those with unknown mutation status.<br><br>The study design for Part 2 was updated to begin enrollment with all-comers (DDR-deficient and non DDR-deficient and unknown DDR status). Once enrollment is completed in this cohort, enrollment will continue but will be restricted to a second cohort of patients with DDR deficiencies (Cohort 2).<br><br>The talazoparib dose in combination with enzalutamide to be used in the randomized portion of the study (Part 2) is specified in Protocol Amendment 2 based on assessment of preliminary safety and PK data from Part 1.

090177e19c2fb240\Approved\Approved On: 23-Dec-2022 10:25 (GMT)

Document	Version Date	Summary of Changes and Rationale
		A high-level summary of changes made to the protocol specific by section is as follows:<br><br>• Title updated to remove reference to DDR deficiency.<br><br>• Overview and Background and Rationale updated with data from 2018 talazoparib IB.<br><br>• Part 2 Primary Objectives and Endpoints, Sample Size, Randomization Stratification, per updated study design.<br><br>• Schedule of Activities updated to reflect different visit schedule, patient contraception requirements and use added to screening, allowance of specific local laboratory evaluations, additional CTC collection at screening, and an additional PK sample at Weeks 3 and 17.<br><br>• Inclusion criteria #4 updated per Study Design update and eligibility of patients with or without DDR deficiencies.<br><br>• Inclusion #13, #14, and exclusion #18 updated to clarify required use and length of time for condom, contraception use, and sperm donation; not allowed to 4 months for patients that in the opinion of the investigator are capable of ejaculating.<br><br>• Exclusion #1 updated to clarify exclusionary prior systemic cancer treatment.<br><br>• Exclusion #5 updated to clarify prednisone >10 mg/day is exclusionary.<br><br>• Exclusion #16 deleted time periods for history of MDS or AML.<br><br>• Exclusion #21 added per enzalutamide safety language.

090177e19c2fb240\Approved\Approved On: 23-Dec-2022 10:25 (GMT)

Document	Version Date	Summary of Changes and Rationale
		• Contraception language updated per 2018 talazoparib IB.<br>• Information regarding talazoparib overdose added per 2018 talazoparib IB.<br>• Added information regarding talazoparib/placebo continuing if enzalutamide is permanently discontinued and enzalutamide continuing if talazoparib/placebo is permanently discontinued.<br>• Timeframe to allow anemia to resolve prior to talazoparib/placebo discontinuation updated from 4 to 8 weeks.<br>• End of treatment requirements clarified.<br>• Efficacy assessments (Part 2) per updated study design.<br>• Clarified non DDR deficiency and unknown DDR status.<br>• Data Analysis and Statistical Methods (Part 2) per updated study design.<br>• General Data Protection Regulation (GDPR) language added.<br>• Other administrative and/or editorial changes were also incorporated to improve overall document clarity and accuracy.<br>Typographical corrections made throughout the document.
Amendment 1	30 March 2018	Title of Study updated to reflect the deletion of abiraterone treatment arm.<br>Study Design:<br>• Deleted the abiraterone treatment arm from both Part 1 and Part 2.

090177e19c2fb240\Approved\Approved On: 23-Dec-2022 10:25 (GMT)

Document	Version Date	Summary of Changes and Rationale
		• Sample size of patients in both study parts adjusted accordingly (Part 1 to 12; Part 2 to 340).<br><br>• Clarification provided to explain how Part 1 safety data from targeted safety events and PK analysis will be assessed for confirmation of the starting talazoparib daily dose in Part 2; tolerability added to evaluation in Part 1.<br><br>Schedule of Activities and Study Procedures:<br><br>• Separated optional Prescreen visit activities and Screen activities.<br><br>• Clarification that if a prescreen visit is done collection of demography, history of prostate cancer, and prior cancer therapy is to be completed at this visit.<br><br>• Visit windows ±3 days, and treatment is to be started within 3 days after randomization per SOA footnote 'e'.<br><br>• Deleted the single item questionnaire on identity of treatment received.<br><br>• Clarified that tumor tissue (de novo or archival) is to be done at Prescreen or Screen visit; not Day 1 visit.<br><br>• Added the option of taking PSA lab test at an unscheduled visit.<br><br>• PK collection in Part 2 deleted from Weeks 1 and 17, and added to unscheduled visit to ease patient burden.<br><br>• CTC and ctDNA analyses deleted from Week 5 and unscheduled, and added to safety follow-up visit.<br><br>• Footnote clarified to indicate study visits following Week 25 while still on study drug are

090177e19c2fb240\Approved\Approved On: 23-Dec-2022 10:25 (GMT)

Document	Version Date	Summary of Changes and Rationale
		to occur every 60 days for accurate drug dispensing.<br><br>• Footnote to clarify 2 x 20 ml blood samples are to be collected for DNA analysis prior to first dose of study drug.<br><br>• Upon PSA response (decline) or progression a consecutive confirmation lab value is required at least 21 days later.<br><br>Safety and Efficacy data of talazoparib updated with most recent information.<br><br>Rationale for enzalutamide dose added.<br><br>Objectives:<br><br>• Part 1: added an additional exploratory objective "to explore correlations between talazoparib/enzalutamide exposure and biomarker, efficacy and safety outcome parameters, if data allow".<br><br>• Part 2: deleted exploratory objective "to explore whether talazoparib in combination with NHT induces a high degree of PSA response".<br><br>Endpoints:<br><br>• Part 2: Included details of Patient Reported Outcome assessment measurements.<br><br>Study Schematic updated to reflect amended study design.<br><br>Subject Eligibility.<br><br>Inclusion Criteria:<br><br>• Clarification regarding type of confirmed adenocarcinoma of the prostate without small cell or signet cell features.

090177e19c2fb240\Approved\Approved On: 23-Dec-2022 10:25 (GMT)

Document	Version Date	Summary of Changes and Rationale
		• Allow historic genomic testing of tumor tissue showing DDR positivity on selected genes following sponsor approval.<br><br>• Clarification allowing patients who are on prophylactic opioid therapy for pain related to their prostate cancer diagnosis as long as no pain related to prostate cancer has been reported in the 28 days prior to enrollment (Part 1) or randomization (Part 2).<br><br>• Clarification of the purpose of the saliva sample collection.<br><br>• Relative time point for previous medications clarified as enrollment (Part 1) or randomization (Part 2).<br><br>• Definition of PSA progression must be from 3 consecutive assessments.<br><br>• Increased the length of time for contraception use and sperm donation from 105 to 120 days.<br><br>Exclusion Criteria:<br><br>• Previous cancer treatment initiated in the mCRPC state updated to be any prior systemic cancer treatment.<br><br>• Clarification that patients whose only evidence of metastasis is adenopathy below the aortic bifurcation are excluded.<br><br>• Prior platinum-based chemotherapy is only exclusionary if administered within the previous 6 months.<br><br>• Concomitant medications due to potential DDI with talazoparib updated.<br><br>• Concomitant medications due to potential DDI with enzalutamide updated.

090177e19c2fb240\Approved\Approved On: 23-Dec-2022 10:25 (GMT)

Document	Version Date	Summary of Changes and Rationale
		• Exclusionary value for albumin updated to <2.8 g/dL.<br><br>• Added an exclusionary criterion if patient is not willing to comply with protocol contraception requirements.<br><br>Stratification Factors for Randomization Criteria: Updated due to change in study design to (1) previous treatment with NHT for castration-sensitive prostate cancer or non-metastatic castration-resistant prostate cancer (M0 CRPC) (yes/no), and (2) previous treatment with taxane-based chemotherapy for castration-sensitive prostate cancer (yes/no).<br><br>PK analyses updated to clarify inclusion of enzalutamide N-desmethyl metabolite.<br><br>Breaking the Blind procedure updated per Pfizer SOP language.<br><br>Investigational Product Supplies updated with details of talazoparib and identical placebo, and enzalutamide.<br><br>Clarification that dispensing of study drugs in Part 1 will be performed manually by the sites.<br><br>Clarification that in Part 2 the starting dose of talazoparib in patients with moderate renal impairment will be determined based on the starting dose for patients with normal renal function for Part 2.<br><br>Instructions included if a patient forgets to take his daily dose of both blinded study drug and enzalutamide.<br><br>Concomitant Treatments:<br><br>• Updated with details of specific treatments allowed, prohibited, and information regarding the use of anticoagulants in the study.

090177e19c2fb240\Approved\Approved On: 23-Dec-2022 10:25 (GMT)

Document	Version Date	Summary of Changes and Rationale
		• Clarification added for the dose of enzalutamide to be reduced to 80 mg/day if strong cytochrome P450 2C8 inhibitors are administered.<br><br>Companion Diagnostics updated to clarify that de novo, archival or historical result (with Sponsor pre-approval) must be done by Foundation Medicine using the FoundationOne® test.<br><br>Details added regarding the study biomarkers and banked biospecimens.<br><br>Adverse Events:<br><br>• Additional medical guidance for treatment of anemia, neutropenia, and thrombocytopenia included in Table 7.<br><br>• The time period for collection of SAEs updated to 28 calendar days without regard of initiation of antineoplastic or investigational therapy.<br><br>• Clarification of reporting of potential research related injury events following de novo tumor biopsy if performed at a prescreening visit.<br><br>• Emphasis that all events of myelodysplastic syndrome or acute myeloid leukemia are to be reported as SAEs irrespective of time of diagnosis or investigators opinion of causality.<br><br>• Update to version 4.03 of CTCAE for the investigator's definitions of severity of adverse events.<br><br>Medication Errors updated to include:<br><br>• Incorrect study drug dose taken by patient.<br><br>• Approximately <80% of expected dosing.<br><br>Patient Reported Outcomes:<br><br>• Clarification throughout the document that Patient Reported Outcome assessments are only

090177e19c2fb240\Approved\Approved On: 23-Dec-2022 10:25 (GMT)

Document	Version Date	Summary of Changes and Rationale
		to be completed by patients in Part 2 of the study.<br><br>• Analyses of Patient Reported Outcomes detailed.<br><br>Data Analysis:<br><br>• Part 2 Key Secondary Endpoints analyses detailed.<br><br>• Analysis of radiographic progression free survival updated to be based on the updated 2 stratification factors.<br><br>Interim Analysis updated to be one interim analysis for futility.<br><br>Definition of End of Trial updated to indicate last patient last visit for all countries.
Original protocol	Final 28 July-2017	Not applicable (N/A)

This amendment incorporates all revisions to date, including amendments made at the request of country health authorities and institutional review boards (IRBs)/ethics committees (ECs).

090177e19c2fb240\Approved\Approved On: 23-Dec-2022 10:25 (GMT)

TABLE OF CONTENTS

LIST OF TABLES 23
LIST OF FIGURES 23
PROTOCOL SUMMARY 24
SCHEDULE OF ACTIVITIES 37
1. INTRODUCTION 47
1.1. Overview and Mechanism of Action 47
1.1.1. Overview 47
1.1.2. Mechanism of Action/Indication 47
1.2. Background and Rationale 48
1.2.1. Prostate Cancer 48
1.2.2. PARP Inhibition 49
1.2.3. PARP Inhibitors as a Potential Targeted Therapy in mCRPC, Including in Androgen Pathway Manipulation 49
1.3. Summary of Relevant Clinical Experience with Talazoparib 51
1.3.1. Efficacy 52
1.3.2. Safety 53
1.3.3. Pharmacokinetics and Metabolism 54
1.3.3.1. Talazoparib 54
1.3.3.2. Enzalutamide 55
1.3.3.3. Drug Drug Interactions between Talazoparib and Enzalutamide 56
1.4. Talazoparib Benefits and Risks Assessment 56
1.5. Enzalutamide Benefits and Risks Assessments 57
1.6. Rationale for Combination Treatment 59
1.6.1. Rationale for Study Treatment Dose 60
1.6.1.1. Talazoparib 60
1.6.1.2. Enzalutamide 61
2. STUDY OBJECTIVES AND ENDPOINTS 61
3. STUDY DESIGN 65
3.1. Part 1: Open-Label Treatment 70
3.2. Part 2: Double-Blind Treatment 70
4. PATIENT ELIGIBILITY CRITERIA 72

090177e19c2fb240\Approved\Approved On: 23-Dec-2022 10:25 (GMT)

4.1. Inclusion Criteria 72
4.2. Exclusion Criteria 74
4.3. Randomization Criteria 77
4.4. Lifestyle Requirements 78
4.4.1. Contraception 78
4.5. Sponsor's Qualified Medical Personnel 79
5. STUDY TREATMENTS 79
5.1. Allocation to Treatment 79
5.2. Breaking the Blind 80
5.3. Patient Compliance 81
5.4. Investigational Product Supplies 81
5.4.1. Dosage Forms and Packaging 81
5.4.1.1. Talazoparib and Identical Placebo 81
5.4.1.2. Enzalutamide 81
5.4.2. Preparation and Dispensing 81
5.5. Administration 82
5.5.1. Talazoparib or Blinded Therapy 82
5.5.1.1. Talazoparib Overdose 83
5.5.2. Enzalutamide 83
5.6. Dose Modifications 83
5.6.1. Part 1: Talazoparib Dose Determination 83
5.6.2. Study Treatment Dose Modification 84
5.6.2.1. Dose Modifications for Talazoparib or Talazoparib/Placebo Due to Adverse Events 84
5.6.2.2. Dose Modifications for Enzalutamide Due to Adverse Events 87
5.6.2.3. Talazoparib or Talazoparib/Placebo Dose Escalation Only if Enzalutamide Permanently Discontinued 87
5.6.3. Modifications Due to Abnormal Liver Tests (All Treatments) 88
5.6.4. End of Treatment 90
5.7. Investigational Product Storage 90
5.8. Investigational Product Accountability 91
5.8.1. Destruction of Investigational Product Supplies 92

090177e19c2fb240\Approved\Approved On: 23-Dec-2022 10:25 (GMT)

5.9. Concomitant Treatment(s) 92
5.10. Rescue/Salvage Medication 94
6. STUDY PROCEDURES 94
6.1. Prescreening (optional) 94
6.2. Screening 95
6.3. Treatment Period (Part 1 and Part 2) 96
6.4. Safety Follow-up 98
6.5. Long-Term Follow-up 98
6.6. Patient Withdrawal 99
7. ASSESSMENTS 100
7.1. Companion Diagnostics for Eligibility 100
7.2. Efficacy Assessments 101
7.2.1. Primary Efficacy Endpoint: Radiographic Progression-Free Survival 101
7.2.2. Assessment of Secondary Efficacy Endpoints 102
7.2.3. Exploratory Efficacy Endpoint Assessments 103
7.3. Patient-Reported Outcomes Assessments 103
7.4. Pharmacokinetic Assessments 104
7.5. Biomarkers 104
7.5.1. China 106
7.6. Banked Biospecimens 106
7.6.1. Additional Research 107
7.7. Imaging Assessments 107
7.8. Rater Qualifications 108
7.9. Safety Assessments 108
7.9.1. Adverse Events 108
7.9.2. Physical Examinations 108
7.9.3. Vital Signs 108
7.9.4. Clinical Laboratory Assessments 108
8. ADVERSE EVENT REPORTING 110
8.1. Requirements 110
8.1.1. Additional Details On Recording Adverse Events on the CRF 111
8.1.2. Eliciting Adverse Event Information 111

090177e19c2fb240\Approved\Approved On: 23-Dec-2022 10:25 (GMT)

8.1.3. Withdrawal From the Study Due to Adverse Events (see also the Patient Withdrawal section) 111
8.1.4. Time Period for Collecting AE/SAE Information 111
8.1.4.1. Reporting SAEs to Pfizer Safety 112
8.1.4.2. Recording Non-serious AEs and SAEs on the CRF 112
8.1.5. Time Period for Collecting AEs and/or Research Related Injuries for Patients Who Sign the Molecular Prescreening Consent 112
8.1.5.1. Adverse Events 112
8.1.5.2. Research-Related Injuries 113
8.1.6. Causality Assessment 113
8.1.7. Sponsor's Reporting Requirements to Regulatory Authorities 114
8.2. Definitions 114
8.2.1. Adverse Events 114
8.2.2. Abnormal Test Findings 115
8.2.3. Serious Adverse Events 115
8.2.4. Hospitalization 116
8.3. Severity Assessment 117
8.4. Special Situations 118
8.4.1. Protocol-Specified Serious Adverse Events 118
8.4.2. Potential Cases of Drug-Induced Liver Injury 118
8.4.3. Exposure to the Investigational Product During Pregnancy or Breastfeeding, and Occupational Exposure 120
8.4.3.1. Exposure During Pregnancy 120
8.4.3.2. Exposure During Breastfeeding 121
8.4.3.3. Occupational Exposure 121
8.4.4. Medication Errors 121
8.4.4.1. Medication Errors 122
9. DATA ANALYSIS/STATISTICAL METHODS 123
9.1. Sample Size Determination 123
9.2. Analysis Sets 127
9.2.1. Intent-to-Treat Population 128
9.2.2. Safety Population 129
9.2.3. Patient-Reported Outcome Population 129

090177e19c2fb240\Approved\Approved On: 23-Dec-2022 10:25 (GMT)

9.2.4. Pharmacokinetic Analysis Population 129
9.3. Efficacy Analysis 129
9.3.1. Primary Endpoint: Radiographic PFS 129
9.3.2. Analysis of Secondary Efficacy Endpoints 130
9.3.3. Analysis of Exploratory Endpoints 131
9.4. Patient-Reported Outcome Analyses 131
9.5. Pharmacokinetic Analyses 132
9.6. Biomarker Analyses 133
9.7. Safety Analysis 134
9.7.1. Adverse Events 134
9.7.2. Analysis of Clinical Laboratory Results 134
9.8. Interim Analysis 135
9.8.1. Primary Endpoint 135
9.9. Data Monitoring Committee 136
10. QUALITY CONTROL AND QUALITY ASSURANCE 136
11. DATA HANDLING AND RECORD KEEPING 137
11.1. Case Report Forms/Electronic Data Record 137
11.2. Record Retention 137
12. ETHICS 138
12.1. Institutional Review Board/Ethics Committee 138
12.2. Ethical Conduct of the Study 138
12.3. Patient Information and Consent 138
12.4. Reporting of Safety Issues and Serious Breaches of the Protocol or ICH GCP 140
13. DEFINITION OF END OF STUDY 140
13.1. End of Study in All Participating Countries 140
14. SPONSOR DISCONTINUATION CRITERIA 140
15. PUBLICATION OF STUDY RESULTS 140
15.1. Communication of Results by Pfizer 140
15.2. Publications by Investigators 141
16. REFERENCES 143
17. SUPPORTING DOCUMENTATION AND OPERATIONAL CONSIDERATIONS 149

090177e19c2fb240\Approved\Approved On: 23-Dec-2022 10:25 (GMT)

17.1. Appendix 1: Abbreviations 149
17.2. Appendix 2: France Appendix 154
17.3. Appendix 3: GCP Training 154
17.4. Appendix 4: Investigational Product 154
17.5. Appendix 5: eGFR Calculation Prior to Randomization 155
17.6. Appendix 6: China-Specific Requirements 156
17.7. Cohort Appendix 7: Country-Specific Requirements 157

090177e19c2fb240\Approved\Approved On: 23-Dec-2022 10:25 (GMT)

LIST OF TABLES

Table 1. Study Schedule of Activities: Prescreening (Optional) and Screening 37
Table 2. Study Schedule of Activities: Treatment Period 41
Table 3. Study Schedule of Activities: Long-term Follow-up.....45
Table 4. Objectives and Endpoints for Study Part 1 (Open-Label Treatment).....61
Table 5. Objectives and Endpoints for Study Part 2 (Double-Blind Treatment).....62
Table 6. Target Safety Events: Part 1 (Open-Label Treatment) 84
Table 7. Talazoparib or Talazoparib/Placebo Dose Modification Levels for Adverse Reactions.....85
Table 8. Dose Modification of Talazoparib or Talazoparib/Placebo Due to Adverse Events 85
Table 9. Talazoparib or Talazoparib/Placebo Dose Escalation After 6 Weeks of Enzalutamide Discontinuation.....88
Table 10. Criteria for Temporary Withholding of Study Treatment in Association With Liver Test Abnormalities.....88
Table 11. Monitoring of Liver Tests for Potential Drug-Induced Liver Injury.....90
Table 12. Required Laboratory Tests (fasting is not mandatory) 109
CCI [redacted]

LIST OF FIGURES

Figure 1. Dual Cytotoxic Mechanisms of PARP Inhibitors.....47
Figure 2. Study Schematic 69
Figure 3. China Cohort.....156

090177e19c2fb240\Approved\Approved On: 23-Dec-2022 10:25 (GMT)

PROTOCOL SUMMARY

Overview

Talazoparib (also known as PF-06944076, MDV3800) in combination with enzalutamide is being investigated for the treatment of metastatic castration-resistant prostate cancer (mCRPC) unselected for deoxyribonucleic acid (DNA) damage repair (DDR) deficiencies (referred to as "all-comers population"). Although patients will be unselected for DDR status in the all-comers population, DDR deficiency assessment prior to randomization is required for stratification.

Enrollment will begin in the all-comers population, Cohort 1. Once enrollment is complete in Cohort 1, enrollment will continue but will be restricted to patients with DDR deficiencies (Cohort 2).

Analysis on all-comers will include patients randomized into the DDR unselected cohort (Cohort 1). Analysis on the DDR-deficient population will include DDR-deficient patients enrolled in Cohort 2 as well as any patients enrolled in Cohort 1 who are DDR-deficient.

Exploratory subgroup analyses in patients with mCRPC not harboring DDR deficiencies will be conducted.

Background and Rationale

Castration-resistant prostate cancer represents a lethal transition in the progression of prostate cancer, with most patients ultimately succumbing to the disease. Prior to the recent approval of novel hormonal therapies (NHT) (enzalutamide, abiraterone acetate/prednisone), the only approved therapies for mCRPC were docetaxel, cabazitaxel and sipuleucel-T. The approval of NHT in mCRPC previously treated with docetaxel[7,12] represented a therapeutic advance for these patients, followed shortly thereafter by their approvals for the larger population of men with chemotherapy-naïve mCRPC.

Poly (adenosine diphosphate [ADP]-ribose) polymerase 1 (PARP1) and PARP2 play important roles in DNA repair.[25,26] Inhibition of PARP catalytic activity results in accumulation of single-strand DNA breaks, which in turn leads to irreparable double-strand DNA breaks in cells with defective homologous recombination mechanisms. Moreover, PARP inhibitors also trap the PARP enzyme on DNA to a variable extent, including that of tumor cells, thereby preventing DNA replication and transcription.[1,33]

Talazoparib is a potent, small molecule PARP inhibitor in development for the treatment of a variety of human cancers. Talazoparib is a particularly potent PARP trapper, a property that is associated with cytotoxicity in preclinical models.[1] Single-agent treatment with talazoparib demonstrates potent antitumor effects in tissue culture studies, mouse tumor xenograft models, and in Phase 1 studies in patients with solid tumors. Talazoparib has also been shown to enhance the cytotoxic effects of DNA-damaging chemotherapy.

090177e19c2fb240\Approved\Approved On: 23-Dec-2022 10:25 (GMT)

Proof of concept of PARP inhibitor efficacy in prostate cancer with DDR deficiency was established in a Phase 2 study (TOPARP-A) with olaparib, which enrolled men heavily pre-treated with taxane-based chemotherapy and NHT.[33] Furthermore, the potential for improved efficacy via combination of PARP inhibitors with NHT is being evaluated in ongoing Phase 1 and 2 studies.

Data presented at the 2018 American Society of Clinical Oncology (ASCO) Annual Meeting[35] and published in Lancet Oncology, from a Phase 2 study of 142 men with mCRPC regardless of mutation status, revealed that the combination of PARP inhibitor olaparib with the antiandrogen agent abiraterone acetate reduced the risk of disease progression or death by 35% compared with abiraterone alone in pretreated patients with mCRPC (hazard ration [HR]: 0.65; 95% confidence interval: 0.44-0.97; p-value 0.034). Moreover homologous recombination repair (HRR) mutation status was available for only 56 (39%) of the 142 patients. Of these patients, 21 had HRR mutations and 35 did not. In the cohort of 21 HRRm patients, 11 received the combination and 10 were treated with abiraterone alone. The median progression free survival (PFS) was 17.8 months in the experimental arm compared with 6.5 months in the control arm (HR, 0.74; 95% CI, 0.26-2.12). Among 35 homologous recombination repair mutation status (HRRm) wild-type patients, the median PFS was 15.0 months for 15 patients receiving olaparib/abiraterone compared with 9.7 months for 20 patients receiving abiraterone alone (HR, 0.52; 95% CI, 0.24-1.15). Therefore, this Phase II trial results suggested that targeting antiandrogen pathway with abiraterone in combination with PARP inhibitor olaparib significantly improved median PFS among patients with metastatic castration-resistant prostate cancer.[48]

The current study was designed given the results of TOPARP-A, the recently published results of olaparib in combination with abiraterone, and the role of NHTs such as enzalutamide in the treatment of mCRPC.

Rationale for combining PARP inhibitors with androgen-receptor (AR) signaling inhibitors in castration-resistant prostate cancer (CRPC) regardless of DDR mutational status is based on research on nonclinical models and clinical samples. AR signaling inhibition suppresses the expression of homologous recombination repair genes including breast cancer 1 (BRCA1), resulting in "BRCAness" and sensitivity to PARPi. PARP1 activity has been shown to be required for maximal AR function and thus inhibiting PARP is expected to reduce AR signaling and increase sensitivity to NHT. Clinical resistance to AR blockade is sometimes associated with co-deletion of retinoblastoma RB and BRCA2, which is in turn associated with sensitivity to PARP inhibition.

Recently, several investigations are focusing on the identification of BRCAness phenotype changes in sporadic cancers that would lead to similar treatment tumor sensitivity. The therapeutic aim of induction of BRCAness is to expand the use of PARP inhibitors to other tumor types whereas additional inhibition of other DNA repair pathways might lead to synthetic lethality in HR-deficient cells. Multiple lines of evidence link androgen receptor (AR) signaling to the DNA damage response (DDR) in prostate cancer cells and preclinical studies suggested that BRCAness phenotype can be achieved with a therapeutic combination of antiandrogen (enzalutamide) and PARP inhibitor given the ability of enzalutamide to

090177e19c2fb240\Approved\Approved On: 23-Dec-2022 10:25 (GMT)

down-regulate the expression of genes encoding components of cellular HR machinery. Hence, concomitant administration of these 2 classes of drugs can pharmacologically induce BRCAness and expand the clinical use of PARP inhibitors in the treatment of mCRPC.[43]

Objectives and Endpoints

Part 1 (Open-Label Treatment)

Primary Objective

To determine the starting dose of talazoparib when given in combination with enzalutamide during Part 2 (double-blind treatment period).

Secondary Objectives

To characterize the steady state pharmacokinetics (PK) of talazoparib and enzalutamide and its N-desmethyl metabolite when given in combination.

Exploratory Objectives

- To explore correlation of changes in circulating tumor cells (CTCs) with efficacy outcome parameters.
- To identify potential biomarkers associated with response and/or resistance to talazoparib in combination with enzalutamide.
- To explore correlations between talazoparib/enzalutamide exposure and biomarker, efficacy and safety outcome parameters if data allow.
- To collect banked biospecimens for exploratory research, unless prohibited by local regulations or ethics committee decision.

Part 2 (Double-Blind Treatment)

Primary Objectives

- To demonstrate that talazoparib in combination with enzalutamide is superior to placebo in combination with enzalutamide in prolonging blinded independent central review (BICR) assessed rPFS in patients with mCRPC unselected for DDR status.
- To demonstrate that talazoparib in combination with enzalutamide is superior to placebo in combination with enzalutamide in prolonging BICR assessed rPFS in patients with mCRPC harboring DDR deficiencies.

090177e19c2fb240\Approved\Approved On: 23-Dec-2022 10:25 (GMT)

Secondary Objectives

Key Secondary Objectives:

- To demonstrate that talazoparib in combination with enzalutamide is superior to placebo in combination with enzalutamide in prolonging overall survival (OS) in patients with mCRPC unselected for DDR status.

- To demonstrate that talazoparib in combination with enzalutamide is superior to placebo in combination with enzalutamide in prolonging OS in patients with mCRPC harboring DDR deficiencies.

Other Secondary Objectives:

- To evaluate anti-tumor activity in patients with mCRPC unselected for DDR status and in patients with mCRPC harboring DDR deficiencies with respect to the following:

    - BICR assessed objective response in measurable soft tissue disease;

    - BICR assessed duration of response in measurable soft tissue disease;

    - Prostate-Specific Antigen (PSA) Response;

    - Time to PSA progression;

    - Time to initiation of cytotoxic chemotherapy;

    - Time to initiation of antineoplastic therapy;

    - Time to first symptomatic skeletal event;

    - Progression free survival (PFS) on next line therapy (PFS2);

    - Opiate use for prostate cancer pain.

- To evaluate the safety of talazoparib and enzalutamide administered in combination.

- To evaluate the PK of talazoparib and enzalutamide (and its N-desmethyl metabolite) when dosed in combination.

- To evaluate the following patient-reported outcomes in each treatment arm in patients with mCRPC unselected for DDR status and in patients with mCRPC harboring DDR deficiencies:

    - Pain symptoms;

090177e19c2fb240\Approved\Approved On: 23-Dec-2022 10:25 (GMT)

- Cancer-specific global health status/Quality of life (QoL), functioning, and symptoms;
- General health status.

Exploratory Objectives

- To explore BICR assessed rPFS in patients with mCRPC without DDR deficiencies.
- To explore correlation of changes in circulating tumor cells (CTCs) with efficacy outcome parameters.
- To identify potential biomarkers associated with response and/or resistance to talazoparib in combination with enzalutamide.
- To explore correlations between talazoparib exposure and biomarker, efficacy and safety endpoints if data allow.
- To collect banked biospecimens for exploratory research, unless prohibited by local regulations or ethics committee decision.
- To explore concordance of DDR deficiency results between the blood and tumor tissue based tests.

Endpoints

Part 1

Primary Endpoint

- Occurrence of target safety events.

Secondary Endpoint

- Multiple-dose PK parameters of talazoparib and enzalutamide and its N-desmethyl metabolite: Multiple-dose maximum plasma concentration (C_{max}), lowest plasma concentration of drug directly before next dose (C_{trough}), time to reach peak concentration following drug administration (T_{max}), area under the plasma concentration-time curve over the dosing interval (from time zero to 24 hours post-dose) (AUC_{τ}), and apparent oral clearance (CL/F) as data permit.

090177e19c2fb240\Approved\Approved On: 23-Dec-2022 10:25 (GMT)

Exploratory Endpoints

- Proportion of patients with conversion from 5 or more CTCs per 7.5 mL at baseline to 4 or fewer CTCs per 7.5 mL post-baseline.
- Proportion of patients with conversion from detectable CTCs per 7.5 mL at baseline to CTCs = 0 per 7.5 mL post-baseline.
- Proportion of patients with baseline CTCs <5 who show increased CTCs post-baseline.
- Molecular profiling of tumor tissue remaining after genomic testing for eligibility and of saliva and circulating tumor deoxyribonucleic acid (ctDNA); circulating protein biomarker profiles.
- Potential results from exploratory analyses of banked biospecimens.

Part 2

Primary Endpoints

- BICR assessed rPFS per Response Evaluation Criteria in Solid Tumors (RECIST) 1.1 (soft tissue disease) and Prostate Cancer Working Group (PCWG3) (bone disease) in patients with mCRPC unselected for DDR status.
- BICR assessed rPFS per RECIST 1.1 (soft tissue disease) and PCWG3 (bone disease) in patients with mCRPC harboring DDR deficiencies.

Key Secondary Endpoints (alpha-protected)

- OS inpatients with mCRPC unselected for DDR status (alpha-protected).
- OS in patients with mCRPC harboring DDR deficiencies (alpha-protected).

Other Secondary Endpoints

- Proportion of patients with measurable soft tissue disease at baseline with an objective response per RECIST 1.1 (assessed by the BICR) in patients with mCRPC unselected for DDR status and in patients with mCRPC harboring DDR deficiencies.
- Duration of soft tissue response per RECIST 1.1 (assessed by the BICR) in patients with mCRPC unselected for DDR status and in patients with mCRPC harboring DDR deficiencies.
- Proportion of patients with PSA response ≥50% in patients with mCRPC unselected for DDR status and in patients with mCRPC harboring DDR deficiencies.

090177e19c2fb240\Approved\Approved On: 23-Dec-2022 10:25 (GMT)

- Time to PSA progression in patients with mCRPC unselected for DDR status and in patients with mCRPC harboring DDR deficiencies.
- Time to initiation of cytotoxic chemotherapy in patients with mCRPC unselected for DDR status and in patients with mCRPC harboring DDR deficiencies.
- Time to initiation of antineoplastic therapy in patients with mCRPC unselected for DDR status and in patients with mCRPC harboring DDR deficiencies.
- Time to first symptomatic skeletal event in patients with mCRPC unselected for DDR status and in patients with mCRPC harboring DDR deficiencies.
- PFS2 based on investigator assessment in patients with mCRPC unselected for DDR status and in patients with mCRPC harboring DDR deficiencies.
- Time to opiate use for prostate cancer pain in patients with mCRPC unselected for DDR status and in patients with mCRPC harboring DDR deficiencies.
- Incidence of adverse events characterized by type, severity (graded by National Cancer Institute [NCI] Common Toxicity Criteria for Adverse Events [CTCAE] version 4.03), timing, seriousness and relationship to study treatment.
- PK characterized by pre-dose trough and post-dose plasma concentrations of talazoparib and enzalutamide and its N-desmethyl metabolite.
- Patient Reported Outcomes (PROs) in patients with mCRPC unselected for DDR status and in patients with mCRPC harboring DDR deficiencies:
    - Change from baseline in patient-reported pain symptoms per brief pain inventory-short form (BPI-SF);
    - Change from baseline in patient-reported general health status per European Quality of Life 5-dimension, 5-level scale (EQ-5D-5L);
    - Change from baseline in patient-reported cancer-specific global health status/QoL, functioning, and symptoms per European Organisation for Research and Treatment of Cancer (EORTC) QLQ-C30;
    - Time to deterioration in patient-reported pain symptoms per BPI-SF;
    - Time to definitive deterioration in patient-reported global health status/QoL per European Organisation for Research and Treatment of Cancer cancer-specific global health questionnaire (EORTC QLQ-C30);
    - Time to definitive deterioration in patient-reported disease-specific urinary symptoms per European Organisation for Research and Treatment of Cancer disease specific urinary symptoms questionnaire (EORTC QLQ-PR25).

090177e19c2fb240\Approved\Approved On: 23-Dec-2022 10:25 (GMT)

Exploratory Endpoints

- BICR assessed rPFS per RECIST 1.1 (soft-tissue disease) and PCWG3 (bone disease) in patients with mCRPC without DDR deficiencies.

- Proportion of patients with conversion from 5 or more CTCs per 7.5 mL at baseline to 4 or fewer CTCs per 7.5 mL post-baseline, in patients with mCRPC unselected for DDR status and in patients with mCRPC harboring DDR deficiencies.

- Proportion of patients with conversion from detectable CTCs per 7.5 ml at baseline to CTCs = 0 per 7.5 ml post-baseline, in patients with mCRPC unselected for DDR status and in patients with mCRPC harboring DDR deficiencies.

- Proportion of patients with baseline CTC counts <5 who show increased CTC counts post-baseline, in patients with mCRPC unselected for DDR status and in patients with mCRPC harboring DDR deficiencies.

- Molecular profiling of tumor tissue remaining after genomic testing for eligibility during prescreening/screening, of saliva, and ctDNA; circulating protein biomarker profiles.

- Concordance of DDR deficiency results between the blood and tumor tissue based tests.

Study Design

This is an international, Phase 3, two-part study enrolling patients with mCRPC where no systemic cancer treatments have been initiated after documentation of CRPC with the exception of androgen deprivation therapy and first generation anti-androgens.

Part 1 was open-label and non-randomized and evaluated the safety, tolerability, and PK of talazoparib in combination with enzalutamide. Part 2 is randomized, double-blind, and placebo-controlled and will evaluate the safety and efficacy of talazoparib in combination with enzalutamide compared with placebo in combination with enzalutamide. Part 2 will enroll two patient cohorts. The first cohort (Cohort 1) will enroll approximately 750 mCRPC patients unselected for DDR status (all-comers population). Although patients will be unselected for DDR status, DDR deficiency assessment prior to randomization is required for stratification.

After enrollment in Cohort 1 is completed, enrollment will continue but will be restricted to patients with DDR deficiencies (Cohort 2). Cohort 2 will enroll approximately 268 additional patients harboring DDR gene mutations likely to sensitize to PARP inhibition (DDR-deficient). Patients who are DDR-deficient from Cohorts 1 and 2 will be combined such that a total of approximately 380 DDR-deficient patients are enrolled to assess efficacy separately within this population.

090177e19c2fb240\Approved\Approved On: 23-Dec-2022 10:25 (GMT)

Approximately 1037 men (19 in Part 1 and approximately 1018 in Part 2) with mCRPC will be enrolled. Genomic screening to identify alterations in DDR genes is optional for patients in Part 1, but is required for randomization in Part 2. Mutational status for patients randomized in Part 2 will be determined by testing for the presence of mutations in defined DDR genes likely to sensitize to PARP inhibition using next generation sequencing (NGS) based gene panel test.

A patient will participate in up to 5 periods: prescreening (optional), screening, open-label (Part 1) or double-blind (Part 2), safety follow-up, and long-term follow-up. Testing for DDR gene mutational status will be performed at prescreening or screening.

The primary objective of Part 1 was to determine the starting dose of talazoparib in combination with enzalutamide to be used in Part 2. The first patients enrolled into Part 1 initially received a starting dose of talazoparib 1 mg once a day in combination with enzalutamide 160 mg once a day, since talazoparib has demonstrated efficacy as a single agent at a dose of 1 mg once a day in patients with germline BRCA mutation, human epidermal growth factor receptor 2 (HER2) negative and locally advanced and/or metastatic breast cancer.

Evaluation of available preliminary safety data from patients enrolled with a starting dose of talazoparib of 1 mg once daily in combination with 160 mg once daily enzalutamide showed higher than expected dose reductions and interruptions mainly due to Grade 3 hematological toxicities. Evaluation of available preliminary talazoparib PK data indicated that enzalutamide increased talazoparib C_{trough} at Week 5 Visit by ~2-fold compared to talazoparib steady-state C_{trough} in patients with advanced solid tumors and in patients with breast cancer receiving 1 mg once a day monotherapy. Based on these preliminary safety and PK data, talazoparib dose was reduced from 1 mg once a day to 0.5 mg once a day for all patients continuing to receive talazoparib in combination with enzalutamide in Part 1. Additional patients were enrolled in Part 1 and received a talazoparib starting dose of 0.5 mg once a day in combination with 160 mg once a day enzalutamide.

Safety and PK data from patients who received talazoparib at the 0.5 mg once a day talazoparib dose level either as a starting dose or for patients who started on 1 mg once a day and later reduced to 0.5 mg showed an acceptable safety profile and comparable C_{trough} to that achieved in patients with advanced solid tumors and in patients with breast cancer receiving 1 mg once a day. This indicates that reducing the talazoparib dose to 0.5 mg once a day in combination with enzalutamide is expected to account for the observed drug-drug interaction (DDI) and maintain similar talazoparib exposure and safety profile to that achieved with 1 mg once a day monotherapy. Therefore, a talazoparib dose of 0.5 mg once a day will be administered in combination with enzalutamide 160 mg once a day in Part 2 of this study. The starting dose of talazoparib for patients with moderate renal impairment will be 0.35 mg once a day to account for the lower talazoparib clearance in this subpopulation.

090177e19c2fb240\Approved\Approved On: 23-Dec-2022 10:25 (GMT)

In Part 2, patients will be randomized to receive either talazoparib or placebo in combination with open-label enzalutamide. For all patients, study treatment (including enzalutamide) should continue until radiographic progression is determined by blinded independent central review (BICR) (Part 2) or local review (Part 1), unless in the opinion of the investigator the patient is still deriving benefit at this time, or following radiographic progression the patient is then no longer clinically benefitting in the opinion of the investigator, or an adverse event leading to permanent discontinuation, or patient decision to discontinue treatment, or death. During Part 2, study treatment(s) should not be discontinued based solely on increases in PSA levels in the absence of documented radiographic disease progression.

Safety follow-up visit will occur approximately 28 days after the last dose of study treatment (either the combination or enzalutamide or talazoparib or talazoparib/placebo whichever is later) or before initiation of a new antineoplastic or investigational therapy, whichever occurs first.

Long-term follow-up will begin after safety follow-up and will follow the radiographic imaging schedule (ie, every 8 weeks through Week 25 and then every 12 weeks thereafter until death, withdrawal of consent for follow-up, or study termination by the Sponsor). In Part 2, patients for whom study treatment was permanently discontinued without radiographic progression will continue with per-protocol imaging in long-term follow-up until such radiographic progression is documented regardless of initiation of a new antineoplastic therapy.

Study Treatments

During Part 1, patients will receive talazoparib in combination with enzalutamide. During Part 2, patients will be randomized to one of two treatment groups: talazoparib in combination with enzalutamide, or matching placebo in combination with enzalutamide.

CCI [redacted]

090177e19c2fb240\Approved\Approved On: 23-Dec-2022 10:25 (GMT)

CCI [redacted]

Enrollment will begin in the all-comers population, Cohort 1. Once enrollment is complete in Cohort 1, enrollment will continue but will be restricted to patients with DDR deficiencies (Cohort 2).

Analysis on all-comers population will include patients randomized into the DDR unselected cohort (Cohort 1). Analysis of the DDR-deficient population data will include patients with DDR deficiencies from both Cohorts 1 and 2.

The intent-to-treat (ITT) population will be defined as all patients randomly assigned to double-blind study treatment. All safety analyses will use the safety population, defined as all patients who receive at least one dose of study treatment.

The PRO population will be defined as a subset of ITT patients in Part 2 who have completed a baseline and at least one post-baseline quality-of-life assessment prior to the end of study.

The primary endpoint of rPFS in Part 2 (evaluated separately in the all-comers population and the DDR-deficient population which are subsets of the ITT population) is defined as the time from the date of randomization to first objective evidence of radiographic progression as assessed in soft tissue per RECIST 1.1 or in bone (upon subsequent confirmation) per PCWG3[59] guidelines by BICR, or death, whichever occurs first. CCI [redacted]

090177e19c2fb240\Approved\Approved On: 23-Dec-2022 10:25 (GMT)

CCI [redacted]

Objective response rate will be evaluated in the subset of patients with measurable soft tissue disease at baseline. Confirmation of soft tissue response will be required. CCI [redacted]

The time from the first objective evidence of a complete response (CR) or partial response (PR) to the first objective evidence of disease progression or death (whichever occurs first) will be analyzed in the subset of ITT patients entering with measurable soft tissue disease and a reported CR or PR on study.

The proportion of patients with a ≥50% decline from baseline in PSA that is confirmed by a second consecutive value at least 21 days later will be calculated for each treatment arm CCI [redacted]

PFS2 is defined as time from randomization to investigatordocumented disease progression (PSA progression, progression on imaging, or clinical progression) on the first subsequent antineoplastic therapy for prostate cancer, or death from any cause, whichever occurs first.

Time to deterioration of pain (≥2-point increase from baseline question 3 of the BPI-SF) will be measured. Pain and analgesic assessments will be completed (via pain and analgesic logs, respectively) for 7 consecutive days before study visits; pain score averages during the period of reporting will be calculated. Analgesic data (from the analgesic log) will be mapped to the World Health Organization (WHO) analgesic usage score and used concurrently to define pain progression with the BPI-SF.

Time to definitive deterioration of patient-reported global health status/QoL and time to deterioration of patient-reported urinary symptoms will be summarized CCI [redacted] Definitive deterioration of patient-reported global health status/QoL is defined as ≥10-point decrease from baseline and no subsequent observations of a <10-point decrease from baseline. Definitive deterioration of patient-reported urinary symptoms is defined as ≥10-point increase from baseline and not subsequent observations of a <10-point increase from baseline.

CCI [redacted]

090177e19c2fb240\Approved\Approved On: 23-Dec-2022 10:25 (GMT)

Safety will be evaluated for the safety population using adverse event, laboratory and vital sign data. All adverse events (AEs) will be coded to preferred term and system organ class using current Medical Dictionary for Regulatory Activities (MedDRA). Treatment-emergent AEs will be presented with and without regard to causality based on the investigator's judgment and by frequency of overall toxicity categorized by NCI CTCAE (version 4.03) grades. Laboratory shift tables of baseline to maximum post-baseline results to each subsequent visit will be produced as appropriate.

For patients who participate in Part 1, the final PK data analyses will include calculation of steady state PK parameters (as specified in secondary endpoints for Part 1) of talazoparib, enzalutamide, and its N-desmethyl metabolite. For all patients (Part 1 and Part 2), PK data analyses will include descriptive summary statistics of the pre-dose trough concentration (C_{min}) and post-dose plasma concentrations of talazoparib, enzalutamide and its N-desmethyl metabolite by study visit and time point. In addition, the PK data from this study may be used to develop a population PK model. The correlation between exposure parameters and biomarker, efficacy and safety endpoints may be explored if data allows. The results of these modeling analyses may be reported separately from the clinical study report.

090177e19c2fb240\Approved\Approved On: 23-Dec-2022 10:25 (GMT)

090177e19c2fb240\Approved\Approved On: 23-Dec-2022 10:25 (GMT)

SCHEDULE OF ACTIVITIES

The schedule of activities tables (Table 1, Table 2, and Table 3) provide an overview of the protocol visits (including prescreening [optional], screening, and long term follow-up) and procedures. Refer to the STUDY PROCEDURES and ASSESSMENTS sections (Section 6 and Section 7 respectively) of the protocol for detailed information on each procedure and assessment required for compliance with the protocol.

The investigator may schedule unplanned visits, assessments or tests to conduct evaluations or assessments required to protect the well-being of the patient.

Table 1. Study Schedule of Activities: Prescreening (Optional) and Screening

**Prescreen Visit** is optional.<br>The purpose of this visit is to allow for blood/plasma testing (circulating tumor DNA or ctDNA), tumor biopsy testing (de novo, archival) or obtaining Sponsor pre-approval for historical FoundationOne® test results for eligibility prior to other screening activities.		
**General Activities**		**Comments**
Prescreening informed consent, SSID number	Prescreen (optional)	Prescreening activities may be performed after molecular prescreening consent is signed (before consent is signed for the screening period) for the sole purpose of submitting blood samples (liquid biopsy), and de novo or archival tumor tissue, or obtaining Sponsor pre-approval for use of prior FoundationOne® test result for eligibility.
Blood, de novo or archival tumor tissue for genomic testing or historical FoundationOne® results with Sponsor pre-approval	X	*Required for patients enrolling in Part 2 (optional for patients enrolling in Part 1):*<br>• Submit 20 mL of blood for DDR assessment. Blood sample for prospective DDR assessment is not required in China.<br>AND<br>• Tissue of tumor biopsy (de novo biopsy or archival tissue) for DDR assessment.<br>Biopsies of the brain, lung/mediastinum, pancreas, or endoscopic procedures extending beyond the esophagus, stomach, or bowel may not be performed for the sole purpose of determining study eligibility.<br>OR<br>• Historical FoundationOne® test results may be used for eligibility (after Sponsor pre-approval).

090177e19c2fb240\Approved\Approved On: 23-Dec-2022 10:25 (GMT)

Table 1. Study Schedule of Activities: Prescreening (Optional) and Screening

**Prescreen Visit** is optional.<br>The purpose of this visit is to allow for blood/plasma testing (circulating tumor DNA or ctDNA), tumor biopsy testing (de novo, archival) or obtaining Sponsor pre-approval for historical FoundationOne® test results for eligibility prior to other screening activities.		
**General Activities**		**Comments**
		Both blood and tumor tissue biopsy must be provided prior to randomization (it is not necessary to submit both at the prescreening visit). Results from either blood or tissue biopsy (or historical result for tissue biopsy) will be needed for eligibility and randomization (stratification). If results from liquid and tumor tissue sample are available prior to randomization and results are not concordant for the presence of the selected DDR mutation(s), positive DDR results will be used for eligibility and stratification regardless of the tissue used to generate the data. If either the blood or tissue sample is not provided during prescreening, the missing sample will have to be submitted prior to randomization (at screening) for retrospective concordance studies. In cases where historical FoundationOne® test results are approved by the Sponsor, tumor tissue is not required for the study, but may be requested by the Sponsor if available.
Demography	X	
Adverse Event review	X	For prescreening only: Record adverse events (AEs) and research-related injuries in the CRF from the time the blood sample was collected or de novo biopsy procedure was performed through and including 14 days. See Section 8.1.5.

**Screening Visit**		
**General Activities**	**-28 to -1 days**	**Comments**<br>Screening activities may be repeated any time as clinically indicated.
Informed consent, SSID number, contact cards.	Screening X	Obtain any time before any study-specific procedures. Ensure consent is on the current version of the form approved by the ethics committee and Sponsor. Consent can be obtained up to 42 days prior to randomization. All other screening tests and assessments (excluding CT/MRI and bone scans) must be done within 28 days prior to randomization. For information about the contact card please see section 4.5.
Blood, de novo or archival tumor tissue for genomic testing or historical FoundationOne® results with Sponsor pre-approval	X	ONLY IF NOT DONE DURING A PRE-SCREEN VISIT submit blood and tumor tissue samples. Blood sample for prospective DDR assessment is not required in China.<br>If either the blood or tumor tissue sample was not provided during prescreening, the missing sample will have to be submitted for retrospective concordance studies. In cases where historical FoundationOne® test results are approved by the Sponsor, tumor tissue is not required for the study, but may be requested by the Sponsor if available.

090177e19c2fb240\Approved\Approved On: 23-Dec-2022 10:25 (GMT)

**Screening Visit**		
Demographics	X	
Medical History	X	Includes histology, TNM classification and Gleason score assessment, and all other medical history.
Prior treatment for prostate cancer	X	
Eligibility criteria	X	
Enrollment /Randomization authorization form	X	Complete, sign, and fax or email the form with requested items to the sponsor or designee at least 2 business days before Day 1 for Part 1 or randomization for Part 2.
12-Lead electrocardiogram	X	Local (Note, ECG monitoring during the study for signs or symptoms of ischemic heart disease is to be per general clinical practice and local guidelines).
Vital signs, weight, height	X	Measure blood pressure, heart rate, temperature, weight, and height.
ECOG performance status	X	
Complete physical examination	X	Assess systems (eg, general appearance, head, eyes, ears, nose, mouth, skin, heart, lung, lymph nodes, gastrointestinal, genitourinary, neurologic, and skeletal).
Adverse events review	X	Report serious and non-serious adverse event information from time informed consent form is signed through screen failure or through a minimum of 28 days after the last dose of study treatment (talazoparib or talazoparib/placebo or enzalutamide, whichever is taken last). See Section 8.1.4 for additional details.
Prior and concomitant medications and treatments	X	
Contraception	X	The investigator is to assess if the patient is capable of ejaculating. If capable, instruct the patient to always use a condom to avoid partner/fetal exposure and instruct the patient not to donate sperm for 4 months after last dose of talazoparib. For these patients the investigator is to confirm correct contraception use or note changes in contraception use in the patient medical record as per Table 2. See Section 4.4.1. for contraception.
Brief Pain Inventory – Short Form	X	Administer question #3 of the questionnaire. Score on question 3 must be <4 to qualify for entry. (Referred to as "Modified Brief Pain Inventory – Full Form" in the CRF).
**Laboratory Evaluations**		
Hematology, serum chemistry, reticulocyte count, erythropoietin, serum folate and B12	X	Local laboratory test results will be used.
Testosterone, PSA	X	Local laboratory test results will be used.

090177e19c2fb240\Approved\Approved On: 23-Dec-2022 10:25 (GMT)

**Screening Visit**		
		Prostate specific antigen (PSA) progression defined by rising PSA of at least 2 consecutive rises in most recent PSA to be documented over a reference value (measure 1) taken at least 7 days apart within the last 12 months. If the third PSA measure is not greater than the second measure, a fourth PSA measure is required to be taken and be greater than the second measure. The third (or the fourth) confirmatory PSA should be taken within 4 weeks prior to randomization. The PSA value obtained before randomization (third or fourth measurement) must be ≥1 µg/L.
HIV (ELISA) in South Africa only	X	Optional HIV testing using the ELISA test may be conducted for subjects of unknown HIV status per investigator discretion. Subjects will be asked to sign an HIV consent form and be given pre and post test counseling at the site.
Part 2 only: Blood sample for CTC analysis	X	Collect 1 tube of 10 mL of blood. Please make sure no less than 10 ml are collected. These samples are sent to the central laboratory immediately upon collection at room temperature; see Section 7.5 and Central Laboratory Manual.
Part 2 only: Blood sample for ctDNA	X	Collect 20 mL of blood optimized for plasma preparation at indicated visits. These samples are sent to the central laboratory and results obtained will be used for exploratory analyses as defined in Section 7.5 and Central Laboratory Manual.
**Study Day**	**-42 to -1**	**Comments**
**Radiographic Assessments**		May use scans obtained as part of standard of care before consent was signed and within 42 days before Day 1 (randomization for Part 2) if scans were performed per the specific study requirements (per imaging manual).
CT scan of chest; CT or MRI of abdomen and pelvis	X	Computed tomography scan, magnetic resonance imaging.
Whole-body bone scan	X	

CRF = case report form; CT = computed tomography; CTCs = circulating tumor cells; ctDNA = circulating tumor deoxyribonucleic acid; ECG = electrocardiogram; ECOG = Eastern Cooperative Oncology Group; MRI = magnetic resonance imaging; PSA = prostate-specific antigen; SSID = single subject identification; TNM = Classification of Malignant Tumours.

090177e19c2fb240\Approved\Approved On: 23-Dec-2022 10:25 (GMT)

Table 2. Study Schedule of Activities: Treatment Period

Study Period or Visit			Treatment											Unsch[a]	Safety FU[b]
**Study Week**	**1**	**3**	**5**	**7**	**9**	**11**	**13**	**15**	**17**	**21**	**25**	**29, 33, 37, 41, 45, 49, 53[c]**	**37, 49, 61, 73, 85, 97, 109[y] +**	**Varies**	**Varies**
**Window (Days)[d]**	**na**	**±3 (±7 for scans)**												**na**	**±3**
Part 1 (Open-label treatment: Enrollment, number *Part 2 (Double-blind treatment)*: Randomization,[e] and randomization numbers	X														
**General Activities**															
Vital signs, height and weight[f]	X		X		X		X		X	X	X	X		X	X
Physical examination[g]	X		X		X		X		X	X	X	X		X	X
ECOG performance status	X		X		X		X		X	X	X	X		X	X
BPI-SF[h]	X		X		X		X		X	X	X	X			X
EORTC QLQ-C30[h]	X		X		X		X		X	X	X	X			X
EORTC QLQ-PR25[h]	X		X		X		X		X	X	X	X			X
EQ-5D-5L[h]	X		X		X		X		X	X	X	X			X
Pain Log[i]			X		X		X		X	X	X	X			X
Analgesic Log[j]			X		X		X		X	X	X	X			X
Symptomatic skeletal event evaluation[z]	X		X		X		X		X	X	X	X		X	X
Adverse events review[k]	X	X	X	X	X	X	X	X	X	X	X	X	X	X	X
Concomitant medications	X	X	X	X	X	X	X	X	X	X	X	X	X	X	X
ECG														X	X
Contraception Use[x]	X	X	X	X	X	X	X	X	X	X	X	X	X	X	X
Study treatment dispensing[l]	X[m]		X		X		X		X	X	X	X		X	
Study treatment accountability			X		X		X		X	X	X	X		X	
**Lab Evaluations**															
Hematology, reticulocyte count, erythropoietin, serum folate, B12 and serum chemistry[n]	X	X	X	X	X	X	X	X	X	X	X	X		X	X
Prostate-specific antigen[o]	X				X				X		X	X (q8weeks)		X	X
Blood sample for PK[p]	X (Part 1 only)	X	X		X		X		X					X	
Blood samples for CTC analysis[q]	X				X				X		X				X
Blood samples for ctDNA[r]	X				X				X		X				X

090177e19c2fb240\Approved\Approved On: 23-Dec-2022 10:25 (GMT)

Table 2. Study Schedule of Activities: Treatment Period

Study Period or Visit			Treatment											Unsch[a]	Safety FU[b]
**Study Week**	**1**	**3**	**5**	**7**	**9**	**11**	**13**	**15**	**17**	**21**	**25**	**29, 33, 37, 41, 45, 49, 53[c]**	**37, 49, 61, 73, 85, 97, 109[y] +**	**Varies**	**Varies**
**Window (Days)[d]**	**na**	**±3 (±7 for scans)**												**na**	**±3**
Blood samples for protein biomarkers[s]	X				X				X		X				X
Blood sample for banked biospecimen[t]	X														
Blood sample to be stored for reflex testing for HBV and HCV[u]	X														
Saliva sample for germline comparator[v]	X														
**Radiographic Assessments[w]**															
CT scan of chest					X				X		X		X	X[a]	
CT or MRI of abdomen and pelvis					X				X		X		X	X[a]	
Bone scan					X				X		X		X	X[a]	

Anti-HBc = hepatitis B core antibody; BICR = blinded independent central review; BPI-SF = Brief Pain Inventory Short Form; CRF = case report form; CT = computed tomography; CTCs = circulating tumor cell; ctDNA = circulating tumor DNA; DNA = deoxyribonucleic acid; ECG = electrocardiogram; ECOG = Eastern Cooperative Oncology Group; EORTC = European Organisation for Research and Treatment of Cancer; EQ-5D-5L = European Quality of Life 5-Domain 5-Level Scale; FFPE = formalin-fixed paraffin-embedded; FU = follow-up; HBsAg = hepatitis B surface antigen; HBV = hepatitis B virus; HCV = hepatitis C virus; lab = laboratory; MRI = magnetic resonance imaging; na = not applicable; PCWG = Prostate Cancer Working Group; PK = pharmacokinetics; PSA = prostate specific antigen; QLQ-C30 = Quality of Life Cancer Questionnaire; QLQ-PR25 = Quality of Life Questionnaire-Prostate 25; RECIST = Response Evaluation Criteria in Solid Tumors; RNA = ribonucleic acid; unsch = unscheduled.

a. Unscheduled assessments/visits may be done anytime necessary to assess or follow up adverse events (eg, labs or ECG), at the patient's request, or per investigator decision or due to missed assessments or sample not analyzed due to technical issues. Perform imaging if disease progression is suspected.

b. 28 days after permanent treatment discontinuation of all study treatments (talazoparib, talazoparib/placebo or enzalutamide) or before initiation of a new antineoplastic or investigational therapy, whichever occurs first. Final CTC, ctDNA and protein biomarker samples are to be collected. Phone patients for adverse event follow-up if they do not come to the clinic.

c. Visits are to be every 2 weeks through Week 17, every 4 weeks through Week 53 and following Week 53, visits repeat every 8 weeks while on study treatment – and are to continue every 8 weeks until study treatment is discontinued. Radiographic imaging is every 8 weeks through Week 25 of the study, then every 12 weeks thereafter – and are to continue every 12 weeks until study treatment is discontinued for radiographic progression. During this time visits must be within 60 days to allow for accurate drug dispensing (sites will dispense 2 bottles per visit).

d. Dispensing of study treatment should be taken into account when scheduling visits: visits up to Week 53 should occur within 30 days for drug dispensing (sites will dispense a 30 day drug supply).

e. Patients should start treatment within 3 days after randomization. Day 1 is the first day of dosing for Part 1 and the day of randomization for Part 2.

f. Measure blood pressure, heart rate, temperature, height and weight (Height is only applicable to patients in Part 2 and only required to be collected at Screening).

090177e19c2fb240\Approved\Approved On: 23-Dec-2022 10:25 (GMT)

Table 2. Study Schedule of Activities: Treatment Period

Study Period or Visit			Treatment											Unsch[a]	Safety FU[b]
Study Week	1	3	5	7	9	11	13	15	17	21	25	29, 33, 37, 41, 45, 49, 53[c]	37, 49, 61, 73, 85, 97, 109[y] +	Varies	Varies
Window (Days)[d]	na	±3 (±7 for scans)												na	±3

g. Assess systems (eg, general appearance, head, eyes, ears, nose, mouth, throat, skin, heart, lung, lymph nodes, gastrointestinal, genitourinary, neurologic, and skeletal) per standard of care at the study site or as clinically indicated by symptoms.

h. Part 2: Patients are to complete questionnaires before the first dose of study treatment on Day 1. At all subsequent visits, the patient is to complete the questionnaires before any other study activities. Questionnaires should be completed while alone in the same order at each visit. Perform post-baseline assessments every 4 weeks through Week 53, or radiographic progression, whichever is earlier; every 8 weeks after Week 53 until radiographic progression when no such progression had been previously documented; every 12 weeks after radiographic progression until end of study. In Part 1 the BPI-SF is completed per paper version.

i. Pain log is to be completed by patients in Part 2 for each of 7 consecutive days preceding scheduled site visits.

j. Analgesic log is to be completed by patients in Part 2 for each of 7 consecutive days preceding scheduled site visits.

k. Collect serious and non-serious adverse event information from the time of signed informed consent through a minimum of 28 days after the last dose of study treatment. Refer to Section 8.1.4 regarding the reporting timeframe for SAEs. Any diagnosis of myelodysplastic syndrome or acute myeloid leukemia must be reported as an SAE.

l. Administer talazoparib in the clinic on study days with PK assessments.

m. Instruct patient to self-administer study treatments.

n. Hematology, reticulocyte count, erythropoietin, serum folate, B12 and serum chemistry are to be evaluated locally per the visit schedule (every 2 weeks through Week 17, then every 4 weeks trough Week 53, and then every 8 weeks thereafter). Investigators may collect samples for laboratory assessment up to 3 days prior to a scheduled visit to guide decision making. Samples for PK, CTC, ctDNA, protein biomarkers, and a sample for reflex HBV and HCV testing are to be evaluated via the central laboratory. Refer to the central laboratory instruction manual for sample processing.

o. Collect samples for analysis of PSA every 8 weeks until determination of radiographic progression (local for Part 1 or by BICR for Part 2). An additional PSA is to be collected at Week 53 visit, then to continue every 8 weeks thereafter, thus aligning the PSA and site visits to be every 8 weeks.

p. *Treatment Part 1:* Collect blood samples for PK pre-dose, 1, 2, 4, 6 and 24 hours post-dose at Day 1 (Week 1) and Week 9. Collect blood samples for PK pre-dose and 2 hours post-dose at Week 5 and pre-dose at Week 13.
Treatment Part 2: Collect blood samples for PK pre-dose and 2 hours post-dose at Weeks 3, 5 and 9, and pre-dose at Weeks 13 and 17. *All patients*: Additional PK should be taken based on discretion of investigators, eg, adverse events. Record the dose amounts and time of dose administration on both the day of and the day before PK sampling. Record the date and time of each PK sample. Scheduled PK samples for enzalutamide should be collected if talazoparib or talazoparib/placebo is discontinued. Similarly, scheduled PK samples for talazoparib or talazoparib/placebo should be collected if enzalutamide is discontinued.

q. For CTC analysis, collect 1 tube of 10 mL of blood at Day 1 prior to dosing and 1 tube of 10 mL at all other visits per schedule. Please make sure no less than 10 ml are collected. These samples are sent to the central laboratory immediately upon collection at room temperature. Subjects who prove non-evaluable for CTC enumeration at baseline (both screening and Day 1 sample must be non-evaluable) may forego collection of additional CTC samples. See Section 7.5 and Central Laboratory Manual. For subjects enrolled in China: instructions for CTC samples are in Section 7.5.1.

r. For ctDNA collect 20 mL of blood optimized for plasma preparation at indicated visits. See Section 7.5 and Central Laboratory Manual. For subjects enrolled in China: instructions for the ctDNA sample are in Section 7.5.1.

090177e19c2fb240\Approved\Approved On: 23-Dec-2022 10:25 (GMT)

Table 2. Study Schedule of Activities: Treatment Period

Study Period or Visit			Treatment											Unsch[a]	Safety FU[b]
Study Week	1	3	5	7	9	11	13	15	17	21	25	29, 33, 37, 41, 45, 49, 53[c]	37, 49, 61, 73, 85, 97, 109[y] +	Varies	Varies
Window (Days)[d]	na	±3 (±7 for scans)												na	±3

s. For protein biomarkers collect 10 mL of whole blood for processing into serum at indicated visits. See Section 7.5 and Central Laboratory Manual. For subjects enrolled in China: instructions for the protein biomarker sample are in Section 7.5.1. Protein biomarkers sample will not be collected and analyzed in China.

t. Unless prohibited by local regulations or ethics committee decision, collect a blood sample (4 mL) optimized for DNA analysis before the first dose of study treatment. Retain for potential pharmacogenomic/biomarker analyses. Refer to Section 7.6 and Central Laboratory Manual. See Section 7.5 and Central Laboratory Manual. If not collected on the designated collection day, collect at the next available time point when biospecimens are being collected in conjunction with a patient visit. Blood sample for banked biospecimens will not be collected and analyzed in China.

u. Collect sample to be stored centrally for reflex testing for HBV (HBsAg, anti-HBc) and HCV (HCV Ab, reflex testing for HCV RNA if positive). Tests on this sample will be done if liver toxicities occur during treatment period.

v. Optional for Part 1; collect only if tumor tissue provided for the same patient.
Required for Part 2, unless prohibited by local regulations or ethics committee decision, collect a saliva sample optimized for DNA analysis before the first dose of study treatment. Refer to Section 7.5 and Central Laboratory Manual. For subjects enrolled in China: instructions for saliva sample are in Section 7.5.1. Depending on assay availability, blood may be substituted for saliva in China.

w. CT and MRI to be done for assessment of soft tissue disease only. Bone lesions will be evaluated only on bone scans. Radiographic imaging should be performed every 8 weeks through Week 25 and then every 12 weeks until radiographic progression is determined by investigator (Part 1) or by investigator and BICR (Part 2) regardless of initiation of subsequent antineoplastic therapy. RECIST 1.1 and PCWG3 assessments are to be completed when radiographic imaging tests are done and documented on the CRF. If radiographic progression is documented per BICR and investigator for Part 2 and investigator for Part 1 and the patient continues on study treatment as the patient is clinically benefitting per investigator, all radiographic imaging is to be continued per standard of care at the institution.

x. For patients capable of ejaculation, the investigator is to instruct the patient to always use a condom to avoid partner/fetal exposure and instruct the patient not to donate sperm for 4 months after last dose of talazoparib. Refer also to Section 4.4.1 for contraception language. Document in the patient medical records if the patient was informed on how to prevent partner/fetal exposure, not to donate sperm for 4 months after discontinuation of talazoparib, and if contraception instructions were provided.

y. For patients remaining on study treatment beyond radiographic progression per BICR and investigator for Part 2 and investigator for Part 1 (because in the opinion of the investigator the patient is still deriving benefit at this time), subsequent radiographic imaging should continue per standard of care at the institution.

z. Symptomatic skeletal event evaluation includes symptomatic fractures, surgery or radiotherapy to the bone and spinal cord compression. Non-symptomatic fractures will be also recorded in the CRF.[58]

090177e19c2fb240\Approved\Approved On: 23-Dec-2022 10:25 (GMT)

Table 3. Study Schedule of Activities: Long-term Follow-up

Study Period or Visit	Long-Term Follow-Up	Long-term follow-up begins after safety follow-up and may be conducted by telephone unless imaging is required.
**Window (Days)**	**±7**	Follow-up frequency is per the radiographic imaging schedule based on Day 1 and is every 8 weeks through week 25 of the study, then every 12 weeks thereafter until the patient dies or withdraws consent for follow-up, or the study is terminated by the Sponsor.
**General Activities**		
Radiographic imaging (Part 1 and 2) and PSA assessment (Part 2 only)	X	Continue radiographic imaging and PSA assessment (per imaging schedule) for patients who permanently discontinue study treatment for any reason other than radiographic progression determined by investigator for Part 1 or investigator and BICR for Part 2 regardless of the initiation of a new antineoplastic therapy, until radiographic progression is determined by investigator for Part 1 or investigator and BICR for Part 2, withdrawal of consent for follow-up, or death. RECIST 1.1 and PCWG3 assessments are to be done when radiographic assessment required and documented in the CRF. For patients on new anticancer therapy, scans should continue according to the schedule of assessments, or, if this is not possible, as per local SOC.
New antineoplastic therapy(ies)	X	Record all subsequent treatments for patients starting a new antineoplastic or investigational therapy.
Diagnosis of myelodysplastic syndrome or acute myeloid leukemia	X	Record any diagnosis of myelodysplastic syndrome or acute myeloid leukemia and report as an SAE. Provide tissue samples and other supporting data used to enable the diagnosis of myelodysplastic syndrome or acute myeloid leukemia for central review if requested and possible.
Survival status	X	Obtain survival status by any means including telephone, clinic visit, chart review, or by communicating with an individual (eg, family, friend, or referring health care provider) who is knowledgeable of the patient's survival status.
Part 2: BPI-SF, EORTC QLQ-C30 and QLQ-PR25, EQ-5D-5L, Pain Log, and Analgesic Log via electronic patient reported outcome device	X	Ask the patient to complete the questionnaires before any other study activities. Questionnaires should be completed independently and in the same order at each visit. Pain and analgesic assessments (via pain and analgesic logs, respectively) will be completed for 7 consecutive days before a study visit until study results are determined. During Long Term Follow up when the visits may be conducted by telephone (if imaging is not being performed due to radiographic progression being met) these assessments should also be completed remotely from home as per the long term visit schedule.
Symptomatic skeletal event assessment	X	Every effort should be taken to follow patients who have not had a symptomatic skeletal event for the onset of such an event.

090177e19c2fb240\Approved\Approved On: 23-Dec-2022 10:25 (GMT)

Table 3. Study Schedule of Activities: Long-term Follow-up

Study Period or Visit	Long-Term Follow-Up	
Window (Days)	±7	Long-term follow-up begins after safety follow-up and may be conducted by telephone unless imaging is required.<br>Follow-up frequency is per the radiographic imaging schedule based on Day 1 and is every 8 weeks through week 25 of the study, then every 12 weeks thereafter until the patient dies or withdraws consent for follow-up, or the study is terminated by the Sponsor.

BICR = blinded independent central review; BPI-SF = Brief Pain Inventory Short Form; CRF = case report form; EORTC = European Organisation for Research and Treatment of Cancer; EQ-5D-5L = European Quality of Life 5-Domain 5-Level Scale; Part 1 = open-label treatment; Part 2 = double-blind treatment; PCWG3 = Prostate Cancer Working Group; QLQ-C30 = Quality of Life Cancer Questionnaire; QLQ-PR25 = Quality of Life Questionnaire-Prostate 25; RECIST = Response Evaluation Criteria in Solid Tumors; SAE = serious adverse event.

1. INTRODUCTION

1.1. Overview and Mechanism of Action

1.1.1. Overview

Talazoparib (also known as PF-06944076, MDV3800) in combination with enzalutamide is being investigated for the treatment of metastatic castration-resistant prostate cancer (mCRPC) unselected for deoxyribonucleic acid (DNA) damage repair (DDR) deficiencies (referred to as "all-comers population"). Although patients will be unselected for DDR status, DDR deficiency assessment prior to randomization is required for stratification.

1.1.2. Mechanism of Action/Indication

Talazoparib is a potent, small molecule poly (adenosine diphosphate [ADP]-ribose) polymerase (PARP) inhibitor in development for the treatment of a variety of human cancers. Talazoparib exerts cytotoxic effects via 2 mechanisms: (1) inhibition of PARP1 and PARP2 catalytic activity, and (2) PARP trapping, a process in which PARP protein bound to a PARP inhibitor does not readily dissociate from DNA, thereby preventing DNA repair, replication, and transcription.[1]

Figure 1. Dual Cytotoxic Mechanisms of PARP Inhibitors

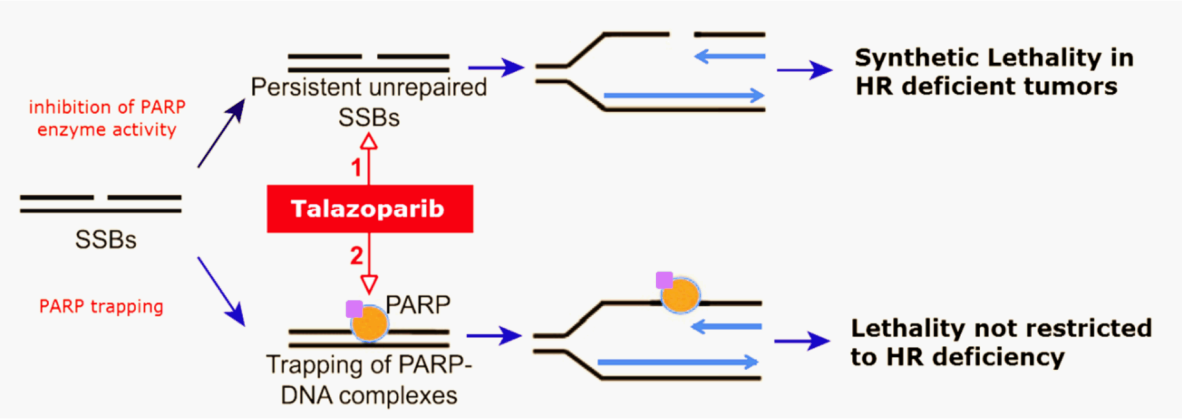

Source: Adapted from Reference 1

PARP = poly (adenosine diphosphate-ribose) polymerase
Inhibition of PARP catalytic activity (upper pathway) interferes with the repair of single-strand breaks, leading to replication fork damage that requires homologous recombination DNA repair for cell survival. Trapping of PARP–DNA complexes with PARP inhibitor (lower pathway; PARP inhibitor represented by ■) also leads to replication fork damage with more DNA repair processes required for cell survival.

Single-agent treatment with talazoparib has demonstrated potent antitumor effects in tissue culture studies, mouse tumor xenograft models, and in Phase 1 studies in patients with solid tumors. Talazoparib has also been shown to enhance the cytotoxic effects of DNA-damaging chemotherapy, including temozolomide and irinotecan, in both in vitro and in vivo preclinical models.

090177e19c2fb240\Approved\Approved On: 23-Dec-2022 10:25 (GMT)

1.2. Background and Rationale

1.2.1. Prostate Cancer

Prostate cancer is the second leading cause of cancer death in men. The American Cancer Society estimates that up to 161,360 men in the United States (US) were diagnosed with prostate cancer and approximately 26,730 will die of the disease in 2017.[2] In Europe in 2012, prostate cancer was the third most common cancer, with an estimated 416,700 new cases and 92,200 deaths.[3]

The androgen receptor (AR) signaling axis, the principal driver of prostate cancer growth, has been targeted by castration and other systemic therapies. However, a proportion of tumors progress despite castrate levels of testosterone, at which point the disease is considered castration-resistant. Castration-resistant prostate cancer represents a lethal transition in the progression of prostate cancer, with most patients ultimately succumbing to the disease.

Molecular profiling studies have revealed that the AR remains functional in a majority of progressing tumors.[4] Rationally designed therapies targeting the AR signaling pathway in the castrate setting include enzalutamide, a novel AR signaling inhibitor active in the presence of AR overexpression;[5] and abiraterone acetate/prednisone,[6,7] an inhibitor of 17,20-lyase (an androgen biosynthetic enzyme overexpressed in CRPC).[8,9] These therapies have conferred improved overall survival and radiographic progression-free survival (PFS) benefit compared with prednisone or placebo in patients with mCRPC, in both pre- and post-chemotherapy settings.[7,10,11,12] Recently, addition of abiraterone plus prednisone (or prednisolone) to androgen deprivation therapy (ADT) was also shown to improve overall survival and radiographic PFS in men with high-risk metastatic hormone-naïve prostate cancer (metastatic [M1] HNPC)[13,14] while enzalutamide has been shown superior to placebo in extending metastasis-free survival of patients with non-metastatic CRPC.[15]

The PROSPER study evaluated enzalutamide plus ADT versus ADT alone in 1,401 patients with non-metastatic CRPC. The study met its primary endpoint, demonstrating that the use of enzalutamide plus ADT significantly reduced the risk of developing metastasis or death compared to ADT alone. The median for the primary endpoint, metastasis-free survival (MFS), was 36.6 months for men who received XTANDI compared to 14.7 months with ADT alone (n=1401; HR=0.29 [95% CI: 0.24-0.35]; p<0.0001). As such, the Food and Drug Administration (FDA) has approved enzalutamide also for the treatment of patients with nonmetastatic castration-resistant prostate cancer (CRPC) in July 2018 based on the results of the study.

In the same setting, in February 2018, FDA approved apalutamide, next-generation nonsteroidal antiandrogen, for patients with non-metastatic castration-resistant prostate cancer (NM-CRPC). In SPARTAN study apalutamide significantly improved metastasis-free survival in patients with nonmetastatic castration-resistant prostate cancer. The estimated median MFS was 40.5 months for patients receiving apalutamide and 16.2 months for those receiving placebo (hazard ratio 0.28; 95% CI: 0.23, 0.35; p<0.0001).

090177e19c2fb240\Approved\Approved On: 23-Dec-2022 10:25 (GMT)

Taxane-based chemotherapy (docetaxel, cabazitaxel) has been shown to improve progression-free survival (PFS) and overall survival in patients with mCRPC.[16,17] At present, patients whose disease progresses on novel hormonal agents are recommended treatment with taxane-based chemotherapy, based on retrospective series supporting efficacy, although no randomized study data are available.[18,19] Recently, docetaxel-based chemotherapy also has been shown to provide significant benefit on overall survival when given in combination with androgen deprivation therapy in high-volume, metastatic or locally advanced, hormone-naïve disease;[20,21,22] such chemotherapeutic treatment earlier in the disease course may affect treatment decisions (such as re-treatment with docetaxel) once patients develop mCRPC. Additionally, many patients with advanced prostate cancer may not be candidates for taxane-based chemotherapy due to their comorbidities or performance status or may prefer to delay treatment with such chemotherapy as a last resort.

Other agents approved for treatment of mCRPC have more specific indications, including radium-223 for symptomatic osseous disease without known visceral disease[23] and sipuleucel-T for asymptomatic or minimally symptomatic mCRPC.[24] Thus, a significant unmet medical need remains for new therapeutic options for men with mCRPC.

1.2.2. PARP Inhibition

PARP1 and PARP2 play important roles in DNA repair.[25,26] Following DNA damage, PARP1 and PARP2 bind to single-stranded DNA breaks, cleave nicotinamide adenine dinucleotide, and attach multiple ADP-ribose units to the target protein, including itself.[27-30] The outcome is a highly negatively charged protein, which leads to the unwinding of the DNA strands and recruitment of proteins to repair the damaged DNA through the base-excision repair process. When PARP1 and PARP2 are inhibited, single-strand DNA breaks persist, resulting in stalled replication forks and conversion of single-strand breaks into double-strand breaks. These breaks must be repaired by homologous recombination or nonhomologous end joining or they may become lethal. Thus, inhibition of PARP catalytic activity results in synthetic lethality as defects in homologous recombination DNA repair prevent double strand breaks from being repaired, thereby killing the cell, including cancer cells.

In addition, PARP inhibitors bind to PARP-DNA complexes (ie, become trapped), thereby inhibiting DNA repair, replication, and transcription, which is cytotoxic to cancer cells. Although other PARP inhibitors possess both activities (ie, inhibition of PARP catalytic activity and PARP trapping), in vitro studies demonstrated that talazoparib is a more potent PARP trapper than other PARP inhibitors in clinical development, a property that has been associated with significant cytotoxicity in preclinical models.[30,31,32]

1.2.3. PARP Inhibitors as a Potential Targeted Therapy in mCRPC, Including in Androgen Pathway Manipulation

Proof of concept for the efficacy of PARP inhibition in prostate cancer with DDR deficiency was established in a Phase 2 study (TOPARP-A) with the PARP inhibitor olaparib.[33] In the TOPARP-A study, 50 patients with mCRPC were treated with the monotherapy PARP inhibitor olaparib (400 mg orally twice daily). All had been previously treated with docetaxel, 98% with abiraterone or enzalutamide, and 58% with cabazitaxel. Eligible

090177e19c2fb240\Approved\Approved On: 23-Dec-2022 10:25 (GMT)

patients had an Eastern Cooperative Oncology Group (ECOG) score of 0 to 2; disease progression per Prostate Cancer Working Group 2 (PCWG2) and/or Response Evaluation Criteria in Solid Tumors, version 1.1 (RECIST 1.1); 5 or more circulating tumor cells (CTCs) per 7.5 mL of blood; and consented to a fresh tumor biopsy. No prior exposure to platinum, cyclophosphamide, mitoxantrone, or other PARP inhibitors was permitted. Forty-nine patients were evaluable for response based on a composite endpoint of objective response according to RECIST 1.1, a reduction of at least 50% in prostate-specific antigen (PSA), or a confirmed reduction in CTC counts from 5 or more cells per 7.5 mL of blood to less than 5 cells per 7.5 mL of blood in patients with an identified homozygous deletion and/or putative deleterious mutation in DNA repair genes. Sixteen of 49 evaluable patients (33%) had a response and the median duration of treatment was 40 weeks for responding patients. Treatment with olaparib led to a response based on the composite endpoint definition in 14 of 16 patients (88%) with identified genomic deficiencies associated with DDR. On the other hand, only 2 responses (6%) were seen in 33 patients without such genomic deficiencies.

Of the 32 evaluable patients (65% of the 49 evaluable patients) who had measurable disease at baseline per RECIST 1.1, 7 patients had DDR deficiencies and 5 of those 7 (71%) had a partial response (PR) per RECIST 1.1. Patients with DDR deficiencies had longer median rPFS (9.8 vs 2.7 months) and median overall survival (13.8 vs 7.5 months) compared with patients without DDR deficiencies.

In an integrated analysis of somatic and germline mutation status in tumor biopsies from 150 patients with mCRPC, 22.7% of cases had mutations in DNA repair genes associated with sensitivity to a PARP inhibitor, including BRCA2, BRCA1, CDK12, FANCA, RAD51B, RAD51C, MLH1, MSH2, and ATM.[4] Loss of BRCA2 was observed in 12.7% of cases (approximately 90% of these were biallelic loss), and 8% harbored a germline alteration. Another clinical targeted sequencing analysis of prostate cancer identified similar frequencies of somatic and germline alterations in DDR genes BRCA2, BRCA1, and ATM.[34]

There is a three-fold rationale for combining PARP inhibitors with androgen-receptor (AR) signaling inhibitors in CRPC regardless of DDR mutational status, based on research on nonclinical models and clinical samples: First, AR signaling inhibition suppresses the expression of homologous recombination repair genes including BRCA1, resulting in "BRCAness" and sensitivity to PARPi.[43,44] Second, PARP1 activity has been shown to be required for maximal AR function[46] and thus inhibiting PARP is expected to reduce AR signaling and increase sensitivity to NHT. Third, clinical resistance to AR blockade is sometimes associated with co-deletion of RB and BRCA2, which is in turn associated with sensitivity to PARP inhibition sensitivity.[45]

In a recent double-blind, randomized, placebo-controlled Phase 2 study, 142 patients with mCRPC who had previously received docetaxel were randomly assigned to receive olaparib plus abiraterone (n=71) or placebo and abiraterone (n=71). The objective of the study was to assess efficacy of olaparib plus abiraterone in patients regardless of homologous recombination DDR mutations. The primary endpoint was rPFS, with secondary endpoints of safety and tolerability, time to second progression, overall survival, time to first

090177e19c2fb240\Approved\Approved On: 23-Dec-2022 10:25 (GMT)

subsequent anticancer therapy, time to second subsequent anticancer therapy, and change in circulating tumor cell count. Olaparib plus abiraterone provided a significant rPFS benefit to mCRPC patients compared with abiraterone alone; median rPFS was 13.8 months (95% CI 10.8–20.4) with olaparib and abiraterone and 8.2 months (5.5–9.7) with placebo and abiraterone (hazard ratio [HR] 0.65, 95% CI 0.44–0.97, p=0.034).

A prespecified subgroup analyses of rPFS by DDR mutation status revealed that within the cohort of patients with DDR mutation rPFS was 17.8 months (95% CI 2.9–27.6) in the olaparib group compared with 6.5 months (2.7–not reached) in the placebo group (N=21 in patients with DDR mutation [HR 0.74; 95% CI 0.26, 2.12]). Among the DDR wild-type subgroup the median rPFS of 15.0 months (95% CI 5.4–not reached) in the olaparib group versus 9.7 months (2.9–17.5) in the placebo group (N=35 in wild-type [HR 0.52; 95% CI 0.24, 1.15]). Finally median rPFS median rPFS was 13.1 months (95% CI 8.1–22.4) in the olaparib group versus 6.4 months in subgroup of patients whose DDR status was reported to be unknown. (N=142 in ITT [HR 0.65; 95% CI 0.44, 0.97]). Overall, the efficacy results of this study indicated that the pharmacological alteration of AR signaling in combination with the administration of PARP inhibitor prolonged clinical benefit in patients with mCRPC, regardless of DDR mutation status. As expected more adverse events were reported with olaparib plus abiraterone than abiraterone alone with nausea (37% vs 18% respectively), anemia (21% vs none respectively), pneumonia (6% vs 4% respectively), and myocardial infraction (6% vs none respectively) being the most frequent. More serious adverse events were reported with olaparib plus abiraterone (34%) than abiraterone alone (18%). There was 1 treatment-related death that occurred in the olaparib plus abiraterone group.[35] The results of this study, coupled with the pre-clinical rationale detailed above, provides support for the inclusion of patients without DDR deficiencies or with unknown DDR mutational status in addition to those with DDR deficiencies to allow for evaluation of rPFS in mCRPC patient treated with talazoparib plus enzalutamide compared to placebo plus enzalutamide irrespective of DDR status as a primary objective.

1.3. Summary of Relevant Clinical Experience with Talazoparib

The clinical data presented here are as of 31 Jan 2018, unless otherwise stated. Approximately 659 patients and 18 healthy volunteers have received talazoparib at doses up to 2 mg/day in company sponsored clinical studies in hematologic malignancies and solid tumors. Aggregate safety data from 5 open label studies, including 1 randomized, company sponsored clinical study (PRP-001, 673 201, 673-301, MDV3800-13, and MDV3800 14; N=502 patients) evaluating talazoparib monotherapy at the proposed dose of 1 mg/day (Talazoparib 1 mg/day Population) provide the basis for the reported treatment emergent adverse events (TEAEs).

Results from these company-sponsored studies are summarized in the following sections.

090177e19c2fb240\Approved\Approved On: 23-Dec-2022 10:25 (GMT)

1.3.1. Efficacy

A phase 1 clinical study supports the efficacy of talazoparib monotherapy at 1 mg once daily.

PRP-001 is a Phase 1, open-label, safety, pharmacokinetics (PK), and dose-escalation (0.025-1.1 mg/day) and expansion (1 mg/day) study of talazoparib monotherapy in 110 patients with advanced or recurrent solid tumors with DNA repair deficiencies. As of the data cutoff date of 30 Nov 2015, objective responses (CR or PR) per RECIST 1.1 were observed in 8 of 18 patients (44%) with breast cancer and 12 of 25 patients (48%) with ovarian/primary peritoneal cancer with deleterious germline BRCA mutations; clinical benefit (CR, PR, or stable disease ≥24 weeks) was observed in 13 of 18 patients (72%) and 19 of 25 patients (76%), respectively. Seven of 12 patients with confirmed objective responses were treated with talazoparib at 0.1 to 0.9 mg/day, and 5 patients were treated at the recommended dose of 1 mg/day.

Study 673-201 (ABRAZO) is a Phase 2, 2-stage, 2-cohort study of talazoparib in patients with germline BRCA mutation and locally advanced and/or metastatic breast cancer who are either platinum responders (Cohort 1) or had at least 3 prior chemotherapy regimens with no platinum for metastatic disease (Cohort 2). As of December 2016, the confirmed objective response rate (CRs and PRs) was 20.8% for Cohort 1 (95% confidence interval [CI]: 10.47, 34.99), including 2 CRs (4.2%), and 37.1% for Cohort 2 (95% CI: 21.47, 55.08) per independent central radiology assessment. The objective response rate across both cohorts was 27.7% (95% CI: 18.45, 38.62), demonstrating clinically meaningful responses in this population with a poor prognosis and a high unmet medical need.

In addition, talazoparib significantly extended progression-free survival in the Phase 3 Study 673-301 (EMBRACA). The EMBRACA study, which was a global, open-label, randomized, parallel, 2-arm study of talazoparib versus protocol-specific physician's choice of standard single-agent chemotherapy (PCT [capecitabine, eribulin, gemcitabine or vinorelbine]) in gBRCA+ patients who may have received up to three prior cytotoxic chemotherapy regimens for locally advanced and/or metastatic breast cancer. Patients enrolled had a diagnosis of triple-negative breast cancer (TNBC) or HR+/HER2-negative breast cancer. The study randomized (2:1) 431 patients to receive talazoparib (1.0 mg) once daily or PCT.

The primary objective was progression-free survival, assessed by blinded independent central review, and secondary objectives were overall survival, overall response rate and clinical benefit rate at 24 weeks, and safety. Patient-reported outcomes were also measured.

Median PFS was 8.6 months (95% CI: 7.2, 9.3) for patients treated with talazoparib and 5.6 months (95% CI: 4.2, 6.7) for those treated with chemotherapy [HR: 0.54 (95% CI: 0.41, 0.71), p<0.0001]. This represents a 46% reduction in the risk of disease progression. In addition, the proportion of patients achieving a complete or partial response (objective response rate) in the talazoparib group was more than twice that of the control arm (62.6% for talazoparib vs. 27.2% for chemotherapy [OR: 4.99 (95% CI: 2.9-8.8), p<0.0001]). In addition PFS benefit consistent across metastatic BRCA-positive patients, including those with hormone receptor-positive and triple negative disease.[49] Finally patients who received talazoparib reported better global health status across all functional domains, including global

090177e19c2fb240\Approved\Approved On: 23-Dec-2022 10:25 (GMT)

health status (mean change from baseline 3.0 in the talazoparib group vs –5.4 in the standard chemotherapy group, p<0.001). Talazoparib is approved in the U.S. for the treatment of adult patients with deleterious or suspected deleterious germline breast cancer susceptibility gene (g*BRCA*)-mutated human epidermal growth factor receptor 2-negative (HER2-), locally advanced or metastatic breast cancer. Select patients for therapy based on an FDA-approved companion diagnostic for talazoparib. In addition, on April 26, 2019 European Medicines Agency (EMA) also adopted a positive opinion recommending talazoparib for marketing authorization in the European Union (EU).

1.3.2. Safety

This is the first study evaluating the efficacy and safety of talazoparib and enzalutamide when administered in combination.

TEAEs in ≥20% of the 502 patients in the talazoparib 1 mg/day population were related to myelosuppression (anemia, neutropenia), gastrointestinal (GI) toxicity (nausea, diarrhea, vomiting, constipation, and decreased appetite), fatigue, headache, and alopecia. Grade 3 or 4 TEAEs in ≥5% of patients were associated with myelosuppression.

Study treatment related TEAEs occurring in ≥20% of patients in the Talazoparib 1 mg/day population were anemia (45.8%), fatigue (36.1%), nausea (32.5%), neutropenia (21.9%), and alopecia (20.1%). Grade 3 or 4 drug related TEAEs occurring in ≥5% of patients were anemia (34.1%), neutropenia (13.9%), thrombocytopenia (10.6%), and platelet count decreased (5.4%).

A total of 23 of 502 patients in the Talazoparib 1 mg/day population had a TEAE that led to death (8 associated with malignancies including 1 also associated with pneumonia; 2 dyspnea; 2 general physical health deterioration; 3 disease progression; and 1 each lung infection, cerebral hemorrhage, cerebrovascular accident, fatigue (after the data cutoff date of 31 January 2018, the adverse event of Grade 5 fatigue was changed to Grade 5 failure to thrive), liver disorder, neurological symptom, respiratory failure, and veno-occlusive liver disease). Of these, only veno-occlusive liver disease (reported in 1 patient in the Phase 3 Study 673-301) was assessed as related to study treatment by the investigator.

Serious adverse events (SAEs) occurred in 164 of 502 patients (32.7%) in the talazoparib 1 mg/day population. SAEs occurring in ≥2% of patients were anemia (5.2%) and dyspnea and pleural effusion (2.2% each). Forty-seven (47) patients had SAEs considered related to study treatment. Study treatment-related SAEs occurring in ≥1% of patients were anemia (4.6%), thrombocytopenia (1.2%), and platelet count decreased (1.2%).

Twenty (20) of 502 patients (4.0%) in the talazoparib 1 mg/day population discontinued study treatment due to a TEAE. The events that led to study treatment discontinuation were anemia (3 patients), increased alanine aminotransferase (ALT, 2 patients), and accidental overdose, increased aspartate aminotransferase (AST), bradycardia, metastatic breast cancer, cerebral hemorrhage, dyspnea, glioblastoma multiforme, headache, metastases to meninges, muscular weakness, neutropenia, obstructive airways disorder, thrombocytopenia, transient ischemic attack, and vomiting (1 patient each).

090177e19c2fb240\Approved\Approved On: 23-Dec-2022 10:25 (GMT)

Among the 502 patients in the talazoparib 1 mg/day population, 63.9% had a TEAE that led to dose reduction and 61.2% had a TEAE that led to dosing interruption. The most common TEAEs that led to dose reduction or interruption were associated with myelosuppression.

In the EMBRACA study the adverse events observed with talazoparib were consistent with findings from previous studies. The most common AEs observed with talazoparib (any grade in at least 15% of patients) were anemia (52.8%), fatigue (50.3%), nausea (48.6%), neutropenia (34.6%), headache (32.5%), thrombocytopenia (26.9%), alopecia (25.2%), vomiting (24.8%), diarrhea (22%), constipation (22%), decreased appetite (21.3%), back pain (21%) and dyspnea (17.5%). The incidence of serious AEs was 31.8% in the talazoparib arm and 29.4% in the PCT arm. Discontinuations due to AEs occurred in 7.7% of patients in the talazoparib arm and 9.5% of patients in the PCT arm.

Recent results from an international, Phase 2, open-label, response rate study of talazoparib in DDR-deficient mCRPC patients (TALAPRO-1) demonstrated that talazoparib 1 mg/day was generally tolerated and TEAEs were manageable through dosing interruption, dose reduction, and/or standard supportive care. The safety profile to date in the TALAPRO-1 study was consistent with the known safety profile of talazoparib. No new safety signals were identified.

Additional safety information from other talazoparib studies may be found in the current talazoparib Investigator's Brochure.

1.3.3. Pharmacokinetics and Metabolism

1.3.3.1. Talazoparib

Talazoparib plasma exposure was dose proportional in the dose range of 0.025 mg to 2 mg once a day suggesting linear PK. Talazoparib absolute bioavailability is at least 54.6% based on excretion of unchanged talazoparib in urine. After administration of a single 1 mg dose of talazoparib to cancer patients, the median T_{max} ranged from 0.5 to 2.0 hours across studies. Administration of talazoparib with food (a high-fat, high-calorie meal) had no impact on the AUC while reduced the C_{max} by 46%. The reduction in the rate of absorption with food is not expected to be clinically relevant as efficacy is generally driven by total exposure. Therefore, talazoparib can be taken with or without food.

Mean talazoparib binding to human plasma proteins is 74%. Population PK analysis showed that talazoparib apparent steady-state volume of distribution (Vss/F) was 420 L, which is significantly greater than total body water (42 L), indicating that talazoparib extensively distributes to peripheral tissues.

Talazoparib undergoes minimal hepatic metabolism. Based on population PK analysis, there was no effect of mild hepatic impairment (total bilirubin ≤ upper limit of normal [ULN] and AST > ULN, or total bilirubin >1.0 to 1.5 ULN and any AST) on talazoparib exposure. No dose adjustment is necessary for patients with mild hepatic impairment.

090177e19c2fb240\Approved\Approved On: 23-Dec-2022 10:25 (GMT)

Talazoparib was eliminated slowly with a mean terminal plasma half-life (t½) of 89.8 hours. Talazoparib accumulated after 1 mg once a day dosing with a median accumulation ratio ranging from 2.33 to 5.15, consistent with its t½. Population PK analysis showed that talazoparib apparent oral clearance (CL/F) was 6.45 L/hr. Excretion of unchanged talazoparib in urine was the major route of elimination accounting for 54.6% of the administered dose. Population PK analysis showed that talazoparib CL/F was reduced by 14.4% and 37.1% in patients with mild renal impairment (creatinine clearance [CrCl], 60 to 89 mL/min) and moderate renal impairment (30 mL/min ≥ CrCl <60 mL/min), respectively, compared to that of patients with normal renal function (CrCl ≥90 mL/min). No dose adjustment is recommended for patients with mild renal impairment. For patients with moderate renal impairment, the recommended dose of talazoparib in combination with enzalutamide is 0.35 mg once a day.

In vitro studies showed that talazoparib is a substrate for the efflux transporters P-glycoprotein (P-gp) and breast cancer resistance protein (BCRP). Population PK analysis indicated that concomitant administration of strong P-gp inhibitors with talazoparib increased talazoparib exposure by 44.7% relative to talazoparib administered alone. Guidelines for concomitant use of talazoparib with P-gp inhibitors or inducers and BCRP inhibitors are provided in Section 5.9.

1.3.3.2. Enzalutamide

The pharmacokinetics of enzalutamide and its major active metabolite (N-desmethyl enzalutamide) were evaluated in patients with mCRPC and healthy male volunteers. Peak enzalutamide concentrations were generally achieved 0.5 to 3 hours after oral administration. With daily administration, enzalutamide steady state is achieved by Day 28 consistent with a mean terminal half-life of 5.8 days. The mean terminal half-life for N-desmethyl enzalutamide is approximately 7.8 to 8.6 days.

Administration of enzalutamide with a high-fat meal in healthy volunteers did not alter the AUC for enzalutamide or N-desmethyl enzalutamide compared to administration in fasted condition. Therefore, enzalutamide can be administered without regard to food.

No significant difference in enzalutamide clearance was observed in patients with mild to moderate renal impairment compared to patients with baseline normal renal function (creatinine clearance (CrCL) ≥90 mL/min). Therefore, no dose adjustment is necessary for patients with mild to moderate renal impairment. Exposure of enzalutamide and its N-desmethyl metabolite was similar in subjects with mild or moderate hepatic impairment as compared to subjects with normal hepatic function. Therefore, no enzalutamide dose adjustment is necessary for patients with baseline mild or moderate hepatic impairment.

In vitro, enzalutamide, N-desmethyl enzalutamide, and the major inactive carboxylic acid metabolite are not substrates for human P-gp. In vitro, enzalutamide and N-desmethyl enzalutamide exhibited both induction and inhibition properties of human P-gp [XTANDI® US Package Insert (USPI)]. Furthermore, in vitro studies showed that enzalutamide is an inhibitor of BCRP. However, the in vivo effect of enzalutamide on exposure of P-gp and

090177e19c2fb240\Approved\Approved On: 23-Dec-2022 10:25 (GMT)

BCRP substrates, such as talazoparib, has not been studied prior to the initiation of Part 1 of the current study.

1.3.3.3. Drug Drug Interactions between Talazoparib and Enzalutamide

Enzalutamide is primarily eliminated by hepatic metabolism mainly by cytochrome P450 (CYP450) enzymes CYP2C8 and CYP3A4. At the therapeutic dose of 1 mg/day, talazoparib did not markedly induce or inhibit CYP450 enzymes or transporters and is therefore unlikely to demonstrate clinically significant drug drug interactions or drug transporter inhibition when co-administered with corresponding substrates. Preliminary enzalutamide and N-desmethyl enzalutamide Ctrough values from Part 1 were generally consistent with published enzalutamide monotherapy Ctrough indicating no apparent effect of talazoparib on enzalutamide exposure.[50]

Talazoparib is a substrate of P-gp and BCRP, and plasma talazoparib concentrations may increase when co administered with P-gp or BCRP inhibitors (Section 1.3.3.1). In vitro data show that enzalutamide and its N-desmethyl metabolite are potential inhibitors and inducers, but not substrates, of the efflux transporter P-gp. The in vivo effect of enzalutamide on exposure of P-gp substrates, such as talazoparib, has not been studied prior to the initiation of Part 1 of the current study (Xtandi® Summary of Product Characteristics). Part 1 safety and PK data showed higher than expected frequency of Grade 3/4 hematological AEs and a ~2-fold higher than expected talazoparib C_{trough} at Week 5 when talazoparib 1 mg once daily was administered in combination with enzalutamide. The increase in talazoparib exposure might be due to inhibition of P-gp and/or BCRP by enzalutamide and its N-desmethyl metabolite. Reducing talazoparib dose to 0.5 mg once daily in combination with enzalutamide is expected to account for the observed interaction and maintain similar talazoparib exposure to that achieved with 1 mg once daily monotherapy with an acceptable safety profile (Section 1.6.1).

Based on existing clinical experience with enzalutamide from AFFIRM, PREVAIL, and PROSPER study as well as clinical experience with talazoparib monotherapy data, fatigue and gastrointestinal signs symptoms and hematological AEs may be reasonably expected with combination therapy. Additional information for talazoparib may be found in the Investigator's Brochure, which is the single reference safety document (SRSD) for talazoparib. The SRSD for enzalutamide is the Summary of Product Characteristics (SmPC), which is available in the European public assessment reports for Xtandi® (enzalutamide).

1.4. Talazoparib Benefits and Risks Assessment

The doses of talazoparib in this protocol are supported by nonclinical studies, Phase 1-3 studies in patients with advanced malignancies, and studies in patients with germline BRCA-mutated HER2-negative locally advanced or metastatic breast cancer. The expected adverse events with talazoparib include myelosuppression, gastrointestinal toxicity, fatigue, and alopecia. Myelodysplastic syndrome (MDS), acute myeloid leukemia (AML), hepatotoxicity, second primary non hematologic malignancies and pneumonitis are adverse events of special interest per the Investigator's Brochure. The activity of talazoparib as monotherapy and in combination with other agents is being evaluated in multiple indications.

090177e19c2fb240\Approved\Approved On: 23-Dec-2022 10:25 (GMT)

1.5. Enzalutamide Benefits and Risks Assessments

The tolerability, pharmacological property, and efficacy of enzalutamide were first investigated in a multicenter, open-label, dose-escalation Phase I/II study. A total of 140 men with CRPC were enrolled in the study, the majority with bone metastasis (78%) and half of whom had previously received chemotherapy (54%). The oral daily doses of enzalutamide ranged from 30 to 600 mg depending on the dosing cohort until the occurrence of disease progression or intolerable adverse events.

Fatigue was the most common dose-limiting toxicity, occurring at a Grade 3 or greater (Grade 3/4) severity in 11% and the maximum tolerated dose for sustained treatment >28 days was established as 240 mg. Subsequent studies in both the post- and pre-chemotherapy settings used 160 mg enzalutamide once daily as the recommended dose for further development of enzalutamide to be tested.

Enzalutamide received initial FDA approval on August 31, 2012, for the treatment of patients with mCRPC who have previously received docetaxel based on the results of the AFFIRM study, an international, Phase 3, randomized, double blind, placebo-controlled study of enzalutamide in CRPC patients previously treated with one or two chemotherapy regimens, at least one of which contained docetaxel.[51] The AFFIRM study enrolled 1199 patients (800 in the enzalutamide arm and 399 in the placebo arm) with a primary end point of overall survival (OS). Secondary end points included measures of response (PSA-level response, objective soft tissue response according to RECISTv1.1, and quality of life [QoL] score responses) and measures of progression (time to PSA progression [TTPP], radiographic progression-free survival [rPFS], and time to the first skeletal related event [SRE]). At the time of the prespecified interim analysis, enzalutamide significantly prolonged OS compared to placebo (HR 0.63; 95% CI 0.53-0.75; P <0.001) which indicated a reduced risk of death of 37% (median rPFS of 18.4 vs. 13.6 months).

PREVAIL was a Phase III study comparing enzalutamide activity with placebo in asymptomatic or minimally symptomatic chemotherapy-naive mCRPC patient. The co-primary end points of the study were rPFS and OS.[11] The results revealed that enzalutamide treatment significantly improved OS compared to placebo (HR 0.71; 95% CI 0.60–0.84; P <0.001) corresponding to a 29% decrease in the risk of death (median OS of 32.4 months vs. 30.2 months). The OS benefit of enzalutamide was consistent across all prespecified subgroups including elderly patients, visceral metastatic diseases, and anemic patients at baseline.

The FDA recently (July 2018) approved enzalutamide for the treatment of patients with nonmetastatic castration-resistant prostate cancer (CRPC). The approval is based on the Phase III PROSPER study, in which the combination of enzalutamide and androgen deprivation therapy (ADT) reduced the risk of metastases or death by 71% compared with ADT alone for patients with non-metastatic CRPC (HR 0.29; 95% CI, 0.24-0.35; P ,0.001). In the double-blind study, the median metastasis-free survival (MFS) was 36.6 months with enzalutamide plus ADT versus 14.7 months with ADT alone.

090177e19c2fb240\Approved\Approved On: 23-Dec-2022 10:25 (GMT)

The Grade 3/4 adverse events (AEs) that occurred in the enzalutamide and placebo arms were 45.3 and 53.1%, respectively. Specifically the more common AEs with enzalutamide (all Grades) were fatigue (34 vs. 29% in the placebo arm), diarrhea (21 vs. 18%), hot flush (20 vs. 10%), musculoskeletal pain (14 vs. 10%), and headache (12 vs. 6%). Grade 3 or greater side effects of interest were fatigue (6.3% in the MDV3100 group versus 7.3% in the placebo group), cardiac disorders (0.9% versus 2.0%) including myocardial infarction (0.3% versus 0.5%), seizure (0.6% versus 0.0%) and liver function test abnormalities (0.4% versus 0.8%). On the other hand the discontinuations due to AEs were reported in 8% of the patients in the enzalutamide arm and in 10% of the patients in the placebo arm. Some of the patients who manifested a seizure had potentially predisposing factors, including brain metastasis, lidocaine injection, and brain atrophy.

In the subsequent PREVAIL study, the safety profile was generally consistent with the previous AFFIRM results. More Grade 3/4 AEs were reported in the enzalutamide arm than in the placebo arm (43 vs. 37%). Seizure occurred in only 1 patient (0.1%) who had a history of seizure in the enzalutamide arm. Hypertension was more commonly observed in the enzalutamide arm than in the placebo arm (13 vs. 4%) all Grades. A similar proportion of patients in each arm discontinued the treatment due to AE (6%) and notably in contrast to the other antiandrogens, enzalutamide was not associated with hepatotoxicity.

Also in PROSPER study the adverse events were generally consistent with those reported in prior enzalutamide clinical studies in patients with mCRPC.[52] Grade 3 or higher adverse events were reported in 31% of men treated with Enzalutamide plus ADT and in 23% of men treated with ADT alone. The most common (≥2%) Grade 3 or higher adverse events that were reported more often in enzalutamide plus ADT-treated patients included hypertension (5% vs. 2%) and fatigue (3% vs. 1%).1%). 5% of patients who received enzalutamide plus ADT and 3% with ADT alone had major adverse cardiovascular events. Three seizures (<1%) were reported with enzalutamide plus ADT patients and none were reported for those who received ADT alone. The percentage of patients in whom adverse events were the primary reason leading to treatment discontinuation was low in both study arms (9% with enzalutamide plus ADT versus 6% with ADT alone).

In the combined Phase-3 studies, the incidence of ischemic heart disease events was higher in the enzalutamide group compared with the placebo group (2.6% vs 1.3%). Grade ≥3 events of ischemic heart disease occurred in 1.5% of patients in the enzalutamide group compared with 0.7% in the placebo group. Events of ischemic heart disease that led to death were reported in 0.4% of patients in the enzalutamide group compared with 0.1% in the placebo group.

The incidence of TEAEs of any event of fracture in the combined Phase 3 studies was higher in the enzalutamide group compared with placebo (10.2% vs 4.4%). When adjusted for differences in treatment duration, the event rates per 100 patient-years of treatment were 8.1 vs 4.8. The most common types of fracture reported among the enzalutamide group of the combined Phase 3 studies were rib fracture (2.4%) followed by spinal compression fracture (1.2%), femur fracture (0.4%) and upper limb fracture (0.3%). However the incidence of SAEs of fractures was low in the enzalutamide group of the combined Phase 3

090177e19c2fb240\Approved\Approved On: 23-Dec-2022 10:25 (GMT)

studies (2.9%) as were Grade ≥3 events of fracture (2.6%) and any event of fracture as the primary reason for discontinuation (0.1%). Finally the incidence of fractures increased with the length of treatment duration. The incidence of any event of fracture during the first 30 days of treatment in the combined Phase 3 studies was 0.5% for both the enzalutamide and placebo groups. During the first 180 days of treatment, the incidence of fractures was slightly higher in the enzalutamide group compared with the placebo group (3.1% vs 2.6%).

For the others TEAEs of interest in the combined Phase 3 Studies please refer to the Enzalutamide SmPC.

1.6. Rationale for Combination Treatment

Prior to the recent approval of novel hormonal therapies (NHTs) (enzalutamide, abiraterone acetate/prednisone), the only approved therapies for mCRPC were docetaxel, cabazitaxel, and (for asymptomatic or minimally symptomatic mCRPC), sipuleucel-T.[24] The approval of NHTs in mCRPC patients previously treated with docetaxel[7,12] represented a therapeutic advance for these patients, followed shortly thereafter by their approvals for the larger population of men with chemotherapy-naïve mCRPC. Genomically unselective studies of enzalutamide and abiraterone acetate/prednisone in patients with mCRPC who have not received chemotherapy[11,37,38,39] have demonstrated similar median radiographic PFS and overall survival of approximately 16 and 35 months, respectively. Unfortunately, disease progression eventually occurs and leads to death absent other fatal comorbidities. Thus, an unmet medical need remains to further improve the treatments available for these patients with mCRPC. The development program of which the current study is a part was prompted based on the results of the TOPARP-A study[33] and the established use of NHTs such as enzalutamide in mCRPC patients. Furthermore preclinical data demonstrated potential biochemical basis for synergy between androgen receptor signaling and DNA repair mechanism pathway and the combined inhibition of both pathways selectively leads to synthetic lethality in prostate cancer.[53]

In a recent double-blind, randomized, placebo-controlled Phase 2 study, 142 patients with mCRPC who had previously received docetaxel were randomly assigned to receive olaparib plus abiraterone (n=71) or placebo and abiraterone (n=71). The aim of the study was to assess efficacy of olaparib plus abiraterone in patients regardless of homologous recombination DDR mutations. The primary endpoint was rPFS, with secondary endpoints of safety and tolerability, time to second progression, overall survival, time to first subsequent anticancer therapy, time to second subsequent anticancer therapy, and change in circulating tumor cell count. Olaparib plus abiraterone provided a significant rPFS benefit to mCRPC for patients compared with abiraterone alone. More adverse events were reported with olaparib plus abiraterone than abiraterone alone with nausea (37% vs 18% respectively), anemia (21% vs none respectively), pneumonia (6% vs 4% respectively), and myocardial infraction (6% vs none respectively) being the most frequent. More serious adverse events were reported with olaparib plus abiraterone (34%) than abiraterone alone (18%). There was 1 treatment-related death that occurred in the olaparib plus abiraterone group.[35] In summary, in the light of preclinical and clinical data suggesting that blocking androgen receptor signaling could induce a BRCAness phenotype, making tumors more sensitive to PARP inhibitors TALAPRO-2 will compare the safety and efficacy of talazoparib in combination

090177e19c2fb240\Approved\Approved On: 23-Dec-2022 10:25 (GMT)

with enzalutamide versus placebo in combination with enzalutamide in mCRPC regardless the present of DDR deficiencies. Backbone treatment with enzalutamide is justified given that enzalutamide is currently approved and recommended treatment, irrespective of genomic status, per clinical practice guidelines. In addition, PARP inhibitors have demonstrated substantial clinical efficacy in ovarian cancer and breast cancer with DDR deficiencies. As such, it is expected that talazoparib, may significantly improve clinical outcomes in mCRPC when combined with enzalutamide in all-comers population.

1.6.1. Rationale for Study Treatment Dose

1.6.1.1. Talazoparib

Talazoparib has demonstrated efficacy as single agent at a dose of 1 mg once daily in patients with germline BRCA mutation and locally advanced and/or metastatic breast cancer. Therefore, the first patients enrolled into Part 1 initially received 1 mg once daily.

As of 04 May 2018, evaluation of available preliminary safety data from 13 patients enrolled with a starting dose of talazoparib of 1 mg once daily in combination with 160 mg once daily enzalutamide showed higher than expected dose reductions and interruptions due to Grade 3 hematological toxicities (anemia, thrombocytopenia and neutropenia) which were reported in 7 patients. Dose modifications (reductions and interruptions) were reported in 9 patients and treatment discontinuation in 1 patient. Evaluation of available preliminary talazoparib PK data (C_{trough} at Week 5, n=11 patients who received talazoparib 1 mg once daily) suggested that enzalutamide increased talazoparib C_{trough} at Week 5 Visit by ~2-fold compared to talazoparib steady-state C_{trough} in patients with advanced solid tumors and in patients with breast cancer receiving 1 mg once daily monotherapy. The increase in talazoparib exposure might be due to inhibition of P-glycoprotein by enzalutamide and its N-desmethyl metabolite in the intestine (increasing talazoparib bioavailability) or the renal tubules (reducing talazoparib elimination), or both. Based on these preliminary safety and PK data, talazoparib dose was reduced from 1 mg once daily to 0.5 mg once daily for all patients continuing to receive talazoparib in combination with enzalutamide in Part 1. Additional patients were enrolled in Part 1 and received a talazoparib starting dose of 0.5 mg once daily in combination with enzalutamide to evaluate safety and PK data at the reduced talazoparib dose.

As of 18 October 2018, 13 patients received talazoparib with a starting dose of 1 mg/day in combination with 160 mg once daily enzalutamide for a median of 25.4 weeks (4.0 weeks - 39.6 weeks) and 6 patients received talazoparib with a starting dose of 0.5 mg/day in combination with 160 mg once daily enzalutamide for a median of 11.0 weeks (8.1 weeks - 16.3 weeks). Patients that started talazoparib dosing at 1 mg/day (n=13) had their dose reduced either due to AE (n=6) or per Sponsor communication (n=4) while 3 patients discontinued treatment prior to dose reduction. Adverse events reported in the 4 patients whose dose was reduced per Sponsor communication and the 6 patients starting at 0.5 mg/day showed acceptable safety profile for the 0.5 mg once daily talazoparib dose in combination with 160 mg once daily enzalutamide. Evaluation of PK data from 6 patients who started on 0.5 mg once daily talazoparib and from patients who started on 1 mg once daily and later reduced to 0.5 mg once daily suggested that a starting dose of talazoparib

090177e19c2fb240\Approved\Approved On: 23-Dec-2022 10:25 (GMT)

0.5 mg once daily in combination with enzalutamide is expected to maintain talazoparib AUC_{τ} at levels similar to that obtained with talazoparib 1 mg once daily monotherapy. This indicates that reducing the talazoparib dose to 0.5 mg once daily in combination with enzalutamide is expected to account for the observed interaction and maintain similar talazoparib exposure to that achieved with 1 mg once daily monotherapy with an acceptable safety profile.

Therefore, a talazoparib dose of 0.5 mg once daily will be administered in combination with enzalutamide 160 mg once daily in Part 2 of this study. The starting dose of talazoparib for patients with moderate renal impairment will be 0.35 mg once daily to account for the lower talazoparib clearance in this subpopulation (refer to Section 1.3.3.1).

1.6.1.2. Enzalutamide

Enzalutamide is administered at the approved dose of 160 mg once daily via oral administration with or without food. No enzalutamide dose adjustment is necessary for patients with mild to moderate renal impairment or patients with mild or moderate hepatic impairment given the similar enzalutamide exposure compared to patients with normal organ function.

2. STUDY OBJECTIVES AND ENDPOINTS

The study will include an open-label treatment period, hereafter referred to as Part 1, which will include a limited number of patients to confirm the starting dose of talazoparib for the double-blind period, hereafter referred to as Part 2.

Study objectives and corresponding endpoints for Part 1 and Part 2 are provided in Table 4 and Table 5, respectively.

Table 4. Objectives and Endpoints for Study Part 1 (Open-Label Treatment)

**Primary Objective(s):**	**Primary Endpoint(s):**
• To determine the starting dose of talazoparib when given in combination with enzalutamide during Part 2 (double-blind treatment period).	• Occurrence of target safety events.
**Secondary Objective(s):**	**Secondary Endpoint(s):**
• To characterize the steady state PK of talazoparib and enzalutamide and its N-desmethyl metabolite when given in combination.	• Multiple-dose PK parameters of talazoparib and enzalutamide and its N-desmethyl metabolite (Multiple-dose C_{max}, C_{trough}, T_{max}, AUC_{τ} and CL/F as data permit).
**Exploratory Objective(s):**	**Exploratory Endpoint(s):**
• To explore correlation of changes in CTCs with efficacy outcome parameters.<br>• To identify potential biomarkers associated with response and/or resistance to talazoparib in combination with enzalutamide.<br>• To explore correlations between talazoparib/enzalutamide exposure and biomarker, efficacy and safety outcome parameters if data allow.	• Proportion of patients with conversion from 5 or more CTCs per 7.5 mL at baseline to 4 or fewer CTCs per 7.5 mL post-baseline.<br>• Proportion of patients with conversion from detectable CTCs per 7.5 mL at baseline to CTCs = 0 per 7.5 mL post-baseline.<br>• Proportion of patients with baseline CTCs <5 who show increased CTCs post-baseline.<br>• Molecular profiling of tumor tissue remaining after genomic testing for eligibility and of saliva

090177e19c2fb240\Approved\Approved On: 23-Dec-2022 10:25 (GMT)

Table 4. Objectives and Endpoints for Study Part 1 (Open-Label Treatment)

• To collect banked biospecimens for exploratory research, unless prohibited by local regulations or ethics committee decision.	and ctDNA; circulating protein biomarker profiles.<br>• Potential results from exploratory analyses of banked biospecimens.

CTCs = circulating tumor cells; NHT = novel hormonal therapy; PK = pharmacokinetics.

Table 5. Objectives and Endpoints for Study Part 2 (Double-Blind Treatment)

**Primary Objective(s):**	**Primary Endpoint(s):**
• To demonstrate that talazoparib in combination with enzalutamide is superior to placebo in combination with enzalutamide in prolonging BICR assessed rPFS, in patients with mCRPC unselected for DDR status.<br>• To demonstrate that talazoparib in combination with enzalutamide is superior to placebo in combination with enzalutamide in prolonging BICR assessed rPFS, in patients with mCRPC harboring DDR deficiencies.	• BICR assessed rPFS per RECIST 1.1 (soft tissue disease) and PCWG3 (bone disease) in patients with mCRPC unselected for DDR status.<br>• BICR assessed rPFS per RECIST 1.1 (soft tissue disease) and PCWG3 (bone disease) in patients with mCRPC harboring DDR deficiencies.
**Secondary Objective(s):**	**Secondary Endpoint(s):**
Key Secondary Objectives:<br>• To demonstrate that talazoparib in combination with enzalutamide is superior to placebo in combination with enzalutamide in prolonging OS in patients with mCRPC unselected for DDR status.<br>• To demonstrate that talazoparib in combination with enzalutamide is superior to placebo in combination with enzalutamide in prolonging OS in patients with mCRPC harboring DDR deficiencies.<br>Other Secondary Objectives:<br>• To evaluate anti-tumor activity in patients with mCRPC unselected for DDR status and in patients with mCRPC harboring DDR deficiencies with respect to the following:<br>◦ BICR assessed objective response in measurable soft tissue disease;<br>◦ BICR assessed duration of response in measurable soft tissue disease;<br>◦ PSA response;<br>◦ Time to PSA progression;<br>◦ Time to initiation of cytotoxic chemotherapy;<br>◦ Time to initiation of antineoplastic therapy;<br>◦ Time to first symptomatic skeletal event;<br>◦ PFS on next line therapy (PFS2);	Key Secondary Endpoints:<br>• OS in patients with mCRPC unselected for DDR status (alpha-protected).<br>• OS patients with mCRPC harboring DDR deficiencies (alpha-protected).<br>Other Secondary Endpoints:<br>• Proportion of patients with measurable soft tissue disease at baseline with an objective response per RECIST 1.1 (assessed by BICR) in patients with mCRPC unselected for DDR status and in patients with mCRPC harboring DDR deficiencies.<br>• Duration of soft tissue response per RECIST 1.1 (assessed by BICR) in patients with mCRPC unselected for DDR status and in patients with mCRPC harboring DDR deficiencies.<br>• Proportion of patients with PSA response ≥50% in patients with mCRPC unselected for DDR status and in patients with mCRPC harboring DDR deficiencies.<br>• Time to PSA progression in patients with mCRPC unselected for DDR status and in patients with mCRPC harboring DDR deficiencies.<br>• Time to initiation of cytotoxic chemotherapy in patients with mCRPC unselected for DDR status

090177e19c2fb240\Approved\Approved On: 23-Dec-2022 10:25 (GMT)

Table 5. Objectives and Endpoints for Study Part 2 (Double-Blind Treatment)

• Opiate use for prostate cancer pain.<br>• To evaluate safety of talazoparib and enzalutamide administered in combination.<br>• To evaluate the PK of talazoparib and enzalutamide (and its N-desmethyl metabolite) when dosed in combination.<br>• To evaluate the following patient-reported outcomes in each treatment arm in patients with mCRPC unselected for DDR status and in patients with mCRPC harboring DDR deficiencies:<br>• Pain symptoms;<br>• Cancer-specific global health status/QoL, functioning, and symptoms outcomes;<br>• General health status.	and in patients with mCRPC harboring DDR deficiencies.<br>• Time to initiation of antineoplastic therapy in patients with mCRPC unselected for DDR status and in patients with mCRPC harboring DDR deficiencies.<br>• Time to first symptomatic skeletal event in patients with mCRPC unselected for DDR status and in patients with mCRPC harboring DDR deficiencies.<br>• PFS2 based on investigator assessment separately in patients with mCRPC unselected for DDR status and in patients with mCRPC harboring DDR deficiencies.<br>• Time to opiate use for prostate cancer pain in patients with mCRPC unselected for DDR status and in patients with mCRPC harboring DDR deficiencies.<br>• Incidence of adverse events characterized by type, severity (graded by NCI CTCAE version 4.03), timing, seriousness and relationship to study treatment.<br>• PK characterized by pre-dose trough and post-dose plasma concentrations of talazoparib, enzalutamide and its N-desmethyl metabolite.<br>• PROs in patients with mCRPC unselected for DDR status and in patients with mCRPC harboring DDR deficiencies:<br>• Change from baseline in patient-reported pain symptoms per BPI-SF;<br>• Change from baseline in patient-reported general health status per EQ-5D-5L;<br>• Change from baseline in patient-reported cancer-specific global health status/QoL, functioning, and symptoms per EORTC QLQ-C30;<br>• Time to deterioration in patient-reported pain symptoms per BPI-SF;<br>• Time to definitive deterioration in patient-reported global health status/QoL per EORTC QLQ-C30;<br>• Time to definitive deterioration in patient-reported disease-specific urinary symptoms per EORTC QLQ-PR25.

090177e19c2fb240\Approved\Approved On: 23-Dec-2022 10:25 (GMT)

Table 5. Objectives and Endpoints for Study Part 2 (Double-Blind Treatment)

Exploratory Objective(s):	Exploratory Endpoint(s):
• To explore BICR assessed rPFS in patients with mCRPC without DDR deficiencies.<br>• To explore correlation of changes in CTCs with efficacy outcome parameters.<br>• To identify potential biomarkers associated with response and/or resistance to talazoparib in combination with enzalutamide.<br>• To explore correlations between talazoparib exposure and biomarker, efficacy and safety endpoints if data allow.<br>• To collect banked biospecimens for exploratory research, unless prohibited by local regulations or ethics committee decision.<br>• To explore the concordance of DDR deficiency results between blood and tumor tissue based tests.	• BICR assessed rPFS per RECIST 1.1 (soft tissue disease) and PCWG3 (bone disease) in patients with mCRPC without DDR deficiencies.<br>• Proportion of patients with conversion from 5 or more CTCs per 7.5 mL at baseline to 4 or fewer CTCs per 7.5 mL post-baseline, in patients with mCRPC unselected for DDR status and in patients with mCRPC harboring DDR deficiencies.<br>• Proportion of patients with conversion from detectable CTCs at baseline to CTCs = 0 per 7.5 mL post-baseline, in patients with mCRPC unselected for DDR status and in patients with mCRPC harboring DDR deficiencies.<br>• Proportion of patients with baseline CTCs <5 who show increased CTCs post-baseline, in patients with mCRPC unselected for DDR status and in patients with mCRPC harboring DDR deficiencies.<br>• Molecular profiles of tumor tissue remaining after genomic testing for eligibility during prescreening/screening, and of saliva and, of ctDNA; circulating protein biomarker profile.<br>• Potential results from exploratory analysis of banked biospecimens.<br>• Concordance of DDR deficiency results between blood and tumor tissue based tests.

ctDNA = circulating tumor DNA; CTCAE = Common Toxicity Criteria for Adverse Events; CTCs = circulating tumor cells; DDR = DNA damage repair; EORTC = European Organisation for Research and Treatment of Cancer; QLQ-PR25; EORTC Quality-of-Life Cancer Questionnaire Prostate Cancer Module; EQ-5D-5L = European Quality-of-Life 5-Domain 5-Level Scale; NCI = National Cancer Institute; PFS = progression-free survival; PK = pharmacokinetics; PSA = prostate-specific antigen; QLQ-C30 = Quality-of-Life Cancer Questionnaire.

Efficacy assessments are described in Section 7.2. Response in soft tissue disease will be evaluated only on CT or MRI per Response Evaluation Criteria in Solid Tumors version 1.1 (RECIST 1.1), and bone disease progression will be evaluated only on bone scan per Prostate Cancer Working Group 3 (PCWG3) [59] by the investigator for Part 1 and BICR for Part 2. Safety assessments are described in Section 7.9. An external data monitoring committee (E-DMC) will conduct unblinded safety reviews throughout Part 2 of the study and will also conduct unblinded efficacy reviews at the pre-planned interim analyses (Section 9.8). Frequency of the E-DMC review meetings will be defined in the E-DMC charter.

090177e19c2fb240\Approved\Approved On: 23-Dec-2022 10:25 (GMT)

3. STUDY DESIGN

This is an international, Phase 3, two-part study enrolling patients with mCRPC where no systemic cancer treatments have been initiated after documentation of CRPC with the exception of ADT and first generation anti-androgen agents.

Part 1 was open-label and non-randomized, and evaluated the safety, tolerability and PK of talazoparib in combination with enzalutamide. Part 2 is randomized, double-blind, and placebo-controlled, and will evaluate the safety and efficacy of talazoparib in combination with enzalutamide versus placebo in combination with enzalutamide.

Two cohorts will be enrolled in Part 2. Cohort 1 will enroll approximately 750 mCRPC patients unselected for DDR status (all-comers population). Although patients will be unselected for DDR status, DDR efficiency assessment prior to randomization is required for stratification.

Once enrollment in Cohort 1 is complete, enrollment will continue but will be restricted to patients harboring DDR gene mutations likely to sensitize to PARP inhibition (Cohort 2). Approximately 268 additional patients harboring DDR gene mutations likely to sensitize to PARP inhibition (DDR-deficient) will be enrolled into Cohort 2. Patients who are DDR-deficient from Cohorts 1 and 2 will be combined such that a total of approximately 380 DDR-deficient patients are enrolled to assess efficacy separately within this population.

Approximately 1037 men (19 enrolled in Part 1 and approximately 1018 in Part 2) with mCRPC will be enrolled. Genomic screening to identify alterations in DDR genes is optional for patients in Part 1, but is required for enrollment in Part 2.

Mutational status for patients randomized in Part 2 will be determined by testing for the presence of mutations in defined DDR genes likely to sensitize to PARP inhibition using the FoundationOne® or the FoundationOneLiquid® next generation sequencing (NGS) gene panel, consisting of over 300 genes. For enrolled subjects, results for these defined DDR genes and other genes included in the FoundationOne® panels used in these tests will also be used to support the exploratory correlative analyses detailed in Section 7.5.

This study will be conducted at approximately 270 sites worldwide.

Eligible patients may have received taxane-based chemotherapy or NHT (second generation anti-androgens are excluded) in the castration-sensitive prostate cancer (CSPC) setting.

Genomic screening of circulating tumor DNA (blood/plasma) or tumor tissue (de novo biopsy or archival, previously collected tissue) to identify alterations in DDR genes will be done by testing for the presence of mutations likely to sensitize to PARP inhibition using NGS-based gene panel tests. (Refer to the Laboratory manual for specific information on the genes included in the NGS panel; assessment described in Section 7.1). This testing will be performed during an optional prescreening visit or during the screening period. For patients enrolling in Part 1, submission and testing of tumor tissue is optional and patients do not need to have a genomic deficiency in DDR genes to enroll. However, patients enrolling in Part 2 are required to give consent during an optional prescreening period or at screening to submit both a peripheral blood sample (liquid biopsy) and sufficient tumor tissue (de novo or

090177e19c2fb240\Approved\Approved On: 23-Dec-2022 10:25 (GMT)

archived tissue) for genomic assessment; the result from the central laboratory is required prior to patient randomization in to Part 2 of the study for stratification. Prior (ie, historical) testing results of tumor tissue performed using the FoundationOne® test may be considered upon Sponsor pre-approval.

Preferably both liquid and tumor tissue (or request for approval to use historical data) will be submitted at prescreening. In cases where historical FoundationOne® test results are approved by the Sponsor, tumor tissue is not required for the study, but may be requested by the Sponsor if available.

If blood and tumor tissue are not submitted at the same time, patients will be stratified according to the results obtained from the analysis of either the liquid or the tumor tissue, depending on which test result is available. If the test results are inconclusive or no result could be obtained due, for example, to technical reasons the investigator may submit the alternative sample (tumor tissue or blood) for testing as well. If the blood and tumor tissue are not submitted at the same time and results required for eligibility and stratification are already available, the sample (tumor tissue or liquid biopsy) submitted at a later point (but prior to randomization) will be analyzed for exploratory concordance analyses and to help support development of potential diagnostic tests for talazoparib for use in this and future clinical trials. If results from liquid and tumor tissue sample are available prior to randomization and results are not concordant for the presence of the selected DDR mutation(s), positive DDR results will be used for eligibility and stratification regardless of the tissue used to generate the data.

A patient will participate in up to 5 periods: prescreening (optional), screening, open-label (Part 1) or double-blind treatment (Part 2), safety follow-up, and long-term follow-up. The study schematic is provided in Figure 2. Testing for tumor DDR gene mutational status will be performed at prescreening or screening. Details regarding conduct of Part 1 and Part 2 are provided in Section 3.1 and Section 3.2, respectively.

For all patients, study treatment (including enzalutamide) should continue until-radiographic progression is determined by BICR (Part 2) or investigator (Part 1) unless in the opinion of the investigator the patient is still deriving benefit at this time, or following radiographic progression the patient is then no longer clinically benefitting in the opinion of the investigator, or an adverse event leading to permanent study treatment discontinuation, or patient decision to discontinue treatment, or death.

For patients remaining on study treatment beyond radiographic progression, additional therapy (ie, radiotherapy or surgery) will be allowed, following consultation with the Sponsor. Specifically, palliative radiotherapy may be used for the treatment of pain at the site of bone metastases that were present at baseline. Patients should continue to be monitored as per usual schedule (see Table 2).

If talazoparib or talazoparib/placebo is permanently discontinued due to an AE, dosing with enzalutamide may continue. If enzalutamide is permanently discontinued due to an AE, the talazoparib or talazoparib/placebo dose at the time of enzalutamide discontinuation may be doubled after 6 weeks of enzalutamide discontinuation, based on patient's safety and tolerability and only after investigator discussion with the sponsor.

090177e19c2fb240\Approved\Approved On: 23-Dec-2022 10:25 (GMT)

France only: During Part 2, the discontinuation of study treatment(s) based on increases in PSA levels is not required if the patient entered the study base on eligibility criteria of progressive disease per soft tissue progression or bone disease progression. If the patient entered the study based on eligibility criteria of progressive disease solely per PSA progression (see Section 4.1), study treatment(s) must be discontinued if PSA continues to rise above maximum eligibility level without an initial confirmed biological response (PSA response) without any clinical or radiological progression. These patients are to continue to be followed for radiographic progression and survival status.

Safety follow-up visit will occur 28 days after permanent treatment discontinuation of all study treatments (either the combination or enzalutamide or talazoparib or talazoparib/placebo whichever is later) or before initiation of a new antineoplastic or investigational therapy, whichever occurs first.

Long-term follow-up will begin after safety follow-up and will follow the radiographic imaging schedule (ie, every 8 weeks through Week 25 and then every 12 weeks thereafter until death, withdrawal of consent for follow-up, or study termination by the Sponsor). Radiographic imaging (for Part 1 and 2) and PSA assessment (for Part 2 only) should continue during long-term follow-up for patients who permanently discontinue study treatment(s) for any reason other than radiographic progression until radiographic progression as determined by the investigator (patients in Part 1) or by the investigator and BICR (patients enrolled in Part 2) regardless of the initiation of a new antineoplastic therapy. Survival status, diagnosis of myelodysplastic syndrome or acute myeloid leukemia, onset of symptomatic skeletal events,[58] and initiation of a new antineoplastic therapy (or therapies) will be monitored until the patient dies or withdraws consent for follow-up, or the study is terminated by the Sponsor.

An independent E-DMC will be established to review interim analysis of radiographic PFS during Part 2 (see details in Section 9.8) and analyses of aggregate safety data. The roles and responsibilities of the E-DMC will be described in a separate charter.

For all patients, disease status will be assessed at screening and at regular intervals (as well as when investigator assessment of signs and symptoms is warranted) during the study by computed tomography (CT) scans (chest, abdomen, pelvis) or magnetic resonance imaging (MRI) (abdomen, pelvis) for assessment only of soft tissue disease, whole-body bone scan, PSA measurements, and patient-reported outcome measures. To determine the primary endpoint of radiographic progression, radiographic assessments will be performed at screening, every 8 weeks from Week 9 through Week 25, and every 12 weeks thereafter until radiographic progression is determined by investigator (Part 1) or investigator and BICR (Part 2) regardless of the initiation of a new antineoplastic therapy. Response and progression for soft tissue disease will be evaluated per Response Evaluation Criteria in Solid Tumors version 1.1 (RECIST 1.1) and bone disease progression will be evaluated only per Prostate Cancer Working Group 3 (PCWG3).

Time to cytotoxic chemotherapy and time to opiate use for prostate cancer pain will be evaluated with concomitant and follow-up medication assessments.

090177e19c2fb240\Approved\Approved On: 23-Dec-2022 10:25 (GMT)

In Part 2, patient-reported outcomes will be assessed to determine time to deterioration in pain using the Brief Pain Inventory Short Form (BPI-SF); to evaluate global health status/quality of life (QoL), functions, and symptoms using the EORTC QLQ-C30 and QLQ-PR25 questionnaires; and general health status using the EQ-5D-5L health questionnaire. These assessments will be performed at:

- Baseline (Day 1);
- Every 4 weeks through Week 53, or centrally determined radiographic progression, whichever is earlier;
- Every 8 weeks after Week 53 until centrally determined radiographic progression when no such progression had been previously documented;
- Every 12 weeks after centrally determined radiographic progression until end of study.

Procedures to account for any missing data for patient-reported outcome assessments will be described in the statistical analysis plan.

Unless prohibited by local regulation or ethics committee decision, a blood sample optimized for DNA analysis will be collected on Day 1 prior to the first dose of talazoparib or matching placebo and retained as a banked biospecimen for potential pharmacogenomics/genomic/biomarker analyses related to drug response and prostate cancer. For enrolled subjects, exploratory targeted and/or whole exome/genome sequencing and/or transcriptome and/or epigenetic analyses will be performed using the de novo or archival tumor tissue samples submitted at prescreening or screening remaining after eligibility assessment (optional for patients in Part 1). Additional patient samples including saliva and blood will be obtained for exploratory biomarker assessments as described in the Schedule of Activities and Section 7.5.

Hematology, reticulocyte count, erythropoietin, serum folate, B12 and serum chemistry samples will be collected every 2 weeks through Week 17, every 4 weeks through Week 53, and every 8 weeks thereafter until the end of treatment; a final sample will be collected approximately 28 days after the last dose of study treatment or prior to initiation of new antineoplastic therapy, whichever occurs first. Investigators may collect samples for laboratory assessment up to 3 days prior to a scheduled visit to guide decision making. Samples for analysis of PSA will be collected every 8 weeks until confirmation of radiographic progression, and other general and laboratory assessments will be performed at regular intervals throughout the study according to the schedules of activities (Table 1, Table 2, and Table 3).

Safety will be assessed by adverse events, physical examinations, vital signs, and clinical laboratory tests. Assessment of abnormal liver tests is described in Section 5.6.3.

090177e19c2fb240\Approved\Approved On: 23-Dec-2022 10:25 (GMT)

Figure 2. Study Schematic

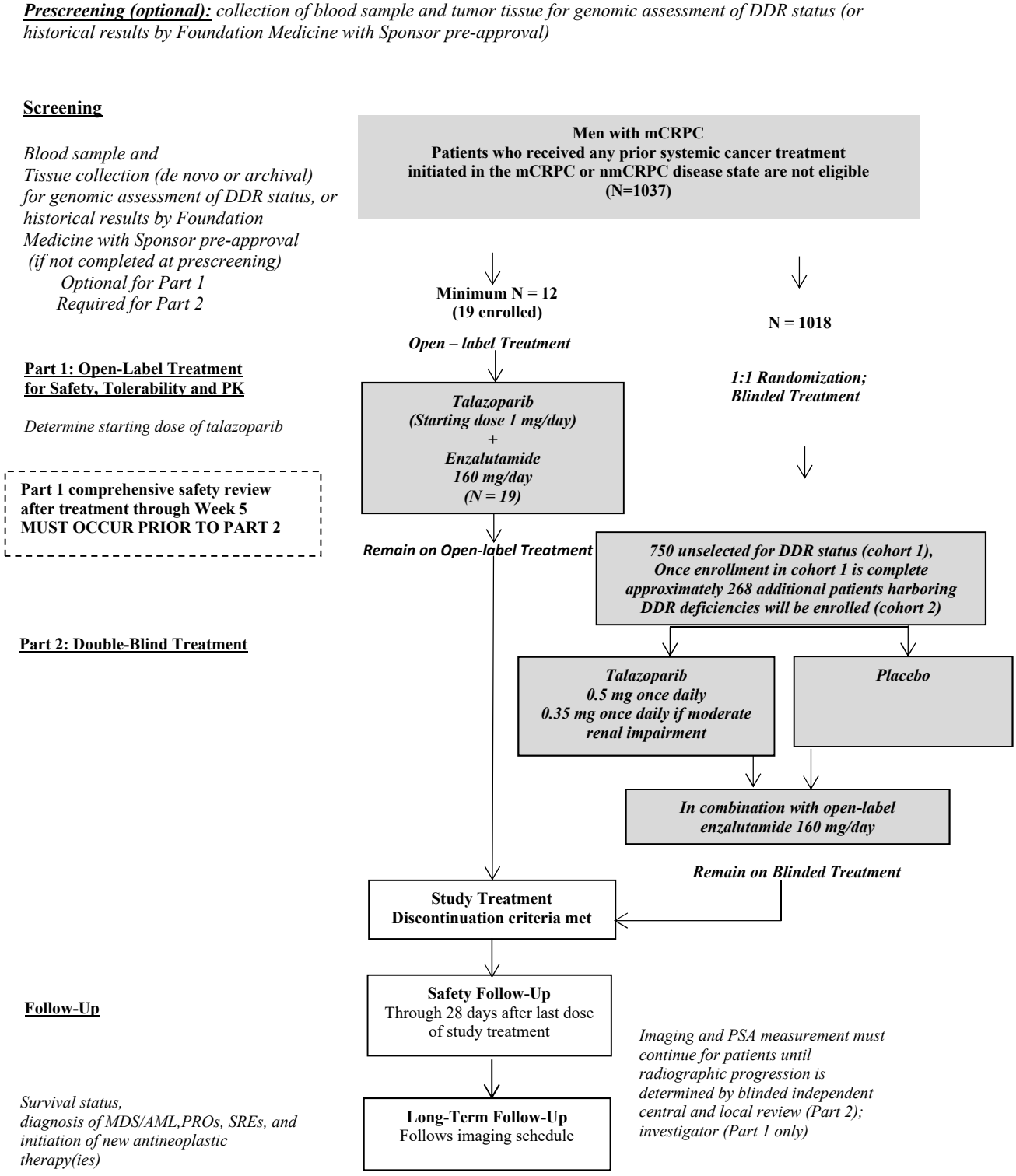

090177e19c2fb240\Approved\Approved On: 23-Dec-2022 10:25 (GMT)

3.1. Part 1: Open-Label Treatment

Patients enrolled in Part 1 were not required to test for DDR deficiency but were permitted to submit tissue for genomic testing. Patients with moderate renal impairment (estimated glomerular filtration rate [eGFR] 30-59 mL/min/1.73 m^2 by the Modification of Diet in Renal Disease [MDRD] equation) at screening were not eligible for Part 1.

The preliminary results summarizing the safety profile and PK data of talazoparib in combination with enzalutamide in Part 1 are discussed in Section 1.6.1. Briefly, enzalutamide increased talazoparib C_{trough} at Week 5 Visit by ~2-fold compared to talazoparib steady-state C_{trough} in patients with advanced solid tumors and in patients with breast cancer receiving 1 mg once daily monotherapy. The increase in talazoparib exposure was associated with higher than expected incidence of Grade 3/4 hematological AEs. The results of Part 1 suggested that reducing the talazoparib dose to 0.5 mg once daily in combination with enzalutamide is expected to account for the observed interaction and maintain similar talazoparib exposure to that achieved with 1 mg once daily monotherapy with an acceptable safety profile. Therefore, a talazoparib dose of 0.5 mg once daily will be administered in combination with enzalutamide 160 mg once daily in Part 2 of this study (refer to Section 1.6.1). The starting dose of talazoparib for patients with moderate renal impairment will be 0.35 mg once daily to account for the lower talazoparib clearance in this subpopulation (refer to Section 1.3.3.1).

Patients receiving treatment in Part 1 will undergo study assessments (Table 2 and Table 3) and may continue treatment despite radiographic progression as long as in the opinion of the investigator the patient is still deriving benefit, and do not meet other discontinuation criteria.

3.2. Part 2: Double-Blind Treatment

Approximately 1018 patients are planned to be enrolled in Part 2. Initially, approximately 750 patients unselected for DDR deficiencies (referred to as “all-comers population”) will be enrolled. Although patients will be unselected for DDR status, DDR deficiency assessment from a blood sample or the most recent tumor tissue sample completed prior to randomization is required for stratification. Once enrollment is complete in the all-comers population, enrollment will continue in a separate cohort of patients harboring DDR deficiencies. Additional all-comers patients will be randomized in China while randomization into Cohort 2 continues as described in Appendix 6.

Patients will be randomly assigned to either of 2 treatment groups as follows:

- Talazoparib in combination with enzalutamide.
- Placebo capsules identical in appearance to talazoparib capsules in combination with enzalutamide.

Talazoparib or identical placebo treatment will be blinded. Enzalutamide will be open-label.

090177e19c2fb240\Approved\Approved On: 23-Dec-2022 10:25 (GMT)

In addition to analyses in the all-comers population (Cohort 1), analyses will be performed on the DDR-deficient population which will include patients who are identified as DDR-deficient from the all-comers population combined with patients from the DDR-deficient Cohort 2. Based on review of safety and PK data from Part 1 of this study (see Section 1.6.1) the dose of talazoparib to be given in combination with enzalutamide is 0.5 mg once daily. During Part 2, patients with moderate renal impairment (eGFR 30-59 mL/min/1.73 m^2 by the MDRD equation) at screening may be enrolled and the talazoparib dose will be 0.35 mg once daily.

Patients will take all study treatments daily (at approximately the same time) and undergo the assessments shown in Table 2 and Table 3 until permanent discontinuation criteria are met.

Dose modifications are permitted and are described in Section 5.6.

CCI [redacted]

CCI [redacted]

090177e19c2fb240\Approved\Approved On: 23-Dec-2022 10:25 (GMT)

Analyses on the all-comers population will include patients randomized into the first cohort of patients unselected for DDR status. Analysis on the DDR-deficient population will include DDR-deficient patients enrolled into Cohort 2 as well as any patients enrolled into Cohort 1 who are DDR-deficient.

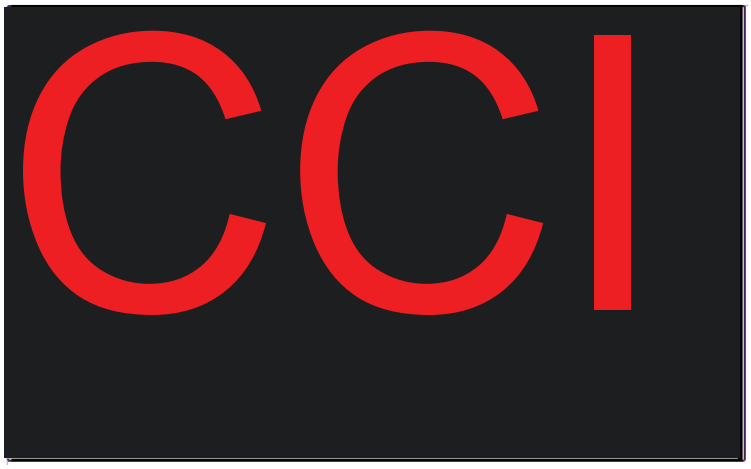

4. PATIENT ELIGIBILITY CRITERIA

This study can fulfill its objectives only if appropriate patients are enrolled. The following eligibility criteria are designed to select patients for whom participation in the study is considered appropriate. All relevant medical and nonmedical conditions should be taken into consideration when deciding whether a particular patient is suitable for this protocol.

Patient eligibility should be reviewed and documented by an appropriate member of the investigator's study team before patients are included in the study.

4.1. Inclusion Criteria

Patients must meet all of the following inclusion criteria to be eligible for enrollment into the study:

1. At least 18 years of age. For Japan, at least 20 years of age.

2. Histologically or cytologically confirmed adenocarcinoma of the prostate without small cell or signet cell features. If the patient does not have a prior histological diagnosis, a baseline de novo biopsy must be used to confirm the diagnosis and to support biomarker analysis.

3. Asymptomatic or mildly symptomatic metastatic castration resistant prostate cancer (mCRPC) (score on BPI-SF Question #3 must be <4).

090177e19c2fb240\Approved\Approved On: 23-Dec-2022 10:25 (GMT)

4. For enrollment into Part 2 only (optional in Part 1): assessment of DDR mutation status by prospective analysis of blood (liquid biopsy), or tissue (de novo or archival tissue), or historical analysis (with Sponsor pre-approval), of most recent tumor tissue per FoundationOne® testing. (Note: for patients enrolling in Part 1, DDR deficiency testing is optional).

    - Biopsies of the brain, lung/mediastinum, pancreas, or endoscopic procedures extending beyond the esophagus, stomach, or bowel may not be performed for the sole purpose of determining study eligibility.

5. For enrollment into Part 2 only (optional for Part 1): Unless prohibited by local regulations or ethics committee decision, consent to a saliva sample collection for retrospective sequencing of the same DDR genes tested on tumor tissue and blood (liquid biopsy), or a subset thereof, and to serve as a germline control in identifying tumor mutations.

6. Surgically or medically castrated, with serum testosterone ≤50 ng/dL (≤1.73 nmol/L) at screening. Ongoing androgen deprivation therapy with a gonadotropin releasing hormone (GnRH) agonist or antagonist for patients who have not undergone bilateral orchiectomy must be initiated at least 4 weeks before Day 1 (Part 1) or randomization (Part 2) and must continue throughout the study.

7. Metastatic disease in bone documented on bone scan or in soft tissue documented on CT/MRI scan. Scans obtained as part of standard of care in the 6 weeks (42 days) prior to Day 1 (Part 1) or randomization (Part 2) can be used if they meet study requirements. Measurable soft tissue disease is not required. (Adenopathy below the aortic bifurcation alone does not qualify).

8. Progressive disease at study entry in the setting of medical or surgical castration as defined by 1 or more of the following 3 criteria:

    - Prostate specific antigen (PSA) progression defined by rising PSA of at least 2 consecutive rises in most recent PSA to be documented over a reference value (measure 1) taken at least 7 days apart within the last 12 months. If the third PSA measure is not greater than the second measure, a fourth PSA measure is required to be taken and be greater than the second measure. The third (or the fourth) confirmatory PSA should be taken within 4 weeks prior to randomization. The third (or the fourth) PSA value, obtained before randomization must be ≥1 µg/L if qualifying only by PSA progression.

    - Soft tissue disease progression as defined by RECIST 1.1.

    - Bone disease progression defined by Prostate Cancer Working Group (PCWG3) with 2 or more new metastatic bone lesions on a whole body radionuclide bone scan.

090177e19c2fb240\Approved\Approved On: 23-Dec-2022 10:25 (GMT)

9. Ongoing bisphosphonate or denosumab use prior to Day 1 (Part 1) or randomization (Part 2) is allowed but not mandatory.

10. Eastern Cooperative Oncology Group (ECOG) performance status ≤1.

11. Life expectancy ≥12 months as assessed by the investigator.

12. Able to swallow the study treatment and have no known intolerance to study treatments or excipients.

13. Sexually active participants that in the opinion of the investigator are capable of ejaculating, must agree to use a condom when having sex with a partner (female or male) from the time of the first dose of study treatment through 4 months after last dose of study treatment. Must also agree for female partner of childbearing potential to use an additional highly effective form of contraception (Section 4.4.1) from the time of the first dose of study treatment through 4 months after last dose of study treatment when having sex with a non-pregnant female partner of childbearing potential.

14. Must agree not to donate sperm from the first dose of study treatment to 4 months after the last dose of study treatment.

15. Evidence of a personally signed and dated informed consent document (and molecular prescreening consent if appropriate) indicating that the patient [or a legally acceptable representative] has been informed of all pertinent aspects of the study.

16. Willing and able to comply with scheduled visits, treatment plan, laboratory tests, and other study procedures.

4.2. Exclusion Criteria

Patients with any of the following characteristics/conditions will be excluded:

1. Any prior systemic cancer treatment initiated in the non-metastatic CRPC or mCRPC disease state. (ADT and first generation anti-androgens received in the CRPC disease state are NOT exclusionary).

2. Patients whose only evidence of metastasis is adenopathy below the aortic bifurcation.

3. Prior treatment with second-generation androgen receptor inhibitors (enzalutamide, apalutamide, and darolutamide), a PARP inhibitor, cyclophosphamide, or mitoxantrone for prostate cancer.

4. Prior treatment with platinum-based chemotherapy within 6 months (from the last dose) prior to Day 1 (Part 1) or randomization (Part 2), or any history of disease progression on platinum-based therapy within 6 months (from the last dose).

    - Prior docetaxel for mCSPC is allowed if more than 4 weeks have elapsed from the last dose of docetaxel.

090177e19c2fb240\Approved\Approved On: 23-Dec-2022 10:25 (GMT)

5. Treatment with cytotoxic chemotherapy which includes but is not limited to docetaxel, biologic therapy including sipuleucel-T, or radionuclide therapy received in the castration-sensitive prostate cancer is NOT exclusionary if discontinued in the 28 days prior to Day 1 (Part 1) or randomization (Part 2). Prior treatment with abiraterone in the castration-sensitive settings is not exclusionary if discontinued prior to randomization. Hormonal therapy (eg, bicalutamide, nilutamide, flutamide, estrogens) are not exclusionary if discontinued prior to randomization. Prednisone >10 mg/day (or equivalents) is exclusionary.

6. Treatment with any investigational agent within 4 weeks before Day 1 (Part 1) or randomization (Part 2).

7. Prior treatment with opioids for pain related to either primary prostate cancer or metastasis within 28 days prior to Day 1 (Part 1) or randomization (Part 2).

8. Current use of potent P-gp inhibitors within 7 days prior to Day 1 (Part 1) or randomization (Part 2). For a list of potent P-gp inhibitors, and other medications which are exclusionary because of interaction with either talazoparib or enzalutamide, refer to Section 5.9.

9. Major surgery (as defined by the investigator) within 2 weeks before Day 1 (Part 1) or randomization (Part 2), or palliative localized radiation therapy within 3 weeks before randomization (Part 2).

10. Clinically significant cardiovascular disease, including any of the following:

    - Myocardial infarction or symptomatic cardiac ischemia within 6 months before Day 1 (Part 1) or randomization (Part 2).

    - Congestive heart failure New York Heart Association class III or IV.

    - History of clinically significant ventricular arrhythmias (eg, sustained ventricular tachycardia, ventricular fibrillation, torsade de pointes) within 1 year before screening.

    - History of Mobitz II second degree or third degree heart block unless a permanent pacemaker is in place.

    - Hypotension as indicated by systolic blood pressure <86 mm Hg at screening.

    - Bradycardia as indicated by a heart rate of <45 beats per minute on the screening electrocardiogram.

    - Uncontrolled hypertension as indicated by systolic blood pressure >170 mm Hg or diastolic blood pressure >105 mm Hg at screening. However, patients can be rescreened after adequate control of blood pressure is achieved.

090177e19c2fb240\Approved\Approved On: 23-Dec-2022 10:25 (GMT)

11. Significant renal dysfunction as defined by any of the following laboratory abnormalities:

    - eGFR <30 mL/min/1.73 m^2 by the MDRD equation (available via www.mdrd.com, see Appendix 5).

    - *Patients enrolled in Part 1 only:* Moderate renal impairment (eGFR 30-59 mL/min/1.73 m^2) at screening.

12. Significant hepatic dysfunction as defined by any of the following laboratory abnormalities on screening labs:

    - Total serum bilirubin >1.5 times the upper limit of normal (ULN) (>3 × ULN for patients with documented Gilbert syndrome or for whom indirect bilirubin concentrations suggest an extrahepatic source of elevation).

    - Aspartate aminotransferase (AST) or alanine aminotransferase (ALT) >2.5 times ULN (>5 × ULN if liver function abnormalities are due to hepatic metastasis).

    - Albumin <2.8 g/dL.

13. Absolute neutrophil count (ANC) <1500/μL, platelets <100,000/μL, or hemoglobin <9 g/dL (may not have received growth factors or blood transfusions within 14 days before obtaining the hematology laboratory tests at screening).

14. Known or suspected brain metastasis or active leptomeningeal disease.

15. Symptomatic or impending spinal cord compression or cauda equina syndrome.

16. Any history of myelodysplastic syndrome, acute myeloid leukemia, or prior malignancy *except* for any of the following:

    - Carcinoma in situ or non melanoma skin cancer.

    - Any prior malignancies ≥3 years before randomization with no subsequent evidence of recurrence or progression regardless of the stage.

    - Stage 0 or Stage 1 cancer <3 years before randomization that has a remote probability of recurrence or progression in the opinion of the investigator.

17. In the opinion of the investigator, any clinically significant gastrointestinal disorder affecting absorption.

18. Fertile male subjects who are unwilling or unable to use a condom and highly effective methods of contraception as appropriate as outlined in this protocol for the duration of the study and for 4 months after the last dose of investigational product.

090177e19c2fb240\Approved\Approved On: 23-Dec-2022 10:25 (GMT)

19. Investigator site staff members directly involved in the conduct of the study and their family members, site staff members otherwise supervised by the investigator, or patients who are Pfizer employees, including their family members, directly involved in the conduct of the study.

20. Other acute or chronic medical (concurrent disease, infection, or comorbidity) or psychiatric condition including recent (within the past year) or active suicidal ideation or behavior or laboratory abnormality that interferes with ability to participate in the study, may increase the risk associated with study participation or investigational product administration, or may interfere with the interpretation of study results and, in the judgment of the investigator, would make the patient inappropriate for entry into this study.

21. History of seizure or any condition that may predispose to seizure (eg, prior cortical stroke, significant brain trauma). Also, history of loss of consciousness or transient ischemic attack within 12 months of randomization (Part 2).

4.3. Randomization Criteria

In Part 2, central randomization (1:1) will be used to assign eligible patients to one of the treatment groups. Randomization will be stratified by the following factors:

- Previous treatment with NHT or taxane-based chemotherapy for CSPC (yes/no).
    - Prior treatment with NHT refers only to prior treatment with abiraterone or orteronel in the castration sensitive setting since starting with amendment 4 prior treatment with enzalutamide, darolutamide or apalutamide is prohibited.
- DDR mutation status (deficient vs. non deficient/unknown):
    - In the case of a test failure due to not meeting specified quality control metrics, or insufficient or inadequate blood or tumor tissue sample, the patient DDR mutational status will be considered unknown.
    - If results from liquid and tumor tissue sample are available prior to randomization and results are not concordant for the presence of the selected DDR mutation(s), positive DDR results will be used for stratification regardless of the tissue used to generate the data.

The stratification factors will be specified by the investigator and recorded in the Interactive Web Response System (IWRS) before randomization. Refer to Section 5.1 for additional information.

Initially 750 patients will be enrolled regardless of DDR mutation status (Cohort 1). These patients will be stratified by DDR mutation status but will be considered an all-comers population as there will be not restrictions on the number of patients with or without DDR deficiencies or with unknown mutation status.

090177e19c2fb240\Approved\Approved On: 23-Dec-2022 10:25 (GMT)

Once enrollment is complete in the all-comers population, additional patients harboring DDR deficiencies will be enrolled (Cohort 2) until there are approximately 380 patients with mCRPC harboring DDR deficiencies including patients with DDR deficiencies from Cohorts 1 and 2. Analysis on the DDR-deficient population will include DDR-deficient patients enrolled into Cohort 2 as well as any patients within the first cohort who are DDR-deficient.

4.4. Lifestyle Requirements

4.4.1. Contraception

All sexually active male patients that in the opinion of the investigator are capable of ejaculating must agree to prevent potential transfer to and exposure of partner (female or male) of drug through ejaculate (even after vasectomy) by using a condom consistently and correctly, beginning with the first dose of study treatment and continuing for at least 4 months after the last dose of study treatment.

In addition, participants with female partners of child bearing potential will be advised to use an additional highly effective method of contraception. Highly effective methods of contraception are those that, alone or in combination, result in a failure rate of less than 1% per year when used consistently and correctly (ie, perfect use) and include the following.

The investigator is to assess if the patient is biologically capable of fathering a child or exposing a fetus via ejaculate. If capable, at screening instruct the patient to use a condom to avoid pregnancy and/or fetal exposure and verify at every visit that the patient is following instructions provided. The investigator or designee will instruct the subject to call immediately if the selected contraception method is discontinued. Investigators should document this/these conversations with the patient in the medical records.

Highly effective methods of contraception are those that, alone or in combination, result in a failure rate of less than 1% per year when used consistently and correctly (ie, perfect use) and include the following:

1. Established use of hormonal methods of contraception associated with inhibition of ovulation (eg, oral, inserted, injected, implanted, transdermal), provided the subject or male subject's female partner plans to remain on the same treatment throughout the entire study and has been using that hormonal contraceptive for an adequate period of time to ensure effectiveness.

2. Correctly placed copper-containing intrauterine device (IUD).

3. Male sterilization with absence of sperm in the post-vasectomy ejaculate.

4. Bilateral tubal ligation/bilateral salpingectomy or bilateral tubal occlusive procedure (provided that occlusion has been confirmed in accordance with the device's label).

090177e19c2fb240\Approved\Approved On: 23-Dec-2022 10:25 (GMT)

Note: Sexual abstinence, defined as completely and persistently refraining from all heterosexual intercourse (including during the entire period of risk associated with the study treatments) may obviate the need for contraception ONLY if this is the preferred and usual lifestyle of the subject.

All fertile male must agree not to donate sperm from the first dose of study treatment to 4 months after the last dose of study treatment.

4.5. Sponsor's Qualified Medical Personnel

The contact information for the sponsor's appropriately qualified medical personnel for the study is documented in the study contact list located in the team SharePoint site.

To facilitate access to appropriately qualified medical personnel on study-related medical questions or problems, patients are provided with a contact card. The contact card contains, at a minimum, protocol and investigational product identifiers, patient study numbers, contact information for the investigator site, and contact details for a contact center in the event that the investigator site staff cannot be reached to provide advice on a medical question or problem originating from another healthcare professional not involved in the patient's participation in the study. The contact number can also be used by investigator staff if they are seeking advice on medical questions or problems; however, it should be used only in the event that the established communication pathways between the investigator site and the study team are not available. It is therefore intended to augment, but not replace, the established communication pathways between the investigator site and the study team for advice on medical questions or problems that may arise during the study. The contact number is not intended for use by the patient directly, and if a patient calls that number, he or she will be directed back to the investigator site.

5. STUDY TREATMENTS

For the purposes of this study, and per International Conference on Harmonization (ICH) guidelines, investigational product is defined as a pharmaceutical form of an active ingredient or placebo being tested or used as a reference/comparator in a clinical study, including a product with a marketing authorization when used or assembled (formulated or packaged) in a way different from the approved form, or when used for an unapproved indication, or when used to gain further information about an approved use (ICH E6 1.33).

For this study, talazoparib or placebo is being investigated in combination with enzalutamide.

5.1. Allocation to Treatment

For Part 1, talazoparib and enzalutamide were open-label. Every patient signing either the prescreening or screening informed consent document (ICD) will be assigned a single subject/patient identification (SSID) number.

090177e19c2fb240\Approved\Approved On: 23-Dec-2022 10:25 (GMT)

For Part 2, allocation of patients to talazoparib or identical placebo will be blinded, while enzalutamide will be open-label. Randomization to talazoparib or placebo will proceed through the use of the IWRS based on the following stratification factors:

- Previous treatment with NHT or taxane-based chemotherapy for CSPC (yes/no).
    - Prior treatment with NHT refers only to prior treatment with abiraterone or orteronel in the castration sensitive setting since starting with amendment 4 prior treatment with enzalutamide, darolutamide or apalutamide is prohibited.
- DDR mutation status (deficient vs. non deficient/unknown):
    - In the case of a test failure due to not meeting specified quality control metrics, or insufficient or inadequate blood or tumor tissue sample, the patient DDR mutational status will be considered unknown.
    - If results from liquid and tumor tissue sample are available prior to randomization and results are not concordant for the presence of the selected DDR mutation(s), positive DDR results will be used for stratification regardless of the tissue used to generate the data.

The site personnel (study coordinator or specified designee) will be required to enter or select information including but not limited to the user's identification (ID) and password and the protocol number. The site personnel will then be provided with an SSID number. After confirming eligibility and obtaining approval from Pfizer personnel or designee, site personnel will randomize the patient via IWRS. The patient's randomization number and a dispensable unit (DU) or container number will be provided. The IWRS will provide a confirmation report containing the SSID number, randomization number, and DU or container number assigned. The confirmation report must be stored in the site's files.

The study-specific IWRS reference manual will provide the contact information and further details on the use of the IWRS system.

5.2. Breaking the Blind

Part 2 of the study will be patient and investigator blinded to talazoparib or matching placebo; enzalutamide will be open-label.

At the initiation of Part 2, the investigator site will be instructed on the method for breaking the blind. The method will be an electronic process. Blinding codes should be broken only in exceptional circumstances when knowledge of the actual treatment code is absolutely essential for further management of the subject. Investigators are encouraged to discuss with a member of the study team if they believe that unblinding is necessary. When the blinding code is broken, the reason must be fully documented and entered on the case report form (CRF).

090177e19c2fb240\Approved\Approved On: 23-Dec-2022 10:25 (GMT)

5.3. Patient Compliance

In Part 1, for self-administration of talazoparib and enzalutamide at home, compliance will be captured by the sites' drug accountability data. In Part 2, patients will record the daily self-administration of all study treatments (talazoparib/placebo and enzalutamide) using an electronic diary. Data entered in the diary will be compared with drug accountability done at the site prior to dispensing additional study treatment. In the event of unexplained discrepancies, drug accountability data will prevail in determining patients' degree of compliance, which will be recorded in the CRF. Patients will be considered out of compliance if ≥20% of monthly doses of each treatment are missed.

5.4. Investigational Product Supplies

5.4.1. Dosage Forms and Packaging

5.4.1.1. Talazoparib and Identical Placebo

Talazoparib will be provided as capsules for oral administration containing talazoparib tosylate formulated with silicified microcrystalline cellulose filled into hydroxypropyl methylcellulose as immediate-release capsules. The 0.25 mg and 0.1 mg capsules will be supplied in separate bottles and labeled according to local regulatory requirements. Talazoparib is packaged in induction sealed, high density polyethylene bottles with child resistant caps of a single strength per bottle.

Placebo capsules, which do not contain talazoparib, will be identical in appearance to each dosage strength of talazoparib capsules and will be provided by the sponsor.

5.4.1.2. Enzalutamide

Enzalutamide will be provided as soft gelatin capsules of 40 mg for oral administration.

For patients in Part 1, enzalutamide capsules were sourced by the site to the patient.

In Part 2, enzalutamide will be provided to the sites by the Sponsor, and dispensed from the sites to the patients. All supplies of these drugs will be used in accordance with local regulations.

5.4.2. Preparation and Dispensing

For Part 1, open-label talazoparib and enzalutamide will be dispensed manually (without the use of an IWRS system) every 4 weeks through Week 25 and every 12 weeks thereafter. A qualified staff member will dispense the investigational product in bottles provided, in quantities appropriate for the study visit schedule. The patient/caregiver should be instructed to maintain the product in the bottle provided throughout the course of dosing and return the bottle to the site at the next study visit.

090177e19c2fb240\Approved\Approved On: 23-Dec-2022 10:25 (GMT)

For Part 2, talazoparib or matching placebo and enzalutamide will be dispensed using an IWRS drug management system every 4 weeks through Week 53 and every 8-weeks thereafter. A qualified staff member will dispense the investigational product via unique container numbers in bottles provided, in quantities appropriate for the study visit schedule. The patient/caregiver should be instructed to maintain the product in the bottle provided throughout the course of dosing and return the bottle to the site at the next study visit.

Talazoparib is considered a cytotoxic and clastogenic agent; precautions regarding appropriate secure storage and handling must be used by healthcare professionals, including personal protective clothing, disposable gloves, and equipment. Patients should be advised that oral anticancer agents are toxic substances and that other caregivers (including family members) should always use gloves when handling the study treatment.

5.5. Administration

5.5.1. Talazoparib or Blinded Therapy

Talazoparib (or blinded therapy) will be self-administered by mouth outside the clinic, except on days when PK is assessed; patients will withhold their dose until after the pre-dose PK blood sample is drawn. For Part 1, patients initially received a starting dose of 1 mg once daily prior to reducing the dose to 0.5 mg/day based on results from a review of available safety and PK data (Section 1.6.1).

Talazoparib (or blinded therapy) should be administered with enzalutamide at approximately same time (morning of each day to ensure that PK collection is compliant per protocol). Switching to evening dosing, if necessary, is permitted after Week 13 and Week 17 visit, which is the last scheduled PK sample collection in Part 1 and Part 2 respectively.

The starting dose of blinded therapy for Part 2 will be 0.5 mg once daily, and will be administered as two 0.25 mg capsules. The starting dose for patients with moderate renal impairment (30 to 59 ml/min/1.73 m^2 by the MDRD equation per www.mdrd.com) (permitted to enroll only in Part 2) will be 0.35 mg once daily, and will be administered as one 0.25 mg capsule and one 0.1 mg capsule.

Talazoparib or talazoparib/placebo can be administered with or without food. Patients will swallow the investigational product whole with a large glass of water (~250 ml), and will not manipulate or chew, dissolve, or open the capsules prior to swallowing.

If a patient forgets his daily dose of talazoparib or talazoparib/placebo at the time he typically takes it, but remembers this on the same day, within 12 hours of the usual dose time, the dose may be taken at that time. Any dose that is missed (not taken within 12 hours of the intended time) should be skipped and should not be replaced or made up on the following day. Patients should not make up vomited doses; dosing should resume on the next calendar day unless otherwise instructed.

090177e19c2fb240\Approved\Approved On: 23-Dec-2022 10:25 (GMT)

5.5.1.1. Talazoparib Overdose

There is no specific treatment in the event of talazoparib overdose, and symptoms of overdose are not established. In the event of overdose, treatment with talazoparib or talazoparib/placebo should be stopped, and physicians should consider gastric decontamination, follow general supportive measures and treat symptomatically.

5.5.2. Enzalutamide

Enzalutamide will be administered by mouth as four 40-mg soft gelatin capsules once daily (160 mg per day). If a patient forgets his daily dose of enzalutamide at the time he typically takes it, but remembers this on the same day, within 12 hours of the usual dose time, the dose may be taken at that time. Any dose that is missed (not taken within 12 hours of the intended time) should be skipped and should not be replaced or made up on a subsequent day. Patients should not make up vomited doses; dosing should resume on the next calendar day unless otherwise instructed.

Enzalutamide can be administered with or without food. Enzalutamide should be administered with talazoparib or talazoparib/placebo at the same time each day (ie, morning of each day with switching to evening doses permitted after Week 13 (Part 1) and Week 17 visit (Part 2).

Patients will swallow the investigational product whole, and will not manipulate or chew, dissolve, or open the capsules prior to swallowing.

Reduction of the enzalutamide dose to 120 mg (3 capsules) or 80 mg (2 capsules) daily is permitted per the enzalutamide label.

Patients should be instructed to take study treatments with a large glass of water (~250 ml) at the same time each day.

5.6. Dose Modifications

5.6.1. Part 1: Talazoparib Dose Determination

The preliminary results summarizing the safety profile and PK data of talazoparib in combination with enzalutamide in Part 1 are discussed in Section 1.6.1. Briefly, enzalutamide increased talazoparib C_{trough} at Week 5 Visit by ~2-fold compared to talazoparib steady-state C_{trough} in patients with advanced solid tumors and in patients with breast cancer receiving 1 mg/day monotherapy. The increase in talazoparib exposure was associated with higher than expected incidence of Grade 3/4 hematological AEs. The results of Part 1 suggested that reducing the talazoparib dose to 0.5 mg QD in combination with enzalutamide is expected to account for the observed interaction and maintain similar talazoparib exposure to that achieved with 1 mg QD monotherapy with an acceptable safety profile. Therefore, a talazoparib dose of 0.5 mg QD will be administered in combination with enzalutamide 160 mg QD in Part 2 of this study.

See Section 1.6.1.1 for the summary of Part 1 safety and PK data.

090177e19c2fb240\Approved\Approved On: 23-Dec-2022 10:25 (GMT)

The Sponsor evaluated the emerging safety data including the occurrence of the listed target safety events defined in Table 6.

Table 6. Target Safety Events: Part 1 (Open-Label Treatment)

**Hematologic Toxicity**
Any of the following considered possibly or probably related to talazoparib:
• Grade 4 anemia;<br>• Grade 4 thrombocytopenia;<br>• Grade 4 neutropenia (ANC <500/μL or <0.5 × 10^9/L);<br>• Grade 3 thrombocytopenia associated with clinically significant bleeding;<br>• Grade 3 neutropenia or Grade 3 thrombocytopenia if daily dosing is interrupted for ≥7 days;<br>• Febrile neutropenia, defined as ANC <1000/μL with a single temperature of >38.3°C (>101°F) or a temperature of >38°C (100.4°F) sustained over a 1-hour period;<br>• Neutropenic infection (ANC <1000/μL or <1.0 × 10^9/L).
**Nonhematologic Toxicity**
Any Grade ≥3 adverse event considered possibly or probably related to talazoparib excluding the following:
• Grade ≥3 laboratory abnormalities not considered clinically significant;<br>• Grade ≥3 adverse event not considered clinically significant;<br>• Grade ≥3 nausea or vomiting that responds to medical intervention within 72 hours;<br>• Grade ≥3 diarrhea that can be medically managed to Grade ≤2 within 72 hours;<br>• Grade ≥3 fatigue that improves to Grade ≤2 within 7 days.
**Liver Toxicity**
Any of the following considered possibly or probably related to talazoparib:
• ALT or AST >3 × ULN if baseline ALT or AST ≤3 × ULN;<br>• ALT or AST >5 × ULN (a lower threshold should be considered if the ALT or AST abnormalities are accompanied by symptoms and signs of hepatitis) AND 2-fold increases above baseline;<br>• ALT or AST ≥3 × ULN and total bilirubin >2 × ULN;<br>• Total bilirubin >5 × ULN.

ANC = absolute neutrophil count, ALT = alanine aminotransferase, AST = aspartate aminotransferase, ULN = upper limit of normal.
Toxicities will be classified by severity according to the National Cancer Institute Common Terminology Criteria for Adverse Events, version 4.03.

5.6.2. Study Treatment Dose Modification

5.6.2.1. Dose Modifications for Talazoparib or Talazoparib/Placebo Due to Adverse Events

In Part 2, a 0.5 mg once daily dose of talazoparib (or placebo) will be administered in combination with enzalutamide. The daily dose of talazoparib/placebo may be reduced sequentially to 0.35 mg once daily, 0.25 mg once daily and 0.1 mg once daily to manage adverse events as described in Table 7. The starting dose of talazoparib/placebo for patients with moderate renal impairment per www.mdrd.com will be 0.35 mg once daily with potential for sequential dose reductions to 0.25 mg once daily and 0.1 mg once daily to manage toxicity (Table 7).

090177e19c2fb240\Approved\Approved On: 23-Dec-2022 10:25 (GMT)

Dose modifications for talazoparib (Part 1) and talazoparib/placebo (Part 2) due to adverse events are described in Table 7 and Table 8 below.

Table 7. Talazoparib or Talazoparib/Placebo Dose Modification Levels for Adverse Reactions

Dose Level	Dose	Administration of Dose
Starting dose	0.5 mg once daily	two 0.25 mg capsules once daily
First dose reduction[a]	0.35 mg once daily	one 0.25 mg capsule and one 0.1 mg capsule once daily
Second dose reduction	0.25 mg once daily	one 0.25 mg capsule once daily
Third dose reduction	0.1 mg once daily	one 0.1 mg capsule once daily

a. Starting dose for patients with moderate renal impairment (eGFR 30-59 mL/min/1.73 m^2 based on MDRD equation) at Screening. See Appendix 5.

Table 8. Dose Modification of Talazoparib or Talazoparib/Placebo Due to Adverse Events

Toxicity	Management of Adverse Events
Grade 1 or 2	No requirement for dose interruption or dose reduction.
Grade 1 or 2 anemia	If Grade 1 or 2 anemia is clinically symptomatic (eg fatigue), management per investigator may be warranted. Dose interruption is allowed for talazoparib/placebo for 4-8 weeks. However, patients should resume talazoparib/placebo as soon as symptoms that resulted in dose interruption have ceded to minimize treatment interruptions. If the talazoparib or talazoparib/placebo dose was reduced because of Grade 1 or 2 anemia, re-escalation by 1 dose level is allowed if symptoms improve.
Grade 3 or 4 Anemia (hemoglobin <8.0 g/dL)	Hold talazoparib or talazoparib/placebo and implement supportive care per local guidelines.<br><br>Monitor weekly until hemoglobin returns to ≥9 g/dL or better, then resume talazoparib or talazoparib/placebo at a reduced dose level. Dose re-escalation is NOT allowed once dose of talazoparib or talazoparib/placebo was reduced due to Grade 3 or 4 anemia.<br><br>If anemia persists for >8 weeks without recovery to at least 9.0 g/dL despite supportive care measures, discontinue talazoparib or talazoparib/placebo and refer to a hematologist for evaluation.<br><br>• Transfusions and erythropoiesis-stimulating agents are permitted to support management of hematological toxicities. (see Section 5.9).
Grade 3 or 4 Neutropenia (ANC <1000/µL)	Hold talazoparib or talazoparib/placebo and implement supportive care per local guidelines.<br><br>Monitor weekly until ANC ≥1500/µL, then resume talazoparib or talazoparib/placebo at a reduced dose level.<br><br>If neutropenia recurs after the dose reduction, hold talazoparib or talazoparib/placebo and implement supportive care per local guidelines.

090177e19c2fb240\Approved\Approved On: 23-Dec-2022 10:25 (GMT)

Table 8. Dose Modification of Talazoparib or Talazoparib/Placebo Due to Adverse Events

Toxicity	Management of Adverse Events
	Monitor weekly until ANC ≥1500/µL, then resume talazoparib or talazoparib/placebo at a further reduced dose.<br><br>If neutropenia persists for >4 weeks without recovery to ≥1500/µL at any dose level despite supportive care measures, discontinue talazoparib or talazoparib/placebo and refer to a hematologist for evaluation.<br><br>• G-CSF and GM-CSF may be used at investigators discretion for the supportive treatment of neutropenia (see Section 5.9).
Grade 3 or 4 Thrombocytopenia (platelets <50,000/µL)	Hold talazoparib or talazoparib/placebo and implement supportive care per local guidelines.<br><br>Monitor weekly until platelets ≥50,000/µL, and then resume talazoparib or talazoparib/placebo treatment at a reduced dose.<br><br>If thrombocytopenia (<50,000/µL) recurs after one dose reduction, hold talazoparib or talazoparib/placebo and implement supportive care per local guidelines. Monitor weekly until platelets ≥75,000/µL, then resume talazoparib or talazoparib/placebo at a further reduced dose.<br><br>If thrombocytopenia persists for >4 weeks without recovery to ≥75,000/µL despite supportive care measures, discontinue talazoparib or talazoparib/placebo and refer to a hematologist for evaluation.<br><br>• Thrombopoietin analogues and/or platelet transfusions may be used at investigators discretion for the supportive treatment of thrombocytopenia. (see Section 5.9).
Nonhematologic laboratory Grade ≥3 events, considered related to talazoparib or talazoparib/placebo, except abnormal liver tests	Hold talazoparib or talazoparib/placebo as follows:<br><br>For Grade 3 laboratory abnormalities, hold talazoparib or talazoparib/placebo until the laboratory abnormality resolves to Grade ≤2 (to baseline grade for creatinine increases). Resume talazoparib or talazoparib/placebo at the same dose or reduce by 1 dose level.<br><br>For Grade 4 laboratory abnormalities, hold talazoparib or talazoparib/placebo until the laboratory abnormality resolves to Grade ≤2 (to baseline grade for creatinine increases). Resume talazoparib or talazoparib/placebo at a 1 dose level reduction.<br><br>Implement supportive care per local guidelines.<br><br>Contact the sponsor to discuss potential dose modification.<br><br>Talazoparib or talazoparib/placebo must be permanently discontinued for unresolved Grade 3 toxicity lasting longer than 14 days or for Grade 4 toxicity lasting longer than 3 days. Treatment may be resumed at a 1 dose level reduction if clear clinical benefit is observed, after discussion with the sponsor.
Grade ≥3 abnormal liver tests	Hold talazoparib or talazoparib/placebo for liver test abnormalities as specified in Section 5.6.3. Guidelines for follow-up for possible drug-induced liver injury and for resuming talazoparib or talazoparib/placebo after the liver test abnormalities resolve to baseline grade are provided in Section 5.6.3.

090177e19c2fb240\Approved\Approved On: 23-Dec-2022 10:25 (GMT)

Table 8. Dose Modification of Talazoparib or Talazoparib/Placebo Due to Adverse Events

Toxicity	Management of Adverse Events
	The criteria for permanent discontinuation talazoparib or talazoparib/placebo are provided in Section 5.6.3.
Nonlaboratory Grade ≥3 events, considered related to talazoparib or talazoparib/placebo	Hold talazoparib or talazoparib/placebo as follows:<br>For Grade 3 adverse events, hold talazoparib or talazoparib/placebo until the adverse event resolves to Grade ≤1 or baseline. Resume talazoparib or talazoparib/placebo at the same dose or reduce by 1 dose level.<br>For Grade 4 adverse events, permanently discontinue talazoparib or talazoparib/placebo except for the following:<br>• Vomiting that responds to medical intervention within 3 days;<br>• Diarrhea that is medically managed to Grade ≤2 within 3 days;<br>• Constipation that is medically managed to Grade ≤2 within 7 days.<br>Resume talazoparib or talazoparib/placebo at a 1 dose level reduction.<br>Implement supportive care per local guidelines.

For non-hematologic events, not related to study treatment, talazoparib or talazoparib/placebo dose re-escalation may be allowed after the reduced dose is tolerated without recurrence of toxicities, and only after investigator discussion with the sponsor.

5.6.2.2. Dose Modifications for Enzalutamide Due to Adverse Events

Dose modifications of enzalutamide are to occur according to the local label. If talazoparib or talazoparib/placebo is permanently discontinued, enzalutamide may continue. Scheduled PK samples for enzalutamide should be collected if talazoparib or talazoparib/placebo is discontinued as shown in Table 2.

5.6.2.3. Talazoparib or Talazoparib/Placebo Dose Escalation Only if Enzalutamide Permanently Discontinued

If enzalutamide treatment is permanently discontinued while continuing talazoparib or talazoparib/placebo treatment, the talazoparib or talazoparib/placebo dose at the time of enzalutamide discontinuation may be escalated after 6 weeks of enzalutamide discontinuation as illustrated in Table 9 based on patient's safety and tolerability and only after investigator discussion with the sponsor. Should this decision be made, the escalated talazoparib or talazoparib/placebo dose will be dispensed at an unplanned study visit. Scheduled PK samples for talazoparib or talazoparib/placebo should be collected if enzalutamide is discontinued as shown in Table 2.

090177e19c2fb240\Approved\Approved On: 23-Dec-2022 10:25 (GMT)

Table 9. Talazoparib or Talazoparib/Placebo Dose Escalation After 6 Weeks of Enzalutamide Discontinuation[a]

Talazoparib or Talazoparib/Placebo Dose At the time of Enzalutamide Discontinuation (mg once daily)	Talazoparib or Talazoparib/Placebo Dose Levels After 6 Weeks From Enzalutamide Discontinuation (mg once daily)
0.5	1
0.35	0.75
0.25	0.5
0.1	0.2

a. After escalation, talazoparib or talazoparib/placebo dose can be reduced in 0.25 mg decrements to manage AEs (refer to Table 7 and Table 8). Patients on 0.2 mg can dose reduce to 0.1 mg.

5.6.3. Modifications Due to Abnormal Liver Tests (All Treatments)

Both talazoparib or talazoparib/placebo and enzalutamide should be withheld if liver enzymes abnormalities are reported. Patients who develop abnormal liver tests (AST, ALT, total bilirubin [TBili]), abnormal international normalized ratio (INR) values, or signs or symptoms consistent with hepatitis during study treatment may meet the criteria for temporarily withholding or permanently discontinuing study treatment talazoparib (or placebo) and enzalutamide. Patients who have abnormal liver tests or meet the criteria for permanent discontinuation or temporary withholding of study treatment will be followed up according to the recommendations in this section.

Both study treatments should be withheld for any liver test abnormality listed in Table 10. When withholding study treatments, follow-up should continue for possible drug-induced liver injury (Section 8.4.2) until the liver test abnormalities resolve to baseline grade.

Table 10. Criteria for Temporary Withholding of Study Treatment in Association With Liver Test Abnormalities

Baseline[a] AST or ALT Value	Elevation[b]
≤3 × ULN	• >5 × ULN<br>• ALT or AST ≥3 × ULN with the presence of signs and symptoms consistent with acute hepatitis and/or eosinophilia (≥500 eosinophils/µL)
>3 × ULN and ≤5 × ULN	>8 × ULN
**Baseline[a] Total Bilirubin Value**	
≤1.5 × ULN	>3 × ULN
>1.5 × ULN and ≤3 × ULN (Patients with Gilbert syndrome or for whom indirect bilirubin concentrations suggest an extrahepatic source of elevation)	>5 × ULN

For rechallenge, dose modification may be required per Table 7.
ALT = alanine aminotransferase; AST = aspartate aminotransferase; ULN = upper limit of normal.
a. Baseline value is the laboratory value obtained at Day 1 prior to dosing.
b. Talazoparib or talazoparib/placebo should be permanently discontinued if any elevation related to study treatments persists for more than 7 days or AST/ALT >20 × ULN persists for longer than 3 days.

090177e19c2fb240\Approved\Approved On: 23-Dec-2022 10:25 (GMT)

Rechallenge with enzalutamide may only be considered according to local label. Rechallenge with talazoparib or talazoparib/placebo following liver test abnormalities is not permitted if these abnormalities are accompanied with signs or symptoms consistent with drug-induced liver toxicity or meet permanent discontinuation criteria. Rechallenge with talazoparib (or identical placebo) may be considered at 1 reduced dose level (see Table 7) if an alternative cause for the abnormal liver tests (ALT, AST, Tbili) is discovered and the laboratory abnormalities resolve to normal or baseline values. The investigator and sponsor should discuss and agree with any decision to rechallenge any of the assigned study treatments. Following rechallenge, patients should be closely monitored for signs and symptoms of hepatitis and/or abnormal liver test results.

Study treatment should be permanently discontinued permanently if any of the following criteria are met and no alternative cause explains the laboratory abnormalities:

- Patients with AST/ALT and Tbili baseline values within the normal range who subsequently present with AST **OR** ALT values >3 × ULN **AND** a Tbili value >2 × ULN with no evidence of hemolysis and an alkaline phosphatase value <2 × ULN or not available (Note: in the presence of elevated alkaline phosphatase associated with bone metastases, gamma glutamyl transferase [GGT] should be tested and the results should be within the reference range);

- For subjects with baseline AST **OR** ALT **OR** Tbili values above the ULN, the following threshold values are used in the definition mentioned above, as needed, depending on which values are above the ULN at baseline:

  - Preexisting AST or ALT baseline values above the normal range: AST or ALT values >2 times the baseline values AND >3 × ULN; or >8 × ULN (whichever is smaller).

  - Preexisting values of Tbili above the normal range: Tbili level increased from baseline value by an amount of at least 1 × ULN **or** if the value reaches >3 × ULN (whichever is smaller).

- Patients with AST/ALT >5 × ULN that persists for more than 7 days (AST/ALT >8 × ULN for patients with hepatic involvement).

- Patients with AST/ALT >20 × ULN that persists for longer than 3 days.

- Patients with Tbili >3 × ULN that persists for longer than 7 days (>5 × ULN for patients with Gilbert's disease).

When study treatment is temporarily withheld or permanently discontinued due to a potential drug induced liver injury, a period of close observation is to commence until the liver test abnormalities return to baseline or normal values. The evaluations listed in Table 11 should be performed.

090177e19c2fb240\Approved\Approved On: 23-Dec-2022 10:25 (GMT)

Table 11. Monitoring of Liver Tests for Potential Drug-Induced Liver Injury

Results	Frequency for Repeating Liver (AST, ALT, Bilirubin [Total and Direct]) and INR Tests
After the initial liver test abnormality	Preferably within 48 hours (See Section 8.4.2).
If AST or ALT ≥3 × ULN (>5 × ULN if baseline ALT/AST is >3 × ULN), and total bilirubin >2 × ULN or INR >1.5	Every 24 hours until laboratory abnormalities improve.
If ALT or AST ≥3 × ULN (>5 × ULN if baseline ALT/AST is >3 × ULN) and total bilirubin or INR are normal	Every 48 to 72 hours until laboratory abnormalities improve.
If the liver test abnormalities improve AND the patient is asymptomatic	Frequency may decrease.

ALT = alanine aminotransferase; AST = aspartate aminotransferase; INR = international normalized ratio; ULN = upper limit of normal.

As drug induced liver injury is a diagnosis of exclusion, it is important to initiate investigation of alternative causes for abnormal liver tests, which may include consultation with a hepatologist. Contact the sponsor with questions regarding sufficient follow-up tests.

5.6.4. End of Treatment

Patients are to continue study treatment until radiographic progression is determined (by local review in Part 1; by BICR in Part 2) unless in the opinion of the investigator the patient is still deriving benefit at this time, or following radiographic progression the patient is no longer clinically benefitting in the opinion of the investigator (see Section 3), or an adverse event leading to permanent discontinuation, or patient decision to discontinue study treatment, or death.

5.7. Investigational Product Storage

The investigator or an approved representative, eg, pharmacist, will ensure that talazoparib (or placebo) and enzalutamide are stored in a secured area with controlled access under required storage conditions and in accordance with applicable regulatory requirements.

Investigational products should be stored in their original containers and in accordance with the labels.

Any storage conditions stated in the talazoparib Investigator's Brochure and the Summary of Product Characteristics for enzalutamide will be superseded by the storage conditions stated on the product label.

090177e19c2fb240\Approved\Approved On: 23-Dec-2022 10:25 (GMT)

Site systems must be capable of measuring and documenting (for example, via a log), at a minimum, daily minimum and maximum temperatures for all site storage locations (as applicable, including frozen, refrigerated, and/or room-temperature products). This should be captured from the time of investigational product receipt throughout the study. Even for continuous-monitoring systems, a log or site procedure that ensures active evaluation for excursions should be available. The intent is to ensure that the minimum and maximum temperature is checked each business day to confirm that no excursion occurred since the last evaluation and to provide the site with the capability to store or view the minimum/maximum temperature for all non-working days upon return to normal operations. The operation of the temperature monitoring device and storage unit (for example, refrigerator), as applicable, should be regularly inspected to ensure they are maintained in working order.

Any excursions from the product label storage conditions should be reported to Pfizer upon discovery. The site should actively pursue options for returning the product to the storage conditions described in the labeling, as soon as possible. Deviations from the storage requirements, including any actions taken, must be documented and reported to Pfizer.

Once an excursion is identified, the investigational product must be quarantined and not used until Pfizer provides permission to use the investigational product. It will not be considered a protocol deviation if Pfizer approves the use of the investigational product after the temperature excursion. Use of the investigational product prior to Pfizer approval will be considered a protocol deviation. Specific details regarding information the site should report for each excursion will be provided to the site.

Receipt of materials, door opening and closing, and other routine handling operations where the products are briefly out of the temperature range described in the labeling are not considered excursions.

Site staff will instruct patients on the proper storage requirements for take home investigational products.

Refer to the Investigational Product Manual for additional guidance on storage conditions and actions to be taken when conditions are outside of the prespecified range.

5.8. Investigational Product Accountability

The investigator site must maintain adequate records documenting the receipt, use, loss, or other disposition of the investigational product supplies. All investigational products will be accounted for using a drug accountability form/record.

All bottles of study treatment must be returned to the investigator by the patient at every visit and at the end-of-treatment. Patients who do not return bottles at the end-of-treatment will be reminded to bring them at their next visit.

090177e19c2fb240\Approved\Approved On: 23-Dec-2022 10:25 (GMT)

5.8.1. Destruction of Investigational Product Supplies

The sponsor or designee will provide guidance on the destruction of unused investigational product (eg, at the site). If destruction is authorized to take place at the investigator site, the investigator must ensure that the materials are destroyed in compliance with applicable environmental regulations, institutional policy, and any special instructions provided by Pfizer, and all destruction must be adequately documented.

5.9. Concomitant Treatment(s)

Use of the following treatments is prohibited during the study:

- Prednisone >10 mg (or equivalent) once daily (Note: short term use [<= 4 weeks] of higher doses of steroids although discouraged is permitted if no alternative therapy is available).

- Cytotoxic chemotherapy (eg, platinum based chemotherapy, cyclophosphamide, taxanes, or mitoxantrone chemotherapy) for metastatic prostate cancer.

- Hormonal therapy (eg, bicalutamide, nilutamide, flutamide, estrogens, 5-alpha reductase inhibitors), NHT with the exception of enzalutamide (eg, abiraterone, apalutamide, darolutamide), biologic therapy, or radionuclide therapy for prostate cancer or any other investigational agent.

- Another PARP inhibitor.

Use of the following treatments is permitted during the study as below:

- Treatment with bisphosphonates or denosumab during the study are permitted.

- If patient met eligibility requirements of medical castration, ongoing androgen deprivation therapy with a GnRH agonist or antagonist is required to continue throughout the study.

- Hematopoietic growth factors (eg, granulocyte colony stimulating factor [G CSF], granulocyte macrophage colony stimulating factor [GM CSF]): Primary prophylactic use of granulocyte colony stimulating factors is not permitted but they may be used to treat treatment emergent neutropenia as indicated by the current American Society of Clinical Oncology (ASCO) guideline. If neutropenic complications are observed in a cycle in which primary prophylaxis with colony stimulating factors (CSFs) was not received, secondary prophylaxis may be given at the discretion of the investigator, but only if dose reduction or delay are not considered a reasonable alternative.

- Red blood cell (RBC) transfusions, erythropoietin and erythropoiesis-stimulating agents may be used at the investigator's discretion for the supportive treatment of anemia.

090177e19c2fb240\Approved\Approved On: 23-Dec-2022 10:25 (GMT)

- Thrombopoietin analogues and/or platelet transfusions may be used at investigator's discretion for the supportive treatment of thrombocytopenia.

- COVID-19 vaccines are allowed. The timing of the vaccination relative to the administration of study treatments is at the discretion of the investigator.

The start date, stop date, and indication for concomitant treatments and/or therapies and medications received from screening until safety follow visit will be recorded on the CRF.

In Part 2, patients will use an analgesic log to document their use of analgesics for prostate cancer pain during each of the 7 consecutive days before a scheduled visit (or scheduled contact during the post-treatment follow-up periods).

Analgesics (including opioids) will be collected on the concomitant medication CRF page where start and stop date, indication, dose and route is recorded from screening until end of treatment.

Patients must also not take any of the following concomitant medications during the study:

- Prohibited medication due to potential DDI with enzalutamide (also to be considered as an exclusion criteria for enrollment if used within 7 days prior to Day 1 in Part 1 or randomization in Part 2): strong cytochrome P450 2C8 (CYP2C8) inhibitors (eg, clopidogrel, gemfibrozil) and inducers (eg, rifampin), strong CYP3A4 inducers (eg, carbamazepine, phenobarbital, phenytoin, rifabutin, rifampin, rifapentine and St. John's Wort), moderate CYP3A4 inducers (eg, bosentan, efavirenz, etravirine, modafinil and nafcillin), or substrates of CYP3A4 (eg, alfentanil, cyclosporine, dihydroergotamine, ergotamine, fentanyl, pimozide, quinidine, sirolimus and tacrolimus), CYP2C9 (eg, phenytoin, warfarin), or CYP2C19 (eg, S-mephenytoin) with a narrow therapeutic index unless considered medically necessary to treat a life-threatening condition. If the use of warfarin cannot be avoided and is clinically necessary, caution and additional international normalized ratio (INR) monitoring is recommended. Patients treated with strong cytochrome P450 2C8 (CYP2C8) inhibitors (eg, clopidogrel, gemfibrozil) within 7 days from randomization are not eligible; if administration cannot be avoided during treatment, the dose of enzalutamide must be reduced to 80 mg once daily.

- Rivaroxaban and apixaban (both are direct oral anticoagulants and are substrates of CYP3A4 and P-gp) should be avoided as enzalutamide might result in reduced plasma exposure which may lead to loss of efficacy or bleeding complications. Switch to anticoagulants allowed per local enzalutamide prescribing information (if warfarin is used apply additional monitoring as stated above). Dabigatran and edoxaban (direct oral anticoagulants that are substrates for P-gp but not for CYP3A4) should be used with caution given the unknown clinical effect of enzalutamide on P-gp inhibition; additional monitoring should be conducted as warranted.

090177e19c2fb240\Approved\Approved On: 23-Dec-2022 10:25 (GMT)

- Prohibited medications due to potential DDI with talazoparib (also to be considered as an exclusion criteria for enrollment if used within 7 days prior to Day 1 in Part 1 or randomization in Part 2): Potent P-gp inhibitors that result in ≥2-fold increase in the exposure of an in vivo probe P-gp substrate according to the FDA website[55] and University of Washington Drug-Drug Interaction database[56] are not recommended: amiodarone, carvedilol, clarithromycin, cobicistat, dronedarone, erythromycin, glecaprevir/pibrentasvir, indinavir, itraconazole, ketoconazole, lapatinib, lopinavir, propafenone, quinidine, ranolazine, ritonavir, saquinavir, sofosbuvir/velpatasvir/voxilaprevir, telaprevir, tipranavir, valspodar and verapamil. Alternative therapies should be considered whenever possible. If usage of any of the above treatment is deemed medically necessary, consultation and agreement with the Sponsor is required prior to treatment initiation as appropriate dose modification of talazoparib may be needed.

- Caution and monitoring for potential increased adverse reactions should be used upon concomitant use of the following transporter inhibitors with talazoparib: atorvastatin, azithromycin, conivaptan, curcumin, cyclosporine, diltiazem, diosmin, elacridar [GF120918], eliglustat, eltrombopag, felodipine, flibanserin, fluvoxamine, piperine, quercetin, and schisandra chinensis extract.

Concomitant anticancer therapy is prohibited during the study. Recording of prior and post-study cancer therapies including start and stop dates, and response to treatment, will be recorded in the CRF.

5.10. Rescue/Salvage Medication

New treatments for prostate cancer prior to the determination of radiographic progression are strongly discouraged.

6. STUDY PROCEDURES

6.1. Prescreening (optional)

For prescreening procedures, see Table 1.

Prescreening is optional and may be performed at any time before the primary consent form is signed. Patients who undergo prescreening will sign and date a prescreening consent form different from the main consent form signed at the screening visit. Preferably a blood sample and tumor tissue (or historical results) will all be submitted at this time. During this period, the blood sample will be analyzed at Foundation Medicine (FoundationOne® Liquid test) to determine whether patients meet eligibility criteria (historical liquid biopsy results are not acceptable) and concordance assessment. De novo or archival tumor tissue (preferably of metastatic disease, keeping in mind bone biopsies have high percentage of technical failure while lymph nodes have higher probability of success) will be also submitted for eligibility/stratification and concordance assessment with liquid biopsy results. Patients who have prior FoundationOne® test results, based on tumor tissue, can submit their report indicating DDR gene status for evaluation by sponsor for eligibility consideration with Sponsor pre-approval. If historical testing results will be used for eligibility and any tumor

090177e19c2fb240\Approved\Approved On: 23-Dec-2022 10:25 (GMT)

tissue/DNA is still available at Foundation Medicine, the remaining tumor tissue/DNA may be used to confirm historical results. Although results from either the liquid or tumor tissue are required for eligibility and stratification in IWRS, both blood and tumor tissue sample must be submitted prior to randomization. Disease progression (eg, preliminary PSA increase) should be observed prior to submitting blood or tumor tissue samples for analysis to Foundation Medicine. Demography, AE and research related injuries (see Section 8.1.5) will also be collected at this visit.

6.2. Screening

For screening procedures in Part 1 and Part 2, see Table 1. All patients will sign and date an informed consent form prior to undergoing any study-specific procedures. Screening activities may be repeated any time as clinically indicated. Genomic testing will not be repeated if the patient was found genomically eligible by the central tissue lab during prescreening.

If the patient did not participate in a prescreening visit, the blood sample and de novo or archival tumor tissue (preferably of metastatic disease) will be analyzed at Foundation Medicine (FoundationOne® Liquid test or FoundationOne® test) to determine whether patients meet eligibility criteria. Patients who have prior FoundationOne® test results can submit their report indicating DDR gene status for evaluation by sponsor for eligibility consideration with Sponsor pre approval. If historical testing results will be used for eligibility and any tumor tissue/DNA is still available at Foundation Medicine, the remaining material may be used to confirm historical results. Results from any one of these tests will be needed for eligibility and stratification. If results from liquid and tumor tissue sample are available prior to randomization and results are not concordant for the presence of the selected DDR mutation(s), positive DDR results will be used for eligibility and stratification regardless of the tissue used to generate the data.

If not submitted during prescreening, a de novo or archival tumor tissue (preferably of metastatic disease) or a liquid biopsy will be also submitted for retrospective testing and exploratory concordance studies. Tumor tissue may not be needed for patients with historical FoundationOne® results approved by the Sponsor.

Screening procedures are to be performed in the 28 days before enrollment (Part 1) or randomization (Part 2). However, radiographic scans obtained in the 42 days preceding enrollment (Part 1) or randomization (Part 2) are acceptable. Assessments obtained before the patient signs the informed consent form can be used to determine eligibility if obtained as part of standard of care and were performed per the specific study requirements.

Laboratory evaluations to be done at Screening include hematology and serum chemistry, testosterone, PSA, and in Part 2 include CTCs, and ctDNA. In South Africa only: Optional HIV testing using the ELISA test may be conducted for subjects of unknown HIV status per investigator discretion. Subjects will be asked to sign an HIV consent form and be given pre and post test counseling at the site.

090177e19c2fb240\Approved\Approved On: 23-Dec-2022 10:25 (GMT)

Data collected will be recorded in CRF.

Histological characterization of prostate cancer will not be performed at Foundation Medicine, but must be provided by the site. At the time of screening, the patient must provide the answer to question #3 of the BPI-SF to assess eligibility, and total testosterone must be measured to confirm that the patient is surgically or medically castrated. The patient must have M1CRPC. M1 disease means the cancer has to spread to the bone, distant lymph nodes (eg, aortic, common iliac, inguinal, supraclavicular, scalene, cervical lymph nodes) or distant organs. Patients with evidence of disease spread to only regional lymph nodes (eg, pelvic, internal iliac [hypogastric], obturator, sacral) are not eligible. Metastatic soft tissue disease must be seen on either CT scan (preferred) or MRI while bone lesions must be seen on bone scan (no other imaging modalities are allowed to assess bone lesions). The last PSA value collected at screening prior to randomization must be at least 1 ng/ml. Patients may have received abiraterone and taxane in the castration sensitive settings. Abiraterone must be discontinued prior to randomization while docetaxel must be discontinued 28 days prior to randomization. Abiraterone or taxanes or any other prostate cancer therapy (including sipuleucel-T) received in the CRPC settings (metastatic or non-metastatic) will be considered exclusionary. Only prior treatment with first generation antiandrogens (eg, bicalutamide, nilutamide, flutamide) in conjunction with ADT or old hormonal therapies (eg, estrogen, cyproterone acetate) are allowed in the metastatic or non-metastatic CRPC setting if discontinued prior to randomization. Prior treatment with enzalutamide, darolutamide, apalutamide is not allowed.

Investigator is to assess if the patient is biologically capable of fathering a child or exposing a fetus via ejaculate. If applicable, the investigator or designee will inform the subject of the need to use highly effective contraception consistently and correctly and document the conversation and the subject's affirmation in the subject's chart (subjects need to affirm their consistent and correct use of at least 1 of the selected methods of contraception). If applicable, sperm donation and fetus/partner exposure should also be discussed as per Schedule of Activities. In addition, the investigator or designee will instruct the subject to call immediately if the selected contraception method is discontinued or if pregnancy or fetus/partner exposure is known or suspected.

6.3. Treatment Period (Part 1 and Part 2)

For procedures/assessments, see Table 2.

Study visits during the treatment period will occur every 2 weeks through Week 17, every 4 weeks through Week 53, and every 8 weeks after Week 53. In Part 2 patients will document their prostate cancer pain symptoms and their use of analgesics for prostate cancer pain by completing both an electronic pain log and an electronic analgesic log during each of the 7 consecutive days preceding a scheduled visit as indicated on Schedule of Activities.

Patient-reported outcomes assessments will be conducted in Part 2 at baseline (Day 1) and are then to occur at scheduled study visits as follows: every 4 weeks through Week 53 or until determination of radiographic progression whichever is earlier; every 8 weeks after Week 53 until determination of radiographic progression when no such progression has been

090177e19c2fb240\Approved\Approved On: 23-Dec-2022 10:25 (GMT)

previously documented; every 12 weeks after radiographic progression until the end of the study.

Blood samples will be collected for various assessments on Day 1, and at the following time points thereafter per the Schedule of Activities (SOA):

- Hematology, serum chemistry, reticulocyte count, erythropoietin, serum folate and B12: every 2 weeks through Week 17, then every 4 weeks through Week 53, and then every 8 weeks thereafter. Investigators may collect samples for laboratory assessment up to 3 days prior to a scheduled visit to guide decision making. Day 1 only: sample to be stored for reflex testing for hepatitis B virus (HBV) (hepatitis B surface antigen [HbsAg], and anti-HBc), hepatitis C virus (HCV), hepatitis C antibody (HCV Ab), and reflex testing for HCV RNA if positive. Tests on this sample will be done if liver toxicities occur during treatment period.

- PSA assessments: every 8 weeks until determination of radiographic progression. Upon PSA progression or response, confirmation by a second consecutive value at least 21 days later is required. (An additional PSA is to be collected at the Week 53 visit, then to continue every 8 weeks thereafter thus aligning the PSA and site visits to be every 8 weeks).

- PK assessments:

  - Part 1: Collect blood samples for PK pre-dose, 1, 2, 4, 6 and 24 hours post-dose at Day 1 (Week 1) and Week 9. Collect blood samples for PK pre-dose and 2 hours post-dose at Week 5 and pre-dose at Week 13.

  - Part 2: Collect blood samples for PK pre-dose and 2 hours post-dose at Weeks 3, 5, and 9, and pre-dose at Weeks 13 and 17.

  - All patients: Additional PK should be taken based on discretion of investigators, eg, adverse events.

  - Record the dose amounts and time of dose administration on both the day of and the day before PK sampling. Record the date and time of each PK sample.

- Saliva sample for DNA sequencing (optional for Part 1) will be collected on Day 1.

- Samples for CTC analysis, circulating tumor DNA (ctDNA), and protein biomarkers: at Day 1, Weeks 9, 17, 25, and then at safety follow-up visit. Samples will be sent to a central laboratory as detailed in the Central Laboratory Manual.

See Table 2 Schedule of Activities for details.

Radiographic scans (CT of chest, CT/MRI of abdomen and pelvis, and whole body bone scan) will be conducted every 8 weeks through Week 25, then every 12 weeks thereafter until progression is confirmed by the investigator (Part 1) or an independent blinded reviewer and

090177e19c2fb240\Approved\Approved On: 23-Dec-2022 10:25 (GMT)

investigator (Part 2). For Part 2 scans will be submitted electronically for BICR review and results will be communicated back to the site only if the request for progressive disease verification will be submitted to the vendor (see the imaging manual). If radiographic progression is documented (by the BICR and investigator for Part 2 and investigator for Part 1) and the patient continues on study treatment as the patient is clinically benefitting per investigator, all radiographic imaging is to be continued per standard of care at the institution.

Any diagnosis of myelodysplastic syndrome (MDS) or AML for any patient enrolled/randomized will be reported as an SAE (Section 8.2.3).

If disease progression is suspected, additional radiographic scans may be performed, and additional PSA may be collected. These may be performed at an unscheduled visit as appropriate.

6.4. Safety Follow-up

For safety follow-up procedures after Part 1 and Part 2, see Table 2. Safety follow-up will occur approximately 28 calendar days after the last dose of study treatment (talazoparib, or talazoparib/placebo, or enzalutamide) or before initiation of a new antineoplastic therapy or investigational therapy (whichever occurs first). Contact with the patient for AE follow-up may be done via phone call if the patient does not come to the site.

6.5. Long-Term Follow-up

For long-term follow-up procedures after Part 1 and Part 2, see Table 3. This period begins after safety follow-up and can be conducted by telephone unless imaging is required.

Survival status can be obtained by any means including telephone, during a visit, chart review, or contact with someone who is knowledgeable of the patient's survival status (eg, relative, friend, referring healthcare provider). If allowed by local laws and regulations, survival status may be obtained from public records for patients withdrawing consent from the study or for patients lost to follow-up.

Patients who have not had a symptomatic skeletal event will continue to be assessed for the onset of such an event.

Patients will continue to complete the BPI-SF, EORTC QLQ-C30 and QLQ PR25, EQ 5D 5L, Pain Log, and Analgesic Log prior or during any scheduled visit as per Table 3. During Long Term Follow up when the visits may be conducted by telephone (if imaging is not being performed due to radiographic progression being met) these assessments should also be completed remotely from home as per the long term visit schedule.

Patients will be followed for subsequent antineoplastic or investigational therapy treatments and for diagnosis of myelodysplastic syndrome or acute myeloid leukemia.

090177e19c2fb240\Approved\Approved On: 23-Dec-2022 10:25 (GMT)

Patients will be followed for radiographic progression (Part 1 and 2) and PSA assessments (Part 2 only) per the radiographic imaging schedule if radiographic progression was not determined prior to discontinuation of study treatments.

6.6. Patient Withdrawal

Patients may withdraw from the study at any time at their own request, or they may be withdrawn at any time at the discretion of the investigator or sponsor for safety (see also the Withdrawal From the Study Due to Adverse Events section) or behavioral reasons, or the inability of the patient to comply with the protocol-required schedule of study visits or procedures at a given study site.

There will be 3 forms of voluntary withdrawal from the study: withdrawal from further receipt of investigational product, withdrawal from further study procedures, and withdrawal from any post-treatment follow-up. Consent withdrawal should only be obtained if withdrawing from any follow-up including overall survival. These will be entered on the appropriate CRF page. Patients who request to discontinue all study treatment will be asked to remain in the study and continue to be followed for protocol specified follow-up procedures. Patients should notify the investigator in writing of the decision to withdraw consent from future follow-up, whenever possible. The withdrawal of consent should be explained in detail in the medical records by the investigator. In the event that vital status (whether the patient is alive or dead) is being measured, publicly available information should be used to determine vital status only as appropriately directed in accordance with local law.

If a patient does not return for a scheduled visit, every effort should be made to contact the patient. This includes follow-up with persons authorized by the patient. "Lost to follow-up" is defined by the inability to reach the patient after a minimum of 2 documented phone calls, faxes, or e-mails as well as lack of response by the patient to 1 registered mail letter. All attempts to contact the patient and information received during contact attempts must be documented in the patient's medical record and CRF. If it is determined that the patient has died, the site will use locally permissible methods to obtain the date and cause of death. If the investigator's use of a third-party representative to assist in the follow-up portion of the study has been included in the patient's informed consent, then the investigator may use a sponsor-retained third-party representative to assist site staff with obtaining the patient's contact information or other public vital status data necessary to complete the follow-up portion of the study. The site staff and representative will consult publicly available sources, such as public health registries and databases, in order to obtain updated contact information. If, after all attempts, the patient remains lost to follow-up, then the last contact date should be reported and documented in the patient's medical records and CRF.

In any circumstance, every effort should be made to document patient outcome, if possible. The investigator should inquire about the reason for withdrawal, request that the patient return all unused investigational product(s), request that the patient return for a final visit, if applicable, and follow up with the patient regarding any unresolved AEs. If applicable, patients who return for a final visit will undergo safety follow-up procedures as shown in Table 2 and Table 3.

090177e19c2fb240\Approved\Approved On: 23-Dec-2022 10:25 (GMT)

If the patient withdraws from the study and also withdraws consent for the disclosure of future information, no further evaluations should be performed, and no additional data should be collected. The sponsor may retain and continue to use any data collected before such withdrawal of consent.

7. ASSESSMENTS

Every effort should be made to ensure that the protocol-required tests and procedures are completed as described. However, it is anticipated that from time to time there may be circumstances outside of the control of the investigator that may make it unfeasible to perform the test. In these cases the investigator will take all steps necessary to ensure the safety and well-being of the patient. When a protocol-required test cannot be performed, the investigator will document the reason for this and any corrective and preventive actions that he or she has taken to ensure that normal processes are adhered to as soon as possible. The study team will be informed of these incidents in a timely manner.

For samples being collected and shipped, detailed collection, processing, storage, and shipment instructions and contact information will be provided to the investigator site prior to initiation of the study.

7.1. Companion Diagnostics for Eligibility

Patients enrolling in Part 2 are required to have their disease assessed for a genomic mutation in one or more pre-defined DDR genes based on testing a blood sample or de novo or archival tumor tissue using the FoundationOne® tests. Testing may be performed at prescreening (optional) or screening. Results from testing of tumor tissue with the FoundationOne® test may be obtained from results of previously performed FoundationOne® testing, with Sponsor pre-approval. In cases where historical FoundationOne® test results are approved by the Sponsor, tumor tissue is not required for the study, but may be requested by the Sponsor if available. Assessment of genomic deficiency in DNA damage repair genes is optional for patients enrolling in Part 1.

DDR gene deficiencies will be identified via an NGS gene panel using blood, or de novo or archival tumor tissue collected at prescreening (optional) or screening. The panel evaluates genomic defects in genes involved in DDR associated with hereditary cancer syndromes and somatic genomic alterations (mutations, insertions/deletions, and copy number variants) in tumor DNA likely to respond to talazoparib (refer to the Laboratory Manual for specific information on the genes included in the biomarker panel). The FoundationOne® NGS gene panel test used to determine DDR gene status in tumor tissue samples will be performed by a single Certified Authorization Professional/Clinical Laboratory Improvement Act (CAP/CLIA)-certified central clinical laboratory site (Foundation Medicine), although results may be obtained from either study-sponsored prospective testing or from prior (ie, historic), non-study sponsored testing previously performed by Foundation Medicine.

090177e19c2fb240\Approved\Approved On: 23-Dec-2022 10:25 (GMT)

Blood and tumor tissue samples will be sent to Foundation Medicine for analysis with results returned within 2 weeks (longer time is required in case of technical issues). If blood and tumor tissue are not submitted at the same time, patients will be stratified according to the results obtained from the analysis of either the liquid or tumor tissue biopsy, depending on which test result is available. All patients will have to submit a blood sample for ctDNA analysis. All patients will have to submit a tumor tissue sample unless randomized using historical results approved by the Sponsor. If the test results are inconclusive or no result could be obtained due for example to technical reasons, the investigator may submit the alternative sample (tumor tissue or blood) for testing as well. In the case of both tests fail due to not meeting specified quality control metrics, or insufficient or inadequate tissue sample, the patient DDR mutational status will be considered unknown. If results from liquid and tumor tissue sample are available prior to randomization and results are not concordant for the presence of the selected DDR mutation(s), positive DDR results will be used for eligibility and stratification regardless of the tissue used to generate the data. See Section 7.5.1 for collection of these samples in China.

If the blood and tumor tissue are not submitted at the same time and results required for eligibility and stratification are already available, the sample (tumor tissue or liquid biopsy) submitted at a later point (but prior to randomization) will be analyzed for exploratory concordance analyses and to help support development of potential diagnostic tests for talazoparib for use in this and future clinical trials.

Patient mutational status will be considered positive if homozygous deletions, putative deleterious genomic alterations, or both are detected in genes in the panel. Neither single-copy somatic deletions without mutations detected in the second allele nor mono-allelic loss will be considered positive.

Alternately the patient mutational status will be considered negative if homozygous deletions, putative deleterious genomic alterations, or both are not detected in genes in the panel.

7.2. Efficacy Assessments

7.2.1. Primary Efficacy Endpoint: Radiographic Progression-Free Survival

For Part 2, the primary efficacy endpoints are:

- BICR assessed rPFS per RECIST 1.1 (soft tissue disease) and PCWG3 (bone disease) in patients with mCRPC unselected for DDR status.

- BICR assessed rPFS per RECIST 1.1 (soft tissue disease) and PCWG3 (bone disease) in patients with mCRPC harboring DDR deficiencies.

Soft tissue disease status will be assessed at regular intervals during the course of the study by CT of chest and CT or MRI of abdomen and pelvis. Bone disease status will be assessed by whole body radionuclide bone scan.

090177e19c2fb240\Approved\Approved On: 23-Dec-2022 10:25 (GMT)

When medically acceptable, contrast-enhanced CT scan is the preferred method for assessment of soft tissue disease and will be performed on the chest, abdomen, and pelvis. Patients who are intolerant to CT contrast agents should undergo CT scans without contrast enhancement of the chest and contrast-enhanced MRI scans of the abdomen and pelvis for assessment of soft tissue disease. As a final alternative, CT scans without contrast enhancement of the chest, abdomen and pelvis are also acceptable. Soft tissue response and progression will be evaluated per RECIST 1.1. Whole-body bone scans will be performed to assess the presence of bone disease. Bone disease is not to be assessed by CT or MRI. Progression of bone disease will be evaluated per PCWG3 [59].

For any given patient, the imaging modality(s) used for soft tissue assessment of a given lesion at screening should be the same used throughout the study, unless doing so would compromise patient safety. The investigator is asked to inform the sponsor prior to a change in soft tissue imaging modality and the reason for doing do, prior to the radiographic assessment, whenever possible.

For patients enrolled in Part 1, all scans performed at screening and during the study will be read locally by radiologists and nuclear medicine bone scan readers at the study site and results entered in the CRF.

For patients enrolled in Part 2, all scans performed at screening and during the study will be submitted electronically or by mail to the blinded independent central reviewers, and results communicated back to the site as described in the imaging manual; please see Section 7.7. All images will also be read by radiologists at the study site and locally determined assessments will be entered in the appropriate CRF.

7.2.2. Assessment of Secondary Efficacy Endpoints

The secondary efficacy endpoints include overall survival, proportion of patients with measurable soft tissue disease at baseline with a confirmed objective response, time to confirmed PSA progression, time to initiation of cytotoxic chemotherapy, proportion of patients with confirmed PSA response ≥50%, duration of soft tissue response, time to pain progression, time to first symptomatic skeletal event, time to initiation of antineoplastic therapy, and time to opiate use for prostate cancer pain.

The study assessments of efficacy for these endpoints will include standard radiographic and imaging methods to evaluate disease progression and response (Section 7.2.1), PSA, survival status monitoring (Section 6.5), evaluation of concomitant treatment (Section 5.9), and pain evaluation using the BPI-SF questionnaire.

Blood samples will be drawn for analysis of PSA levels to determine biochemical response and progression. PSA will be assessed locally throughout the study until radiographic progression according to the Schedules of Activities (Table 1, Table 2, and Table 3). Investigators are strongly discouraged from discontinuing study treatments or initiating new systemic therapy due to a rising PSA for patients in Part 2, where study treatment administration should continue until study discontinuation criteria (BICR determined radiographic progression, or the patient is no longer clinically benefitting in the opinion of the investigator, or unacceptable toxicity, or withdrawal of consent, or death) are met.

090177e19c2fb240\Approved\Approved On: 23-Dec-2022 10:25 (GMT)

Time to first symptomatic skeletal event will be defined as time to spinal cord compression, symptomatic bone fracture, or radiotherapy or surgery to bone, whichever occurs first, as determined from assessment of adverse event data.

7.2.3. Exploratory Efficacy Endpoint Assessments

Proportion of patients with conversion from 5 or more CTCs per 7.5 ml at baseline to 4 or fewer CTCs per 7.5 ml post-baseline; proportion of patients with conversion from detectable CTCs at baseline to CTCs=0 post-baseline; proportion of patients with baseline CTCs <5 showing increased CTCs post baseline;[54] molecular profiles of tumor tissue remaining after genomic testing for eligibility, of saliva, and of ctDNA; circulating protein biomarker profiles. These results will be analyzed for patients with mCRPC unselected for DDR status and patients with mCRPC harboring DDR mutations.

7.3. Patient-Reported Outcomes Assessments

Patient-reported outcome assessments will be completed by patients in Part 2 as follows. On Day 1, patients will complete PRO questionnaires before the first dose of study treatment. At subsequent visits, patients will complete these questionnaires at the site before any other study activities and in the same order at each visit. These questionnaires will be collected according to the Schedule of Assessments.

Prostate cancer related pain will be assessed electronically using the BPI-SF questionnaire, a validated 9-item instrument that uses a self-reported scale assessing level of pain, its effect on activities of daily living, and analgesic medication use. Pain severity at its worst will be assessed electronically using BPI-SF question 3. Tracking analgesic use is particularly important to ensure that the delay in pain progression observed is truly the result of the treatment being studied rather than the result of an increase in analgesic use. Pain (assessed using BPI-SF question 3) and analgesic use (assessed using an analgesic log) will be collected electronically for each of the 7 consecutive days prior to study visits; the timing of collection is shown in Table 2 and described in Section 6.2.

Cancer-specific global health status/quality of life, functioning, and symptoms data will be collected electronically using the EORTC QLQ-C30 and QLQ-PR25 questionnaires and general health status will be assessed using the EQ-5D-5L health questionnaire.

The EORTC QLQ-C30 is a well-known standard instrument used in oncology trials. It consists of a 30-item questionnaire which are grouped into five multi-item functional subscales (physical, role, emotional, cognitive , and social functioning), three multi-item symptom scales (fatigue, nausea/vomiting, and pain), a global quality of life (QOL) subscale, and six single item symptom scales assessing other cancer-related symptoms (dyspnea, sleep disturbance, appetite loss, constipation, diarrhea, and the financial impact of cancer). The questionnaire uses 4-point Likert scales with responses from “not at all” to “very much” to assess all functioning and symptoms items and two 7-point Likert scales for overall health and overall QOL. Responses to all items are then converted to a 0 to 100 scale using a standard scoring algorithm. For functional and global QOL scales, higher scores represent a

090177e19c2fb240\Approved\Approved On: 23-Dec-2022 10:25 (GMT)

better level of functioning/QOL. For symptom-oriented scales, a higher score represents more extreme symptoms.

The EORTC QLQ-PR25 is a prostate cancer-specific module of the EORTC questionnaire that assesses the functioning and symptoms of prostate cancer patients. Patients will self-rate their current state of pain as it relates to urination, ease and frequency of urination, and bowel and other symptoms and signs during the past week. Patients will also answer 5 questions about weight loss/gain and sexual interest and 4 questions about sexual activity during the past 4 weeks. Patients will choose 1 of 4 possible responses that record level of intensity (not at all, a little, quite a bit, very much) within each dimension.

The EQ-5D-5L is a validated and standardized instrument that measures the general health status of patients. Patients will self-rate their current state of mobility, self-care, usual activities, pain/discomfort, and anxiety/depression by choosing 1 of 5 possible responses that record the level of severity (no problems, slight problems, moderate problems, severe problems, or extreme problems) within each dimension. The questionnaire also includes a visual analog scale to self-rate general health state on a scale from "the worst health you can imagine" to "the best health you can imagine."

7.4. Pharmacokinetic Assessments

For patients participating in Part 1, blood samples for PK analysis of talazoparib, enzalutamide and its N-desmethyl metabolite will be collected as shown in Table 2.

All patients participating in Part 2 will have blood samples collected for PK assessments as shown in Table 2. For patients who receive talazoparib and enzalutamide, PK samples will be analyzed for talazoparib and enzalutamide (and its N-desmethyl metabolite) concentrations. For patients who receive placebo and enzalutamide, PK samples will be analyzed for enzalutamide (and its N-desmethyl metabolite) concentration. Additional PK blood samples may be collected from patients experiencing unexpected or serious adverse events, or adverse events that lead to discontinuation.

All efforts will be made to obtain the PK samples at the scheduled nominal time relative to dosing. Samples obtained within 10% of the nominal time will be considered protocol compliant. For pre-dose PK samples, collection should occur prior to administration of the investigational product on that day. Patients must be instructed to withhold their daily dose of study treatment on PK sampling days until the pre-dose PK sample collection has been completed. The actual time of the sample collection and the most recent dosing time before and after each collection will be recorded on the CRF. The date of missing/reduced dose should also be recorded in the CRF.

Refer to the lab manual for detailed collection, processing and shipping procedures.

7.5. Biomarkers

For enrolled subjects, exploratory targeted and/or whole exome/genome sequencing and/or transcriptome and/or epigenetic analyses will be performed using the remainder of the de novo or archival tumor tissue samples submitted at prescreening or screening for eligibility

090177e19c2fb240\Approved\Approved On: 23-Dec-2022 10:25 (GMT)

assessment (optional for patients enrolled in Part 1) to gain insight into the mechanisms that confer sensitivity to study treatment. The results of these analyses may be combined with the results from targeted gene sequencing performed for enrollment eligibility assessment (defined DDR genes and other genes included in the FoundationOne® gene panel used in this test, and potential additional genomic computational assessments determined using these FoundationOne® data such as genomic loss of heterozygosity, tumor mutational burden, and somatic-germline-zygosity analysis) to further explore correlations of molecular profile with response to study treatment.

For enrolled subjects, blood samples for circulating tumor DNA (ctDNA) analyses will be collected for central lab analysis using a targeted sequencing panel including DDR genes. In addition, exploratory whole exome/genome sequencing and/or epigenetic analyses may be performed. The goal of these analyses is to explore potential correlations of tumor molecular profile and, potentially, ctDNA burden (as assessed by variant allele frequency and/or other validated method) with response to study treatment and to understand potential mechanisms of acquired resistance to study treatment. The results of these ctDNA analyses may be combined with the results from targeted gene sequencing performed for enrollment eligibility assessment (defined DDR genes and other genes included in the FoundationOne Liquid NGS gene panel used in this test, and any potential associated genomic computational assessments). In addition, these samples may be used to help support development of a potential ctDNA-based diagnostic test for talazoparib.

Unless prohibited by local regulations or ethics committee decision, saliva samples will be collected on Day 1 before the first dose of study treatment for exploratory targeted and/or whole exome/genome sequencing. These samples will be used to characterize the germline status of the DDR genes used to assess patient eligibility or a subset thereof (see STUDY DESIGN) in order to explore the potential contribution of germline DDR status to efficacy and safety/tolerability with study treatment. They will not otherwise be used to generate free-standing germline sequencing results, but rather only as controls to assist in identifying and profiling somatic tumor DNA mutations. Collection of these samples is optional for patients enrolled in Part 1 but they should be collected if tumor tissue for genomic screening is collected from the same patient; collection of these samples is required for patients enrolled in Part 2.

CTC enumeration will be performed at a central lab using blood samples collected during the study. CTC counts will be assessed as a candidate pharmacodynamic biomarker and/or predictive biomarker of response and/or resistance. The proportion of patients with conversion from 5 or more CTCs per 7.5 ml blood at baseline to 4 or fewer CTCs per 7.5 ml blood post-baseline will be determined, as will the proportion of patients with conversion from detectable CTCs at baseline to CTCs=0 per 7.5 ml blood post-baseline. In addition, the proportion of patients with baseline CTC counts <5 showing increased CTC counts post-baseline will be assessed. Results will be analyzed for patients with mCRPC unselected for DDR status and for patients with mCRPC harboring DDR mutations. Subjects who prove non-evaluable for CTC enumeration at baseline (both screening and Day 1 sample must be non-evaluable) may forego collection of additional CTC samples.

090177e19c2fb240\Approved\Approved On: 23-Dec-2022 10:25 (GMT)

Blood samples for protein biomarker analyses will be collected during the study for analyses to help understand the pharmacodynamic effects of study treatment and to explore candidate predictive molecular signatures associated with response and resistance.

Please refer to footnotes in Table 2, Schedule of Activities, for details.

7.5.1. China

Whole exome/genome sequencing and/or transcriptome and/or epigenetic analyses will not be required for subjects enrolled in China. Biospecimens for banking and protein biomarkers test will not be collected and analyzed in China.

Exploratory biomarker samples will not be required for subjects enrolled in China until the Human Genetics Resources Administration of China (HGRAC) approves the exploratory research.

Exploratory biomarkers are depicted under the Biomarker Assessment Category in the Schedule of Activities (Table 2). Exploratory Biomarker Assessments in China include blood samples for CTC analysis, ctDNA, and saliva. These samples should not be collected until the HGRAC approves the exploratory research. Depending on assay availability, blood may be substituted for saliva in China for retrospective exploratory characterization of the germline status of the DDR genes used to assess patient eligibility or a subset thereof. Furthermore, depending on the assay availability, ctDNA might be tested by China local CRO using different NGS panel from FoundationOneLiquid®.

7.6. Banked Biospecimens

Banked biospecimens will be collected from patients for exploratory research relating to the drug response and prostate cancer. These collections are not typically associated with a planned assessment described in the protocol. They will be handled in a manner that protects each patient's privacy and confidentiality. Banked biospecimens will be assigned the patient's study identification code (ID) at the site. The data generated from these banked biospecimens will also be indexed by this ID. Biospecimens will be kept until destruction in facilities with access limited to authorized personnel, and biospecimen-derived data will be stored on password protected computer systems. The key between the patient's ID and the patient's direct personally identifying information (eg, name, address) will be held at the study site. Biospecimens will be used only for the purposes described in the protocol and informed consent document; any other uses require additional ethical approval. Unless a time limitation is required by local regulations or ethical requirements, biospecimens will be stored for many years (no time limit) to allow for research in the future, including research conducted during the lengthy drug-development process and also post marketing research. Patients may withdraw their consent for the use of their banked biospecimens at any time by making a request to the investigator; in this case, any remaining biospecimens will be destroyed, but data already generated from the biospecimens will continue to be available to protect the integrity of existing analyses.

090177e19c2fb240\Approved\Approved On: 23-Dec-2022 10:25 (GMT)

Unless prohibited by local regulations or ethics committee decision, a 4-mL blood genomic banked bio-specimen Prep D1 (dipotassium edetic acid [ethylenediaminetetraacetic acid] [K_2EDTA] whole-blood collection optimized for DNA analysis) will be collected on Day 1 as specified in Table 2 and will be retained for potential pharmacogenomic/genomic/biomarker analyses related to drug response and prostate cancer. For example, putative safety biomarkers, drug-metabolizing enzyme genes, drug-transport protein genes, or genes thought to be related to the mechanism of drug action may be examined. The primary purpose is to examine DNA; however, the biospecimen may also be used to study other molecules (eg, RNA, proteins, and metabolites).

The banked biospecimens will be collected from all patients unless prohibited by local regulations or IRB/EC decision.

It is possible that the use of these biospecimens may result in commercially viable products. Patients will be advised in the informed consent document that they will not be compensated in this event.

7.6.1. Additional Research

Unless prohibited by local regulations or IRB/EC decision, subjects will be asked to indicate on the consent form whether they will allow banked biospecimens to also be used to design and conduct research in order to gain a further understanding of other diseases and to advance science, including development of other medicines for patients.

Subjects need not provide additional biospecimens for the uses described in this section; the biospecimens specified in the Banked Biospecimens section will be used. Subjects may still participate in the study if they elect not to allow their banked biospecimens to be used for the additional purposes described in this section.

7.7. Imaging Assessments

It is important to the integrity of the study that all imaging studies for patients in Part 2 are forwarded to the independent core imaging laboratory as soon as requested by the Sponsor.

Further information on materials to be forwarded for independent review, correct procedures for the coding/blinding of the patient's name/identity and the return of the source data/documents to the site is provided in the imaging manual.

Management of incidental findings

An incidental finding is one unknown to the subject that has potential health or reproductive importance, which is discovered unexpectedly in the course of a research study, but is unrelated to the purpose and beyond the aims of the study. If, during the independent review process, an unexpected observation is identified, this finding may be shared with the study sponsor for disclosure to the principal investigator. The principal investigator will be responsible for reporting any AEs identified from incidental findings as described in the ADVERSE EVENT REPORTING section. Identification of such incidental findings during the independent review process should not be expected, and the site maintains responsibility for performing local reads of all images obtained in the study.

090177e19c2fb240\Approved\Approved On: 23-Dec-2022 10:25 (GMT)

7.8. Rater Qualifications

Radiologists will be board-certified in their discipline or carry similar credentials as outlined in the Independent Radiographic Review Charter.

7.9. Safety Assessments

Safety endpoints include adverse events (graded according to the NCI CTCAE, version 4.03), physical examination (including blood pressure and pulse), and laboratory tests (hematology and chemistry). In South Africa only: Optional HIV testing using the ELISA test may be conducted for subjects of unknown HIV status per investigator discretion. Subjects will be asked to sign an HIV consent form and be given pre and post test counseling at the site.

7.9.1. Adverse Events

The procedures for the investigator assessment of adverse events are presented in Section 8.

7.9.2. Physical Examinations

Physical examinations will be conducted according to the schedule of activities in Table 1 and Table 2. At screening, this will constitute an assessment of systems (eg, general appearance, head, eyes, ears, nose, mouth, throat, skin, heart, lung, lymph nodes, gastrointestinal, genitourinary, neurologic, and skeletal). During treatment, systems will be assessed per standard of care at the study site or as clinically indicated by symptoms.

7.9.3. Vital Signs

Vital sign measurements (blood pressure, heart rate, and temperature) height and weight will be assessed as shown in Table 1 and Table 2. Height is only applicable to patients in Part 2 and only required to be collected at Screening.

7.9.4. Clinical Laboratory Assessments

Blood samples for hematology, serum chemistry assessments, PSA, testosterone, reticulocyte count, erythropoietin, serum folate and B12 will be collected as shown in Table 1 and Table 2. The required laboratory tests are listed in Table 12.

090177e19c2fb240\Approved\Approved On: 23-Dec-2022 10:25 (GMT)

Table 12. Required Laboratory Tests (fasting is not mandatory)

Hematology	Serum Chemistry	Additional
Hematocrit	Albumin	PSA
Hemoglobin	Total protein	Testosterone (screening only)
Mean corpuscular volume	Alkaline phosphatase	Serum folate[b]
RBC	ALT	B12[b]
Platelets	AST	
Reticulocyte count[b]	Total bilirubin[a]	In South Africa only: Optional HIV testing using the ELISA test may be conducted for subjects of unknown HIV status per investigator discretion.
Erythropoietin[b]		
WBC with differential[d]	BUN or urea	
• ANC	Creatinine	
• Lymphocytes	Glucose	
• Monocytes	Bicarbonate or CO_2[c]	
• Eosinophils	Calcium	
• Basophils	Chloride	
	Magnesium	
	Phosphate	
	Potassium	
	Sodium	
	LDH	

ALT = alanine aminotransferase; ANC = absolute neutrophil count; AST = aspartate aminotransferase; BUN = blood urea nitrogen; CRF = case report form; INR = international normalized ratio; LDH = lactate dehydrogenase; PT = prothrombin time; RBC = red blood cell; WBC = white blood cell.

a. For potential Hy's Law cases, in addition to repeating AST and ALT, laboratory tests should include albumin, creatine kinase, total bilirubin, direct and indirect bilirubin, gamma-glutamyl transferase, PT/INR, and alkaline phosphatase.
b. Not required if the test is not available at the site laboratory.
c. Not required if not included in the chemistry panel at the site laboratory. Investigators will follow up per standard of care of the institution (eg, blood gas analysis) if warranted by required laboratory test results.
d. Absolute values to be entered in the CRF. If only percentages are provided by the local laboratory, percentages can be entered. If % neutrophils are not provided, but % segmented neutrophils and % neutrophil bands are provided these should be entered in the CRF. In the event that % segmented neutrophils and % bands are provided OR only % neutrophils are provided without ANC, the site must manually calculate ANC by using the formula at https://www.mdcalc.com/absolute-neutrophil-count-anc).

090177e19c2fb240\Approved\Approved On: 23-Dec-2022 10:25 (GMT)

8. ADVERSE EVENT REPORTING

8.1. Requirements

The table below summarizes the requirements for recording safety events on the CRF and for reporting safety events on the Clinical Trial (CT) Serious Adverse Event (SAE) Report Form to Pfizer Safety. These requirements are delineated for 3 types of events: (1) SAEs; (2) non-serious adverse events (AEs); and (3) exposure to the investigational product under study during pregnancy or breastfeeding, and occupational exposure.

**Safety Event**	**Recorded on the CRF**	**Reported on the CT SAE Report Form to Pfizer Safety Within 24 Hours of Awareness**
SAE	All	All
Non-serious AE	All	None
Exposure to the investigational product under study during pregnancy or breastfeeding, and occupational exposure	All (regardless of whether associated with an AE), **except occupational exposure**	Exposure during pregnancy, exposure via breastfeeding, occupational exposure (regardless of whether associated with an AE)

All observed or volunteered events regardless of treatment group or suspected causal relationship to the investigational product(s) will be reported as described in the following paragraphs.

Events listed in the table above that require reporting to Pfizer Safety on the CT SAE Report Form within 24 hours of awareness of the event by the investigator **are to be reported regardless of whether the event is determined by the investigator to be related to an investigational product under study**. In particular, if the SAE is fatal or life-threatening, notification to Pfizer Safety must be made immediately, irrespective of the extent of available event information. This time frame also applies to additional new (follow-up) information on previously forwarded reports. In the rare situation that the investigator does not become immediately aware of the occurrence of an event, the investigator must report the event within 24 hours after learning of it and document the time of his/her first awareness of the event.

For each event, the investigator must pursue and obtain adequate information both to determine the outcome and to assess whether it meets the criteria for classification as an SAE (see the Serious Adverse Events section below). In addition, the investigator may be requested by Pfizer Safety to obtain specific follow-up information in an expedited fashion. This information is more detailed than that recorded on the CRF. In general, this will include a description of the event in sufficient detail to allow for a complete medical assessment of

090177e19c2fb240\Approved\Approved On: 23-Dec-2022 10:25 (GMT)

the case and independent determination of possible causality. Any information relevant to the event, such as concomitant medications and illnesses, must be provided. In the case of a patient death, a summary of available autopsy findings must be submitted as soon as possible to Pfizer Safety. Any pertinent additional information must be reported on the CT SAE Report Form; additional source documents (eg, medical records, CRF, laboratory data) are to be sent to Pfizer Safety **ONLY** upon request.

As part of ongoing safety reviews conducted by the sponsor, any non-serious AE that is determined by the sponsor to be serious will be reported by the sponsor as an SAE. To assist in the determination of case seriousness, further information may be requested from the investigator to provide clarity and understanding of the event in the context of the clinical study.

8.1.1. Additional Details On Recording Adverse Events on the CRF

All events detailed in the table above will be recorded on the AE page(s) of the CRF. It should be noted that the CT SAE Report Form for reporting of SAE information is not the same as the AE page of the CRF. When the same data are collected, the forms must be completed in a consistent manner. AEs should be recorded using concise medical terminology and the same AE term should be used on both the CRF and the CT SAE Report Form for reporting of SAE information.

8.1.2. Eliciting Adverse Event Information

The investigator is to record on the CRF all directly observed AEs and all AEs spontaneously reported by the study patient/legally acceptable representative. In addition, each study patient/legally acceptable representative will be questioned about the occurrence of AEs in a non-leading manner.

8.1.3. Withdrawal From the Study Due to Adverse Events (see also the Patient Withdrawal section)

Withdrawal due to AEs should be distinguished from withdrawal due to other causes, according to the definition of AE noted below, and recorded on the CRF.

When a patient withdraws from the study because of an SAE, the SAE must be recorded on the CRF and reported, as appropriate, on the CT SAE Report Form, in accordance with the Requirements section above.

8.1.4. Time Period for Collecting AE/SAE Information

The time period for actively eliciting and collecting AEs and SAEs (“active collection period”) for each patient begins from the time the patient provides informed consent, which is obtained before the patient’s participation in the study (ie, before undergoing any study-related procedure and/or receiving investigational product), through and including a minimum of 28 calendar days after the last administration of the investigational product (talazoparib, talazoparib/placebo, enzalutamide, whichever is taken last).

090177e19c2fb240\Approved\Approved On: 23-Dec-2022 10:25 (GMT)

A separate time period for actively eliciting and collecting AEs and "Research Related Injury" applies only to patients who sign the molecular prescreening consent as described in Section 8.1.5.

For patients who are screen failures, the active collection period ends when screen failure status is determined.

8.1.4.1. Reporting SAEs to Pfizer Safety

All SAEs occurring in a patient during the active collection period are reported to Pfizer Safety on the CT SAE Report Form.

SAEs occurring in a patient after the active collection period has ended are reported to Pfizer Safety if the investigator becomes aware of them; at a minimum, all SAEs that the investigator believes have at least a reasonable possibility of being related to investigational product must be reported to Pfizer Safety. All SAEs of myelodysplastic syndrome (MDS) or acute myeloid leukemia (AML) are to be reported to Pfizer Safety irrespective of study treatment, investigator's opinion of causality or time of diagnosis.

Follow up by the investigator continues throughout and after the active collection period and until the event or its sequelae resolve or stabilize at a level acceptable to the investigator, and Pfizer concurs with that assessment.

If a patient begins a new anticancer therapy, SAEs occurring during the above-indicated active collection period must still be reported to Pfizer Safety irrespective of any intervening treatment.

8.1.4.2. Recording Non-serious AEs and SAEs on the CRF

During the active collection period, both non-serious AEs and SAEs are recorded on the CRF.

Follow-up by the investigator may be required until the event or its sequelae resolve or stabilize at a level acceptable to the investigator, and Pfizer concurs with that assessment.

If a patient begins a new anticancer therapy, the recording period for non-serious AEs ends at the time the new treatment is started; however, SAEs must continue to be recorded on the CRF during the above-indicated active collection period.

8.1.5. Time Period for Collecting AEs and/or Research Related Injuries for Patients Who Sign the Molecular Prescreening Consent

8.1.5.1. Adverse Events

An AE is defined as any untoward medical occurrence and can therefore be any unfavorable and unintended sign (including an abnormal laboratory finding), symptom, or disease, whether or not related to the subject's participation in the study.

090177e19c2fb240\Approved\Approved On: 23-Dec-2022 10:25 (GMT)

During prescreening, any AE that occurs from the time the patient undergoes a procedure to obtain de novo tissue or blood through and including 14 days from completion of said procedure, must be recorded on the CRF. The investigator is required to assess whether the AE may be related to the patient's participation in the study.

The investigator must pursue and obtain information adequate to determine the outcome of the AE and to assess whether it meets the criteria for classification as a research-related injury requiring immediate notification to Pfizer as described below.

8.1.5.2. Research-Related Injuries

Should a subject, in the investigator's opinion, suffer a medically important research-related injury following blood sample collection or de novo tumor biopsy at a prescreening visit caused by their participation in the study, the designated Pfizer clinician or sponsor must be notified immediately. Any research related injury that occurs from the time the patient undergoes a procedure to obtain blood or de novo tissue through and including 14 days upon completion of said procedure, must be recorded on the CRF.

A medically important research-related injury is any untoward medical occurrence that:

- Results in death;
- Is life-threatening (immediate risk of death);
- Requires inpatient hospitalization or prolongation of existing hospitalization;
- Results in persistent or significant disability/incapacity (substantial disruption of the ability to conduct normal life functions);
- Results in congenital anomaly/birth defect.

Medical and scientific judgment is exercised in determining whether an injury is an important medical event. An important medical event may not be immediately life-threatening and/or result in death or hospitalization. However, if it is determined that the event may jeopardize the subject or may require intervention to prevent one of the other outcomes listed in the definition above, the important medical event should be reported as a research related injury.

An investigator may be requested by the designated Pfizer clinician or sponsor to obtain specific additional follow-up information in an expedited fashion. In general, this will include a description of the injury in sufficient detail to allow for a complete medical assessment of the case and independent determination of possible causality. Information on other possible causes of the event, such as concomitant treatments, vaccines, and/or illnesses must be provided. In the case of a subject death, a summary of available autopsy findings must be submitted as soon as possible to Pfizer or its designated representative.

8.1.6. Causality Assessment

The investigator's assessment of causality must be provided for all AEs (serious and non-serious); the investigator must record the causal relationship on the CRF, and report such

090177e19c2fb240\Approved\Approved On: 23-Dec-2022 10:25 (GMT)

an assessment in accordance with the SAE reporting requirements, if applicable. An investigator's causality assessment is the determination of whether there exists a reasonable possibility that the investigational product caused or contributed to an AE; generally the facts (evidence) or arguments to suggest a causal relationship should be provided. If the investigator does not know whether or not the investigational product caused the event, then the event will be handled as "related to investigational product" for reporting purposes, as defined by the sponsor. If the investigator's causality assessment is "unknown but not related" to investigational product, this should be clearly documented on study records.

In addition, if the investigator determines that an SAE is associated with study procedures, the investigator must record this causal relationship in the source documents and CRF, and report such an assessment in the dedicated section of the CT SAE Report Form and in accordance with the SAE reporting requirements.

8.1.7. Sponsor's Reporting Requirements to Regulatory Authorities

AE reporting, including suspected unexpected serious adverse reactions, will be carried out in accordance with applicable local regulations.

8.2. Definitions

8.2.1. Adverse Events

An AE is any untoward medical occurrence in a study patient administered a product or medical device; the event need not necessarily have a causal relationship with the treatment or usage. Examples of AEs include, but are not limited to:

- Abnormal test findings;
- Clinically significant signs and symptoms;
- Changes in physical examination findings;
- Hypersensitivity;
- Drug abuse;
- Drug dependency.

Additionally, AEs may include signs and symptoms resulting from:

- Drug overdose;
- Drug withdrawal;
- Drug misuse;
- Drug interactions;

090177e19c2fb240\Approved\Approved On: 23-Dec-2022 10:25 (GMT)

- Extravasation;
- Exposure during pregnancy (EDP);
- Exposure via breastfeeding;
- Medication error;
- Occupational exposure.

Worsening of signs and symptoms of the malignancy under study should be recorded as AEs in the appropriate section of the CRF. Disease progression assessed by measurement of malignant lesions on radiographs or other methods should not be reported as AEs.

8.2.2. Abnormal Test Findings

Abnormal objective test findings should be recorded as AEs when any of the following conditions are met:

- Test result is associated with accompanying symptoms; and/or
- Test result requires additional diagnostic testing or medical/surgical intervention; and/or
- Test result leads to a change in study dosing (outside of any protocol-specified dose adjustments) or discontinuation from the study, significant additional concomitant drug treatment, or other therapy; and/or
- Test result is considered to be an AE by the investigator or sponsor.

Merely repeating an abnormal test, in the absence of any of the above conditions, does not constitute an AE. Any abnormal test result that is determined to be an error does not require recording as an AE.

8.2.3. Serious Adverse Events

A serious adverse event is any untoward medical occurrence at any dose that:

- Results in death;
- Is life-threatening (immediate risk of death);
- Requires inpatient hospitalization or prolongation of existing hospitalization;
- Results in persistent or significant disability/incapacity (substantial disruption of the ability to conduct normal life functions);
- Results in congenital anomaly/birth defect.

090177e19c2fb240\Approved\Approved On: 23-Dec-2022 10:25 (GMT)

Or that is considered to be:

- An important medical event.

Medical and scientific judgment is exercised in determining whether an event is an important medical event. An important medical event may not be immediately life-threatening and/or result in death or hospitalization. However, if it is determined that the event may jeopardize the patient or may require intervention to prevent one of the other AE outcomes, the important medical event should be reported as serious.

Examples of such events are intensive treatment in an emergency room or at home for allergic bronchospasm; blood dyscrasias or convulsions that do not result in hospitalization; or development of drug dependency or drug abuse.

Progression of the malignancy under study (including signs and symptoms of progression) should not be reported as an SAE unless the outcome is fatal within the active collection period. Hospitalization due to signs and symptoms only of disease progression should not be reported as an SAE. If the malignancy has a fatal outcome during the study or within the active collection period, then the event leading to death must be recorded as an AE on the CRF, and as an SAE with Common Terminology Criteria for Adverse Events (CTCAE, version 4.03) Grade 5 (see the Severity Assessment section).

8.2.4. Hospitalization

Hospitalization is defined as any initial admission (even less than 24 hours) in a hospital or equivalent healthcare facility, or any prolongation of an existing admission. Admission also includes transfer within the hospital to an acute/intensive care unit (eg, from the psychiatric wing to a medical floor, medical floor to a coronary care unit, or neurological floor to a tuberculosis unit). An emergency room visit does not necessarily constitute a hospitalization; however, the event leading to the emergency room visit is assessed for medical importance.

Hospitalization does not include the following:

- Rehabilitation facilities;
- Hospice facilities;
- Respite care (eg, caregiver relief);
- Skilled nursing facilities;
- Nursing homes;
- Same-day surgeries (as outpatient/same-day/ambulatory procedures).

090177e19c2fb240\Approved\Approved On: 23-Dec-2022 10:25 (GMT)

Hospitalization or prolongation of hospitalization in the absence of a precipitating clinical AE is not in itself an SAE. Examples include:

- Admission for treatment of a preexisting condition not associated with the development of a new AE or with a worsening of the preexisting condition (eg, for workup of a persistent pretreatment laboratory abnormality);
- Social admission (eg, patient has no place to sleep);
- Administrative admission (eg, for yearly physical examination);
- Protocol-specified admission during a study (eg, for a procedure required by the study protocol);
- Optional admission not associated with a precipitating clinical AE (eg, for elective cosmetic surgery);
- Hospitalization for observation without a medical AE;
- Preplanned treatments or surgical procedures. These should be noted in the baseline documentation for the entire protocol and/or for the individual patient;
- Admission exclusively for the administration of blood products.

Diagnostic and therapeutic noninvasive and invasive procedures, such as surgery, should not be reported as SAEs. However, the medical condition for which the procedure was performed should be reported if it meets the definition of an SAE. For example, an acute appendicitis that begins during the reporting period should be reported if the SAE requirements are met, and the resulting appendectomy should be recorded as treatment of the AE.

8.3. Severity Assessment

The investigator will use the following definitions of severity in accordance with the current CTCAE version (4.03) to describe the maximum intensity of the adverse event.

GRADE	Clinical Description of Severity
0	No change from normal or reference range (This grade is not included in the Version 4.03 CTCAE document but may be used in certain circumstances.)
1	MILD adverse event
2	MODERATE adverse event
3	SEVERE adverse event
4	LIFE-THREATENING consequences; urgent intervention indicated

090177e19c2fb240\Approved\Approved On: 23-Dec-2022 10:25 (GMT)

GRADE	Clinical Description of Severity
5	DEATH RELATED TO adverse event

Note the distinction between the severity and the seriousness of an AE. A severe event is not necessarily an SAE. For example, a headache may be severe (interferes significantly with the patient's usual function) but would not be classified as serious unless it met one of the criteria for SAEs, listed above.

8.4. Special Situations

8.4.1. Protocol-Specified Serious Adverse Events

There are no protocol-specified SAEs in this study. All SAEs will be reported to Pfizer Safety by the investigator as described in previous sections, and will be handled as SAEs in the safety database.

8.4.2. Potential Cases of Drug-Induced Liver Injury

Humans exposed to a drug who show no sign of liver injury (as determined by elevations in transaminases) are termed "tolerators," while those who show transient liver injury, but adapt are termed "adaptors." In some patients, transaminase elevations are a harbinger of a more serious potential outcome. These patients fail to adapt and therefore are "susceptible" to progressive and serious liver injury, commonly referred to as drug-induced liver injury (DILI). Patients who experience a transaminase elevation above 3 times the upper limit of normal (× ULN) should be monitored more frequently to determine if they are an "adaptor" or are "susceptible."

In the majority of DILI cases, elevations in aspartate aminotransferase (AST) and/or alanine aminotransferase (ALT) precede total bilirubin (TBili) elevations (>2 × ULN) by several days or weeks. The increase in TBili typically occurs while AST/ALT is/are still elevated above 3 × ULN (ie, AST/ALT and TBili values will be elevated within the same lab sample). In rare instances, by the time TBili elevations are detected, AST/ALT values might have decreased. This occurrence is still regarded as a potential DILI. Therefore, abnormal elevations in either AST OR ALT in addition to TBili that meet the criteria outlined below are considered potential DILI (assessed per Hy's law criteria) cases and should always be considered important medical events, even before all other possible causes of liver injury have been excluded.

The threshold of laboratory abnormalities for a potential DILI case depends on the patient's individual baseline values and underlying conditions. Patients who present with the following laboratory abnormalities should be evaluated further as potential DILI (Hy's law) cases to definitively determine the etiology of the abnormal laboratory values:

- Patients with AST/ALT and TBili baseline values within the normal range who subsequently present with AST **OR** ALT values >3 × ULN **AND** a TBili value >2 × ULN with no evidence of hemolysis and an alkaline phosphatase value <2 × ULN or not available;

090177e19c2fb240\Approved\Approved On: 23-Dec-2022 10:25 (GMT)

- For patients with baseline AST **OR** ALT **OR** TBili values above the ULN, the following threshold values are used in the definition mentioned above, as needed, depending on which values are above the ULN at baseline:

  - Preexisting AST or ALT baseline values above the normal range: AST or ALT values >2 times the baseline values AND >3 × ULN; or >8 × ULN (whichever is smaller).

  - Preexisting values of TBili above the normal range: TBili level increased from baseline value by an amount of at least 1 × ULN **or** if the value reaches >3 × ULN (whichever is smaller).

Rises in AST/ALT and TBili separated by more than a few weeks should be assessed individually based on clinical judgment; any case where uncertainty remains as to whether it represents a potential Hy's law case should be reviewed with the sponsor.

The patient should return to the investigator site and be evaluated as soon as possible, preferably within 48 hours from awareness of the abnormal results. This evaluation should include laboratory tests, detailed history, and physical assessment.

In addition to repeating measurements of AST and ALT and TBili, laboratory tests should include albumin, creatine kinase (CK), direct and indirect bilirubin, gamma-glutamyl transferase (GGT), prothrombin time (PT)/international normalized ratio (INR), total bile acids, alkaline phosphatase and acetaminophen drug and/or protein adduct levels. Consideration should also be given to drawing a separate tube of clotted blood and an anticoagulated tube of blood for further testing, as needed, for further contemporaneous analyses at the time of the recognized initial abnormalities to determine etiology. A detailed history, including relevant information, such as review of ethanol, acetaminophen (either by itself or as a coformulated product in prescription or over-the-counter medications), recreational drug, supplement (herbal) use and consumption, family history, sexual history, travel history, history of contact with a jaundiced person, surgery, blood transfusion, history of liver or allergic disease, and potential occupational exposure to chemicals, should be collected. Further testing for acute hepatitis A, B, C, D, and E infection and liver imaging (eg, biliary tract) may be warranted.

All cases demonstrated on repeat testing as meeting the laboratory criteria of AST/ALT and TBili elevation defined above should be considered potential DILI (Hy's law) cases if no other reason for the liver function test (LFT) abnormalities has yet been found. **Such potential DILI (Hy's law) cases are to be reported as SAEs, irrespective of availability of all the results of the investigations performed to determine etiology of the LFT abnormalities.**

A potential DILI (Hy's law) case becomes a confirmed case only after all results of reasonable investigations have been received and have excluded an alternative etiology.

090177e19c2fb240\Approved\Approved On: 23-Dec-2022 10:25 (GMT)

8.4.3. Exposure to the Investigational Product During Pregnancy or Breastfeeding, and Occupational Exposure

Although this study will be conducted exclusively in male patients, it is theoretically possible for female partners to be exposed to study treatment during pregnancy. Exposure to the investigational product under study during pregnancy or breastfeeding and occupational exposure are reportable to Pfizer Safety within 24 hours of investigator awareness.

8.4.3.1. Exposure During Pregnancy

For both unapproved/unlicensed products and for marketed products, an exposure during pregnancy (EDP) occurs if:

- A female becomes, or is found to be, pregnant either while receiving or having been exposed (eg, because of treatment or environmental exposure) to the investigational product; or the female becomes or is found to be pregnant after discontinuing and/or being exposed to the investigational product;
    - An example of environmental exposure would be a case involving direct contact with a Pfizer product in a pregnant woman (eg, a nurse reports that she is pregnant and has been exposed to chemotherapeutic products).
- A male has been exposed (eg, because of treatment or environmental exposure) to the investigational product prior to or around the time of conception and/or is exposed during his partner's pregnancy.

If a patient or patient's partner becomes or is found to be pregnant during the patient's treatment with the investigational product, the investigator must report this information to Pfizer Safety on the CT SAE Report Form and an EDP supplemental form, regardless of whether an SAE has occurred. In addition, the investigator must submit information regarding environmental exposure to a Pfizer product in a pregnant woman (eg, a patient reports that she is pregnant and has been exposed to a cytotoxic product by inhalation or spillage) to Pfizer Safety using the EDP supplemental form. This must be done irrespective of whether an AE has occurred and within 24 hours of awareness of the exposure. The information submitted should include the anticipated date of delivery (see below for information related to termination of pregnancy).

Follow-up is conducted to obtain general information on the pregnancy and its outcome for all EDP reports with an unknown outcome. The investigator will follow the pregnancy until completion (or until pregnancy termination) and notify Pfizer Safety of the outcome as a follow-up to the initial EDP supplemental form. In the case of a live birth, the structural integrity of the neonate can be assessed at the time of birth. In the event of a termination, the reason(s) for termination should be specified and, if clinically possible, the structural integrity of the terminated fetus should be assessed by gross visual inspection (unless pre-procedure test findings are conclusive for a congenital anomaly and the findings are reported).

090177e19c2fb240\Approved\Approved On: 23-Dec-2022 10:25 (GMT)

If the outcome of the pregnancy meets the criteria for an SAE (ie, ectopic pregnancy, spontaneous abortion, intrauterine fetal demise, neonatal death, or congenital anomaly [in a live-born baby, a terminated fetus, an intrauterine fetal demise, or a neonatal death]), the investigator should follow the procedures for reporting SAEs.

Additional information about pregnancy outcomes that are reported to Pfizer Safety as SAEs follows:

- Spontaneous abortion includes miscarriage and missed abortion;
- Neonatal deaths that occur within 1 month of birth should be reported, without regard to causality, as SAEs. In addition, infant deaths after 1 month should be reported as SAEs when the investigator assesses the infant death as related or possibly related to exposure to the investigational product.

Additional information regarding the EDP may be requested by the sponsor. Further follow-up of birth outcomes will be handled on a case-by-case basis (eg, follow-up on preterm infants to identify developmental delays). In the case of paternal exposure, the investigator will provide the patient with the Pregnant Partner Release of Information Form to deliver to his partner. The investigator must document in the source documents that the patient was given the Pregnant Partner Release of Information Form to provide to his partner.

8.4.3.2. Exposure During Breastfeeding

In female partners of study patients, scenarios of exposure during breastfeeding must be reported, irrespective of the presence of an associated SAE, to Pfizer Safety within 24 hours of the investigator's awareness, using the CT SAE Report Form. An exposure during breastfeeding report is not created when a Pfizer drug specifically approved for use in breastfeeding women (eg, vitamins) is administered in accord with authorized use. However, if the infant experiences an SAE associated with such a drug's administration, the SAE is reported together with the exposure during breastfeeding.

8.4.3.3. Occupational Exposure

An occupational exposure occurs when, during the performance of job duties, a person (whether a healthcare professional or otherwise) gets in unplanned direct contact with the product, which may or may not lead to the occurrence of an AE.

An occupational exposure is reported to Pfizer Safety within 24 hours of the investigator's awareness, using the CT SAE Report Form, regardless of whether there is an associated SAE. Since the information does not pertain to a patient enrolled in the study, the information is not recorded on a CRF; however, a copy of the completed CT SAE Report Form is maintained in the investigator site file.

8.4.4. Medication Errors

Other exposures to the investigational product under study may occur in clinical study settings, such as medication errors.

090177e19c2fb240\Approved\Approved On: 23-Dec-2022 10:25 (GMT)

Safety Event	Recorded on the CRF	Reported on the CT SAE Report Form to Pfizer Safety Within 24 Hours of Awareness
Medication errors	All (regardless of whether associated with an AE)	Only if associated with an SAE

8.4.4.1. Medication Errors

Medication errors may result from the administration or consumption of the investigational product (talazoparib, talazoparib/placebo, enzalutamide) by the wrong patient, or at the wrong time, or at the wrong dosage strength.

Medication errors include:

- Medication errors involving patient exposure to the investigational product:
  - Lack of dose reduction as specified by the protocol;
  - Continuation of treatment although patient met discontinuation or drug withholding criteria;
  - Incorrect study treatment dose taken by patient (any overdose or underdose);
  - Patient did not take study medication for 6 or more days (approximately <80%) within 4 weeks, unless dose withheld due to an AE;
  - Patient did not receive treatment as assigned by IWRS (Part 2 only).
- Potential medication errors or uses outside of what is foreseen in the protocol (or in the case of enzalutamide inconsistent with the Summary of Product Characteristics) that do or do not involve the participating patient.

Such medication errors occurring in a study participant are to be captured on the medication error page of the CRF, which is a specific version of the AE page.

In the event of a medication dosing error, the sponsor should be notified immediately.

Whether or not the medication error is accompanied by an AE, as determined by the investigator, the medication error is recorded on the medication error page of the CRF and, if applicable, any associated AE(s), serious and non-serious, are recorded on an AE page of the CRF.

Medication errors should be reported to Pfizer Safety within 24 hours on a CT SAE Report Form **only when associated with an SAE**.

090177e19c2fb240\Approved\Approved On: 23-Dec-2022 10:25 (GMT)

9. DATA ANALYSIS/STATISTICAL METHODS

Detailed methodology for summary and statistical analyses of the data collected in this study is outlined here and further detailed in a statistical analysis plan (SAP), which will be maintained by the sponsor. The SAP may modify what is outlined in the protocol where appropriate; however, any major modifications of the primary endpoint definitions or their analyses will also be reflected in a protocol amendment.

9.1. Sample Size Determination

The primary objective of Part 1 was to determine the starting dose of talazoparib when given in combination with enzalutamide during Part 2 (double-blind treatment period).

In Part 2 enrollment will begin in the all-comers population, Cohort 1. Once enrollment is complete in Cohort 1, enrollment will continue but will be restricted to patients with DDR deficiencies (Cohort 2).

The primary objectives of Part 2 are:

- To demonstrate that talazoparib in combination with enzalutamide is superior to placebo in combination with enzalutamide in prolonging BICR assessed rPFS in patients with mCRPC unselected for DDR status.
- To demonstrate that talazoparib in combination with enzalutamide is superior to placebo in combination with enzalutamide in prolonging BICR assessed rPFS in patients with mCRPC harboring DDR deficiencies.

The key secondary objectives of this study are:

- To demonstrate that talazoparib in combination with enzalutamide is superior to placebo in combination with enzalutamide in prolonging overall survival in patients with mCRPC unselected for DDR status.
- To demonstrate that talazoparib in combination with enzalutamide is superior to placebo in combination with enzalutamide in prolonging overall survival in patients with mCRPC harboring DDR deficiencies.

CCI [redacted]

090177e19c2fb240\Approved\Approved On: 23-Dec-2022 10:25 (GMT)

CCI

CCI

CCI

CCI

090177e19c2fb240\Approved\Approved On: 23-Dec-2022 10:25 (GMT)

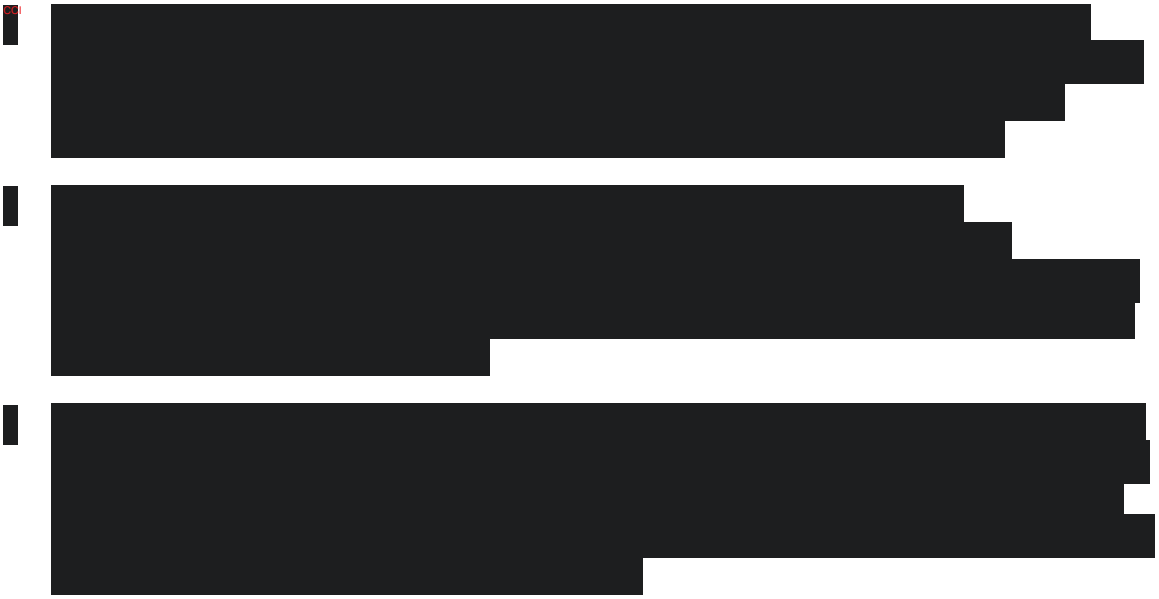

CCI

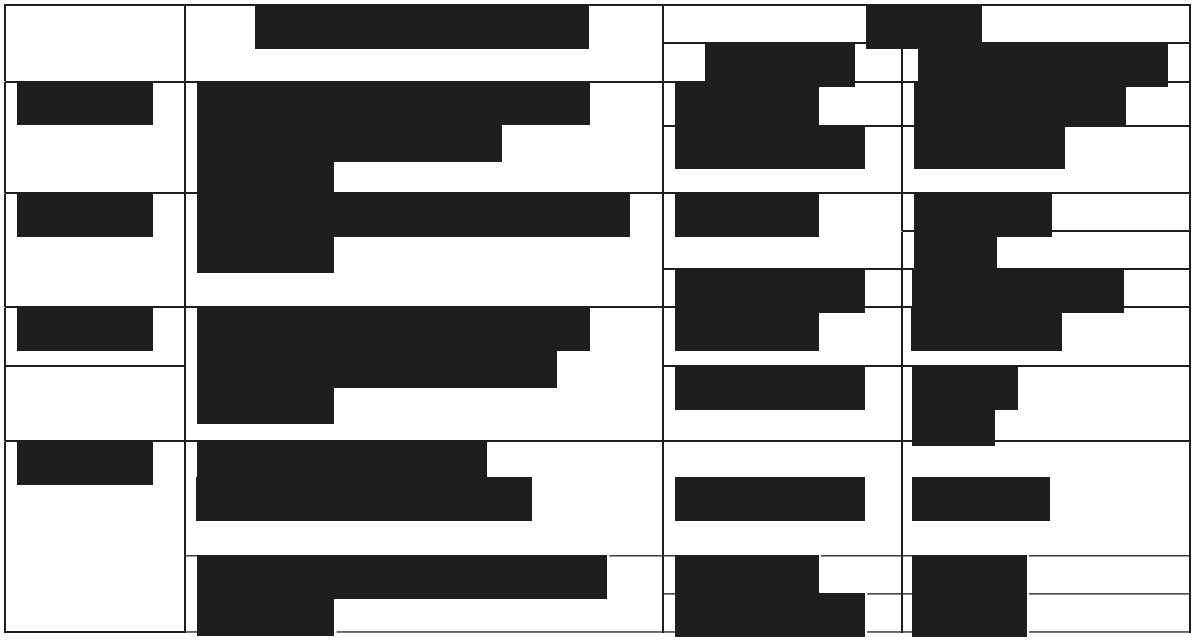

CCI

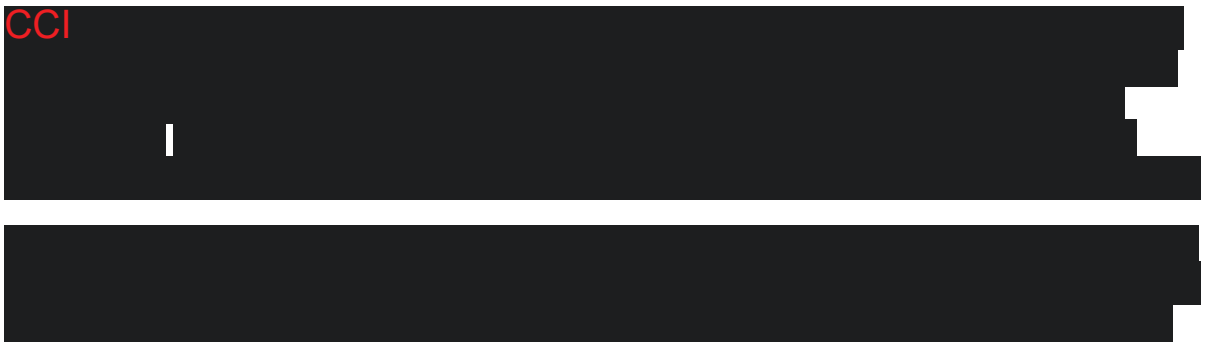

090177e19c2fb240\Approved\Approved On: 23-Dec-2022 10:25 (GMT)

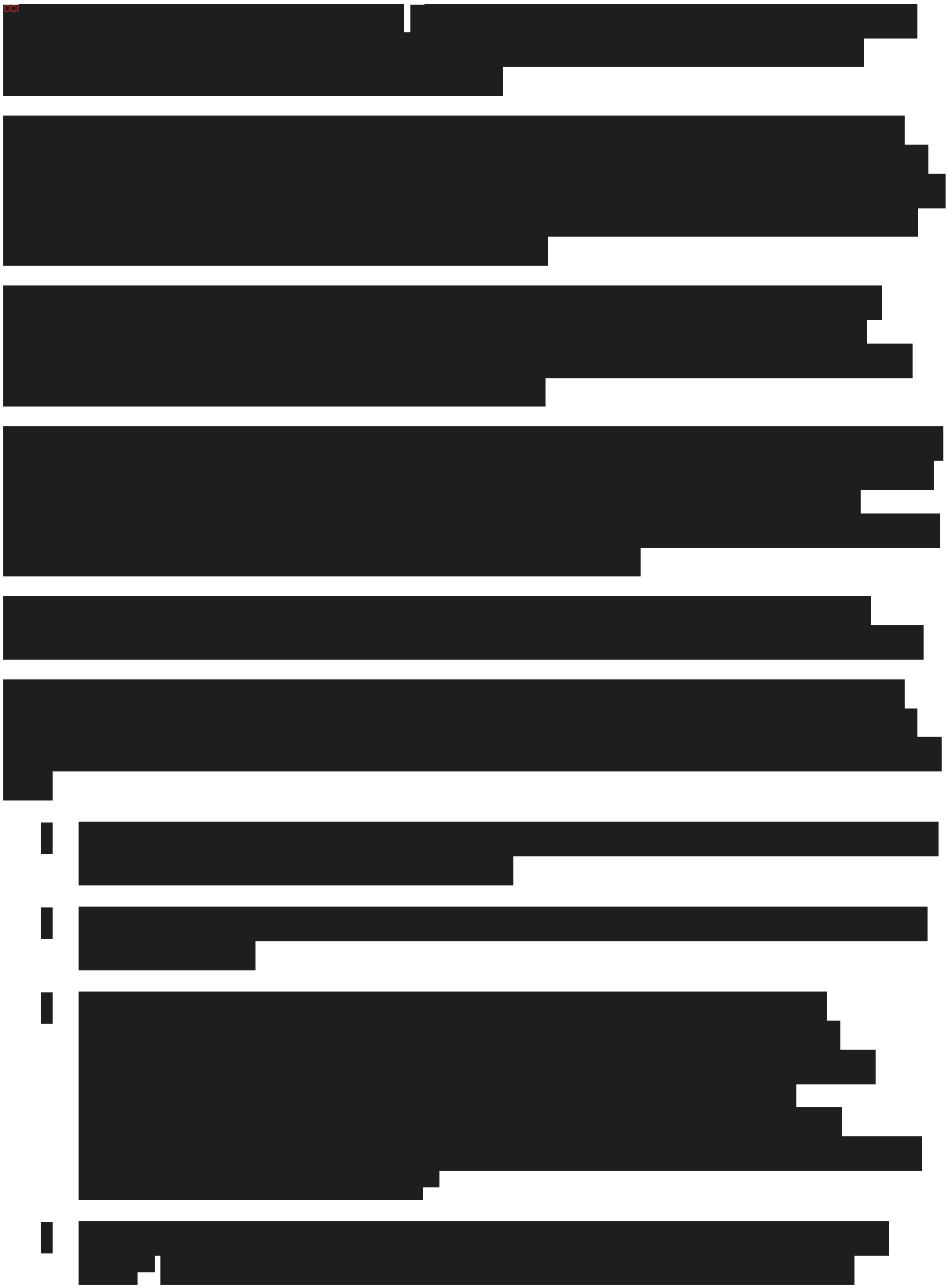

090177e19c2fb240\Approved\Approved On: 23-Dec-2022 10:25 (GMT)

CCI

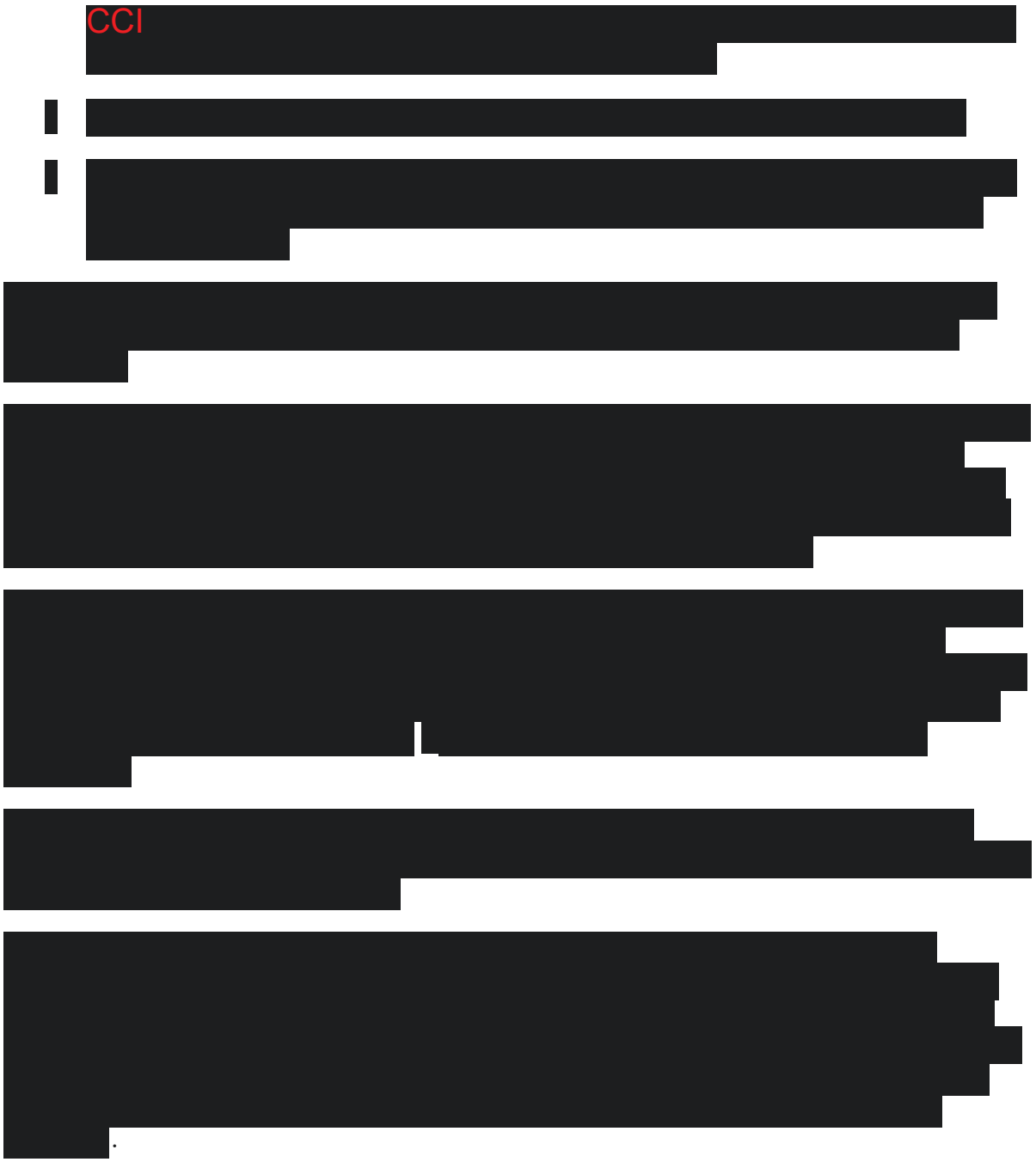

090177e19c2fb240\Approved\Approved On: 23-Dec-2022 10:25 (GMT)

9.2. Analysis Sets

Analyses on the all-comers population will include patients randomized into the first cohort of patients unselected for DDR status. Analysis on the DDR-deficient population will include patients with DDR-deficient disease enrolled into Cohort 2 as well as any patients within the first cohort with DDR-deficient disease. These populations will be subsets of the populations defined below.

Exploratory analyses of patients with mCRPC without DDR deficiencies will also be provided. These will be a subgroup of patients from the all comer population.

9.2.1. Intent-to-Treat Population

The intent-to-treat (ITT) population will be defined as all patients randomly assigned to double-blind study treatment in Part 2. All efficacy analyses will be conducted using the ITT population or a subset of the ITT population as appropriate. An example of the latter would be those in the ITT population who have measurable soft tissue disease at screening in the assessment of proportion of patients with soft tissue response.

Additional subsets will be described in the final SAP as appropriate.

090177e19c2fb240\Approved\Approved On: 23-Dec-2022 10:25 (GMT)

9.2.2. Safety Population

The safety population is defined as all patients who receive at least one dose of study treatment (open-label or double-blind). Study treatments include talazoparib, placebo, and enzalutamide administered as part of this study. Unless otherwise specified, all safety analyses will use the safety population according to the actual treatment received, not the treatment assigned. All safety data analyses will be conducted on the safety population.

9.2.3. Patient-Reported Outcome Population

The PRO population will be defined as a subset of ITT population who have completed a baseline and at least one post-baseline quality-of-life assessment prior to the end of study.

9.2.4. Pharmacokinetic Analysis Population

The PK analysis set (PAS) consists of all patients who received at least one dose of talazoparib or enzalutamide and provided an evaluable PK sample.

9.3. Efficacy Analysis

9.3.1. Primary Endpoint: Radiographic PFS

Radiographic PFS is defined as the time from the date of randomization to first objective evidence of radiographic progression as assessed in soft tissue per RECIST 1.1 or in bone (upon subsequent confirmation) per PCWG3 guidelines by BICR, or death, whichever occurs first, and will be summarized in months using the following calculation:

> rPFS (months) = [date of first objective evidence of radiographic progression (unconfirmed RECIST 1.1 or confirmed PCWG3), death, or censoring – randomization date +1]/30.4375 where date of bone disease progression is censored if not subsequently confirmed per PCWG3.

CCI

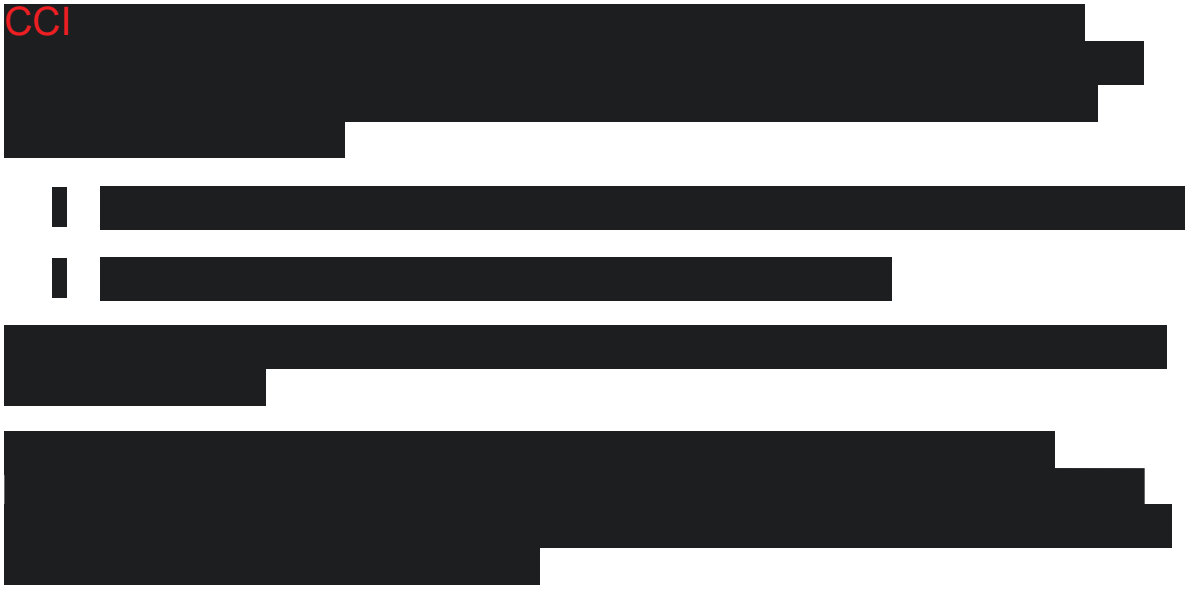

090177e19c2fb240\Approved\Approved On: 23-Dec-2022 10:25 (GMT)

CCI [redacted]

[redacted]

9.3.2. Analysis of Secondary Efficacy Endpoints

OS is defined as the time from randomization to the date of death due to any cause. Patients last known to be alive will be censored at the date of last contact. CCI [redacted]

Additional secondary efficacy endpoints will be evaluated separately in the all-comers population and in the DDR-deficient population. CCI [redacted]

Proportion of patients with measurable soft tissue disease at baseline who have a confirmed objective response per RECIST 1.1 will be summarized in the all-comers population and in the DDR-deficient population. Soft tissue responses must be confirmed by a follow-up radiographic assessment at least 4 weeks later with no evidence of **confirmed bone disease progression on repeated bone scans at least 6 weeks apart per PCWG3 criteria**. Patients without documented CR or PR will be considered non-responders. The primary evaluation of objective response rate (ORR) will be based on BICR overall tumor assessment. An evaluation of ORR based on derived investigator assessment using RECIST 1.1 will also be performed.

CCI [redacted]

Time from the date of randomization to the date of the first PSA progression will also be analyzed. CCI [redacted]

090177e19c2fb240\Approved\Approved On: 23-Dec-2022 10:25 (GMT)

The time from the first objective evidence of a CR or PR to the first objective evidence of disease progression (assessed in soft tissue per RECIST 1.1 or in bone upon subsequent confirmation per PCWG3 guidelines) or death, whichever occurs first, will be analyzed in the subset of patients entering with measurable soft tissue disease.

The time from randomization to first objective evidence of spinal cord compression, symptomatic pathologic bone fracture, or radiation or surgery to bone; time from randomization to the initiation of antineoplastic therapy; and time to opiate use for prostate cancer pain will be analyzed using statistical methods defined in the SAP.

PFS2 is defined as time from randomization to investigator documented disease progression (PSA progression, progression on imaging, or clinical progression) on the first subsequent antineoplastic therapy for prostate cancer, or death from any cause, whichever occurs first.

9.3.3. Analysis of Exploratory Endpoints

Biomarkers will be assessed separately for blood, CTCs, tumor tissue, and saliva collected during Part 1 and Part 2. In each case, summaries of baseline levels, changes from baseline (where appropriate), expression, and mutation will be reported as applicable. CCI

[redacted]

9.4. Patient-Reported Outcome Analyses

The PRO population will be defined as the subset of ITT patients in Part 2 who have completed a baseline and at least one post-baseline quality-of-life assessment prior to the end of study. Missing items will be handled per the scoring manuals of each questionnaire administered.

Pain is measured using the BPI-SF Question #3. Pain and analgesic assessments will be completed (via pain and analgesic logs) for 7 consecutive days before each study visit. Analgesic data will be mapped to the WHO analgesic usage score so that an analgesic score may be calculated for each of the 7 days. The pain score and the analgesic usage score for each visit is the mean score based on the corresponding scores on the day of the visit and the 7 days prior to the visit, assuming at least 4 of these entries are non-missing. They are considered missing otherwise. Deterioration in pain is defined as ⩾2-point increase from the baseline pain score.

Definitive deterioration in patient-reported global health status/QoL is defined as a ≥10-point decrease from baseline and no subsequent observations with a <10-point decrease from baseline assessed by the EORTC QLQ-C30.[42]

090177e19c2fb240\Approved\Approved On: 23-Dec-2022 10:25 (GMT)

Definitive deterioration in patient-reported urinary symptoms is defined as a ≥10-point increase from baseline and no subsequent observations with a <10-point increase from baseline assessed by the EORTC QLQ-PR25.[41]

CCI [redacted]

[redacted].

9.5. Pharmacokinetic Analyses

For patients who participate in Part 1, the final PK data analyses will include calculation of multiple dose PK parameters (as specified in secondary endpoints for Part 1) of talazoparib, enzalutamide, and its N-desmethyl metabolite. These data will be summarized using descriptive statistics and compared with historic data of talazoparib and enzalutamide when given alone to characterize PK and evaluate the DDI effect. Non-Compartmental Analysis (NCA) will be used for Part 1. Population pharmacokinetic (PopPK) analysis may be used for both Part 1 and Part 2 if the data allows.

For all patients (Part 1 and Part 2), PK data analyses will include descriptive summary statistics of the pre-dose and post-dose plasma concentrations of talazoparib, enzalutamide and its N-desmethyl metabolite by study visit and treatment arm.

In addition, the PK data from this study may be used to develop a population PK model. The correlations between talazoparib exposure parameters in combination with enzalutamide and biomarker, efficacy and safety endpoints will be explored if data allows. The results of these modeling analyses will be reported separately from the clinical study report.

The analytical laboratory, and Pfizer clinical assay group (CAG) personnel will be unblinded to study treatment assignment; therefore, only talazoparib PK samples collected from patients randomized to the talazoparib + enzalutamide arm will be analyzed. All collected enzalutamide PK samples will be analyzed. If the need arises for early analysis of the PK data (before database lock and release of the randomization codes for the study), an early PK unblinding plan will be developed. PK analyst(s) and supporting colleagues who are not associated with the study team, will conduct the analysis to avoid unblinding of the study team.

090177e19c2fb240\Approved\Approved On: 23-Dec-2022 10:25 (GMT)

9.6. Biomarker Analyses

Data from biomarker assays will be summarized and analyzed using appropriate methods such as the Wilcoxon signed rank test. CCI [redacted] Graphic displays will be presented. Biomarker results from Part 1 and Part 2 may also be combined for similar statistical analyses as warranted.

090177e19c2fb240\Approved\Approved On: 23-Dec-2022 10:25 (GMT)

9.7. Safety Analysis

Safety will be evaluated for the safety population using adverse event, laboratory and vital signs data. Treatment-emergent safety data will be defined as events from the first dose of study treatment through 28 days after permanent discontinuation of all study-treatments (talazoparib, talazoparib/placebo, or enzalutamide), or before initiation of a new antineoplastic or any investigational therapy, whichever occurs first.

9.7.1. Adverse Events

All analyses will be based on treatment-emergent events unless otherwise specified; events not considered treatment-emergent will be flagged in data listings.

All adverse events will be coded to preferred term and system organ class using current Medical Dictionary for Regulatory Activities (MedDRA). AEs will be presented with and without regard to causality based on the investigator's judgment and by frequency of overall toxicity categorized by NCI CTCAE (version 4.03) grades. Summary tables will include system organ class and/or preferred term; these will be described in the full SAP.

In case a patient has events with missing and non-missing toxicity grades, the maximum of the non-missing grade will be displayed. Missing grade will only be displayed in the event that only one event has been reported for a patient and the grade is missing.

9.7.2. Analysis of Clinical Laboratory Results

If necessary, laboratory results will be converted to International System of Units (Système International d'unités, SI) units which will be used for applying toxicity grades and for all summaries.

Baseline will be defined as the latest assessment performed prior to the first dose of study treatment (or latest assessment prior to randomization for patients randomized but not dosed). If there are multiple assessments that meet the baseline definition on the same day without the ability to determine which was truly last, then the worst grade will be assigned as the baseline grade.

Laboratory results will be programmatically classified according to NCI CTCAE version 4.03. Abnormalities will be described using the worst grade by scheduled time point and overall.

Additional laboratory results that are not part of NCI CTCAE version 4.03 will be presented according to the following categories by scheduled time point as well as overall: below normal limit, within normal limits, and above normal limits.

Laboratory shift tables of baseline to maximum post-baseline results to each subsequent visit will be produced as appropriate.

090177e19c2fb240\Approved\Approved On: 23-Dec-2022 10:25 (GMT)

9.8. Interim Analysis

CCI

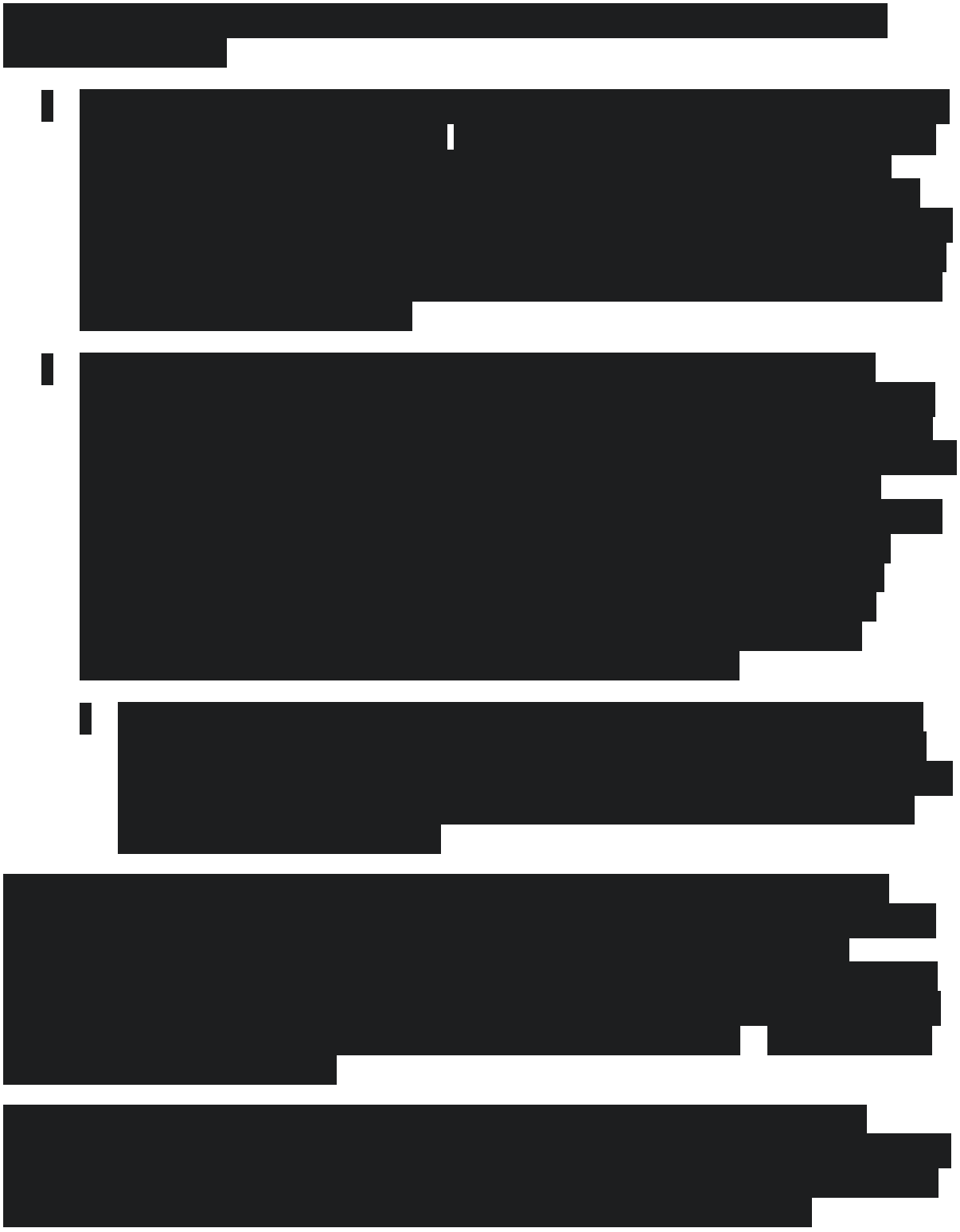

090177e19c2fb240\Approved\Approved On: 23-Dec-2022 10:25 (GMT)

CCI [redacted]

Interim analysis results for the rPFS endpoints in all-comers and DDR-deficient populations will be assessed by the E-DMC.

CCI [redacted]

9.9. Data Monitoring Committee

Part 2 of this study will use an independent external data monitoring committee (E-DMC) independent of the sponsor. The E-DMC will be responsible for ongoing monitoring of the efficacy and safety of patients in the study according to the charter. The recommendations made by the E-DMC to alter the conduct of the study will be forwarded to Pfizer for final decision. Pfizer will forward such decisions, which may include summaries of aggregate analyses of endpoint events and of safety data that are not endpoints, to regulatory authorities, as appropriate.

10. QUALITY CONTROL AND QUALITY ASSURANCE

Pfizer or its agent will conduct periodic monitoring visits during study conduct to ensure that the protocol and Good Clinical Practices (GCPs) are being followed. The monitors may review source documents to confirm that the data recorded on CRFs are accurate. The investigator and institution will allow Pfizer monitors/auditors or its agents and appropriate regulatory authorities direct access to source documents to perform this verification. This verification may also occur after study completion.

During study conduct and/or after study completion, the investigator site may be subject to review by the IRB/EC, and/or to quality assurance audits performed by Pfizer, or companies working with or on behalf of Pfizer, and/or to inspection by appropriate regulatory authorities.

The investigator(s) will notify Pfizer or its agents immediately of any regulatory inspection notification in relation to the study. Furthermore, the investigator will cooperate with Pfizer or its agents to prepare the investigator site for the inspection and will allow Pfizer or its agent, whenever feasible, to be present during the inspection. The investigator site and investigator will promptly resolve any discrepancies that are identified between the study

090177e19c2fb240\Approved\Approved On: 23-Dec-2022 10:25 (GMT)

data and the patient's medical records. The investigator will promptly provide copies of the inspection findings to Pfizer or its agent. Before response submission to the regulatory authorities, the investigator will provide Pfizer or its agents with an opportunity to review and comment on responses to any such findings.

It is important that the investigator(s) and their relevant personnel are available during the monitoring visits and possible audits or inspections and that sufficient time is devoted to the process.

11. DATA HANDLING AND RECORD KEEPING

11.1. Case Report Forms/Electronic Data Record

As used in this protocol, the term CRF should be understood to refer to either a paper form or an electronic data record or both, depending on the data collection method used in this study.

A CRF is required and should be completed for each included patient. The completed original CRFs are the sole property of Pfizer and should not be made available in any form to third parties, except for authorized representatives of Pfizer or appropriate regulatory authorities, without written permission from Pfizer. The investigator shall ensure that the CRFs/DCTs are securely stored at the study site in encrypted electronic and/or paper form and will be password protected or secured in a locked room to prevent access by unauthorized third parties.

The investigator has ultimate responsibility for the collection and reporting of all clinical, safety, and laboratory data entered on the CRFs and any other data collection forms (source documents) and ensuring that they are accurate, authentic/original, attributable, complete, consistent, legible, timely (contemporaneous), enduring, and available when required. The CRFs must be signed by the investigator or by an authorized staff member to attest that the data contained on the CRFs are true. Any corrections to entries made in the CRFs or source documents must be dated, initialed, and explained (if necessary) and should not obscure the original entry.

In most cases, the source documents are the hospital or the physician patient chart. In these cases, data collected on the CRFs must match the data in those charts.

In some cases, the CRF may also serve as the source document. In these cases, a document should be available at the investigator site and at Pfizer that clearly identifies those data that will be recorded on the CRF, and for which the CRF will stand as the source document.

11.2. Record Retention

To enable evaluations and/or inspections/audits from regulatory authorities or Pfizer, the investigator agrees to keep records, including the identity of all participating patients (sufficient information to link records, eg, CRFs and hospital records), all original signed informed consent documents, copies of all CRFs, safety reporting forms, source documents, and detailed records of treatment disposition, and adequate documentation of relevant correspondence (eg, letters, meeting minutes, and telephone call reports). The records should

090177e19c2fb240\Approved\Approved On: 23-Dec-2022 10:25 (GMT)

be retained by the investigator according to the ICH guidelines, according to local regulations, or as specified in the clinical study agreement (CSA), whichever is longer. The investigator must ensure that the records continue to be stored securely for so long as they are retained.

If the investigator becomes unable for any reason to continue to retain study records for the required period (eg, retirement, relocation), Pfizer should be prospectively notified. The study records must be transferred to a designee acceptable to Pfizer, such as another investigator, another institution, or an independent third party arranged by Pfizer.

Investigator records must be kept for a minimum of 15 years after completion or discontinuation of the study or for longer if required by applicable local regulations.

The investigator must obtain Pfizer's written permission before disposing of any records, even if retention requirements have been met.

12. ETHICS

12.1. Institutional Review Board/Ethics Committee

It is the responsibility of the investigator to have prospective approval of the study protocol, protocol amendments, informed consent documents, and other relevant documents, eg, recruitment advertisements, if applicable, from the IRB/EC. All correspondence with the IRB/EC should be retained in the investigator file. Copies of IRB/EC approvals should be forwarded to Pfizer.

The only circumstance in which an amendment may be initiated prior to IRB/EC approval is where the change is necessary to eliminate apparent immediate hazards to the patients. In that event, the investigator must notify the IRB/EC and Pfizer in writing immediately after the implementation.

12.2. Ethical Conduct of the Study

The study will be conducted in accordance with the protocol, legal and regulatory requirements, and the general principles set forth in the International Ethical Guidelines for Biomedical Research Involving Human Subjects (Council for International Organizations of Medical Sciences 2002), ICH Guideline for Good Clinical Practice, and the Declaration of Helsinki.

12.3. Patient Information and Consent

All parties will comply with all applicable laws, including laws regarding the implementation of organizational and technical measures to ensure protection of subject personal data. Such measures will include omitting subject names or other directly identifiable data in any reports, publications, or other disclosures, except where required by applicable laws.

The personal data will be stored at the study site in [encrypted electronic and/or paper] form and will be [password protected or secured in a locked room] to ensure that only authorized study staff have access. The study site will implement appropriate technical and

090177e19c2fb240\Approved\Approved On: 23-Dec-2022 10:25 (GMT)

organizational measures to ensure that the personal data can be recovered in the event of disaster. In the event of a potential personal data breach, the study site shall be responsible for determining whether a personal data breach has in fact occurred and, if so, providing breach notifications as required by law.

To protect the rights and freedoms of natural persons with regard to the processing of personal data, when study data are compiled for transfer to Pfizer and other authorized parties, subject names will be removed and will be replaced by a single, specific, numerical code, based on a numbering system defined by Pfizer. *All other identifiable data transferred to Pfizer or other authorized parties will be identified by this single, subject-specific code.* The investigator site will maintain a confidential list of subjects who participated in the study, linking each subject's numerical code to his or her actual identity. In case of data transfer, Pfizer will maintain high standards of confidentiality and protection of subjects' personal data consistent with the Clinical Study Agreement and applicable privacy laws.

The informed consent documents and any subject recruitment materials must be in compliance with ICH GCP, local regulatory requirements, and legal requirements, including applicable privacy laws.

The informed consent documents used during the informed consent process and any subject recruitment materials must be reviewed and approved by Pfizer, approved by the IRB/EC before use, and available for inspection.

The investigator must ensure that each study subject, or his or her legally acceptable representative, is fully informed about the nature and objectives of the study, the sharing of data relating to the study and possible risks associated with participation, including the risks associated with the processing of the subject's personal data. The investigator further must ensure that each study subject, or his or her legally acceptable representative, is fully informed about his or her right to access and correct his or her personal data and to withdraw consent for the processing of his or her personal data.

Whenever consent is obtained from a patient's legally acceptable representative or legal guardian, the patient's assent (affirmative agreement) must subsequently be obtained when the patient has the capacity to provide assent, as determined by the IRB/EC. If the investigator determines that a patient's decisional capacity is so limited he/she cannot reasonably be consulted, then, as permitted by the IRB/EC and consistent with local regulatory and legal requirements, the patient's assent may be waived with source documentation of the reason assent was not obtained. If the study patient does not provide his or her own consent, the source documents must record why the patient did not provide consent (eg, minor, decisionally impaired adult), how the investigator determined that the person signing the consent was the patient's legally acceptable representative, the consent signer's relationship to the study patient (eg, parent, spouse), and that the patient's assent was obtained or waived. If assent is obtained verbally, it must be documented in the source documents.

090177e19c2fb240\Approved\Approved On: 23-Dec-2022 10:25 (GMT)

The investigator, or a person designated by the investigator, will obtain written informed consent from each patient before any study-specific activity is performed. The investigator will retain the original of each patient's signed consent document.

12.4. Reporting of Safety Issues and Serious Breaches of the Protocol or ICH GCP

In the event of any prohibition or restriction imposed (ie, clinical hold) by an applicable regulatory authority in any area of the world, or if the investigator is aware of any new information that might influence the evaluation of the benefits and risks of the investigational product, Pfizer should be informed immediately.

In addition, the investigator will inform Pfizer immediately of any urgent safety measures taken by the investigator to protect the study patients against any immediate hazard, and of any serious breaches of this protocol or of ICH GCP that the investigator becomes aware of.

13. DEFINITION OF END OF STUDY

13.1. End of Study in All Participating Countries

End of study in all participating countries is defined as last patient last visit (LPLV).

14. SPONSOR DISCONTINUATION CRITERIA

Premature termination of this study may occur because of a regulatory authority decision, change in opinion of the IRB/EC, or investigational product safety problems, or at the discretion of Pfizer. In addition, Pfizer retains the right to discontinue development of talazoparib at any time.

If a study is prematurely terminated, Pfizer will promptly notify the investigator. After notification, the investigator must contact all participating patients and the hospital pharmacy (if applicable) as quickly as is practical. As directed by Pfizer, all study materials must be collected and all CRFs completed to the greatest extent possible.

15. PUBLICATION OF STUDY RESULTS

15.1. Communication of Results by Pfizer

Pfizer fulfills its commitment to publicly disclose clinical study results through posting the results of studies on www.clinicaltrials.gov (ClinicalTrials.gov), the European Clinical Trials Database (EudraCT), and/or www.pfizer.com, and other public registries in accordance with applicable local laws/regulations.

In all cases, study results are reported by Pfizer in an objective, accurate, balanced, and complete manner and are reported regardless of the outcome of the study or the country in which the study was conducted.

www.clinicaltrials.gov

Pfizer posts clinical study US Basic Results on www.clinicaltrials.gov for Pfizer-sponsored interventional studies (conducted in patients) that evaluate the safety and/or efficacy of a

090177e19c2fb240\Approved\Approved On: 23-Dec-2022 10:25 (GMT)

Pfizer product, regardless of the geographical location in which the study is conducted. US Basic Results are submitted for posting within 1 year of the primary completion date (PCD) for studies in adult populations or within 6 months of the PCD for studies in pediatric populations.

PCD is defined as the date that the final patient was examined or received an intervention for the purposes of final collection of data for the primary outcome, whether the clinical study concluded according to the prespecified protocol or was terminated.

EudraCT

Pfizer posts European Union (EU) Basic Results on EudraCT for all Pfizer-sponsored interventional studies that are in scope of EU requirements. EU Basic Results are submitted for posting within 1 year of the PCD for studies in adult populations or within 6 months of the PCD for studies in pediatric populations.

www.pfizer.com

Pfizer posts Public Disclosure Synopses (clinical study report synopses in which any data that could be used to identify individual patients has been removed) on www.pfizer.com for Pfizer-sponsored interventional studies at the same time the US Basic Results document is posted to www.clinicaltrials.gov.

15.2. Publications by Investigators

Pfizer supports the exercise of academic freedom and has no objection to publication by the principal investigator (PI) of the results of the study based on information collected or generated by the PI, whether or not the results are favorable to the Pfizer product. However, to ensure against inadvertent disclosure of confidential information or unprotected inventions, the investigator will provide Pfizer an opportunity to review any proposed publication or other type of disclosure of the results of the study (collectively, "publication") before it is submitted or otherwise disclosed.

The investigator will provide any publication to Pfizer at least 30 days before it is submitted for publication or otherwise disclosed. If any patent action is required to protect intellectual property rights, the investigator agrees to delay the disclosure for a period not to exceed an additional 60 days.

The investigator will, on request, remove any previously undisclosed confidential information before disclosure, except for any study- or Pfizer product-related information necessary to the appropriate scientific presentation or understanding of the study results.

If the study is part of a multicenter study, the investigator agrees that the first publication is to be a joint publication covering all investigator sites, and that any subsequent publications by the PI will reference that primary publication. However, if a joint manuscript has not been submitted for publication within 12 months of completion or termination of the study at

090177e19c2fb240\Approved\Approved On: 23-Dec-2022 10:25 (GMT)

all participating sites, the investigator is free to publish separately, subject to the other requirements of this section.

For all publications relating to the study, the institution will comply with recognized ethical standards concerning publications and authorship, including Section II – "Ethical Considerations in the Conduct and Reporting of Research" of the Uniform Requirements for Manuscripts Submitted to Biomedical Journals, http://www.icmje.org/index.html#authorship, established by the International Committee of Medical Journal Editors.

Publication of study results is also provided for in the CSA between Pfizer and the institution. In this section entitled Publications by Investigators, the defined terms shall have the meanings given to them in the CSA.

If there is any conflict between the CSA and any attachments to it, the terms of the CSA control. If there is any conflict between this protocol and the CSA, this protocol will control as to any issue regarding treatment of study patients, and the CSA will control as to all other issues.

090177e19c2fb240\Approved\Approved On: 23-Dec-2022 10:25 (GMT)

16. REFERENCES

1. Murai J, Huang SY, Das BB, Renaud A, Zhang Y, Doroshow JH, et al. Trapping of PARP1and PARP2 by clinical PARP inhibitors. Cancer Res. 2012 Nov 1;72(21):5588-99.

2. American Cancer Society. Cancer facts & figures 2017 [Internet]. 2017 [cited 2017 Jun 27]. Available from: https://www.cancer.org/content/dam/cancer-org/research/cancer-facts-and-statistics/annual-cancer-facts-and-figures/2017/cancer-facts-and-figures-2017.pdf.

3. Ferlay J, Steliarova Foucher E, Lortet Tieulent J, Rosso S, Coebergh JW, Comber H, et al. Cancer incidence and mortality patterns in Europe: estimates for 40 countries in 2012. Eur J Cancer. 2013 Apr;49(6):1374 403.

4. Robinson D, Van Allen EM, Wu YM, Schultz N, Lonigro RJ, Mosquera JM, et al. Integrative clinical genomics of advanced prostate cancer. Cell. 2015 May 21;161(5):1215 28.

5. Tran C, Ouk S, Clegg NJ, Chen Y, Watson PA, Arora V, et al. Development of a second generation antiandrogen for treatment of advanced prostate cancer. Science. 2009 May 8;324(5928):787 90.

6. Attard G, Reid AH, Yap TA, Raynaud F, Dowsett M, Settatree S, et al. Phase I clinical trial of a selective inhibitor of CYP17, abiraterone acetate, confirms that castration resistant prostate cancer commonly remains hormone driven. J Clin Oncol. 2008 Oct 1;26(28):4563 71. Erratum in J Clin Oncol. 2012 May 20;30(15):1896.

7. de Bono JS, Logothetis CJ, Molina A, Fizazi K, North S, Chu L, et al. Abiraterone and increased survival in metastatic prostate cancer. N Engl J Med. 2011 May 26;364(21):1995 2005.

8. Stanbrough M, Bubley GJ, Ross K, Golub TR, Rubin MA, Penning TM, et al. Increased expression of genes converting adrenal androgens to testosterone in androgen independent prostate cancer. Cancer Res. 2006 Mar;66(5):2815 25.

9. Holzbeierlein J, Lal P, LaTulippe E, Smith A, Satagopan J, Zhang L, et al. Gene expression analysis of human prostate carcinoma during hormonal therapy identifies androgen responsive genes and mechanisms of therapy resistance. Am J Pathol. 2004 Jan;164(1):217 27.

10. Ryan CJ, Smith MR, Fizazi K, Saad F, Mulders PF, Sternberg CN, et al. Abiraterone acetate plus prednisone versus placebo plus prednisone in chemotherapy-naive men with metastatic castration-resistant prostate cancer (COU AA 302): final overall survival analysis of a randomised, double blind, placebo controlled phase 3 study. Lancet Oncol. 2015 Feb;16(2):152 60.

090177e19c2fb240\Approved\Approved On: 23-Dec-2022 10:25 (GMT)

11. Beer TM, Armstrong AJ, Rathkopf DE, Loriot Y, Sternberg CN, Higano CS, et al. Enzalutamide in metastatic prostate cancer before chemotherapy. N Engl J Med. 2014 Jul 31;371(5):424-33.

12. Scher HI, Fizazi K, Saad F, Taplin ME, Sternberg CN, Miller K, et al. Increased survival with enzalutamide in prostate cancer after chemotherapy. N Engl J Med. 2012 Sep 27;367(13):1187 97.

13. Fizazi K, Tran N, Fein LE, et al., LATITUDE: A phase III, double-blind, randomized trial of androgen deprivation therapy with abiraterone acetate plus prednisone or placebos in newly diagnosed high-risk metastatic hormone-naive prostate cancer. J Clin Oncol 2017;35(suppl):Abstract LBA3.

14. James ND, de Bono JS, Spears MR, et al. Adding abiraterone for men with high-risk prostate cancer (PCa) starting long-term androgen deprivation therapy (ADT): Survival results from STAMPEDE (NCT00268476). J Clin Oncol 2017;35(suppl):Abstract LBA5003.

15. https://newsroom.astellas.us/2017-09-14-Pfizer-and-Astellas-Announce-Positive-Top-Line-Results-from-Phase-3-PROSPER-Trial-of-XTANDI-enzalutamide-in-Patients-with-Non-Metastatic-Castration-Resistant-Prostate-Cancer.

16. Tannock IF, de Wit R, Berry WR, Horti J, Pluzanska A, Chi KN, et al. Docetaxel plus prednisone or mitoxantrone plus prednisone for advanced prostate cancer. N Engl J Med. 2004 Oct 7;351(15):1502 12.

17. de Bono JS, Oudard S, Ozguroglu M, Hansen S, Machiels JP, Kocak I, et al. Prednisone plus cabazitaxel or mitoxantrone for metastatic castration resistant prostate cancer progressing after docetaxel treatment: a randomised open label trial. Lancet. 2010 Oct 2;376(9747):1147 54.

18. Al Nakouzi N, Le Moulec S, Algibes L, et al. Cabazitaxel Remains Active in Patients Progressing After Docetaxel Followed by Novel Androgen Receptor Pathway Targeted Therapies. Eur Urol 2015;68(2):228-35.

19. de Bono JS, Smith MR, Saad F, et al. Subsequent Chemotherapy and Treatment Patterns After Abiraterone Acetate in Patients with Metastatic Castration-resistant Prostate Cancer: Post Hoc Analysis of COU-AA-302. Eur Urol 2017;71(4):656-64.

090177e19c2fb240\Approved\Approved On: 23-Dec-2022 10:25 (GMT)

20. James ND, Sydes MR, Clarke NW, Mason MD, Dearnaley DP, Spears MR, et al. Addition of docetaxel, zoledronic acid, or both to first-line long-term hormone therapy in prostate cancer (STAMPEDE): survival results from an adaptive, multiarm, multistage, platform randomised controlled trial. Addition of docetaxel, zoledronic acid, or both to first-line long-term hormone therapy in prostate cancer (STAMPEDE): survival results from an adaptive, multiarm, multistage, platform randomised controlled trial. Addition of docetaxel, zoledronic acid, or both to first-line long-term hormone therapy in prostate cancer (STAMPEDE): survival results from an adaptive, multiarm, multistage, platform randomised controlled trial. Lancet. 2016 Mar 19;387(10024):1163-77.

21. Sweeney CJ, Chen YH, Carducci M, Liu G, Jarrard DF, Eisenberger M, et al. Chemohormonal Therapy in Metastatic Hormone-Sensitive Prostate Cancer. N Engl J Med. 2015 Aug 20;373(8):737-46.

22. Vale C, Burdett S, Rydzewska LHM, et al. Addition of docetaxel or bisphosphonates to standard of care in men with localised or metastatic, hormone-sensitive prostate cancer: a systematic review and meta-analyses of aggregate data. Lancet Oncol 2016;17(2):243-256.

23. Parker C, Nilsson S, Heinrich D, Helle SI, O'Sullivan JM, Fosså SD, et al. Alpha emitter radium 223 and survival in metastatic prostate cancer. N Engl J Med. 2013 Jul 18;369(3):213 23.

24. Sipuleucel – T [package insert]. Seattle, WA: Dendreon Corporation; 2014. Available at http://www.valeant.com/Portals/25/Pdf/PI/Provenge-PI.pdf. 26 June 2017.

25. Schreiber V, Dantzer F, Amé JC, de Murcia G. Poly(ADP ribose): novel functions for an old molecule. Nat Rev Mol Cell Biol. 2006 Jul;7(7):517 28.

26. Curtin NJ. PARP inhibitors for cancer therapy. Expert Rev Mol Med. 2005 Mar 15;7(4):1 20.

27. Gibson BA, Kraus WL. New insights into the molecular and cellular functions of poly(ADP ribose) and PARPs. Nat Rev Mol Cell Biol. 2012 Jun 20;13(7):411-24.

28. Schreiber V, Amé JC, Dollé P, Schultz I, Rinaldi B, Fraulob V, et al. Poly(ADP ribose) polymerase 2 (PARP 2) is required for efficient base excision DNA repair in association with PARP 1 and XRCC1. J Biol Chem. 2002 Jun 21;277(25):23028 36.

29. Amé JC, Rolli V, Schreiber V, Niedergang C, Apiou F, Decker P, et al. PARP 2, a novel mammalian DNA damage dependent poly(ADP ribose) polymerase. J Biol Chem. 1999 Jun 18;274(25):17860 8.

30. Johansson M. A human poly(ADP ribose) polymerase gene family (ADPRTL): cDNA cloning of two novel poly(ADP ribose) polymerase homologues. Genomics. 1999 May 1;57(3):442 5.

090177e19c2fb240\Approved\Approved On: 23-Dec-2022 10:25 (GMT)

31. Hopkins TA, Shi Y, Rodriguez LE, Solomon LR, Donawho CK, DiGiammarino EL, et al. Mechanistic dissection of PARP1 trapping and the impact on in vivo tolerability and efficacy of PARP inhibitors. Mol Cancer Res. 2015 Nov;13(11):1465 77.

32. Lord et al. (2017) Science; 355 (6330): 1152-1158.

33. Mateo J, Carreira S, Sandhu S, Miranda S, Mossop H, Perez Lopez R, et al. DNA repair defects and olaparib in metastatic prostate cancer. N Engl J Med. 2015 Oct 29;373(18):1697 708.

34. Abida W, Curtis KR, Taylor BS, Arcila ME, Brennan R, Danila DC, et al. Genomic characterization of primary and metastatic prostate cancer (PC) using a targeted next generation sequencing assay [abstract]. J Clin Oncol. 2015 May 18;33(15 Suppl):5062.

35. Clarke N, Wiechno P, Alekseev B, et al. Olaparib combined with abiraterone in patients with metastatic castration-resistant prostate cancer: a randomised, double-blind, placebo-controlled, phase 2 trial. Lancet Oncol 2018; 19:975-86.

36. Development Safety Update Report, Talazoparib (PF-06944076), 20 Jan 2017.

37. Ryan CJ, Smith MR, de Bono JS, Molina A, Logothetis CJ, de Souza P, et al. Abiraterone in metastatic prostate cancer without previous chemotherapy. N Engl J Med. 2013 Jan 10;368(2):138-48.

38. European public assessment report (EPAR) for Xtandi (enzalutamide). Accessed at http://www.ema.europa.eu/ema/index.jsp?curl=pages/medicines/human/medicines/002639/human_med_001663.jsp&mid=WC0b01ac058001d124. 12 May 2017.

39. European public assessment report (EPARs) for Zytiga (abiraterone). Accessed at http://www.ema.europa.eu/ema/index.jsp?curl=pages/medicines/human/medicines/002321/human_med_001499.jsp&mid=WC0b01ac058001d124. 12 May 2017.

40. Amit O, Mannino F, Stone AM, et al. Blinded Independent Central Review of Progression in Cancer Clinical Trials: Results from a Meta-analysis. European Journal of Cancer 2011; 47: 1772-8.

41. Mathias SD, Crosby RD, Qian Y, et al. Estimating minimally important differences for the worst pain rating of the Brief Pain Inventory-Short Form. J Support Oncol 2011;9(2):72-8.

42. Osoba D, Rodrigues G, Myles J, et al. Interpreting the significance of changes in health related quality-of-life scores. J Clin Oncol 1998;16(1):139-44.

43. Li L, Karanika S, Yang G, et al. Androgen receptor inhibitor-induced "BRCAness" and PARP inhibition are synthetically lethal for castration-resistant prostate cancer. Sci Signal 2017; 10:eaam7479.

090177e19c2fb240\Approved\Approved On: 23-Dec-2022 10:25 (GMT)

44. Polkinghorn WR, Parker JS, Lee MX, et al. Androgen receptor signaling regulates DNA repair in prostate cancers. Cancer Discov 2013; 3(11):1245-53.

45. Chakraborty G, Armenia J, Mazzu YZ, et al. Concurrent deletion of BRCA2 and RB1 and aggressive prostate cancer. J Clin Oncol 2018; 36(6):241.

46. Kounatidou E, Nakjang S, McCracken SRC, Dehm SM, Robson CN, Jones D, Gaughan L. A novel CRISPR-engineered prostate cancer cell line defines the AR-V transcriptome and identifies PARP inhibitor sensitivities. Nucleic Acids Res. 2019 Apr 22. pii: gkz286. doi: 10.1093/nar/gkz286. [Epub ahead of print].

47. Schiewer MJ, Goodwin JF, Han S, et al. Dual roles of PARP-1 promote cancer growth and progression. Cancer Discov 2012; 2(12):1134-49.

48. Cancer Discov. 2018 Aug;8(8):OF4. doi: 10.1158/2159-8290.CD-NB2018-084. Epub 2018 Jun 28.

49. N England Journal of Medicine 2018; published online Aug 15. DOI:10.1056/NEJMoa1802905.

50. Gibbons JA, Ouatas T, et al. Clinical pharmacokinetic studies of enzalutamide. Clin Pharmacokinet. 2015; 54: 1043-1055.

51. Scher HI, Fizazi K, Saad F, et al. Increased survival with enzalutamide in prostate cancer after chemotherapy. N Engl J Med. 2012;367(13):1187–1197. Pivotal trial that demonstrate a survival advantage with enzalutamide in chemotherapy-pretreated patients, leading to drug approval.

52. N Engl J Med 2018; 378:2465-2474.

53. Asim M, Tarish F, Zecchini HI, et al. Synthetic lethality between androgen receptor signalling and the PARP pathway in prostate cancer. Nat Commun 2017; 8.

54. Lorente D1,2, Olmos D3, Mateo J2, Dolling D2, Bianchini D2, Seed G2, Flohr P2, Crespo M2, Figueiredo I2, Miranda S2, Scher HI4, Terstappen LWMM5, de Bono JS2. Circulating Tumor Cell Increase as a Biomarker of Disease Progression in Metastatic Castration-Resistant Prostate Cancer Patients with Low Baseline CTC Counts. Ann Oncol. 2018 May 7. doi: 10.1093/annonc/mdy172.

55. https://www.fda.gov/Drugs/DevelopmentApprovalProcess/DevelopmentResources/DrugInteractionsLabeling/ucm093664.htm#table5-2.

56. https://www.druginteractioninfo.org/.

090177e19c2fb240\Approved\Approved On: 23-Dec-2022 10:25 (GMT)

57. Beer TM, Armstrong AJ, Rathkopf D, Loriot Y, Sternberg CN, Higano CS, et al. Enzalutamide in Men with Chemotherapy-naïve Metastatic Castration-resistant Prostate Cancer: Extended Analysis of the Phase 3 PREVAIL Study. Eur Urol. 2017 Feb;71(2):151-154.

58. Scher HI, Morris MJ, Stadler WM, Higano C, Basch E, Fizazi K, et al. Trial design and objectives for castration-resistant prostate cancer: updated recommendations from the Prostate Cancer Clinical Trials Working Group 3. J Clin Oncol. 2016 Apr 20;34(12):1402-18.

59. **The Prostate Cancer Working Group 3 (PCWG3) consensus for trials in castration-resistant prostate cancer (CRPC)**. Howard I. Scher, Michael J. Morris, Walter Michael Stadler, Celestia S. Higano, Susan Halabi, Matthew Raymond Smith, Ethan M. Basch, Karim Fizazi, Charles J. Ryan, Emmanuel S. Antonarakis, Paul Gettys Corn, Glenn Liu, Johann Sebastian De Bono, Lawrence H. Schwartz, Tomasz M. Beer, William Kevin Kelly, Maha Hussain, A. Oliver Sartor, Philip W. Kantoff, and Andrew J. Armstrong Journal of Clinical Oncology 2015 33:15_suppl, 5000-5000

090177e19c2fb240\Approved\Approved On: 23-Dec-2022 10:25 (GMT)

17. SUPPORTING DOCUMENTATION AND OPERATIONAL CONSIDERATIONS

17.1. Appendix 1: Abbreviations

The following is a list of abbreviations that may be used in the protocol.

Abbreviation	Term
ADP	adenosine disphosphate
ADT	androgen deprivation therapy
AE	adverse event
ALT	alanine aminotransferase
AML	acute myeloid leukemia
ANC	absolute neutrophil count
Anti-HBc	hepatitis B core antibody
AR	androgen receptor
ASCO	American Society of Clinical Oncology
AST	aspartate aminotransferase
AUC	area under the plasma concentration-time curve
AUC_{τ}	area under the plasma concentration-time curve over the dosing interval (from time zero to 24 hours post-dose)
BBS	Biospecimen Banking System
BCRP	breast cancer resistant protein
BICR	blinded independent central review
BPI-SF	Brief Pain Inventory-Short Form
BRCA	breast cancer
BUN	blood urea nitrogen
C_{max}	maximum plasma concentration
C_{min}	minimum plasma concentration
C_{trough}	lowest plasma concentration before scheduled dose
CAP/CLIA	Certified Authorization Professional/Clinical Laboratory Improvement Act
CI	confidence interval
CK	creatine kinase
CL/F	apparent oral clearance
CL_{ss}/F	apparent oral clearance, calculated when drug is at steady state
COVID-19	coronavirus disease 2019
CR	complete response
CrCL	creatinine clearance
CRF	case report form

090177e19c2fb240\Approved\Approved On: 23-Dec-2022 10:25 (GMT)

Abbreviation	Term
CRPC	castration-resistant prostate cancer
CSA	clinical study agreement
CSF	colony stimulating factors
CSPC	castration-sensitive prostate cancer
CSR	clinical study report
CT	computed tomography/clinical trial
CTA	clinical trial application
CTCs	circulating tumor cells
ctDNA	circulating tumor deoxyribonucleic acid
CTCAE	Common Terminology Criteria for Adverse Events
C_{trough}	lowest plasma concentration of drug directly before next dose
CV	coefficient variation
CYP	cytochrome 450
DDI	drug-drug interaction
DDR	deoxyribonucleic acid damage repair
DILI	drug-induced liver injury
DNA	deoxyribonucleic acid
DU	dispensable unit
E-DMC	External Data Monitoring Committee
eGFR	estimated glomerular filtration rate
EC	ethics committee
ECG	electrocardiogram
ECOG	Eastern Cooperative Oncology Group
E-DMC	external data monitoring committee
EDP	exposure during pregnancy
EMA	European Medicines Agency
EORTC	European Organisation for Research and Treatment of Cancer
EORTC QLQ-C30	European Organisation for Research and Treatment of Cancer cancer-specific global health questionnaire
EORTC QLQ-PR25	European Organisation for Research and Treatment of Cancer disease specific urinary symptoms questionnaire
EQ-5D-5L	European Quality of Life 5-Dimension, 5-Level Scale
EU	European Union
EudraCT	European Clinical Trials Database
FDA	Food and Drug Administration
FFPE	formalin fixed paraffin embedded

090177e19c2fb240\Approved\Approved On: 23-Dec-2022 10:25 (GMT)

Abbreviation	Term
FSH	follicle-stimulating hormone
FU	follow-up
gBRCA	germline breast cancer susceptibility gene
G-CSF	Granulocyte colony-stimulating factor
GCP	Good Clinical Practice
GGT	gamma-glutamyl transferase
GI	gastrointestinal
GM-CSF	Granulocytemacrophage colony stimulating factor
GnRH	gonadotropin-releasing hormone
HBV	hepatitis B virus
HbsAG	hepatitis B surface antigen
HCP	health care professional
HCV	hepatitis C virus
HCV Ab	hepatitis C virus antibody
HER2	human epidermal growth factor receptor 2
HGRAC	Human Genetics Resources Administration of China
HIV	human immunodeficiency virus
HNPC	hormone-naïve prostate cancer
HR	hazard ratio
HRR	homologous recombination repair
HRRm	homologous recombination repair mutational status
HRD	homologous recombination deficiency
HRQL	health-related quality of life
IB	Investigator's Brochure
ICD	informed consent document
ICH	International Conference on Harmonisation
ID	identification
IDMS	isotope dilution mass spectrometry
IND	investigational new drug application
INR	international normalized ratio
IP	investigational product
IRB	institutional review board
IRC	internal review committee
IRT	interactive response technology
ITT	intent-to-treat

090177e19c2fb240\Approved\Approved On: 23-Dec-2022 10:25 (GMT)

Abbreviation	Term
IUD	intrauterine device
IWRS	Interactive Web Response System
K_2EDTA	dipotassium ethylenediaminetetraacetic acid
LDH	lactate dehydrogenase
LFT	liver function test
LPLV	last patient last visit
M0	non-metastatic
M1	metastatic
mCRPC	metastatic castration-resistant prostate cancer
MDRD	Modification of Diet in Renal Disease
MDS	myelodysplastic syndrome
MedDRA	Medical Dictionary for Regulatory Activities
MFS	metastasis-free survival
MnB	meningococcal serogroup B
MRI	magnetic resonance imaging
N/A	not applicable
NCA	non-compartmental analysis
NCI	National Cancer Institute
NGS	next generation sequencing
NHT	novel hormonal therapy
NLCB	no longer clinically benefitting
NM-CRPC	non-metastatic castration-resistant prostate cancer
ORR	Objective response rate
OS	overall survival
P-gp	P-glycoprotein
PARP	poly(adenosine diphosphate-ribose) polymerase
PAS	PK analysis set
PCD	primary completion date
PCT	physician's choice of treatment
PCWG	Prostate Cancer Working Group
PD	pharmacodynamics(s)
PFS	progression-free survival
PGx	pharmacogenomics(s)
PI	principal investigator
PID	patient identification

090177e19c2fb240\Approved\Approved On: 23-Dec-2022 10:25 (GMT)

Abbreviation	Term
PK	pharmacokinetic
PopPK	population pharmacokinetic
PR	partial response
PR-25	Quality of Life Questionnaire Prostate Cancer Module
PSA	prostate-specific antigen
PRO	patient reported outcomes
PT	prothrombin time
QD	one a day
QoL	quality of life
QLQ-PR25	Quality of Life Questionnaire (prostate cancer module)
QLQ-C30	Quality of Life Questionnaire (cancer module)
RB	retinoblastoma
RBC	red blood cell
RECIST	Response Evaluation Criteria in Solid Tumors
RNA	ribonucleic acid
rPFS	radiographic progression free survival
SAE	serious adverse event
SAP	statistical analysis plan
SC	subcutaneous
SCLC	Small cell lung cancer
SmPC	summary of product characteristics
SMQ	standardized MeDRA queries
SOA	schedule of activities
SOP	standard operating procedure
SRE	skeletal related event
SRSD	single reference safety document
SSID	single subject identification
SUSAR	suspected unexpected serious adverse reaction
t½	half-life
TBili	total bilirubin
TEAE	treatment-emergent adverse event
T_{max}	time to reach peak concentration following drug administration
TNBC	triple-negative breast cancer
TNM	classification of Malignant Tumours
TTPP	time to PSA progression

090177e19c2fb240\Approved\Approved On: 23-Dec-2022 10:25 (GMT)

Abbreviation	Term
ULN	upper limit of normal
US	United States
USPI	United States package insert
Vss/F	steady-state volume of distribution
WBC	white blood cell
WHO	World Health Organization

17.2. Appendix 2: France Appendix

This appendix applies to study sites located in France.

17.3. Appendix 3: GCP Training

Prior to enrollment of any subjects, the investigator and any sub-investigators will complete the Pfizer-provided Good Clinical Practice training course ("Pfizer GCP Training") or training deemed equivalent by Pfizer. Any investigators who later join the study will complete the Pfizer GCP Training or equivalent before performing study-related duties. For studies of applicable duration, the investigator and sub-investigators will complete Pfizer GCP Training or equivalent every three years during the term of the study, or more often if there are significant changes to the ICH GCP guidelines or course materials.

17.4. Appendix 4: Investigational Product

No subjects or third-party payers will be charged for investigational product.

090177e19c2fb240\Approved\Approved On: 23-Dec-2022 10:25 (GMT)

17.5. Appendix 5: eGFR Calculation Prior to Randomization

It is important to correctly calculate the eGFR value at screening to assign the starting dose of the blinded therapy (talazoparib/placebo) in IWRS and avoid overdosing or underdosing errors (which should be reported as protocol deviations and medication errors).

In the protocol, a link to the four-variable MDRD equation is provided (www.mdrd.com). The four variables are the serum creatinine level, age, race, and gender. Only this equation must be used by all sites. Before starting to enter the 4 variables into the MDRD equation it is important to contact the local laboratory and clarify which method is used to measure serum creatinine. Two methods for calculating the four-variable MDRD equation are available: a) older method and b) the isotope dilution mass spectrometry (IDMS) method. Most labs are using the IDMS method given that it is more accurate and less subject to bias compared to the older method.

Therefore:

1. Contact the laboratory to confirm which creatinine reference method is used.
2. Go to www.mdrd.com and enter:
    a. The serum creatinine value (remember to select the correct unit);
    b. The age of the patient at randomization;
    c. The race;
    d. Double check the gender is set on male;
    e. If the laboratory confirmed the IDMS method is used, YES is already pre-checked (since the IDMS method should be the most commonly used).

The eGFR value to be used to select the talazoparib starting dose in IWRS is provided under the line: MDRD study equation.

090177e19c2fb240\Approved\Approved On: 23-Dec-2022 10:25 (GMT)

17.6. Appendix 6: China-Specific Requirements

This appendix is relevant only to study sites located in China.

At least 113 mCRPC patients from China should be enrolled into the study per China's registration requirement.

Therefore, considering global enrollment in Cohort 1 of TALAPRO-2 will be completed prior to randomization of 113 patients from China, additional patients will continue to be randomized in an extension China cohort after completion of enrollment into Cohort 1. Patients enrolled in China in Cohort 1 and in the China extension cohort will be pooled together to form the China cohort (see Figure 3).

Figure 3. China Cohort

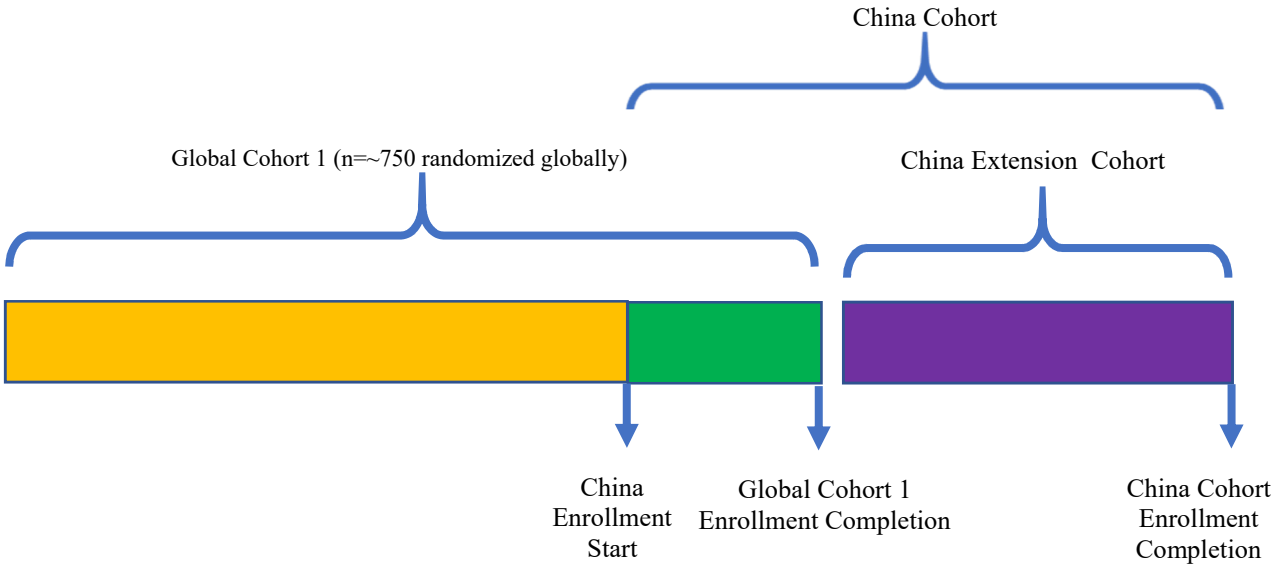

Additional sites will be opened in China. Refer to the body of the protocol for study endpoints, eligibility, and assessments required. No changes from prior requirements will be instituted with this Amendment 7.

Data analysis of primary and secondary study endpoints will not include patients enrolled in this China extension cohort. Data from the China cohort will be analyzed separately per local regulatory requirement. The details of the analysis will be provided in a supplemental SAP.

090177e19c2fb240\Approved\Approved On: 23-Dec-2022 10:25 (GMT)

17.7. Cohort Appendix 7: Country-Specific Requirements

Three country amendments were finalized for this protocol:

Amendment 3: country amendment for France.

Amendment 5: country amendment for South Africa.

Amendment 7: country amendment for China.

Language required by France and South Africa is included in the body of the protocol while language introducing the extension cohort in China is included only in Appendix 6.

090177e19c2fb240\Approved\Approved On: 23-Dec-2022 10:25 (GMT)
