# Supplementary material for: First-line talazoparib with enzalutamide in HRR-deficient metastatic castration-resistant prostate cancer: the phase 3 TALAPRO-2 trial
Source: Nat Med. 2023 Dec 4;30(1):257–64. doi: 10.1038/s41591-023-02704-x (PMC10803259; doi:10.1038/s41591-023-02704-x)
Supplement: Supplementary file 2 — Reporting Summary [file 41591_2023_2704_MOESM2_ESM.pdf]

nature portfolio

Corresponding author(s): Karim Fizazi

Last updated by author(s): Nov 8, 2023

Reporting Summary

Nature Portfolio wishes to improve the reproducibility of the work that we publish. This form provides structure for consistency and transparency in reporting. For further information on Nature Portfolio policies, see our Editorial Policies and the Editorial Policy Checklist.

Statistics

For all statistical analyses, confirm that the following items are present in the figure legend, table legend, main text, or Methods section.

n/a	Confirmed	
☐	☒	The exact sample size (*n*) for each experimental group/condition, given as a discrete number and unit of measurement
☐	☒	A statement on whether measurements were taken from distinct samples or whether the same sample was measured repeatedly
☐	☒	The statistical test(s) used AND whether they are one- or two-sided<br>*Only common tests should be described solely by name; describe more complex techniques in the Methods section.*
☐	☒	A description of all covariates tested
☐	☒	A description of any assumptions or corrections, such as tests of normality and adjustment for multiple comparisons
☐	☒	A full description of the statistical parameters including central tendency (e.g. means) or other basic estimates (e.g. regression coefficient) AND variation (e.g. standard deviation) or associated estimates of uncertainty (e.g. confidence intervals)
☐	☒	For null hypothesis testing, the test statistic (e.g. *F*, *t*, *r*) with confidence intervals, effect sizes, degrees of freedom and *P* value noted<br>*Give P values as exact values whenever suitable.*
☒	☐	For Bayesian analysis, information on the choice of priors and Markov chain Monte Carlo settings
☐	☒	For hierarchical and complex designs, identification of the appropriate level for tests and full reporting of outcomes
☒	☐	Estimates of effect sizes (e.g. Cohen's *d*, Pearson's *r*), indicating how they were calculated

Our web collection on statistics for biologists contains articles on many of the points above.

Software and code

Policy information about availability of computer code

Data collection	Oracle Clinical Remote Data Capture was used for data collection.
Data analysis	SAS version 9.4 was used for the data analysis.

For manuscripts utilizing custom algorithms or software that are central to the research but not yet described in published literature, software must be made available to editors and reviewers. We strongly encourage code deposition in a community repository (e.g. GitHub). See the Nature Portfolio guidelines for submitting code & software for further information.

Data

Policy information about availability of data

All manuscripts must include a data availability statement. This statement should provide the following information, where applicable:

- Accession codes, unique identifiers, or web links for publicly available datasets
- A description of any restrictions on data availability
- For clinical datasets or third party data, please ensure that the statement adheres to our policy

Upon request, and subject to review, Pfizer will provide the data that support the findings of this study. Subject to certain criteria, conditions and exceptions, Pfizer may also provide access to the related individual de-identified participant data. See https://www.pfizer.com/science/clinical-trials/trial-data-and-results for more information.

Research involving human participants, their data, or biological material

Policy information about studies with human participants or human data. See also policy information about sex, gender (identity/presentation), and sexual orientation and race, ethnicity and racism.

Reporting on sex and gender

The study was in patients with metastatic castration-resistant prostate cancer; therefore, all participants were of male sex.

Reporting on race, ethnicity, or other socially relevant groupings

The baseline characteristics, including race, are reported in Table 1. As noted in Table S2 on the representativeness of the study participants, the proportion of Black or African-American patients who underwent randomization was small (3%). Men were enrolled from diverse geographic locations including North America (12%), Europe/United Kingdom (48%), Asia (20% in China, Japan, and Republic of Korea), and the rest of the world (19%).

Population characteristics

The baseline characteristics are reported in Table 1. The age distribution of patients was consistent with that expected, with the majority of patients aged >65 years. This manuscript reports the results in the HRR-deficient population selected for HRR gene alterations; approximately 40% of these had BRCA1/2 gene alterations. More than half of the men received prior first-generation anti-androgen therapy (bicalutamide, flutamide, nilutamide, cyproterone acetate) and approximately 30% received prior docetaxel. Only 8% of patients had received prior abiraterone.

Recruitment

Patients were enrolled in the TALAPRO-2 trial at 223 sites in 26 countries, including the USA, Europe, Israel, South America, South Africa, and the Asia-Pacific region. Part 2 of the TALAPRO-2 trial, including the HRR-deficient population reported in this manuscript, was randomized, double-blind, placebo-controlled, and an independent radiology facility was used to evaluate the primary endpoint (rPFS). Principal investigators selected patients based on their clinical judgment and their ability to ensure that the patients could meet the study requirements. In addition, there was scientific acknowledgement that the participants could benefit overall from the study intervention in alignment with randomized, double-blind, placebo-controlled trial principles. In protocol Amendment No. 6, the inclusion criteria were updated to reflect that HRR gene alteration status for participants randomized in Part 2 was determined by prospective testing using FoundationOne®CDx (tissue) and/or FoundationOne®Liquid CDx. Participants were considered HRR-deficient if the participant had one or more alteration(s) in at least one of the 12 HRR genes or if there was a discordant result between the tissue and liquid results.

Ethics oversight

The trial was conducted in accordance with the International Ethical Guidelines for Biomedical Research Involving Human Subjects, Good Clinical Practice guidelines, the principles of the Declaration of Helsinki, and local laws. The protocol and amendments were approved by the institutional review board and independent ethics committee for each site. The following independent ethics committees or Institutional Review Boards provided study approval: Comite de Revision Institucional - Hospital Britanico de Buenos Aires, CABA, Argentina; Comite de Etica "Dr. Claude Bernard", Rosario, Argentina; Comite de Etica en Investigacion - Centro de Educacion Medica e Investigaciones Clinicas "Norberto Quirno" – CEMIC, CABA, Argentina; Comite de Etica en Investigacion de la Fundacion OncoSalud (CEIFOS), Pergamino, Argentina; Comite Independiente De Etica Para Ensayos En Farmacologia Clinica, CABA, Argentina; Comite Institucional de Etica de la Investigacion en Salud (C.I.E.I.S) de la Clinica Universitaria Reina Fabiola, Cordoba, Argentina; Comite Institucional de Etica de Investigacion en Salud del Hospital Privado Centro Medico de Cordoba, Cordoba, Argentina; St Vincent's Hospital Human Research Ethics Committee, Darlinghurst, Australia; Bellberry Limited, Eastwood, Australia; Commissie Voor Medische Ethiek, Gent, Belgium; Comissao Nacional de Etica em Pesquisa/CONEP, Brasilia, Brazil; Comite de Etica em Pesquisa da Fundacao Pio XII - Hospital de Cancer de Barretos, Barretos, Brazil; Comite de Etica em Pesquisa da Universidade do Vale do Taquari – UNIVATES, Lajeado, Brazil; Comite de Etica em Pesquisa do Hospital Mae de Deus, Porto Alegre, Brazil; Comite de Etica em Pesquisa do Instituto D'Or de Pesquisa e Ensino, Rio de Janeiro, Brazil; Comite de Etica em Pesquisa-Hospital Universitario Pedro Ernesto, Rio de Janeiro, Brazil; Comite de Etica em Pesquisa da Universidade Regional do Noroeste do estado do Rio Grande do Sul, Ijui, Brazil; Comite de Etica em Pesquisa - CEP do Hospital das Clinicas da Faculdade de Medicina da Universidade de Sao Paulo - HCFMUSP, Sao Paulo, Brazil; Comite de Etica em Pesquisa da Faculdade de Medicina do ABC, Santo Andre, Brazil; Comitê de Ética em Pesquisa do Instituto Nacional de Câncer Jose Alencar Gomes da Silva – INCA, Rio de Janeiro, Brazil; Comite de Etica em Pesquisa do Hospital Nossa Senhora da Conceicao - Grupo Hospitalar Conceicao, Porto Alegre, Brazil; Comite de Etica em Pesquisa da Sociedade Beneficente de Senhoras Hospital Sirio Libanes, Sao Paulo, Brazil; Comitê de Ética em Pesquisa do Hospital Alemão Oswaldo Cruz – SP, Sao Paulo, Brazil; Comite de Etica em Pesquisa da Pontificia Universidade Catolica do Rio Grande do Sul-PUC/RS, Porto Alegre, Brazil; Comite d'ethique de la recherche du CHUM, Montreal, Canada; Health Research Ethics Board of Alberta - Cancer Committee, Edmonton, Canada; Ontario Cancer Research Ethics Board, Toronto, Canada; Comite de Etica Cientifico Servicio de Salud Metropolitano Oriente, Santiago, Chile; Comite Etico Cientifico Hospital Dr. Gustavo Fricke Servicio de Salud Vina del Mar – Quillota, Vina del Mar, Chile; Comite de Etica Cientifica Servicio Salud Araucania Sur, Temuco, Chile; Ethics Committee of Zhongshan Hospital Fudan University, Shanghai, China; Ethics Committee of The First Affiliated Hospital of Xi'an Jiaotong University, Xi'an, China; Ethics committee of Zhejiang Cancer Hospital, Hangzhou, China; Ethics Committee of National Cancer Center/ Cancer Hospital, Chinese Academy of Medical Sciences and Peking Union Medical College, Beijing, China; Ethics Committee of Chongqing University Cancer Hospital, Chongqing, China; Ethics Committee of Beijing Cancer Hospital, Beijing, China; Ethics Committee of The First Affiliated Hospital of Anhui Medical University, Hefei, China; Medical Ethics Committee of First Affiliated Hospital of Xiamen University, Xiamen, China; Wuxi People's Hospital Ethics Committee, Wuxi, China; Ethics Committee of Nanjing Drum Tower Hospital, Nanjing, China; Ethics Committee of Shanghai Tenth People's Hospital, Shanghai, China; Clinical Trial Ethics Committee of Huazhong University of Science and Technology, Wuhan, China; Ethics Committee of Huashan Hospital, Fudan University, Shanghai, China; Ethics Committee of Fudan University Cancer Hospital, Shanghai, China; Ethics Committee of Beijing Hospital, Beijing, China; Ethics Committee of Peking University First Hospital, Beijing, China; Peking University Third Hospital Medical Science Research Ethics Committee, Beijing, China; Ethics Committee for Clinical Trials of Drugs (Medical Apparatus) of Ningbo First Hospital, Ningbo, China; Ethics Committee of Ruijin Hospital Affiliated to Shanghai Jiaotong University School of Medicine, Shanghai, China; West China Hospital of Sichuan University Clinical Trial Ethics Committee, Chengdu, China; EC of Second Affiliated Hospital of Suzhou University, Suzhou, China; Shanghai General Hospital Medical Ethics Committee, Shanghai, China; Ethics Committee of Nanjing First Hospital, Nanjing, China; Clinical Trial Ethics Committee of Huazhong University of Science and Technology, Wuhan, China; Drug and Machinery Clinical trial Branch of EC of The First Affil. Hosp. of Fujian Med. University, Fuzhou, China; Ethics Committee of Yunnan Cancer Hospital, Kunming, China; The First Affiliated Hospital of Nanchang

University Ethics Committee, Nanchang, China; Jilin Cancer Hospital Institutional Review Board, Changchun, China; Ethics committee of The Second Hospital of Tianjin Medical University, Tianjin, China; Ethics Committee of Zhejiang Provincial People's Hospital, Hangzhou, China; Ethics Committee of The Fifth People's Hospital of Shanghai, Fudan University, Shanghai, China; Ethics Committee of Nantong Tumor Hospital, Nantong, China; Medical Ethics Committee of The First People's Hospital of Lianyungang, Lianyungang, China; The Clinical Trial Ethics Committee of The First Affiliated Hospital of Wenzhou Medical University, Wenzhou, China; Eticka komise Krajska zdravotni a.s., Masarykova nemocnice v Usti nad Labem, Usti and Labem, Czech Republic; Eticka komise pro multicentricke klinicke hodnoceni Fakultni nemocnice Kralovske Vinohrady, Praha, Czech Republic; Eticka komise Fakultni Nemocnice Ostrava, Ostrava-Poruba, Czech Republic; Eticka komise Fakultni nemocnice Hradec Kralove, Hradec Kralove, Czech Republic; Helsingin ja Uudenmaan sairaanhoitopiiri, Helsinki, Finland; Comite De Protection Des Personnes (CPP) Sud-Ouest Et Outre-Mer III, Bordeaux, France; Ethikkommission der Aerztekammer Hamburg, Hamburg, Germany; Egészségügyi Tudományos Tanács Klinikai Farmakológiai Etikai Bizottsága, Budapest, Hungary; Bnai Zion Medical Center Helsinki Committee, Haifa, Israel; Rambam Health Care Campus Helsinki Committee, Haifa, Israel; Tel Aviv Sourasky Medical Center Helsinki Committee, Tel Aviv, Israel; Rabin Medical Center Helsinki Committee, Petah Tikva, Israel; Shaare Zedek Medical Center Helsinki Committee, Jerusalem, Israel; Comitato Etico Azienda Ospedaliero Universitaria San Luigi Gonzaga, Orbassano, Italy; Comitato Etico Val Padana, Cremona, Italy; Comitato Etico Regionale (CER) dell'Umbria, Perugia, Italy; Comitato Etico Cardarelli-Santobono, Napoli, Italy; Comitato Etico Per Le Sperimentazioni Cliniche Dell'Azienda Provinciale Per I Servizi Sanitari, Trento, Italy; Comitato Etico della Romagna (CEROM), Meldola, Italy; Comitato Etico di Brescia, Brescia, Italy; Comitato Etico di Area Vasta Emilia Centro, Bologna, Italy; Comitato Etico IRCCS Pascale, Napoli, Italy; National Hospital Organization Central Review Board, Meguro-ku, Tokyo, Japan; National Cancer Center IRB, Chuo-ku, Tokyo, Japan; Kindai University Hospital Institutional Review Board, Osakasayama, Japan; Yokohama City University Medical Center IRB, Yokohama, Japan; Keio University Hospital Institutional Review Board, Shinjuku-ku, Tokyo, Japan; Nagoya University Hospital IRB, Nagoya, Japan; Hokkaido University Hospital Institutional Review Board, Sapporo, Japan; Tokushima University Hospital Institutional Review Board, Tokushima, Japan; Chiba Cancer Center Institutional Review Board, Chiba, Japan; Hirosaki University School of Medicine & Hospital IRB, Hirosaki, Japan; Yamagata Prefectural Central Hospital Institutional Review Board, Yamagata, Japan; Yokosuka Kyosai Hospital Institutional Review Board, Yokosuka, Japan; Hamamatsu University School of Medicine, University hospital Institutional Review Board, Hamamatsu, Japan; Osaka International Cancer Institute Institutional Review Board, Osaka-shi, Japan; Osaka University Hospital Institutional Review Board, Suita, Japan; Kanazawa University Hospital IRB, Kanazawa, Japan; Kagoshima University Hospital Institutional Review Board, Kagoshima, Japan; Yamagata University Hospital Institutional Review Board, Yamagata, Japan; Kyungpook National University Chilgok Hospital Institutional Review Board, Daegu, Republic of Korea; Samsung Medical Center Institutional Review Board, Seoul, Republic of Korea; Asan Medical Center Institutional Review Board, Seoul, Republic of Korea; Severance Hospital, Yonsei University Health System Institutional Review Board, Seoul, Republic of Korea; Pusan National University Hospital Institutional Review Board, Busan, Republic of Korea; Seoul National University Hospital Institutional Review Board, Seoul, Republic of Korea; National Cancer Center Institutional Review Board, Goyang-si, Republic of Korea; The Catholic University of Korea Seoul St. Mary's Hospital Institutional Review Board, Seoul, Republic of Korea; Health and Disability Ethics Committee, Wellington, New Zealand; REK Sor-Ost, Oslo, Norway; Comite Institucional de Etica en Investigacion del INEN, Lima, Peru; Comite Institucional de Bioetica de Via Libre, Lima, Peru; Komisja Bioetyczna przy Okregowej Izbie Lekarskiej w Gdansku, Gdansk, Poland; Comissao de Etica para a Investigacao Clinica, Lisboa, Portugal; University of the Witwatersrand Human Research Ethics Committee (Medical), Johannesburg, South Africa; CEIm del Hospital Universitari Vall d'Hebron, Barcelona, Spain; Etikprovningsmyndigheten, Uppsala, Sweden; Health and Care Research Wales, Wales REC 3, Cardiff, United Kingdom; Advarra IRB, Columbia, MD, United States; Vanderbilt Human Research Protection Program (VHRPP) IRB, Nashville, TN, United States; University of Utah Institutional Review Board, Salt Lake City, UT, United States; Biomedical Research Alliance of New York, LLC / Institutional Review Board, Lake Success, NY, United States; Western Institutional Review Board, Puyallup, WA, United States; Sharp HealthCare Institutional Review Board, San Diego, CA, United States; Schulman Associates Institutional Review Board, Cincinnati, OH, United States; Loma Linda University Health - Institutional Review Board, Loma Linda, CA, United States; Administrative Panels on Human Subjects in Medical Research ("Stanford IRB"), Palo Alto, CA, United States; University of Maryland, Baltimore - Institutional Review Board, Baltimore, MD, United States; Cook County Health Office of Research and Regulatory Affairs, Chicago, IL, United States; Samaritan Health Services Regional Institutional Review Board, Corvallis, OR, United States; University of Iowa IRB-01, Human Subjects Office, Iowa City, IA, United States; Lakeland Regional Medical Center, Inc. IRB, Lakeland, FL, United States; VA Med Ctr, Long Beach CA IRB #1, Long Beach, CA, United States; Rush University Medical Center Institutional Review Board, Chicago, IL, United States; UCLA Office of the Human Research Protection Program, Los Angeles, CA, United States; VA Saint Louis Healthcare System Institutional Review Board, St. Louis, MO, United States; Baylor Scott and White Research Institute Institutional Review Board-Gold, Temple, TX, United States; Providence St. Joseph Health IRB, Renton, WA, United States; IntegReview, Austin, TX, United States; Kaiser Permanente Northwest Institutional Review Board, Portland, OR, United States; Ochsner Institutional Review Board, New Orleans, LA, United States; Eisenhower Medical Center, Institutional Review Board, Rancho Mirage, CA, United States. The full list of TALAPRO-2 investigators is included in the Supplementary Information table. All patients provided written informed consent.

Note that full information on the approval of the study protocol must also be provided in the manuscript.

Field-specific reporting

Please select the one below that is the best fit for your research. If you are not sure, read the appropriate sections before making your selection.

☒ Life sciences ☐ Behavioural & social sciences ☐ Ecological, evolutionary & environmental sciences

For a reference copy of the document with all sections, see nature.com/documents/nr-reporting-summary-flat.pdf

Life sciences study design

All studies must disclose on these points even when the disclosure is negative.

Sample size: Sample size and power calculation were based on the log-rank test. For the primary comparison in the HRR-deficient population, 224

Sample size	progression-free survival events would provide 85% power to detect a hazard ratio of 0.64 using a one-sided stratified log-rank test at a significance level of 0.0125 with two pre-specified interim analyses based on Lan-DeMets α–spending and β-spending functions. Approximately 380 patients with HRR gene alterations were to be enrolled.
Data exclusions	Inclusion criteria for the TALAPRO-2 trial were pre-specified and previously published (Agarwal N et al. Future Oncol 2022;18(4):425-436).
Replication	Part 2 of TALAPRO-2 was a randomized, double-blind, placebo-controlled trial including a large number (N=399) of patients with HRR-deficient mCRPC. The FoundationOne®CDx and/or FoundationOne®Liquid CDx next-generation sequencing test was used for prospective assessment of HRR gene alterations. Enrollment of patients with ATM and/or CDK12 gene alterations was paused between January–November 2021 as their observed prevalence exceeded expectations and was anticipated to suppress representation of alterations in the remaining genes under study. The pause in enrollment of patients with ATM and/or CDK12 gene alterations was driven by expected prevalence numbers based on the largest and most comprehensive prospective assessment of prostate cancer tumor samples using the FoundationOne® Assay. This pause occurred in a blinded fashion regarding distribution of HRR alterations to the two treatment arms and allowed a rebalancing of the distribution across the 12 gene panel in an effort to best reflect the prevalence in mCRPC.
Randomization	Participants were randomized 1:1 by site personnel using a centralized Interactive Web Response System to talazoparib plus enzalutamide or matching placebo plus enzalutamide. Randomization was stratified by prior novel hormonal therapy or docetaxel for castration-sensitive prostate cancer.
Blinding	The sponsor, participants, and investigators were blinded to talazoparib or placebo during data collection, while enzalutamide was open-label (both treatment groups received enzalutamide). The blinding methodology in TALAPRO-2 prevented selection or ascertainment (i.e., information) biases and potentially improved the compliance and the retention of trial participants. In TALAPRO-2 the withholding of information about the assigned interventions from participants involved in the trial played a potentially major role in mitigating threats to the internal validity of the trial outcomes.

Reporting for specific materials, systems and methods

We require information from authors about some types of materials, experimental systems and methods used in many studies. Here, indicate whether each material, system or method listed is relevant to your study. If you are not sure if a list item applies to your research, read the appropriate section before selecting a response.

Materials & experimental systems

n/a	Involved in the study	
☒	☐	Antibodies
☒	☐	Eukaryotic cell lines
☒	☐	Palaeontology and archaeology
☒	☐	Animals and other organisms
☐	☒	Clinical data
☒	☐	Dual use research of concern
☒	☐	Plants

Methods

n/a	Involved in the study	
☒	☐	ChIP-seq
☒	☐	Flow cytometry
☒	☐	MRI-based neuroimaging

Clinical data

Policy information about clinical studies
All manuscripts should comply with the ICMJE guidelines for publication of clinical research and a completed CONSORT checklist must be included with all submissions.

Clinical trial registration	NCT03395197
Study protocol	A redacted version of the protocol will be available as part of the Supplementary Information.
Data collection	Data collection occurred at each study site (study start date: Dec 18, 2017; primary completion date: Oct 3, 2022 [NCT03395197; ClinicalTrials.gov]). Patients from 223 sites in 26 countries, including the USA, Europe, Israel, South America, South Africa, and the Asia-Pacific region were enrolled in the TALAPRO-2 trial (Agarwal N et al. Future Oncol 2022;18(4):425-436).
Outcomes	The primary endpoint was rPFS by blinded independent central review per RECIST 1.1 and PCWG3. Key secondary endpoints were overall survival, objective response rate, duration of soft tissue response, time to PSA progression, PSA response, PFS2 (investigator-assessed), safety, patient-reported outcomes, and pharmacokinetics.
